# Functional lncRNA-miRNA-mRNA Networks in Response to Baicalein Treatment in Hepatocellular Carcinoma

**DOI:** 10.1155/2021/8844261

**Published:** 2021-01-14

**Authors:** Xin Zhao, Dongyang Tang, Xiaofei Chen, Shaoqing Chen, Cheng Wang

**Affiliations:** ^1^Department of Pharmacy, Xinxiang Central Hospital, Xinxiang, China; ^2^Department of Experimental Management Center, Henan Institute of Science and Technology, Xinxiang, China; ^3^Clinical Laboratory, The People's Hospital of Suzhou New District, Suzhou, China; ^4^College of Pharmaceutical Sciences, Zhejiang University, Hangzhou, China; ^5^School of Pharmaceutical Engineering & Life Science, School of Nursing, Changzhou University, Changzhou, China

## Abstract

**Introduction:**

Baicalein has been shown to have antitumor activities in several cancer types. However, its acting mechanisms remain to be further investigated. This work is aimed at exploring the functional long noncoding RNA (lncRNA)/microRNA (miRNA)/messenger RNA (mRNA) triplets in response to baicalein in hepatocellular carcinoma (HCC) cell to understand the mechanisms of baicalein in HCC.

**Methods:**

Differentially expressed lncRNAs (DELs) and miRNAs (DEMs) in HCC cell treated with baicalein were first screened using GSE95504 and GSE85511, respectively. miRNA targets for DELs were predicted and intersected with DEMs, after which the miRNA expression was validated using ENCORI and its prognostic value was assessed using Kaplan-Meier plotter. Potential miRNA targets were predicted by 3 prediction tools, after which expression level was validated at UALCAN and Human Protein Atlas. Kaplan-Meier plotter was used to evaluate the effects of these genes on overall survival and recurrence-free survival of HCC patients. Enrichment analyses for these genes were performed at DAVID.

**Results:**

Here, we identified 14 overlapping DELs and 26 overlapping DEMs in the baicalein treatment group than those in the DMSO treatment group. Subsequently, by analyzing expression and clinical significance of miRNAs, hsa-miR-4443 was found as a highly potential miRNA target. Then, targets of hsa-miR-4443 were predicted and analyzed, and we found AKT1 was the most potential target for hsa-miR-4443. Hence, the lncRNAs-hsa-miR-4443-AKT1 axis that can respond to baicalein was established.

**Conclusion:**

Collectively, we elucidated a role of lncRNAs-hsa-miR-4443-AKT1 pathway in response to baicalein treatment in HCC, which could help us understand the roles of baicalein in inhibiting cancer progression and may provide novel insights into the mechanisms behind HCC progression.

## 1. Introduction

Hepatocellular carcinoma (HCC) is the main type of liver cancer and with a high recurrence and morbidity rate, which is a heavy health burden nowadays [1]. It is now clear that exposure to aflatoxin, alcohol history, and infection of hepatitis B virus or hepatitis C virus can increase the risk of HCC [2]. For the further malignant development of HCC, genetic and epigenetic changes will occur [3]. HCC at early stages can be treated with surgical resection method; most of them can experience recurrence [4]. It should be noticed that Chinese herbs are valuable resources for the development of novel cancer treatment reagents and have been shown that these could efficiently inhibit tumor growth and metastasis with a relative low toxicity [5, 6].

Baicalein is an active flavonoid compound extracted from root of a traditional Chinese herb, *Scutellaria baicalensis Georgi* [7]. In recent years, baicalein has been proved to have various pharmacological roles in human including anti-inflammatory, anticancer effect, and antioxidant [8–10]. In nasopharyngeal carcinoma, the treatment of baicalein can reverse the radioresistance of cancer cell via downregulating autophagy [11]. In prostate cancer, baicalein was found that it could suppress cancer growth by arresting cell cycle and stimulating cell apoptosis via regulating the CDK6/FOXM1 axis [12]. The findings in recent decades have proved that Chinese herbs including curcumin and paclitaxel exert anticancer roles by disrupting the expression of noncoding RNAs (ncRNAs) [13, 14]. However, it is unclear to date whether baicalein also exerts its effects on tumor progression by affecting ncRNA expression.

The improvements of high-throughput sequencing technology have revealed that more than 90% of human genome transcripts did not encode proteins or peptides, which are called ncRNAs [15]. ncRNAs can be generally classified into two types based on nucleotides length: long ncRNAs (lncRNAs) and microRNAs (miRNAs). Aberrant expressions of ncRNAs including pseudogenes, lncRNAs, and miRNAs have been widely studied in HCC with a large number of ncRNAs been identified [16–18]. Importantly, the competing endogenous RNA (ceRNA) theory has linked the functions of protein-coding genes and noncoding genes in diseases [19].

In this work, we downloaded the lncRNA and miRNA expression data of HCC cell Bel-7402 after baicalein treatment from Gene Expression Omnibus (GEO, https://www.ncbi.nlm.nih.gov/gds/). Differentially expressed lncRNAs (DELs) and miRNAs (DEMs) were screened out to construct lncRNA-miRNA-mRNA networks that can respond to baicalein treatment in HCC.

## 2. Materials and Method

### 2.1. Data Collection

We downloaded the microarray datasets GSE95504 (https://www.ncbi.nlm.nih.gov/geo/query/acc.cgi?acc=GSE95504) and GSE85511 (https://www.ncbi.nlm.nih.gov/geo/query/acc.cgi?acc=GSE85511) from GEO. GSE95504 data was based on GPL17586 ([HTA-2_0] Affymetrix Human Transcriptome Array 2.0 [transcript (gene) version]). GSE85511 data was based on GPL21572 ([miRNA-4] Affymetrix Multispecies miRNA-4 Array [ProbeSet ID version]). These two datasets both contain three groups treated with DMSO, 40 *μ*M baicalein, and 80 *μ*M baicalein, respectively. Each group contains three replicates.

### 2.2. Differential Expression Analysis

DELs and DEMs (40 *μ*M baicalein group vs. DMSO group and 80 *μ*M baicalein group vs. DMSO group) in these two datasets were screened out using limma package in R. Cut-off criteria were set as |log_2_ fold change| > 1 and adj. *P* value <0.05. Volcano plot was drawn using the tool at SangerBox (http://sangerbox.com/AllTools?tool_id=9699135). Veen diagram was drawn using VENNY 2.1.0 (https://bioinfogp.cnb.csic.es/tools/venny/index.html). Heatmap for the overlapping DELs and DEMs was drawn using Heml 1.0.3.7 software.

### 2.3. Probe Annotation and Identification of lncRNA

DELs screened out were annotated using the annotation file of GPL17586 provided by the manufacturer and translated to lncRNA transcribe ID using NONCODE (http://www.noncode.org/index.php) [20] and LncBook (https://bigd.big.ac.cn/lncbook/index) [21].

### 2.4. miRNA Target Prediction for lncRNA

miRNA targets for DELs were predicted at LncBook. miRNAs that overlapped with DEMs were selected for following the analyses.

### 2.5. Validation of miRNA Expression Level and Clinical Significance in HCC

Expression levels of these identified miRNAs in HCC tissues and normal tissues were analyzed at ENCORI (http://starbase.sysu.edu.cn/) [22]. After entering the name of gene, the expression box plot was automatically generated and the *P* value was automatically calculated on the webpage. The clinical significance of miRNAs on overall survival of HCC patients was analyzed at Kaplan-Meier plotter (http://kmplot.com/analysis/index.php?p=service&default=true) [23].

### 2.6. mRNA Target Prediction for miRNA

Targets for miRNA were analyzed at TargetScan (http://www.targetscan.org/vert_72/) [24], miRWalk (http://mirwalk.umm.uni-heidelberg.de/) [25], and miRDB (http://www.mirdb.org/) [26]. Those targets predicted by all these algorithms were selected for following the analyses.

### 2.7. Functional Enrichment

To understand the function of these genes, we performed gene ontology (GO) and Kyoto Encyclopedia of Genes and Genomes (KEGG) enrichment at Database for Annotation Visualization and Integrated Discovery (DAVID) (https://david.ncifcrf.gov/) [27].

### 2.8. Construction of lncRNA-miRNA-mRNA Network

Furthermore, we constructed a lncRNA-miRNA-mRNA network that responds to baicalein treatment based on the target prediction results. Then, the network was visualized using Cytoscape software.

### 2.9. Protein-Protein Interaction (PPI) Network Construction

STRING (https://string-db.org/) [28] was employed to analyze PPI interactions of the targets for miRNA. Cytoscape software was used to construct PPI network and analyze the hub genes using the CytoHubba plug-in. The gene in central nodes might be core genes that contribute to HCC progression.

### 2.10. Validation of AKT1, MAPK8, AR, and MDM2 Expression in HCC Tissues and Effect on Overall Survival of HCC Patients

AKT1, MAPK8, AR, and MDM2 expression levels in HCC tissues and normal tissues were analyzed at UALCAN (http://ualcan.path.uab.edu/) [29].

### 2.11. Immunohistochemistry (IHC) Analysis of AKT1 and MAPK8 Expression in HCC

Protein expression level of AKT1 and MAPK8 in HCC tissues and normal liver tissues analyzed by IHC method was analyzed at Human Protein Atlas (https://www.proteinatlas.org/search/akt1) [30].

### 2.12. Clinical Significance of AKT1 in HCC

We explored the clinical significance of AKT1 on overall survival and recurrence-free survival of HCC patients using Kaplan-Meier plotter. We also downloaded the clinical data of 365 HCC patients from TCGA (https://www.cancer.gov/about-nci/organization/ccg/research/structural-genomics/tcga). Univariate and multivariate analyses performed with Cox proportional hazard models were performed at SPSS 22.0 to identify independent factors for overall survival of HCC patients.

### 2.13. Analysis of the Correlation between AKT1 and the Infiltration of Immune Cells

We explored the correlation between the expression of AKT1 in HCC and tumor infiltration immune cells (TIICs) using TIMER online analysis platform [31].

### 2.14. Drug Target Prediction

Since AKT1 was identified as a key gene for HCC progression, we screened the drugs that can target AKT1 using DrugDB [32].

## 3. Results

### 3.1. Identification of DELs after Baicalein Treatment

**Figure 1:**
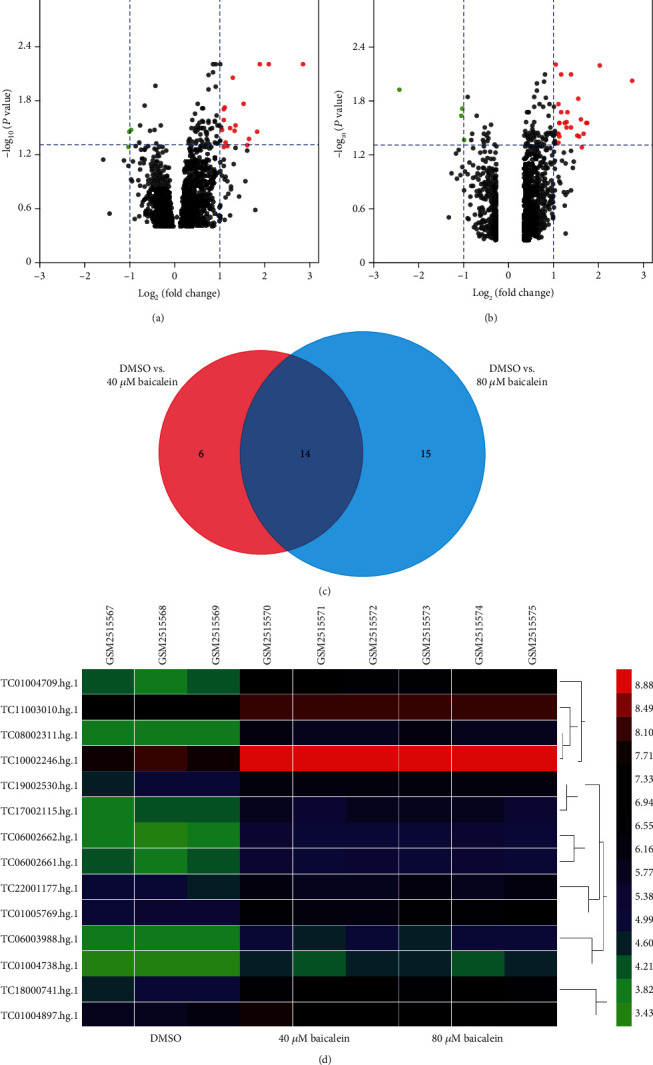
Identification of differentially expressed lncRNAs (DELs) in HCC cell between DMSO and baicalin treatment. (a) Volcano plot showing DELs in HCC cell between the DMSO and 40 *μ*M baicalein groups. (b) Volcano plot showing DELs in HCC cell between the DMSO and 80 *μ*M baicalein groups. (c) Intersection of DELs of “DMSO vs. 40 *μ*M baicalein” and “DMSO vs. 80 *μ*M baicalein.” (d) Heatmap of DELs in HCC cell with DMSO, 40 *μ*M baicalein, or 80 *μ*M baicalein treatment. HCC: hepatocellular carcinoma.

Firstly, DELs at the cells with baicalein or DMSO treatment were explored. 20 DELs in the DMSO vs. 40 *μ*M baicalein group were identified (Figure 1(a)), while 29 DELs were identified in the DMSO vs. 80 *μ*M baicalein group (Figure 1(b)). Venn diagram indicated that there are 14 intersected DELs (Figure 1(c)), and the detailed information of these lncRNAs is listed in Table 1. In addition, heatmap for the expression of these 14 lncRNAs in the DMSO and baicalein treatment groups is shown in Figure 1(d).

### 3.2. Identification of DEMs after Baicalein Treatment

**Figure 2:**
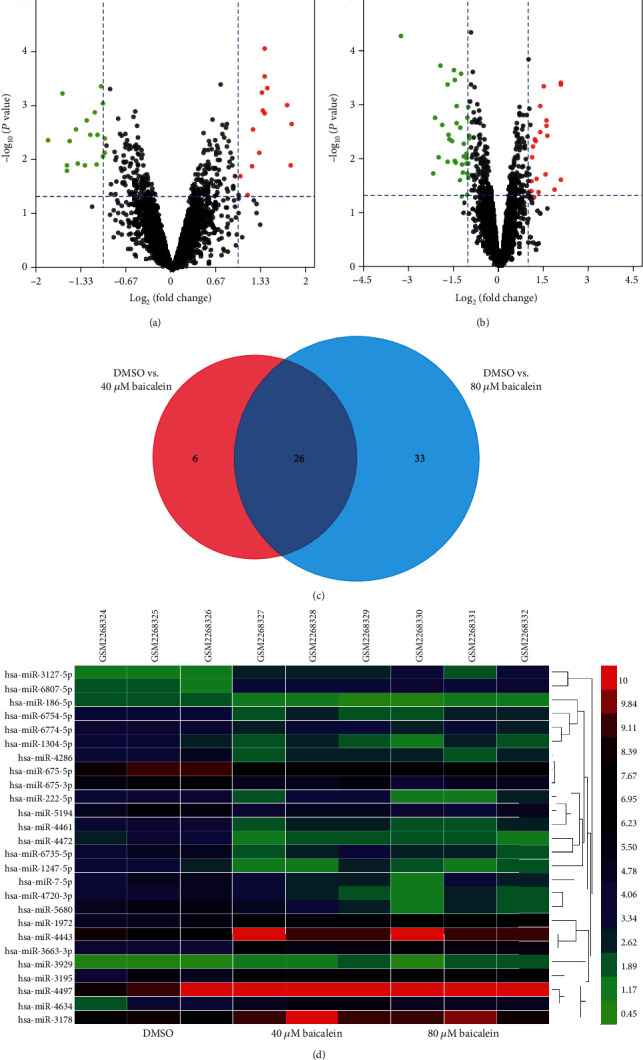
Identification of differentially expressed miRNAs (DEMs) in HCC cell between DMSO and baicalin treatment. (a) Volcano plot showing DEMs in HCC cell between the DMSO and 40 *μ*M baicalein groups. (b) Volcano plot showing DEMs in HCC cell between the DMSO and 80 *μ*M baicalein groups. (c) Intersection of DEMs of “DMSO vs. 40 *μ*M baicalein” and “DMSO vs. 80 *μ*M baicalein.” (d) Heatmap of DEMs in HCC cell with DMSO, 40 *μ*M baicalein, or 80 *μ*M baicalein treatment. HCC: hepatocellular carcinoma.

Then, we screened out the DEMs in cells with baicalein or DMSO treatment. After baicalein treatment, we identified 32 miRNAs in the 40 *μ*M baicalein treatment group (Figure 2(a)), while 59 miRNAs in the 80 *μ*M baicalein treatment group (Figure 2(b)). Furthermore, we showed there are 26 overlapping miRNAs (Figure 2(c)). The detailed information of these 265 miRNAs are presented in Table 2, and their expressions displayed in a heatmap manner are shown in Figure 2(d).

### 3.3. Prediction miRNA Targets of DELs

miRNA targets for the identified 14 DELs were predicted at LncBook (Table S1) and then extracted the overlapped miRNAs with the 26 DEMs for following the analysis. The detailed results are presented in Table 3, and we found that 24 out of 26 DEMs may interact with DELs.

### 3.4. Validation of the Expression of miRNAs Using ENCORI

Furthermore, we explored the expression levels of these 24 miRNAs in HCC tissues and normal tissues using ENCORI. The detailed expression results are presented in Table 4; we showed that 7 out of these 24 miRNAs were indeed abnormally expressed in HCC tissues compared with normal tissues. By comparing miRNA expression level in HCC tissues and in HCC cell after baicalein treatment, hsa-miR-4443 and hsa-miR-675-5p were selected for following the analyses since these two miRNAs have opposite expression pattern in these two conditions.

### 3.5. Validation of the Clinical Significance of hsa-miR-4443 and hsa-miR-675-5p in HCC

**Figure 3:**
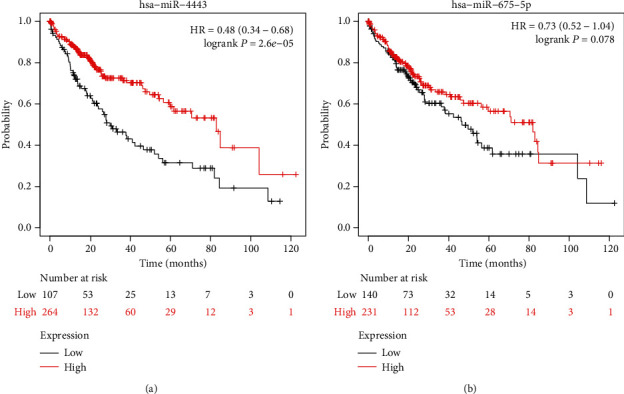
Validation of clinical significance of hsa-miR-4443 and hsa-miR-675-5p in HCC. Effects of (a) hsa-miR-4443 and (b) hsa-miR-675-5p on the overall survival of HCC patients. HCC: hepatocellular carcinoma.

**Figure 4:**
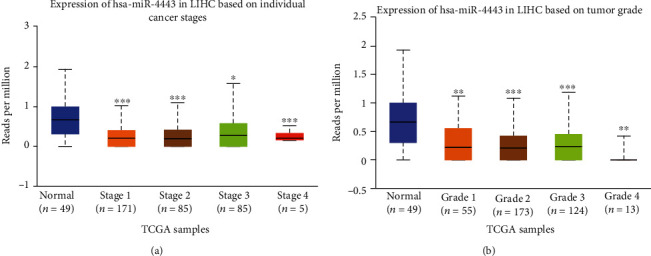
Exploration of hsa-miR-4443 expression in different tumor stage and grade of HCC. (a) hsa-miR-4443 expression level in normal liver tissues and stage 1-4 HCC tissues. (b) hsa-miR-4443 expression level in normal liver tissues and grade 1-4 HCC tissues. HCC: hepatocellular carcinoma.

Then, we explored the effects of hsa-miR-4443 and hsa-miR-675-5p expression on the overall survival of HCC patients using the Kaplan-Meier plotter. We found that low hsa-miR-4443 was a predictor for overall survival of HCC patients (Figure 3(a)), while hsa-miR-675-5p did not have strong correlation with HCC patients' overall survival (Figure 3(b)). To further understand the roles of hsa-miR-4443 expression in HCC, we explored correlation of has-miR-4443 expression and clinical characteristics of HCC patients using Kaplan-Meier plotter database. Low hsa-miR-4443 was associated with worse overall survival in tumor stage at 1 to 3 (Table 5). In addition, low hsa-miR-4443 expression was correlated with overall survival in grades 2 and 3 of HCC patients but was not associated with overall survival in grade 1 (Table 5). In addition, we explored the expression of hsa-miR-4443 in different tumor stages and tumor grades. Our results confirmed that HCC patients at late tumor stage or high tumor grade tend to have low hsa-miR-4443 expression (Figures 4(a) and 4(b)).

### 3.6. Construction of lncRNA-miRNA-mRNA Network

**Figure 5:**
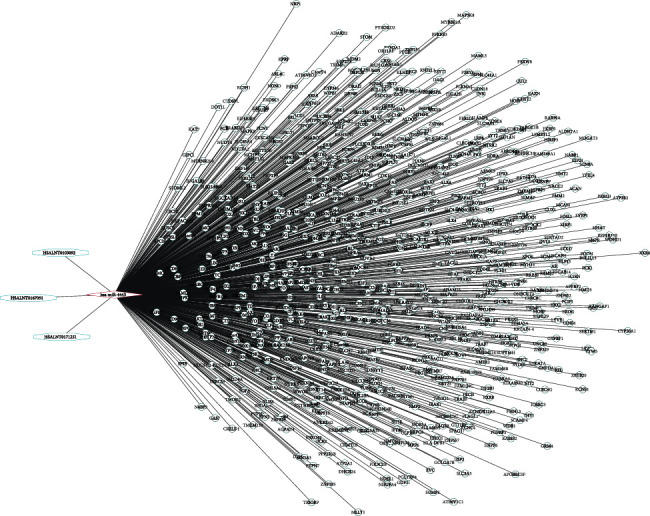
Visualization of the lncRNA-miRNA-mRNA network response to baicalein treatment in HCC. Network is consisted of 3 lncRNAs, 1 miRNA, and 796 mRNAs. HCC: hepatocellular carcinoma.

Then, we predicted the targets of hsa-miR-4443 using three prediction algorithms, TargetScan, miRWalk, and miRDB. A total of 796 overlapping targets for hsa-miR-4443 were identified (Table S2). A lncRNA-miRNA-mRNA network which contains 3 lncRNAs, 1 miRNA, and 796 mRNA was established (Figure 5).

### 3.7. Function Enrichment of the Targets

**Figure 6:**
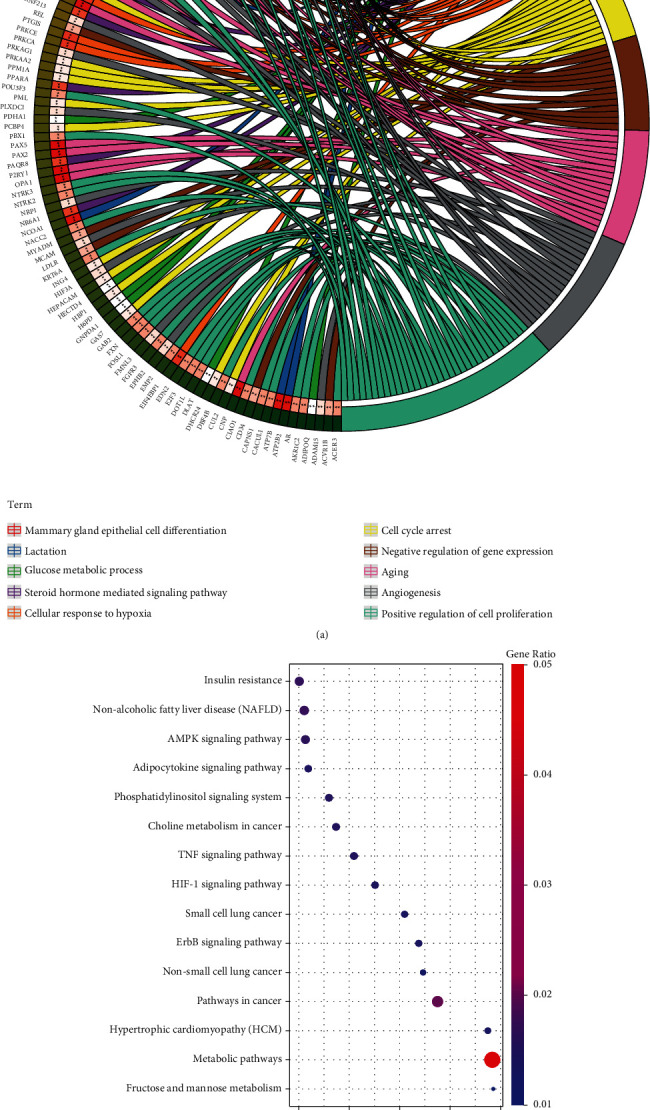
Functional enrichment of the 796 mRNAs identified for hsa-miR-4443. (a) Circos plot showed the top 10 enriched GO terms. (b) Bubble plot showed the top 15 enriched KEGG terms.

Subsequently, we performed GO and KEGG analyses to understand the roles of hsa-miR-4443 targets. As shown in Figure 6(a), we showed 10 enriched biological processes (BP) for the predicted 796 genes and showed AKT1 linked to 4 of 10 BP terms. In addition, KEGG enrichment analysis indicated these genes were also can be enriched in pathways related to cancer including pathways in cancer, non-small-cell lung cancer, small-cell lung cancer, and so on (Figure 6(b)).

### 3.8. Identification of Hub Gene in PPI Network

**Figure 7:**
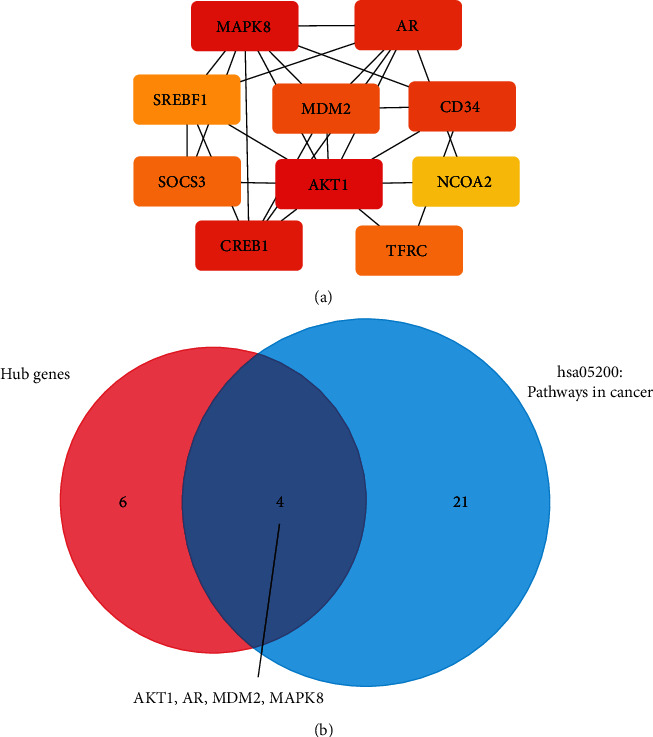
Identification of hub genes in the protein-protein interaction network. (a) Top 10 hub genes identified in the protein-protein interaction network. (b) Intersection of genes of hub genes and genes enriched to the “pathway in cancer”.

PPI network was constructed after analyzing using STRING and visualized at Cytoscape (Figure S1). CytoHubba was used to identify the hub genes in this network (Figure 7(a)), and the detailed information of these top 10 hub genes are shown in Table 6. Subsequently, we selected an enriched pathway (hsa05200: pathways in cancer) and intersected with the top 10 hub genes, and we showed that there are 4 overlapping genes (AKT1, MAPK8, AR, and MDM2) (Figure 7(b)).

### 3.9. Validation of AKT1, MAPK8, AR, and MDM2 Gene Expression and Clinical Significance in HCC

**Figure 8:**
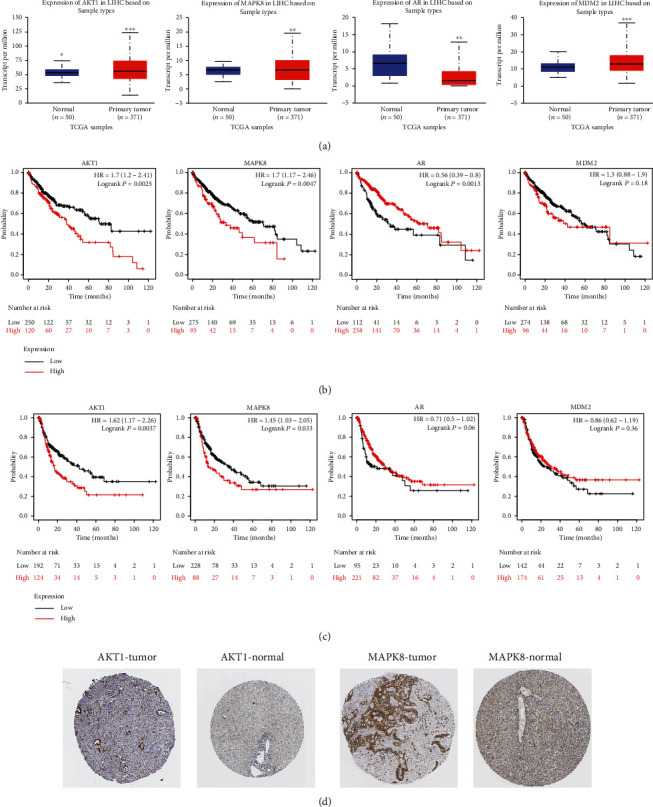
Validation expression and clinical significance of AKT1, MAPK8, AR, and MDM2 in HCC. (a) Expression level of AKT1, MAPK8, AR, and MDM2 in HCC tissues and normal tissues. (b) Effects of AKT1, MAPK8, AR, and MDM2 on overall survival of HCC patients. (c) Effects of AKT1, MAPK8, AR, and MDM2 on recurrence-free survival of HCC patients. (d) Immunohistochemistry assay to indicate AKT1 and MAPK8 protein expression in HCC tissues and normal tissues. HCC: hepatocellular carcinoma.

**Figure 9:**
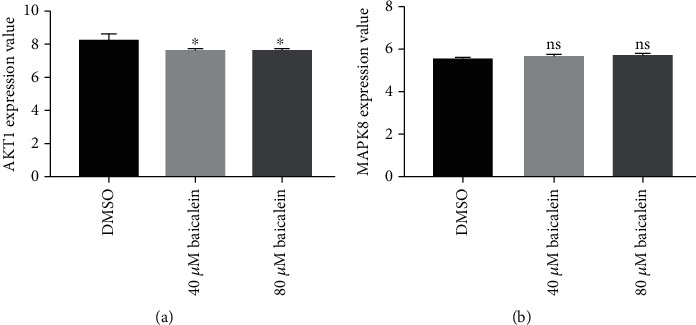
Expression level of AKT1 and MAPK8 in HCC cell after DMSO or baicalein treatment. (a) AKT1 expression level in HCC cell with DMSO, 40 *μ*M baicalein, or 80 *μ*M baicalein treatment. (b) MAPK8 expression level in HCC cell with DMSO, 40 *μ*M baicalein, or 80 *μ*M baicalein treatment. HCC: hepatocellular carcinoma.

UALCAN was used to explore AKT1, MAPK8, AR, and MDM2 expression, and we found that they were all abnormally expressed in HCC tissues compared with normal tissues (Figure 8(a)). However, high AKT1 and MAPK8 were associated with both poorer overall survival and recurrence-free survival of HCC patients (Figures 8(b) and 8(c)). IHC assay confirmed that AKT1 and MAPK8 protein levels were higher in HCC tissues compared than in normal tissues (Figure 8(d)). Interestingly, we showed AKT1 expression level was decreased by baicalin treatment compared with DMSO treatment (Figure 9(a)), whereas MAPK8 expression level was slightly increased by baicalin but the difference was not significant (Figure 9(b)). Hence, AKT1 was selected for following the analyses. As indicated in Table 7, high AKT1 expression was associated with overall survival and recurrence-free survival. Univariate and multivariate analyses showed that AKT1 expression along with tumor stage could be used as independent factors to predict the overall survival of HCC patients (Table 8).

### 3.10. Relationship between AKT1 Expression and TIICs in HCC

**Figure 10:**
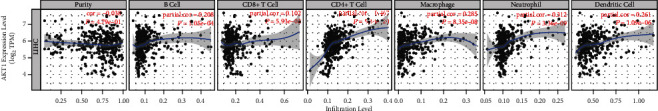
Correlation analysis between AKT1 expression and the infiltration of HCC immune cells. HCC: hepatocellular carcinoma.

We employed TIMER to explore the association of AKT1 expression and TIICs in HCC, and the results in Figure 10 showed that AKT1 was positively correlated with B cell (*r* = 0.208, *P* = 1.05*E* − 4), CD8+ T cell (*r* = 0.102, *P* = 5.93*E* − 2), CD4+ T cell (*r* = 0.467, *P* = 5.34*E* − 20), macrophage (*r* = 0.285, *P* = 8.35*E* − 8), neutrophil (*r* = 0.312, *P* = 3.36*E* − 9), and dendritic cell (*r* = 0.261, *P* = 1.09*E* − 6).

### 3.11. Drugs against AKT1 Predicted at DrugBank

According to DrugBank, the approved or experimental drugs that could act on ACTB and VEGFA as shown in previous studies are summarized in Table 9.

### 3.12. Proposed Highly Potential lncRNA-miRNA-mRNA Network Participates in the Roles of Baicalin on HCC Progression

**Figure 11:**
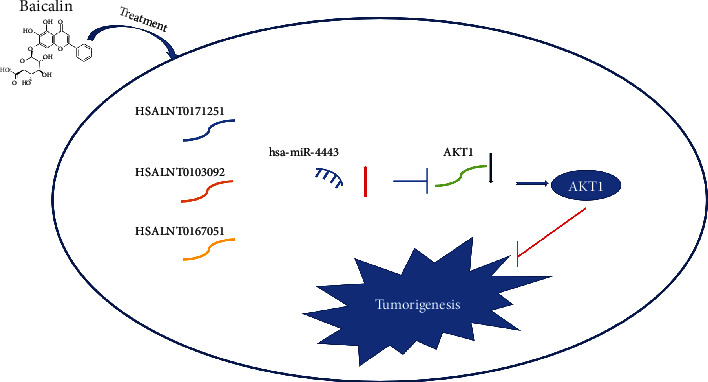
Model of the lncRNA-hsa-miR-4443-AKT1 network and potential roles in HCC progression. HCC: hepatocellular carcinoma.

Based on the above analysis results, we proposed a HSALNT0171251/HSALNT0103092/HSALNT0167051-hsa-miR-4443-AKT1 ceRNA network that can respond to baicalin treatment and hinder HCC tumorigenesis (Figure 11).

## 4. Discussions

Novel ncRNAs are continually to be identified in recent years due to the improvements of high-throughput transcriptome analysis methods [15]. The abnormally expressed lncRNAs were demonstrated that could either stimulate or inhibit cancer progression. Functional pattern of lncRNA-miRNA-mRNA regulatory network has been revealed to participate in the progression of a variety of cancer types [14, 33]. Several studies have been performed to investigate the acting mechanisms of baicalin in cancers [10, 12]. However, the investigation of lncRNA-miRNA-mRNA network that can respond to baicalin treatment remains largely unknown. In this work, our purpose was to construct potential lncRNA-miRNA-mRNA ceRNA triplets in baicalin treatment HCC cells. Using comprehensive bioinformatic analyses methods, several lncRNAs, miRNA, mRNA, and then the lncRNA-miRNA-mRNA triplets that altered after baicalin treatment were identified in HCC.

In this study, we identified 14 overlapping DELs and 26 DEMs via limma package by comparing the microarray data with baicalin or DMSO treatment. By analyzing the miRNA targets of lncRNA and extracting the overlapping miRNAs with DEMs, we screened out 24 miRNAs for following the analyses. After analyzing the expression level and clinical significance of miRNA in HCC, only one miRNA, hsa-miR-4443, was selected for further exploration. hsa-miR-4443 has been revealed with decreased expression in ovarian cancer and stimulated metastasis and tumorigenesis, indicating a tumor-suppressive role [34]. In HCC, hsa-miR-4443 was revealed to be a target of lncRNA FEZF1-AS1 to suppress the aggressive behaviors of cancer cells [35]. In addition, hsa-miR-4443 was also found to be a tumor-suppressive miRNA in other cancers including glioblastoma and osteosarcoma and regulated by lncRNAs [36, 37]. Here, we also showed hsa-miR-4443 decreased expression in HCC and associated with poorer overall survival of cancer patients, suggesting hsa-miR-4443 also function as a tumor-suppressive miRNA in HCC.

Based on the ceRNA hypothesis, lncRNA can serve as decoy for miRNA via miRNA binding site to affect target gene expression. This led us to explore the targets of lncRNA using three prediction tools. A total of 796 overlapped targets were identified and reported to be associated with cancer progression via KEGG enrichment analysis. By identifying hub genes in the PPI network and extracting the overlapping genes with one KEGG pathway that closely associated with tumor progression, we selected four genes for validation. Through analyzing expression and clinical significance of these genes in HCC, we indicated AKT1 and MAPK8 were possible targets. Interestingly, we showed that AKT1 expression level in HCC was opposite with its expression after baicalein treatment, and therefore, it was supposed to be a highly potential target. Hence, we proposed a functional lncRNA-miRNA-mRNA network consisted by three lncRNAs, one miRNA, and one mRNA which was constructed.

It was clear now that levels of TIICs in tumor site influence the response of tumor cell to chemotherapy and immunotherapy. Our results demonstrated that there is a strong positive correlation of AKT1 expression level and infiltration level of CD4+ T cells. Activation of AKT1 was previously reported to be a crucial step for the production of chemoresistant phenotypes, while the inhibition on AKT1 can improve the chemotherapy sensitivity to induce apoptosis [38]. In addition, AKT1 was revealed that it could be upregulated by SET domain containing 5 in breast cancer to stimulate tumor growth and metastasis [39]. Baicalein was found which can regulate cell growth, metastasis, apoptosis, and autophagy in cancer [40, 41]. Hence, the identification of AKT1 as a potential target for baicalein may be used to explain the biological functions of baicalein.

There are several limitations in this work. For example, the analysis of DELs and DEMs was derived from a single dataset obtained from one HCC cell. Therefore, further validation in other HCC cells is necessary. Moreover, the effects of the lncRNA-miRNA-mRNA network we identified in this work should also be validated using in vivo animal experiments.

## 5. Conclusion

In summary, our study performed comprehensive analysis of aberrantly expressed lncRNAs and miRNAs in HCC cell after baicalein treatment. We constructed ceRNA network which contains 3 lncRNAs, 1 miRNA, and 1 target gene that respond to baicalein treatment. Hence, the current study not only helped us to understand the acting mechanisms of baicalein in HCC but also helped us to understand the mechanisms behind HCC progression.

## Figures and Tables

**Table 1:** Information of these differentially expressed lncRNAs identified in this work.

Probe ID	Chromosome	Transcript ID^a^	DMSO vs. 40 *μ*M baicalin		DMSO vs. 80 *μ*M baicalin	
			Log FC	Adj. *P* value	Log FC	Adj. *P* value
TC01004709.hg.1	chr1	HSALNT0014558	-2.8	0.00594	-2.71	0.00895
TC11003010.hg.1	chr11	HSALNT0171251	-1.24	0.00845	-1.35	0.00775
TC08002311.hg.1	chr8	—	-2.04	0.00594	-1.99	0.00614
TC06002662.hg.1	chr6	HSALNT0103092	-1.48	0.01642	-1.5	0.01443
TC22001177.hg.1	chr22	HSALNT0279418	-1.07	0.01808	-1.26	0.02
TC10002246.hg.1	chr10	HSALNT0167051	-1.04	0.01885	-1.13	0.00762
TC01005769.hg.1	chr1	—	-1.04	0.02461	-1.35	0.02953
TC06002661.hg.1	chr6	HSALNT0103091	-1.31	0.02872	-1.24	0.02577
TC06003988.hg.1	chr6	HSALNT0116512	-1.18	0.0303	-1.08	0.02662
TC19002530.hg.1	chr19	—	-1.29	0.0329	-1.24	0.02953
TC18000741.hg.1	chr18	HSALNT0252034	-1.78	0.03355	-1.69	0.02662
TC17002115.hg.1	chr17	HSALNT0238453	-1.61	0.0404	-1.57	0.02406
TC01004837.hg.1	chr1	HSALNT0017759	-1.57	0.04728	-1.52	0.0378
TC01004738.hg.1	chr1	HSALNT0015061	-1.13	0.04796	-1.05	0.0348

^a^Annotation at NONCODE and LncBook.

**Table 2:** Information of these differentially expressed miRNAs identified in this work.

Probe ID	miRNA name	DMSO vs. 40 *μ*M baicalin		DMSO vs. 80 *μ*M baicalin	
		Log FC	Adj. *P* value	Log FC	Adj. *P* value
20515526	hsa-miR-3127-5p	1.37	0.0000801	1.57	0.0034443
20525575	hsa-miR-6807-5p	1.37	0.0002662	2.02	0.0003575
20500789	hsa-miR-186-5p	-1.06	0.0004035	-1.09	0.0016352
20510799	hsa-miR-1972	1.41	0.0004345	2.07	0.0000103
20518460	hsa-miR-3929	1.32	0.0005247	1.34	0.0030186
20517745	hsa-miR-4286	-1.64	0.0005611	-1.75	0.0003857
20505608	hsa-miR-675-5p	-1.04	0.0008396	-1.07	0.0010119
20515637	hsa-miR-3195	1.7	0.0009187	1.54	0.0018014
20518818	hsa-miR-4443	1.34	0.0011303	1.45	0.0004191
20525469	hsa-miR-6754-5p	-1.15	0.0012384	-1.05	0.0038423
20515607	hsa-miR-3178	1.36	0.0013011	1.33	0.0009795
20517916	hsa-miR-3663-3p	1.77	0.0020105	2.03	0.0003883
20506838	hsa-miR-1247-5p	-1.44	0.002577	-1.45	0.0009947
20505609	hsa-miR-675-3p	-1.22	0.0031706	-1.45	0.0020306
20525509	hsa-miR-6774-5p	-1.11	0.0032672	-1.31	0.000251
20506829	hsa-miR-1304-5p	-1.01	0.0036915	-1.14	0.0074363
20518857	hsa-miR-4472	-1.85	0.0041002	-1.71	0.0039872
20500435	hsa-miR-7-5p	-1.53	0.0042541	-2.07	0.008707
20518892	hsa-miR-4497	1.29	0.0068442	1.06	0.0085879
20500485	hsa-miR-222-5p	-1.03	0.00802	-2	0.0001764
20525432	hsa-miR-6735-5p	-1.41	0.0111993	-1.62	0.0044004
20520574	hsa-miR-5194	-1.13	0.0112688	-1.72	0.0033754
20519552	hsa-miR-4720-5p	-1.57	0.0118785	-2.19	0.0016425
20518841	hsa-miR-4461	-1.3	0.0119122	-1.54	0.0002152
20519409	hsa-miR-4634	1.75	0.0120111	1.51	0.0182399
20522011	hsa-miR-5680	-1.57	0. 0.0152221	-3.32	0.0000486

**Table 3:** Overlapping miRNAs between lncRNAs targets predicted by LncBook and DEMs.

Transcript ID	miRNA targets
HSALNT0014558	hsa-miR-4720-5p
HSALNT0171251	hsa-miR-4443, hsa-miR-3127-5p, hsa-miR-6774-5p
HSALNT0103092	hsa-miR-4461, hsa-miR-4443, hsa-miR-1304-5p, hsa-miR-186-5p, hsa-miR-5680, hsa-miR-4720-5p
HSALNT0279418	hsa-miR-4472, hsa-miR-6735-5p, hsa-miR-6754-5p, hsa-miR-3127-5p, hsa-miR-1304-5p, hsa-miR-6807-5p, hsa-miR-5194, hsa-miR-4720-5p
HSALNT0167051	hsa-miR-4461, hsa-miR-4472, hsa-miR-4443, hsa-miR-6735-5p, hsa-miR-6754-5p, hsa-miR-3929, hsa-miR-1972, hsa-miR-222-5p, hsa-miR-6807-5p, hsa-miR-4720-5p
HSALNT0116512	hsa-miR-186-5p
HSALNT0252034	hsa-miR-6735-5p, hsa-miR-3663-3p, hsa-miR-186-5p
HSALNT0238453	hsa-miR-6735-5p, hsa-miR-222-5p, hsa-miR-7-5p
HSALNT0015061	hsa-miR-4461, hsa-miR-6735-5p, hsa-miR-5680

**Table 4:** Validation of miRNA expression with ENCORI.

miRNA name	ENCORI			Significance
	Cancer	Normal	*P* value	
hsa-miR-4720-5p	0.02	0.02	0.91	
hsa-miR-4443	0.34	0.7	2.00*E* − 07	∗∗∗
hsa-miR-4472	0.01	0.01	0.71	
hsa-miR-4461	1.46	1.63	0.034	∗
hsa-miR-3127-5p	3.5	1.63	1.70*E* − 06	∗∗∗
hsa-miR-186-5p	332.27	310.15	0.63	
hsa-miR-6754-5p	0.04	0.02	0.15	
hsa-miR-6774-5p	0.02	0.01	0.82	
hsa-miR-222-5p	3.76	2.76	0.61	
hsa-miR-6735-5p	0.1	0.06	0.17	
hsa-miR-92a-1-5p	4.74	2.46	0.13	
hsa-miR-7-5p	0.92	0.44	0.16	
hsa-miR-3663-3p	0.02	0.01	0.24	
hsa-miR-4286	0.18	0.32	1.80*E* − 06	∗∗∗
hsa-miR-5194	0.01	0.01	0.52	
hsa-miR-5680	0.07	0.11	0.015	∗
hsa-miR-6807-5p	0.07	0.04	0.44	
hsa-miR-1304-5p	0.6	0.45	0.82	
hsa-miR-3929	0.02	0.01	0.2	
hsa-miR-1972	0.01	0.01	0.71	
hsa-miR-3178	0.01	0.02	0.39	
hsa-miR-675-5p	1.27	0.62	0.0031	∗∗∗
hsa-miR-3195	0.02	0.01	0.61	
hsa-miR-1247-5p	8.7	13.43	7.10*E* − 08	∗∗∗

**Table 5:** Correlation of hsa-miR-4443 expression and clinical progression in HCC with different clinicopathological factors.

Clinicopathological parameters	Overall survival (*n* = 372)			
	*N*	HR	95% CI	*P* value
Gender				
Male	252	0.32	0.2-0.49	7.8*E* − 8
Female	119	1.42	0.8-2.5	0.23
Race				
White	182	0.72	0.45-1.15	0.17
Asian	160	0.24	0.13-0.44	4.7*E* − 7
Black/African American	17	—	—	—
Mutation burden				
High	181	0.041	0.25-0.67	0.00027
Low	176	0.55	0.33-0.94	0.028
Stage				
1	172	0.39	0.21-0.73	0.0022
2	85	0.44	0.2-0.97	0.036
3	85	0.45	0.23-0.85	0.012
4	5	—	—	—
Grade				
1	65	2.04	0.79-5.29	0.13
2	176	0.36	0.22-0.61	7.4*E* − 5
3	123	0.54	0.3-0.99	0.044
4	13	—	—	—

HR: hazard ratio.

**Table 6:** Identification of hub genes in PPI network.

Hub gene	Degree	Bottleneck	Eccentricity	Closeness	Betweenness
AKT1	98	289	0.14286	336.59286	162622.2314
MAPK8	42	38	0.14286	279.53571	22548.4148
CREB1	35	12	0.14286	271.16905	20384.65615
AR	34	18	0.14286	269.91905	24113.33091
CD34	32	25	0.14286	270.11905	20585.10932
MDM2	31	18	0.14286	270.09286	22984.0463
SOCS3	25	13	0.125	251.97024	9310.68859
TFRC	25	20	0.14286	259.40952	20815.15833
SREBF1	24	11	0.14286	261.41905	15563.78073
NCOA1	23	2	0.14286	249.02619	6701.76969

**Table 7:** Correlation of AKT1 expression and clinical progression in HCC with different clinicopathological factors.

Clinicopathological parameters	Overall survival (*n* = 371)				Recurrence-free survival (*n* = 316)			
	N	HR	95% CI	*P* value	N	HR	95% CI	*P* value
Gender								
Male	249	1.75	1.11-2.76	0.015	210	1.69	1.13-2.53	0.0094
Female	121	1.66	0.92-3.01	0.0911	106	0.43	0.24-0.8	0.0064
Race								
White	184	1.48	0.94-2.34	0.088	147	1.47	0.93-2.31	0.093
Asian	157	2.19	1.2-3.98	0.0085	145	1.85	1.1-3.1	0.019
Black/African American	17	—	—	—	13	—	—	—
Mutation burden								
High	180	2.22	1.35-3.65	0.0013	155	1.55	0.95-2.51	0.075
Low	177	1.85	1.09-3.15	0.02	150	1.81	1.13-2.91	0.012
Stage								
1	171	2.42	1.32-4.46	0.0034	153	1.77	1.03-3.04	0.038
2	85	2.54	1.09-5.89	0.025	75	0.49	0.25-0.94	0.029
3	85	0.61	0.33-1.14	0.12	70	1.67	0.92-3.05	0.091
4	5	—	—	—	0	—	—	—
Grade								
1	55	2.65	1.05-6.68	0.032	45	0.19	0.07-0.54	0.00046
2	177	1.25	0.74-2.1	0.41	149	1.86	1.14-3.04	0.011
3	121	2.12	1.16-3.88	0.013	107	1.87	1.06-3.3	0.027
4	13	—	—	—	12	—	—	—

HR: hazard ratio.

**Table 8:** Univariate and multivariate analyses of overall survival in patients with HCC.

Variables	Univariate analysis			Multivariate analyses		
	HR	95% CI	*P* value	HR	95% CI	*P* value
AKT1 expression	1.747	1.213-2.516	0.003	1.761	1.222-2.536	0.002
Age	0.790	0.559-1.116	0.181	—	—	—
Gender	1.226	0.860-1.746	0.260	—	—	—
Race	1.063	0.901-1.254	0.470	—	—	—
Tumor stage	1.565	1.119-2.188	0.009	1.565	1.125-2.177	0.008

HR: hazard ratio; CI: confidence interval.

**Table 9:** Drugs acting on AKT1 predicted by DrugBank.

Drug	DrugBank ID	Drug group	Type	PubMed IDs
Arsenic trioxide	DB01169	Approved, investigational	Small molecule	12472888
Inositol 1,3,4,5-tetrakisphosphate	DB01863	Experimental	Small molecule	17139284
Resveratrol	DB02709	Investigational	Small molecule	24968355
N-[2-(5-methyl-4H-1,2,4-triazol-3-yl)phenyl]-7H-pyrrolo[2,3-d]pyrimidin-4-amine	DB07584	Experimental	Small molecule	10592235
ATP	DB00171	Investigational, nutraceutical	Small molecule	17041888
Genistein	DB01645	Investigational	Small molecule	16789737
5-(5-chloro-7H-pyrrolo[2,3-d]pyrimidin-4-yl)-4,5,6,7-tetrahydro-1H-imidazo[4,5-c]pyridine	DB07585	Experimental	Small molecule	10592235
Archexin	DB05971	Investigational	Small molecule	—
Enzastaurin	DB06486	Investigational	Small molecule	—
Perifosine	DB06641	Investigational	Small molecule	18201764

## Data Availability

The data used in this work is available at GEO database or other online tools as described in Materials and Methods.

## Supplementary Materials

Supplementary 1 Supplementary 1 Figure S1: protein-protein interaction network of the 796 target genes for hsa-miR-4443.

Supplementary 2 Supplementary 2 Table S1: miRNA targets for lncRNAs predicted by LncBook.

Supplementary 3 Supplementary 3 Table S2: prediction results for hsa-miR-4443.
