# Supplementary figures and images for: Functional lncRNA-miRNA-mRNA Networks in Response to Baicalein Treatment in Hepatocellular Carcinoma
Source: Biomed Res Int. 2021 Jan 14;2021:8844261. doi: 10.1155/2021/8844261 (PMC7825356; doi:10.1155/2021/8844261)

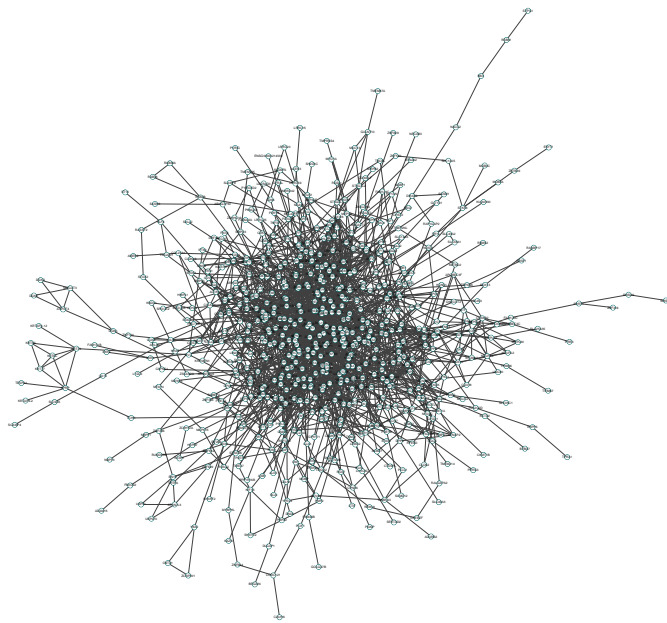

Supplement: Supplementary 1 — Figure S1: protein-protein interaction network of the 796 target genes for hsa-miR-4443. [file 8844261.f1.pdf]
