# Supplementary material for: Functional lncRNA-miRNA-mRNA Networks in Response to Baicalein Treatment in Hepatocellular Carcinoma
Source: Biomed Res Int. 2021 Jan 14;2021:8844261. doi: 10.1155/2021/8844261 (PMC7825356; doi:10.1155/2021/8844261)
Supplement: Supplementary 2 — Table S1: miRNA targets for lncRNAs predicted by LncBook. [file 8844261.f2.docx]

**Table S1** miRNA targets for lncRNAs predicted by LncBOOK

| **Transcript ID** | **miRNA targets** |
| --- | --- |
| HSALNT0014558 | hsa-miR-4655-3p, hsa-miR-4474-3p, hsa-miR-4452, hsa-miR-4436b-5p, hsa-miR-4435, hsa-miR-4421, hsa-miR-5699-3p, hsa-miR-4328, hsa-miR-3940-5p, hsa-miR-4507, hsa-miR-3909, hsa-miR-3714, hsa-miR-3681-5p, hsa-miR-3675-3p, hsa-miR-3606-5p, hsa-miR-3185, hsa-miR-3162-3p, hsa-miR-3153, hsa-miR-6733-5p, hsa-miR-6739-5p, hsa-miR-3140-3p, hsa-miR-3138, hsa-miR-3120-5p, hsa-miR-1976, hsa-miR-1290, hsa-miR-1270, hsa-miR-1238-3p, hsa-miR-1237-3p, hsa-miR-942-5p, hsa-miR-885-3p, hsa-miR-876-5p, hsa-miR-770-5p, hsa-miR-759, hsa-miR-660-5p, hsa-miR-660-3p, hsa-miR-630, hsa-miR-624-5p, hsa-miR-579-3p, hsa-miR-578, hsa-miR-548an, hsa-miR-520f-5p, hsa-miR-516b-5p, hsa-miR-516a-3p, hsa-miR-516b-3p, hsa-miR-7162-5p, hsa-miR-493-5p, hsa-miR-452-3p, hsa-miR-450a-1-3p, hsa-miR-432-5p, hsa-miR-380-5p, hsa-miR-563, hsa-miR-378j, hsa-miR-6839-5p, hsa-miR-335-3p, hsa-miR-205-3p, hsa-miR-204-5p, hsa-miR-211-5p, hsa-miR-188-5p, hsa-miR-145-5p, hsa-miR-145-3p, hsa-miR-142-3p.2, hsa-miR-142-3p.1, hsa-miR-22-5p, hsa-miR-18a-3p, hsa-let-7c-3p, hsa-miR-8056, hsa-miR-7978, hsa-miR-7975, hsa-miR-7154-3p, hsa-miR-6888-3p, hsa-miR-6877-5p, hsa-miR-6874-5p, hsa-miR-6868-3p, hsa-miR-6842-3p, hsa-miR-6841-5p, hsa-miR-6840-3p, hsa-miR-6839-3p, hsa-miR-6838-3p, hsa-miR-6830-3p, hsa-miR-6828-3p, hsa-miR-6809-3p, hsa-miR-6793-5p, hsa-miR-6759-5p, hsa-miR-6748-3p, hsa-miR-6730-5p, hsa-miR-6509-5p, hsa-miR-6073, hsa-miR-5681b, hsa-miR-5681a, hsa-miR-5591-3p, hsa-miR-5004-3p, hsa-miR-4799-5p, hsa-miR-4797-3p, hsa-miR-4782-5p, hsa-miR-5706, hsa-miR-4774-5p, hsa-miR-4724-5p, hsa-miR-4720-5p, hsa-miR-4799-3p, hsa-miR-5588-5p, hsa-miR-4713-3p, hsa-miR-4683, hsa-miR-4681, hsa-miR-4677-5p |
| HSALNT0171251 | hsa-miR-4663, hsa-miR-4659a-3p, hsa-miR-4659b-3p, hsa-miR-4650-5p, hsa-miR-4646-3p, hsa-miR-4649-3p, hsa-miR-4529-5p, hsa-miR-4527, hsa-miR-6503-5p, hsa-miR-4512, hsa-miR-4448, hsa-miR-4443, hsa-miR-7843-5p, hsa-miR-4435, hsa-miR-4434, hsa-miR-4516, hsa-miR-5703, hsa-miR-4426, hsa-miR-4647, hsa-miR-4662b, hsa-miR-4330, hsa-miR-4326, hsa-miR-4325, hsa-miR-4318, hsa-miR-4313, hsa-miR-4297, hsa-miR-5581-5p, hsa-miR-4290, hsa-miR-4282, hsa-miR-4283, hsa-miR-4279, hsa-miR-4271, hsa-miR-4725-3p, hsa-miR-6780b-5p, hsa-miR-4270, hsa-miR-4265, hsa-miR-4296, hsa-miR-4322, hsa-miR-4253, hsa-miR-6862-5p, hsa-miR-3978, hsa-miR-3918, hsa-miR-3682-5p, hsa-miR-3678-3p, hsa-miR-3674, hsa-miR-3667-3p, hsa-miR-3660, hsa-miR-3653-5p, hsa-miR-3622b-5p, hsa-miR-3622a-5p, hsa-miR-3618, hsa-miR-3614-3p, hsa-miR-3612, hsa-miR-3607-5p, hsa-miR-3607-3p, hsa-miR-3202, hsa-miR-3199, hsa-miR-8052, hsa-miR-3192-5p, hsa-miR-3189-3p, hsa-miR-3182, hsa-miR-3179, hsa-miR-6855-5p, hsa-miR-3166, hsa-miR-3158-5p, hsa-miR-3154, hsa-miR-3153, hsa-miR-6733-5p, hsa-miR-6739-5p, hsa-miR-3130-3p, hsa-miR-3128, hsa-miR-3127-5p, hsa-miR-3127-3p, hsa-miR-3121-5p, hsa-miR-3117-3p, hsa-miR-3074-5p, hsa-miR-3065-3p, hsa-miR-2861, hsa-miR-2392, hsa-miR-2355-3p, hsa-miR-2278, hsa-miR-2115-5p, hsa-miR-2114-3p, hsa-miR-1976, hsa-miR-1914-5p, hsa-miR-1910-5p, hsa-miR-1910-3p, hsa-miR-6511a-5p, hsa-miR-1909-3p, hsa-miR-6722-3p, hsa-miR-1827, hsa-miR-1324, hsa-miR-1304-3p, hsa-miR-1302, hsa-miR-4298, hsa-miR-1293, hsa-miR-4483, hsa-miR-1285-5p, hsa-miR-1281, hsa-miR-1273g-3p, hsa-miR-1267, hsa-miR-1266-5p, hsa-miR-4518, hsa-miR-1265, hsa-miR-1264, hsa-miR-1254, hsa-miR-3116, hsa-miR-1251-3p, hsa-miR-1250-3p, hsa-miR-1249-5p, hsa-miR-6797-5p, hsa-miR-1248, hsa-miR-1245b-5p, hsa-miR-1244, hsa-miR-1237-3p, hsa-miR-1236-3p, hsa-miR-1233-3p, hsa-miR-1226-5p, hsa-miR-1208, hsa-miR-4763-3p, hsa-miR-1205, hsa-miR-1200, hsa-miR-1199-5p, hsa-miR-6751-3p, hsa-miR-1183, hsa-miR-1182, hsa-miR-943, hsa-miR-942-5p, hsa-miR-942-3p, hsa-miR-6808-5p, hsa-miR-6893-5p, hsa-miR-939-3p, hsa-miR-936, hsa-miR-934, hsa-miR-890, hsa-miR-877-3p, hsa-miR-876-3p, hsa-miR-875-5p, hsa-miR-767-3p, hsa-miR-766-3p, hsa-miR-759, hsa-miR-758-3p, hsa-miR-711, hsa-miR-676-5p, hsa-miR-671-5p, hsa-miR-664a-5p, hsa-miR-4794, hsa-miR-661, hsa-miR-658, hsa-miR-653-3p, hsa-miR-651-5p, hsa-miR-642b-5p, hsa-miR-639, hsa-miR-637, hsa-miR-6774-5p, hsa-miR-633, hsa-miR-627-3p, hsa-miR-626, hsa-miR-6876-3p, hsa-miR-625-5p, hsa-miR-6506-5p, hsa-miR-612, hsa-miR-6860, hsa-miR-588, hsa-miR-586, hsa-miR-584-3p, hsa-miR-578, hsa-miR-558, hsa-miR-554, hsa-miR-548aa, hsa-miR-548t-3p, hsa-miR-539-5p, hsa-miR-539-3p, hsa-miR-512-3p, hsa-miR-496.1, hsa-miR-483-3p.2, hsa-miR-483-3p.1, hsa-miR-449b-3p, hsa-miR-432-5p, hsa-miR-381-3p, hsa-miR-340-3p, hsa-miR-6827-3p, hsa-miR-335-3p, hsa-miR-324-3p, hsa-miR-1913, hsa-miR-298, hsa-miR-221-5p, hsa-miR-8073, hsa-miR-216a-5p, hsa-miR-212-5p, hsa-miR-211-3p, hsa-miR-204-3p, hsa-miR-4646-5p, hsa-miR-197-3p, hsa-miR-183-5p.1, hsa-miR-4420, hsa-miR-153-5p, hsa-miR-147a, hsa-miR-140-3p.1, hsa-miR-134-5p, hsa-miR-134-3p, hsa-miR-130b-5p, hsa-miR-106b-3p, hsa-miR-103a-2-5p, hsa-miR-100-3p, hsa-miR-32-3p, hsa-miR-590-5p, hsa-miR-16-5p, hsa-miR-195-5p, hsa-miR-424-5p, hsa-miR-497-5p, hsa-miR-10b-3p, hsa-miR-7851-3p, hsa-miR-7847-3p, hsa-miR-7846-3p, hsa-miR-7703, hsa-miR-7162-3p, hsa-miR-7160-3p, hsa-miR-7160-5p, hsa-miR-7152-5p, hsa-miR-7150, hsa-miR-7112-5p, hsa-miR-7110-3p, hsa-miR-7109-5p, hsa-miR-6890-5p, hsa-miR-6889-3p, hsa-miR-6890-3p, hsa-miR-6888-3p, hsa-miR-6886-5p, hsa-miR-6885-3p, hsa-miR-6882-3p, hsa-miR-6881-3p, hsa-miR-6880-5p, hsa-miR-6875-3p, hsa-miR-6874-5p, hsa-miR-6873-3p, hsa-miR-6871-5p, hsa-miR-6868-5p, hsa-miR-6868-3p, hsa-miR-6852-5p, hsa-miR-6847-3p, hsa-miR-6844, hsa-miR-6840-5p, hsa-miR-6838-3p, hsa-miR-6830-5p, hsa-miR-6828-5p, hsa-miR-6828-3p, hsa-miR-6818-3p, hsa-miR-6814-5p, hsa-miR-6808-3p, hsa-miR-6809-3p, hsa-miR-6804-3p, hsa-miR-6802-3p, hsa-miR-6796-5p, hsa-miR-6795-5p, hsa-miR-6887-5p, hsa-miR-6791-3p, hsa-miR-6829-3p, hsa-miR-6783-5p, hsa-miR-6784-3p, hsa-miR-6862-3p, hsa-miR-6780a-3p, hsa-miR-6770-5p, hsa-miR-6765-3p, hsa-miR-6803-5p, hsa-miR-6749-5p, hsa-miR-6750-5p, hsa-miR-6822-5p, hsa-miR-6739-3p, hsa-miR-6735-3p, hsa-miR-6734-5p, hsa-miR-6734-3p, hsa-miR-6721-5p, hsa-miR-6720-5p, hsa-miR-6715b-3p, hsa-miR-6515-5p, hsa-miR-6515-3p, hsa-miR-6508-5p, hsa-miR-8067, hsa-miR-6508-3p, hsa-miR-6502-3p, hsa-miR-6501-3p, hsa-miR-6132, hsa-miR-6836-5p, hsa-miR-6085, hsa-miR-6813-5p, hsa-miR-6079, hsa-miR-5700, hsa-miR-5582-5p, hsa-miR-5193, hsa-miR-5096, hsa-miR-4802-5p, hsa-miR-4802-3p, hsa-miR-4796-5p, hsa-miR-4793-3p, hsa-miR-4778-3p, hsa-miR-4768-5p, hsa-miR-6833-3p, hsa-miR-4761-5p, hsa-miR-4761-3p, hsa-miR-4753-3p, hsa-miR-4755-3p, hsa-miR-4750-5p, hsa-miR-4747-5p, hsa-miR-5196-5p, hsa-miR-4743-3p, hsa-miR-4732-5p, hsa-miR-4727-3p, hsa-miR-4723-5p, hsa-miR-4717-3p, hsa-miR-4704-3p, hsa-miR-4697-3p, hsa-miR-4698, hsa-miR-4691-5p, hsa-miR-4691-3p, hsa-miR-4687-5p, hsa-miR-4686, hsa-miR-4677-3p, hsa-miR-4668-5p |
| HSALNT0103092 | hsa-miR-4652-5p, hsa-miR-4650-5p, hsa-miR-4640-5p, hsa-miR-4633-3p, hsa-miR-6500-5p, hsa-miR-4534, hsa-miR-8082, hsa-miR-4517, hsa-miR-4511, hsa-miR-4509, hsa-miR-4499, hsa-miR-4495, hsa-miR-4494, hsa-miR-4477b, hsa-miR-4476, hsa-miR-6876-5p, hsa-miR-4474-5p, hsa-miR-4463, hsa-miR-4461, hsa-miR-4459, hsa-miR-4457, hsa-miR-4455, hsa-miR-4443, hsa-miR-4428, hsa-miR-4423-3p, hsa-miR-4421, hsa-miR-5699-3p, hsa-miR-4311, hsa-miR-4310, hsa-miR-7157-5p, hsa-miR-4307, hsa-miR-4299, hsa-miR-4284, hsa-miR-4280, hsa-miR-4276, hsa-miR-4269, hsa-miR-6715b-5p, hsa-miR-4263, hsa-miR-4260, hsa-miR-4257, hsa-miR-4251, hsa-miR-3978, hsa-miR-3973, hsa-miR-3942-5p, hsa-miR-3941, hsa-miR-3934-5p, hsa-miR-3928-5p, hsa-miR-6806-3p, hsa-miR-3928-3p, hsa-miR-3927-3p, hsa-miR-6831-5p, hsa-miR-3926, hsa-miR-3925-5p, hsa-miR-3924, hsa-miR-3921, hsa-miR-3915, hsa-miR-3914, hsa-miR-3910, hsa-miR-3714, hsa-miR-3688-5p, hsa-miR-3686, hsa-miR-3680-5p, hsa-miR-3671, hsa-miR-3668, hsa-miR-3665, hsa-miR-3664-3p, hsa-miR-3662, hsa-miR-3653-3p, hsa-miR-3646, hsa-miR-3617-3p, hsa-miR-3613-5p, hsa-miR-3613-3p, hsa-miR-3612, hsa-miR-3591-5p, hsa-miR-3607-3p, hsa-miR-3529-3p, hsa-miR-3201, hsa-miR-4791, hsa-miR-3199, hsa-miR-8052, hsa-miR-3194-5p, hsa-miR-3192-5p, hsa-miR-3188, hsa-miR-3185, hsa-miR-3183, hsa-miR-4723-3p, hsa-miR-6769b-3p, hsa-miR-3176, hsa-miR-3922-3p, hsa-miR-3174, hsa-miR-3173-3p, hsa-miR-6891-5p, hsa-miR-3169, hsa-miR-3165, hsa-miR-3163, hsa-miR-3162-5p, hsa-miR-3160-3p, hsa-miR-3159, hsa-miR-3156-5p, hsa-miR-3154, hsa-miR-3153, hsa-miR-3152-5p, hsa-miR-3148, hsa-miR-3133, hsa-miR-3125, hsa-miR-3916, hsa-miR-6859-5p, hsa-miR-3123, hsa-miR-3121-3p, hsa-miR-3120-3p, hsa-miR-3065-5p, hsa-miR-2681-3p, hsa-miR-2467-3p, hsa-miR-2355-5p, hsa-miR-2355-3p, hsa-miR-2276-3p, hsa-miR-1915-5p, hsa-miR-1910-5p, hsa-miR-1909-3p, hsa-miR-6722-3p, hsa-miR-1304-5p, hsa-miR-1299, hsa-miR-1294, hsa-miR-1289, hsa-miR-1290, hsa-miR-1288-5p, hsa-miR-1283, hsa-miR-1273f, hsa-miR-1266-5p, hsa-miR-1256, hsa-miR-1253, hsa-miR-1254, hsa-miR-1252-5p, hsa-miR-1252-3p, hsa-miR-1246, hsa-miR-1245b-5p, hsa-miR-1244, hsa-miR-1245a, hsa-miR-8079, hsa-miR-1233-5p, hsa-miR-6778-5p, hsa-miR-1228-3p, hsa-miR-1208, hsa-miR-1207-5p, hsa-miR-4763-3p, hsa-miR-1193, hsa-miR-1178-5p, hsa-miR-944, hsa-miR-942-5p, hsa-miR-940, hsa-miR-6808-5p, hsa-miR-6893-5p, hsa-miR-939-3p, hsa-miR-937-5p, hsa-miR-936, hsa-miR-934, hsa-miR-921, hsa-miR-891b, hsa-miR-891a-3p, hsa-miR-890, hsa-miR-876-5p, hsa-miR-876-3p, hsa-miR-875-5p, hsa-miR-766-5p, hsa-miR-766-3p, hsa-miR-765, hsa-miR-708-3p, hsa-miR-671-5p, hsa-miR-665, hsa-miR-661, hsa-miR-655-5p, hsa-miR-653-3p, hsa-miR-652-5p, hsa-miR-651-5p, hsa-miR-651-3p, hsa-miR-645, hsa-miR-642a-5p, hsa-miR-3617-5p, hsa-miR-624-3p, hsa-miR-619-5p, hsa-miR-6506-5p, hsa-miR-612, hsa-miR-1285-3p, hsa-miR-3187-5p, hsa-miR-5189-5p, hsa-miR-6860, hsa-miR-605-3p, hsa-miR-603, hsa-miR-599, hsa-miR-597-5p, hsa-miR-595, hsa-miR-583, hsa-miR-580-3p, hsa-miR-577, hsa-miR-570-3p, hsa-miR-568, hsa-miR-561-5p, hsa-miR-556-5p, hsa-miR-551b-5p, hsa-miR-548v, hsa-miR-548u, hsa-miR-7161-5p, hsa-miR-548n, hsa-miR-548m, hsa-miR-548l, hsa-miR-548b-3p, hsa-miR-548c-3p, hsa-miR-548az-5p, hsa-miR-548t-5p, hsa-miR-548aw, hsa-miR-548ab, hsa-miR-548b-5p, hsa-miR-548bb-5p, hsa-miR-548av-5p, hsa-miR-548k, hsa-miR-8054, hsa-miR-548ae-3p, hsa-miR-548ah-3p, hsa-miR-548aj-3p, hsa-miR-548am-3p, hsa-miR-548aq-3p, hsa-miR-548j-3p, hsa-miR-548x-3p, hsa-miR-548ao-5p, hsa-miR-548ax, hsa-miR-3609, hsa-miR-545-5p, hsa-miR-544a, hsa-miR-543, hsa-miR-541-5p, hsa-miR-539-5p, hsa-miR-524-5p, hsa-miR-520g-5p, hsa-miR-516b-5p, hsa-miR-515-5p, hsa-miR-519e-5p, hsa-miR-514b-5p, hsa-miR-513b-3p, hsa-miR-510-5p, hsa-miR-509-3p, hsa-miR-508-5p, hsa-miR-505-5p, hsa-miR-505-3p.2, hsa-miR-505-3p.1, hsa-miR-495-3p, hsa-miR-490-5p, hsa-miR-486-3p, hsa-miR-485-5p, hsa-miR-452-5p, hsa-miR-451b, hsa-miR-450b-5p, hsa-miR-449b-3p, hsa-miR-421, hsa-miR-409-5p, hsa-miR-383-5p.2, hsa-miR-382-5p, hsa-miR-382-3p, hsa-miR-380-3p, hsa-miR-376b-5p, hsa-miR-376c-5p, hsa-miR-655-3p, hsa-miR-371b-5p, hsa-miR-373-5p, hsa-miR-616-5p, hsa-miR-372-5p, hsa-miR-363-5p, hsa-miR-6745, hsa-miR-342-3p, hsa-miR-340-5p, hsa-miR-335-3p, hsa-miR-330-3p, hsa-miR-324-5p, hsa-miR-302b-5p, hsa-miR-302d-5p, hsa-miR-372-3p, hsa-miR-223-5p, hsa-miR-223-3p, hsa-miR-219a-2-3p, hsa-miR-216b-3p, hsa-miR-214-3p, hsa-miR-3619-5p, hsa-miR-204-3p, hsa-miR-4646-5p, hsa-miR-196a-3p, hsa-miR-192-3p, hsa-miR-190a-3p, hsa-miR-186-5p, hsa-miR-182-5p, hsa-miR-150-3p, hsa-miR-146a-3p, hsa-miR-142-5p, hsa-miR-142-3p.2, hsa-miR-142-3p.1, hsa-miR-141-5p, hsa-miR-140-3p.1, hsa-miR-139-5p, hsa-miR-129-5p, hsa-miR-127-5p, hsa-miR-126-5p, hsa-miR-124-3p.2, hsa-miR-506-3p, hsa-miR-92a-2-5p, hsa-miR-34c-3p, hsa-miR-449c-5p, hsa-miR-32-3p, hsa-miR-6788-5p, hsa-miR-6780a-5p, hsa-miR-28-5p, hsa-miR-708-5p, hsa-miR-25-3p, hsa-miR-32-5p, hsa-miR-363-3p, hsa-miR-367-3p, hsa-miR-21-5p, hsa-miR-590-5p, hsa-miR-17-5p, hsa-miR-93-5p, hsa-miR-15b-3p, hsa-miR-16-5p, hsa-miR-195-5p, hsa-miR-424-5p, hsa-miR-497-5p, hsa-miR-98-5p, hsa-miR-8485, hsa-miR-8087, hsa-miR-8083, hsa-miR-8065, hsa-miR-8060, hsa-miR-7974, hsa-miR-7856-5p, hsa-miR-7855-5p, hsa-miR-7845-5p, hsa-miR-7844-5p, hsa-miR-7843-3p, hsa-miR-7703, hsa-miR-7161-3p, hsa-miR-7156-3p, hsa-miR-7154-5p, hsa-miR-7151-5p, hsa-miR-7150, hsa-miR-6892-3p, hsa-miR-6888-5p, hsa-miR-6888-3p, hsa-miR-6884-3p, hsa-miR-6878-5p, hsa-miR-6873-5p, hsa-miR-6868-5p, hsa-miR-6858-3p, hsa-miR-6857-3p, hsa-miR-6854-5p, hsa-miR-6849-3p, hsa-miR-6847-5p, hsa-miR-6841-5p, hsa-miR-6840-3p, hsa-miR-6839-3p, hsa-miR-6838-3p, hsa-miR-6830-5p, hsa-miR-6828-5p, hsa-miR-6809-3p, hsa-miR-6804-5p, hsa-miR-6801-5p, hsa-miR-6798-5p, hsa-miR-6783-5p, hsa-miR-6779-3p, hsa-miR-6770-5p, hsa-miR-6769a-5p, hsa-miR-6769b-5p, hsa-miR-6768-5p, hsa-miR-6761-5p, hsa-miR-6759-5p, hsa-miR-6759-3p, hsa-miR-6758-5p, hsa-miR-6856-5p, hsa-miR-6766-5p, hsa-miR-6750-3p, hsa-miR-6748-5p, hsa-miR-6734-5p, hsa-miR-6730-5p, hsa-miR-6721-5p, hsa-miR-6720-5p, hsa-miR-6715a-3p, hsa-miR-6515-3p, hsa-miR-6509-5p, hsa-miR-8067, hsa-miR-6505-5p, hsa-miR-6504-3p, hsa-miR-6501-3p, hsa-miR-6134, hsa-miR-6128, hsa-miR-6124, hsa-miR-6089, hsa-miR-6083, hsa-miR-6079, hsa-miR-6073, hsa-miR-6072, hsa-miR-6891-3p, hsa-miR-5702, hsa-miR-5699-5p, hsa-miR-5697, hsa-miR-5695, hsa-miR-5693, hsa-miR-5694, hsa-miR-5692a, hsa-miR-5691, hsa-miR-5687, hsa-miR-5680, hsa-miR-5681a, hsa-miR-5582-5p, hsa-miR-5582-3p, hsa-miR-5572, hsa-miR-5197-3p, hsa-miR-5191, hsa-miR-5094, hsa-miR-5089-3p, hsa-miR-5087, hsa-miR-5007-5p, hsa-miR-5002-5p, hsa-miR-5000-5p, hsa-miR-4999-3p, hsa-miR-4802-5p, hsa-miR-4799-5p, hsa-miR-4797-3p, hsa-miR-4796-3p, hsa-miR-4786-3p, hsa-miR-4779, hsa-miR-4778-5p, hsa-miR-4777-3p, hsa-miR-4774-5p, hsa-miR-4768-3p, hsa-miR-4768-5p, hsa-miR-6833-3p, hsa-miR-4766-5p, hsa-miR-4763-5p, hsa-miR-4760-3p, hsa-miR-4760-5p, hsa-miR-8061, hsa-miR-4743-3p, hsa-miR-4740-5p, hsa-miR-4736, hsa-miR-4728-3p, hsa-miR-4722-5p, hsa-miR-4720-5p, hsa-miR-4799-3p, hsa-miR-5588-5p, hsa-miR-4712-3p, hsa-miR-4710, hsa-miR-4709-3p, hsa-miR-4709-5p, hsa-miR-4704-5p, hsa-miR-4699-3p, hsa-miR-4699-5p, hsa-miR-4697-3p, hsa-miR-4698, hsa-miR-4695-5p, hsa-miR-4696, hsa-miR-5685, hsa-miR-4689, hsa-miR-6858-5p, hsa-miR-4684-3p, hsa-miR-4683, hsa-miR-4668-5p |
| HSALNT0279418 | hsa-miR-8089, hsa-miR-4665-3p, hsa-miR-4663, hsa-miR-4656, hsa-miR-4655-5p, hsa-miR-4655-3p, hsa-miR-4654, hsa-miR-4769-5p, hsa-miR-4652-3p, hsa-miR-4650-5p, hsa-miR-4649-3p, hsa-miR-4648, hsa-miR-4645-5p, hsa-miR-4673, hsa-miR-4640-5p, hsa-miR-4640-3p, hsa-miR-4639-3p, hsa-miR-4638-5p, hsa-miR-4539, hsa-miR-4529-5p, hsa-miR-4524b-3p, hsa-miR-4522, hsa-miR-4514, hsa-miR-4692, hsa-miR-4512, hsa-miR-4505, hsa-miR-5787, hsa-miR-4493, hsa-miR-4487, hsa-miR-4482-3p, hsa-miR-4745-5p, hsa-miR-4476, hsa-miR-6876-5p, hsa-miR-4469, hsa-miR-4467, hsa-miR-4462, hsa-miR-4459, hsa-miR-4453, hsa-miR-4538, hsa-miR-4450, hsa-miR-4447, hsa-miR-4472, hsa-miR-4448, hsa-miR-4446-3p, hsa-miR-4437, hsa-miR-4436b-3p, hsa-miR-4632-5p, hsa-miR-6735-5p, hsa-miR-6879-5p, hsa-miR-7843-5p, hsa-miR-4435, hsa-miR-4434, hsa-miR-4516, hsa-miR-5703, hsa-miR-4433b-5p, hsa-miR-4425, hsa-miR-6127, hsa-miR-6133, hsa-miR-4320, hsa-miR-4314, hsa-miR-4313, hsa-miR-4312, hsa-miR-7157-5p, hsa-miR-4303, hsa-miR-4297, hsa-miR-5581-5p, hsa-miR-4294, hsa-miR-4292, hsa-miR-6791-5p, hsa-miR-4291, hsa-miR-4284  hsa-miR-4278, hsa-miR-4274, hsa-miR-4725-3p, hsa-miR-6780b-5p, hsa-miR-4270, hsa-miR-4441, hsa-miR-6754-5p, hsa-miR-4269, hsa-miR-6715b-5p, hsa-miR-4268, hsa-miR-4267, hsa-miR-4265, hsa-miR-4296, hsa-miR-4322, hsa-miR-4259, hsa-miR-4260, hsa-miR-4254, hsa-miR-4253, hsa-miR-6862-5p, hsa-miR-4251, hsa-miR-3975, hsa-miR-3960, hsa-miR-3944-5p, hsa-miR-3943, hsa-miR-3937, hsa-miR-3934-5p, hsa-miR-3934-3p, hsa-miR-3927-3p, hsa-miR-3921, hsa-miR-4653-5p, hsa-miR-3918, hsa-miR-3915, hsa-miR-3692-5p, hsa-miR-3690, hsa-miR-3689d, hsa-miR-6851-5p, hsa-miR-3679-3p, hsa-miR-3677-3p, hsa-miR-3677-5p, hsa-miR-3675-5p, hsa-miR-3670, hsa-miR-3667-3p, hsa-miR-3665, hsa-miR-3664-5p, hsa-miR-3663-5p, hsa-miR-3657, hsa-miR-3653-5p, hsa-miR-3652, hsa-miR-4430, hsa-miR-3622b-5p, hsa-miR-3622a-5p, hsa-miR-3622a-3p, hsa-miR-3622b-3p, hsa-miR-3620-3p, hsa-miR-3616-3p, hsa-miR-3614-5p, hsa-miR-3611, hsa-miR-3605-5p, hsa-miR-3202, hsa-miR-3194-3p, hsa-miR-3192-5p, hsa-miR-3190-5p, hsa-miR-4723-3p, hsa-miR-3180-3p, hsa-miR-3175, hsa-miR-3176, hsa-miR-3922-3p, hsa-miR-3173-5p, hsa-miR-3170, hsa-miR-6855-5p, hsa-miR-3166, hsa-miR-3160-3p, hsa-miR-3159, hsa-miR-3157-5p, hsa-miR-3151-5p, hsa-miR-3150b-3p, hsa-miR-3150a-3p, hsa-miR-3147, hsa-miR-3141, hsa-miR-3135b, hsa-miR-3135a, hsa-miR-3130-5p, hsa-miR-4482-5p, hsa-miR-3127-5p, hsa-miR-3127-3p, hsa-miR-6756-3p, hsa-miR-3125, hsa-miR-3916, hsa-miR-6859-5p, hsa-miR-3124-3p, hsa-miR-3121-5p, hsa-miR-3064-3p, hsa-miR-2909, hsa-miR-2861, hsa-miR-2682-3p, hsa-miR-6781-3p, hsa-miR-2681-3p, hsa-miR-2467-5p, hsa-miR-2392, hsa-miR-2278, hsa-miR-2276-5p, hsa-miR-2115-5p, hsa-miR-2110, hsa-miR-1976, hsa-miR-1915-5p, hsa-miR-1915-3p, hsa-miR-6764-5p, hsa-miR-1914-5p, hsa-miR-1910-3p, hsa-miR-6511a-5p, hsa-miR-6722-3p, hsa-miR-1827, hsa-miR-1825, hsa-miR-1587, hsa-miR-3620-5p, hsa-miR-1537-5p, hsa-miR-1471, hsa-miR-1470, hsa-miR-1343-3p, hsa-miR-1324, hsa-miR-1306-5p, hsa-miR-1304-5p, hsa-miR-1304-3p, hsa-miR-4298, hsa-miR-1303, hsa-miR-1301-3p, hsa-miR-1296-5p, hsa-miR-1298-3p, hsa-miR-1296-3p, hsa-miR-1292-5p, hsa-miR-1293, hsa-miR-1291, hsa-miR-6775-3p, hsa-miR-1286, hsa-miR-1285-5p, hsa-miR-1282, hsa-miR-1281, hsa-miR-1273h-3p, hsa-miR-1275, hsa-miR-4665-5p, hsa-miR-1273a, hsa-miR-1270, hsa-miR-1266-5p, hsa-miR-4518, hsa-miR-1262, hsa-miR-1258, hsa-miR-1254, hsa-miR-3116, hsa-miR-1252-5p, hsa-miR-1249-5p, hsa-miR-6797-5p, hsa-miR-1245a, hsa-miR-8079, hsa-miR-4758-5p, hsa-miR-1237-3p, hsa-miR-1233-5p, hsa-miR-6778-5p, hsa-miR-1231, hsa-miR-1229-5p, hsa-miR-1227-5p, hsa-miR-1227-3p, hsa-miR-1226-5p, hsa-miR-1207-5p, hsa-miR-4763-3p, hsa-miR-1207-3p, hsa-miR-1205, hsa-miR-1204, hsa-miR-1200, hsa-miR-1197, hsa-miR-1193, hsa-miR-1184, hsa-miR-1178-3p, hsa-miR-940, hsa-miR-6808-5p, hsa-miR-6893-5p, hsa-miR-939-5p, hsa-miR-1343-5p, hsa-miR-939-3p, hsa-miR-935, hsa-miR-924, hsa-miR-920, hsa-miR-4300, hsa-miR-5591-5p, hsa-miR-6726-5p, hsa-miR-891b, hsa-miR-891a-3p, hsa-miR-885-3p, hsa-miR-877-5p, hsa-miR-876-5p, hsa-miR-874-5p, hsa-miR-769-5p, hsa-miR-765, hsa-miR-764, hsa-miR-760, hsa-miR-762, hsa-miR-4498, hsa-miR-5001-5p, hsa-miR-759, hsa-miR-744-3p, hsa-miR-670-5p, hsa-miR-661, hsa-miR-660-3p, hsa-miR-654-5p, hsa-miR-653-3p, hsa-miR-652-3p, hsa-miR-646, hsa-miR-642a-5p, hsa-miR-638, hsa-miR-637, hsa-miR-636, hsa-miR-632, hsa-miR-629-3p, hsa-miR-628-5p, hsa-miR-625-5p, hsa-miR-619-5p, hsa-miR-6506-5p, hsa-miR-617, hsa-miR-615-3p, hsa-miR-612, hsa-miR-3187-5p, hsa-miR-608, hsa-miR-4651, hsa-miR-602, hsa-miR-593-5p, hsa-miR-592, hsa-miR-588, hsa-miR-582-5p, hsa-miR-578, hsa-miR-574-5p, hsa-miR-571, hsa-miR-566, hsa-miR-564, hsa-miR-552-3p, hsa-miR-550b-2-5p, hsa-miR-550a-3-5p, hsa-miR-550a-5p, hsa-miR-1271-3p, hsa-miR-526b-5p, hsa-miR-522-3p, hsa-miR-520g-3p, hsa-miR-518d-5p, hsa-miR-517-5p, hsa-miR-516b-5p, hsa-miR-513b-3p, hsa-miR-513a-5p, hsa-miR-512-5p, hsa-miR-510-5p, hsa-miR-510-3p, hsa-miR-506-5p, hsa-miR-504-3p, hsa-miR-503-5p, hsa-miR-502-3p, hsa-miR-500b-3p, hsa-miR-500a-5p, hsa-miR-500a-3p, hsa-miR-493-3p, hsa-miR-486-3p, hsa-miR-485-5p, hsa-miR-484, hsa-miR-483-5p, hsa-miR-483-3p.2, hsa-miR-483-3p.1, hsa-miR-455-5p, hsa-miR-450b-3p, hsa-miR-769-3p, hsa-miR-432-5p, hsa-miR-431-5p, hsa-miR-423-5p, hsa-miR-421, hsa-miR-412-3p, hsa-miR-6754-3p, hsa-miR-411-5p.2, hsa-miR-409-5p, hsa-miR-383-5p.2, hsa-miR-383-5p.1, hsa-miR-383-3p, hsa-miR-382-5p, hsa-miR-378g, hsa-miR-378a-5p, hsa-miR-377-5p, hsa-miR-371b-3p, hsa-miR-370-3p, hsa-miR-361-3p, hsa-miR-345-5p, hsa-miR-345-3p, hsa-miR-342-5p, hsa-miR-4664-5p, hsa-miR-331-3p, hsa-miR-329-5p, hsa-miR-328-3p, hsa-miR-326  hsa-miR-324-3p, hsa-miR-1913, hsa-miR-323a-5p, hsa-miR-320e, hsa-miR-302c-3p.2, hsa-miR-372-3p, hsa-miR-373-3p, hsa-miR-301a-5p, hsa-miR-296-5p, hsa-miR-296-3p, hsa-miR-223-3p, hsa-miR-221-5p, hsa-miR-8073, hsa-miR-216a-5p, hsa-miR-215-3p, hsa-miR-214-3p, hsa-miR-3619-5p, hsa-miR-212-5p, hsa-miR-210-5p, hsa-miR-205-5p, hsa-miR-204-3p, hsa-miR-4646-5p, hsa-miR-203b-5p, hsa-miR-6718-5p, hsa-miR-194-3p, hsa-miR-191-5p, hsa-miR-188-3p, hsa-miR-185-5p, hsa-miR-183-5p.2, hsa-miR-182-5p, hsa-miR-181d-3p, hsa-miR-150-5p, hsa-miR-149-3p, hsa-miR-4728-5p, hsa-miR-6785-5p, hsa-miR-6883-5p, hsa-miR-148b-5p, hsa-miR-6874-3p, hsa-miR-152-3p, hsa-miR-147b, hsa-miR-143-5p, hsa-miR-143-3p, hsa-miR-140-3p.2, hsa-miR-140-3p.1, hsa-miR-139-5p, hsa-miR-139-3p, hsa-miR-138-5p, hsa-miR-134-3p, hsa-miR-454-3p, hsa-miR-128-1-5p, hsa-miR-126-5p, hsa-miR-125a-3p, hsa-miR-122-5p, hsa-miR-106a-3p, hsa-miR-103a-2-5p, hsa-miR-100-3p, hsa-miR-96-5p, hsa-miR-1271-5p, hsa-miR-93-3p, hsa-miR-92a-2-5p, hsa-miR-449c-5p, hsa-miR-33b-3p, hsa-miR-31-5p, hsa-miR-6788-5p, hsa-miR-30b-3p, hsa-miR-1273h-5p, hsa-miR-6779-5p, hsa-miR-6780a-5p, hsa-miR-29a-5p, hsa-miR-28-5p, hsa-miR-708-5p, hsa-miR-27a-5p, hsa-miR-18a-3p, hsa-miR-17-5p, hsa-miR-93-5p, hsa-miR-1-3p, hsa-miR-206, hsa-miR-98-5p, hsa-miR-9500, hsa-miR-8080, hsa-miR-8077, hsa-miR-8065, hsa-miR-8062, hsa-miR-7977, hsa-miR-7976, hsa-miR-7975, hsa-miR-7974, hsa-miR-7973, hsa-miR-7855-5p, hsa-miR-7854-3p, hsa-miR-7851-3p, hsa-miR-7847-3p, hsa-miR-7846-3p, hsa-miR-7704, hsa-miR-7703, hsa-miR-7160-3p, hsa-miR-7160-5p, hsa-miR-7159-5p, hsa-miR-7159-3p, hsa-miR-7158-5p, hsa-miR-7156-3p, hsa-miR-7155-5p, hsa-miR-7154-3p, hsa-miR-7152-5p, hsa-miR-7113-5p, hsa-miR-7113-3p, hsa-miR-7109-5p, hsa-miR-7106-5p, hsa-miR-6894-5p, hsa-miR-6894-3p, hsa-miR-6892-3p, hsa-miR-6890-5p, hsa-miR-6889-3p, hsa-miR-6888-5p, hsa-miR-6887-3p, hsa-miR-6884-3p, hsa-miR-6878-3p, hsa-miR-6873-3p, hsa-miR-6873-5p, hsa-miR-6872-3p, hsa-miR-6869-5p, hsa-miR-6868-3p, hsa-miR-6867-3p, hsa-miR-6861-5p, hsa-miR-6859-3p, hsa-miR-6857-5p, hsa-miR-6854-5p, hsa-miR-6853-5p, hsa-miR-6852-5p, hsa-miR-6849-3p, hsa-miR-6847-5p, hsa-miR-6845-3p, hsa-miR-6842-3p, hsa-miR-6837-3p, hsa-miR-6835-5p, hsa-miR-6834-5p, hsa-miR-6832-5p, hsa-miR-6827-5p, hsa-miR-6826-3p, hsa-miR-6825-5p, hsa-miR-6820-5p, hsa-miR-6819-3p, hsa-miR-6877-3p, hsa-miR-6815-5p, hsa-miR-6810-5p, hsa-miR-6807-5p, hsa-miR-6804-5p, hsa-miR-6804-3p, hsa-miR-6801-5p, hsa-miR-6801-3p, hsa-miR-6799-5p, hsa-miR-6798-5p, hsa-miR-6796-3p, hsa-miR-6796-5p, hsa-miR-6795-3p, hsa-miR-6794-3p, hsa-miR-6791-3p, hsa-miR-6829-3p, hsa-miR-6790-3p, hsa-miR-6789-5p, hsa-miR-6787-3p, hsa-miR-6785-3p, hsa-miR-6784-5p, hsa-miR-6782-5p, hsa-miR-6782-3p, hsa-miR-6780b-3p, hsa-miR-6778-3p, hsa-miR-6777-5p, hsa-miR-6889-5p, hsa-miR-6856-5p, hsa-miR-6757-5p, hsa-miR-6756-5p, hsa-miR-6766-5p, hsa-miR-6755-5p, hsa-miR-6752-5p, hsa-miR-6842-5p, hsa-miR-7110-5p, hsa-miR-6752-3p, hsa-miR-6751-5p, hsa-miR-6803-5p, hsa-miR-6749-5p, hsa-miR-6750-5p, hsa-miR-6822-5p, hsa-miR-6748-5p, hsa-miR-6747-3p, hsa-miR-6741-5p, hsa-miR-6742-5p, hsa-miR-6742-3p, hsa-miR-6737-5p, hsa-miR-6812-5p, hsa-miR-6819-5p, hsa-miR-6735-3p, hsa-miR-6736-3p, hsa-miR-6734-5p, hsa-miR-6734-3p, hsa-miR-6732-5p, hsa-miR-6731-5p, hsa-miR-8085, hsa-miR-6730-3p, hsa-miR-6728-5p, hsa-miR-6728-3p, hsa-miR-6724-5p, hsa-miR-6773-5p, hsa-miR-6721-5p, hsa-miR-6720-3p, hsa-miR-6716-5p, hsa-miR-6515-5p, hsa-miR-6514-3p, hsa-miR-6508-3p, hsa-miR-6504-3p, hsa-miR-6501-3p, hsa-miR-6165, hsa-miR-6134, hsa-miR-6132, hsa-miR-6836-5p, hsa-miR-6131, hsa-miR-6090, hsa-miR-6089, hsa-miR-6081, hsa-miR-6077, hsa-miR-6075, hsa-miR-6072, hsa-miR-6891-3p, hsa-miR-6071, hsa-miR-5739, hsa-miR-5694, hsa-miR-5691, hsa-miR-5589-3p, hsa-miR-5587-5p, hsa-miR-5572, hsa-miR-5571-5p, hsa-miR-5197-5p, hsa-miR-5194, hsa-miR-5192, hsa-miR-5191, hsa-miR-5187-5p, hsa-miR-5186, hsa-miR-5096, hsa-miR-5095, hsa-miR-5090, hsa-miR-6775-5p, hsa-miR-5089-5p, hsa-miR-5088-5p, hsa-miR-5011-3p, hsa-miR-5010-5p, hsa-miR-5009-5p, hsa-miR-8058, hsa-miR-5008-3p, hsa-miR-5008-5p, hsa-miR-5004-5p, hsa-miR-5002-3p, hsa-miR-4999-5p, hsa-miR-4802-5p, hsa-miR-4797-5p, hsa-miR-4795-3p, hsa-miR-4795-5p, hsa-miR-4782-5p, hsa-miR-5706, hsa-miR-4781-3p, hsa-miR-4779, hsa-miR-4775, hsa-miR-4772-3p, hsa-miR-4769-3p, hsa-miR-4768-3p, hsa-miR-4767, hsa-miR-4763-5p, hsa-miR-4761-3p, hsa-miR-4758-3p, hsa-miR-4757-5p, hsa-miR-6744-3p, hsa-miR-4755-3p, hsa-miR-4749-3p, hsa-miR-4747-5p, hsa-miR-5196-5p, hsa-miR-4750-3p, hsa-miR-4743-5p, hsa-miR-4742-5p, hsa-miR-4742-3p, hsa-miR-4740-3p, hsa-miR-4739, hsa-miR-4738-5p, hsa-miR-4736, hsa-miR-4735-5p, hsa-miR-4732-3p, hsa-miR-4731-5p, hsa-miR-4726-3p, hsa-miR-4725-5p, hsa-miR-4724-3p, hsa-miR-4722-5p, hsa-miR-5698, hsa-miR-7111-5p, hsa-miR-4721, hsa-miR-4722-3p, hsa-miR-6727-3p, hsa-miR-4720-5p, hsa-miR-4799-3p, hsa-miR-5588-5p, hsa-miR-4717-3p, hsa-miR-4716-5p, hsa-miR-4716-3p, hsa-miR-6794-5p, hsa-miR-4715-3p, hsa-miR-4714-5p, hsa-miR-4713-3p, hsa-miR-4713-5p, hsa-miR-4704-3p, hsa-miR-4695-5p, hsa-miR-4693-3p, hsa-miR-4691-5p, hsa-miR-6792-3p, hsa-miR-4691-3p, hsa-miR-4690-5p, hsa-miR-4689, hsa-miR-6858-5p, hsa-miR-4688, hsa-miR-6743-5p, hsa-miR-4685-5p, hsa-miR-6837-5p, hsa-miR-4683, hsa-miR-4682, hsa-miR-4680-5p, hsa-miR-4677-3p, hsa-miR-4675, hsa-miR-4741, hsa-miR-4674, hsa-miR-4667-3p, hsa-miR-4700-5p |
| HSALNT0167051 | hsa-miR-8089, hsa-miR-4666a-3p, hsa-miR-4663, hsa-miR-4656, hsa-miR-4652-5p, hsa-miR-4650-5p, hsa-miR-4649-5p, hsa-miR-4536-5p, hsa-miR-4533, hsa-miR-4531, hsa-miR-4524a-3p, hsa-miR-4517, hsa-miR-4515, hsa-miR-4513, hsa-miR-4512, hsa-miR-4506, hsa-miR-4505, hsa-miR-5787, hsa-miR-4499, hsa-miR-4496, hsa-miR-4494, hsa-miR-4487, hsa-miR-4486, hsa-miR-4482-3p, hsa-miR-4476, hsa-miR-6876-5p, hsa-miR-4475, hsa-miR-4474-3p, hsa-miR-4471, hsa-miR-8059, hsa-miR-4461, hsa-miR-4459, hsa-miR-4454, hsa-miR-4450, hsa-miR-4447, hsa-miR-4472, hsa-miR-4446-3p, hsa-miR-4443, hsa-miR-4436b-5p, hsa-miR-4436b-3p, hsa-miR-4632-5p, hsa-miR-6735-5p, hsa-miR-6879-5p, hsa-miR-7843-5p, hsa-miR-4435, hsa-miR-4433b-3p, hsa-miR-4433a-3p, hsa-miR-4426, hsa-miR-4647, hsa-miR-4662b, hsa-miR-4421, hsa-miR-5699-3p, hsa-miR-4419a, hsa-miR-4510, hsa-miR-6127, hsa-miR-6129, hsa-miR-6130, hsa-miR-6133, hsa-miR-4320, hsa-miR-4318, hsa-miR-4311, hsa-miR-4307, hsa-miR-4303, hsa-miR-4293, hsa-miR-4291, hsa-miR-4284, hsa-miR-4282, hsa-miR-4279, hsa-miR-4271, hsa-miR-4725-3p, hsa-miR-6780b-5p, hsa-miR-4270, hsa-miR-4441, hsa-miR-6754-5p, hsa-miR-4266, hsa-miR-4261, hsa-miR-4260, hsa-miR-4255, hsa-miR-4253, hsa-miR-6862-5p, hsa-miR-4251, hsa-miR-4252, hsa-miR-3974, hsa-miR-3944-5p, hsa-miR-3941, hsa-miR-3940-3p, hsa-miR-3929, hsa-miR-4419b, hsa-miR-4478, hsa-miR-3925-3p, hsa-miR-3921, hsa-miR-4653-5p, hsa-miR-3912-5p, hsa-miR-3909, hsa-miR-3692-3p, hsa-miR-3689d, hsa-miR-6851-5p, hsa-miR-3689f, hsa-miR-3688-3p, hsa-miR-4442, hsa-miR-3680-5p, hsa-miR-3680-3p, hsa-miR-3678-3p, hsa-miR-3675-3p, hsa-miR-3672, hsa-miR-6864-3p, hsa-miR-3667-3p, hsa-miR-3665, hsa-miR-3664-5p, hsa-miR-3664-3p, hsa-miR-3660, hsa-miR-4526, hsa-miR-3658, hsa-miR-3653-3p, hsa-miR-3622b-5p, hsa-miR-3622a-5p, hsa-miR-3622a-3p, hsa-miR-3622b-3p, hsa-miR-3616-3p, hsa-miR-3614-5p, hsa-miR-3591-5p, hsa-miR-3605-3p, hsa-miR-3202, hsa-miR-3200-5p, hsa-miR-3199, hsa-miR-8052, hsa-miR-3194-5p, hsa-miR-3194-3p, hsa-miR-3190-5p, hsa-miR-3190-3p, hsa-miR-3188, hsa-miR-3183, hsa-miR-4723-3p, hsa-miR-6769b-3p, hsa-miR-3180-3p, hsa-miR-3179, hsa-miR-3177-5p, hsa-miR-3922-3p, hsa-miR-6855-5p, hsa-miR-3169, hsa-miR-3166, hsa-miR-3163, hsa-miR-3158-5p, hsa-miR-3158-3p, hsa-miR-3157-5p, hsa-miR-3154, hsa-miR-3153, hsa-miR-6733-5p, hsa-miR-6739-5p, hsa-miR-3151-5p, hsa-miR-3148, hsa-miR-3135b, hsa-miR-3127-3p, hsa-miR-6756-3p, hsa-miR-3913-5p, hsa-miR-3126-5p, hsa-miR-3126-3p, hsa-miR-3125, hsa-miR-3916, hsa-miR-6859-5p, hsa-miR-3124-3p, hsa-miR-3123, hsa-miR-3120-5p, hsa-miR-3120-3p, hsa-miR-3074-5p, hsa-miR-3064-5p, hsa-miR-3064-3p, hsa-miR-2909, hsa-miR-2861, hsa-miR-2681-5p, hsa-miR-2467-3p, hsa-miR-2276-5p, hsa-miR-2110, hsa-miR-1972, hsa-miR-1909-3p, hsa-miR-6722-3p, hsa-miR-1587, hsa-miR-3620-5p, hsa-miR-1538, hsa-miR-4745-3p, hsa-miR-1468-3p, hsa-miR-1324, hsa-miR-1323, hsa-miR-1301-3p, hsa-miR-1296-5p, hsa-miR-1288-5p, hsa-miR-1286, hsa-miR-1278, hsa-miR-1273h-3p, hsa-miR-1275, hsa-miR-4665-5p, hsa-miR-1273g-3p, hsa-miR-1273a, hsa-miR-1273c, hsa-miR-1267, hsa-miR-1266-5p, hsa-miR-1263, hsa-miR-1262, hsa-miR-4701-3p, hsa-miR-6736-5p, hsa-miR-1260a, hsa-miR-1260b, hsa-miR-1255b-2-3p, hsa-miR-1255a, hsa-miR-1255b-5p, hsa-miR-1252-5p, hsa-miR-1251-3p, hsa-miR-1249-5p, hsa-miR-6797-5p, hsa-miR-1247-3p, hsa-miR-1245a, hsa-miR-8079, hsa-miR-1234-3p, hsa-miR-7107-5p, hsa-miR-1233-5p, hsa-miR-6778-5p, hsa-miR-1233-3p, hsa-miR-1229-5p, hsa-miR-1228-5p, hsa-miR-1228-3p, hsa-miR-1227-5p, hsa-miR-1227-3p, hsa-miR-1226-5p, hsa-miR-1225-5p, hsa-miR-1225-3p, hsa-miR-1224-5p, hsa-miR-1208, hsa-miR-1207-5p, hsa-miR-4763-3p, hsa-miR-1206, hsa-miR-1202, hsa-miR-3972, hsa-miR-1199-5p, hsa-miR-6751-3p, hsa-miR-1193, hsa-miR-1184, hsa-miR-1182, hsa-miR-1178-5p, hsa-miR-943, hsa-miR-942-5p, hsa-miR-941, hsa-miR-939-3p, hsa-miR-937-5p, hsa-miR-922, hsa-miR-921, hsa-miR-892b, hsa-miR-891b, hsa-miR-891a-3p, hsa-miR-889-5p, hsa-miR-888-5p, hsa-miR-888-3p, hsa-miR-887-5p, hsa-miR-873-3p, hsa-miR-766-3p, hsa-miR-765, hsa-miR-764, hsa-miR-762, hsa-miR-4498  hsa-miR-718, hsa-miR-671-5p, hsa-miR-670-3p, hsa-miR-665, hsa-miR-661, hsa-miR-660-3p, hsa-miR-658, hsa-miR-653-5p, hsa-miR-652-5p, hsa-miR-651-3p, hsa-miR-634, hsa-miR-630, hsa-miR-629-3p, hsa-miR-626, hsa-miR-6876-3p, hsa-miR-625-5p, hsa-miR-618, hsa-miR-615-3p, hsa-miR-612, hsa-miR-1285-3p, hsa-miR-3187-5p, hsa-miR-5189-5p, hsa-miR-6860, hsa-miR-610, hsa-miR-4651, hsa-miR-606, hsa-miR-605-5p, hsa-miR-603, hsa-miR-599, hsa-miR-597-5p, hsa-miR-596, hsa-miR-592, hsa-miR-583, hsa-miR-582-3p, hsa-miR-4676-5p, hsa-miR-574-5p, hsa-miR-573, hsa-miR-3616-5p, hsa-miR-569, hsa-miR-564, hsa-miR-561-3p, hsa-miR-551b-5p, hsa-miR-550b-3p, hsa-miR-550a-3-5p, hsa-miR-550a-5p, hsa-miR-1271-3p, hsa-miR-548s, hsa-miR-548p, hsa-miR-548n, hsa-miR-548m, hsa-miR-548az-5p, hsa-miR-548t-5p, hsa-miR-548aq-5p, hsa-miR-548ay-5p, hsa-miR-548bb-5p, hsa-miR-548d-5p, hsa-miR-548y, hsa-miR-548at-5p, hsa-miR-548ao-5p, hsa-miR-548ax, hsa-miR-548ag, hsa-miR-548ai, hsa-miR-548ba, hsa-miR-570-5p, hsa-miR-545-5p, hsa-miR-544b, hsa-miR-544a, hsa-miR-539-3p, hsa-miR-532-3p, hsa-miR-522-3p, hsa-miR-520g-5p, hsa-miR-520g-3p, hsa-miR-519d-5p, hsa-miR-518a-5p, hsa-miR-527, hsa-miR-512-5p, hsa-miR-511-5p, hsa-miR-511-3p, hsa-miR-510-5p, hsa-miR-508-5p, hsa-miR-507, hsa-miR-557, hsa-miR-497-3p, hsa-miR-495-3p, hsa-miR-493-5p, hsa-miR-493-3p, hsa-miR-491-5p, hsa-miR-488-3p, hsa-miR-485-5p, hsa-miR-484, hsa-miR-483-3p.2, hsa-miR-455-3p.2, hsa-miR-454-5p, hsa-miR-449b-3p, hsa-miR-432-5p, hsa-miR-411-3p, hsa-miR-383-5p.2, hsa-miR-383-5p.1, hsa-miR-383-3p, hsa-miR-382-5p, hsa-miR-381-3p, hsa-miR-378g, hsa-miR-371b-5p, hsa-miR-373-5p, hsa-miR-616-5p, hsa-miR-372-5p, hsa-miR-370-3p, hsa-miR-363-5p, hsa-miR-6745, hsa-miR-342-3p, hsa-miR-342-5p, hsa-miR-335-5p, hsa-miR-330-3p, hsa-miR-329-3p, hsa-miR-328-3p, hsa-miR-320e, hsa-miR-296-5p, hsa-miR-223-5p, hsa-miR-222-5p, hsa-miR-221-5p, hsa-miR-8073, hsa-miR-219a-2-3p, hsa-miR-216b-3p, hsa-miR-216a-5p, hsa-miR-215-3p, hsa-miR-214-5p, hsa-miR-214-3p, hsa-miR-3619-5p, hsa-miR-211-3p, hsa-miR-203b-5p, hsa-miR-6718-5p, hsa-miR-186-3p, hsa-miR-183-5p.1, hsa-miR-150-5p, hsa-miR-150-3p, hsa-miR-149-5p, hsa-miR-149-3p, hsa-miR-4728-5p, hsa-miR-6785-5p, hsa-miR-6883-5p, hsa-miR-147a, hsa-miR-142-5p, hsa-miR-142-3p.2, hsa-miR-142-3p.1, hsa-miR-141-3p, hsa-miR-140-5p, hsa-miR-140-3p.2, hsa-miR-140-3p.1, hsa-miR-136-5p, hsa-miR-135b-3p, hsa-miR-134-5p, hsa-miR-134-3p, hsa-miR-128-1-5p, hsa-miR-124-5p, hsa-miR-124-3p.2, hsa-miR-506-3p, hsa-miR-124-3p.1, hsa-miR-95-5p, hsa-miR-92a-2-5p, hsa-miR-34b-3p, hsa-miR-34a-3p, hsa-miR-33a-3p, hsa-miR-32-3p, hsa-miR-30b-3p, hsa-miR-1273h-5p, hsa-miR-6779-5p, hsa-miR-6780a-5p, hsa-miR-25-3p, hsa-miR-32-5p, hsa-miR-363-3p, hsa-miR-367-3p, hsa-miR-22-3p, hsa-miR-20b-3p, hsa-miR-17-5p, hsa-miR-17-3p, hsa-miR-15b-3p, hsa-miR-1-3p, hsa-let-7c-3p, hsa-miR-8485, hsa-miR-8077, hsa-miR-8070, hsa-miR-8069, hsa-miR-8066, hsa-miR-8064, hsa-miR-8065, hsa-miR-8062, hsa-miR-8055, hsa-miR-7977, hsa-miR-7855-5p, hsa-miR-7845-5p, hsa-miR-7846-3p, hsa-miR-7843-3p, hsa-miR-7703, hsa-miR-7162-3p, hsa-miR-7515, hsa-miR-7161-3p, hsa-miR-7160-3p, hsa-miR-7160-5p, hsa-miR-7156-3p, hsa-miR-7150, hsa-miR-7111-3p, hsa-miR-7112-5p, hsa-miR-7110-3p, hsa-miR-7109-5p, hsa-miR-7108-5p, hsa-miR-7106-5p, hsa-miR-6895-3p, hsa-miR-6894-5p, hsa-miR-6894-3p, hsa-miR-6888-3p, hsa-miR-6886-5p, hsa-miR-6886-3p, hsa-miR-6882-3p, hsa-miR-6881-3p, hsa-miR-6878-5p, hsa-miR-6877-5p, hsa-miR-6873-3p, hsa-miR-6873-5p, hsa-miR-6868-3p, hsa-miR-6865-3p, hsa-miR-6861-5p, hsa-miR-6857-5p, hsa-miR-6853-5p, hsa-miR-6851-3p, hsa-miR-6850-5p, hsa-miR-6849-3p, hsa-miR-6847-5p, hsa-miR-6846-3p, hsa-miR-6845-3p, hsa-miR-6842-3p, hsa-miR-6840-3p, hsa-miR-6838-3p, hsa-miR-6836-3p, hsa-miR-6832-3p, hsa-miR-6830-5p, hsa-miR-6830-3p, hsa-miR-6828-5p, hsa-miR-6823-5p, hsa-miR-6818-5p, hsa-miR-6818-3p, hsa-miR-6817-3p, hsa-miR-6815-3p, hsa-miR-6815-5p, hsa-miR-6865-5p, hsa-miR-6811-3p, hsa-miR-6810-5p, hsa-miR-6807-5p, hsa-miR-6804-5p, hsa-miR-6804-3p, hsa-miR-6802-3p, hsa-miR-6799-5p, hsa-miR-6798-5p, hsa-miR-6796-5p, hsa-miR-6791-3p, hsa-miR-6829-3p, hsa-miR-6787-3p, hsa-miR-6786-5p, hsa-miR-6783-5p, hsa-miR-6784-3p, hsa-miR-6862-3p, hsa-miR-6780a-3p, hsa-miR-6776-5p, hsa-miR-6771-3p, hsa-miR-6772-5p, hsa-miR-6770-5p, hsa-miR-6769b-5p, hsa-miR-6769a-3p, hsa-miR-6760-5p, hsa-miR-6759-5p, hsa-miR-6756-5p, hsa-miR-6766-5p, hsa-miR-6755-5p, hsa-miR-6755-3p, hsa-miR-6753-5p, hsa-miR-6751-5p, hsa-miR-6803-5p, hsa-miR-6748-5p, hsa-miR-6748-3p, hsa-miR-6739-3p, hsa-miR-6740-3p, hsa-miR-6738-3p, hsa-miR-6737-5p, hsa-miR-6812-5p, hsa-miR-6819-5p, hsa-miR-6735-3p, hsa-miR-6731-5p, hsa-miR-8085, hsa-miR-6730-5p, hsa-miR-6728-3p, hsa-miR-6724-5p, hsa-miR-6773-5p, hsa-miR-6721-5p, hsa-miR-6720-5p, hsa-miR-6717-5p, hsa-miR-6516-5p, hsa-miR-6514-3p, hsa-miR-6510-5p, hsa-miR-6505-5p, hsa-miR-6165, hsa-miR-6134, hsa-miR-6132, hsa-miR-6836-5p, hsa-miR-6124, hsa-miR-6089, hsa-miR-6079, hsa-miR-6078, hsa-miR-6071, hsa-miR-5707, hsa-miR-5702, hsa-miR-5700, hsa-miR-5694, hsa-miR-5690, hsa-miR-5689, hsa-miR-5683, hsa-miR-5681a, hsa-miR-5587-5p, hsa-miR-5586-5p, hsa-miR-5585-5p, hsa-miR-5585-3p, hsa-miR-5583-3p, hsa-miR-5582-5p, hsa-miR-5580-5p, hsa-miR-5579-5p, hsa-miR-5572, hsa-miR-5571-5p, hsa-miR-5197-3p, hsa-miR-5195-5p, hsa-miR-5192, hsa-miR-5193, hsa-miR-5187-5p, hsa-miR-5100, hsa-miR-5091, hsa-miR-5007-3p, hsa-miR-5006-5p, hsa-miR-5001-3p, hsa-miR-4802-5p, hsa-miR-4796-3p, hsa-miR-4795-5p, hsa-miR-4792, hsa-miR-4789-3p, hsa-miR-4786-3p, hsa-miR-4786-5p, hsa-miR-4779, hsa-miR-4778-5p, hsa-miR-4771, hsa-miR-4772-3p, hsa-miR-4768-3p, hsa-miR-6833-3p, hsa-miR-4767, hsa-miR-4766-5p, hsa-miR-4756-3p, hsa-miR-4755-3p, hsa-miR-4749-3p, hsa-miR-4751, hsa-miR-4747-5p, hsa-miR-5196-5p, hsa-miR-4747-3p, hsa-miR-4740-5p, hsa-miR-4739, hsa-miR-4736, hsa-miR-4733-3p, hsa-miR-4727-3p, hsa-miR-4727-5p, hsa-miR-4722-5p, hsa-miR-5698, hsa-miR-6870-5p, hsa-miR-7111-5p, hsa-miR-4720-5p, hsa-miR-4799-3p, hsa-miR-5588-5p, hsa-miR-4715-3p, hsa-miR-4713-5p, hsa-miR-4710, hsa-miR-4711-3p, hsa-miR-4708-5p, hsa-miR-4697-3p, hsa-miR-4695-5p, hsa-miR-4691-5p, hsa-miR-6792-3p, hsa-miR-4691-3p, hsa-miR-5685, hsa-miR-6858-5p, hsa-miR-4688, hsa-miR-6743-5p, hsa-miR-4687-3p, hsa-miR-4683, hsa-miR-4682, hsa-miR-4679, hsa-miR-4677-3p, hsa-miR-4674, hsa-miR-4667-5p, hsa-miR-4700-5p |
| HSALNT0103091 | hsa-miR-4652-3p, hsa-miR-4509, hsa-miR-4464, hsa-miR-4748, hsa-miR-3177-5p, hsa-miR-3119, hsa-miR-1264, hsa-miR-1243, hsa-miR-892a, hsa-miR-664a-3p, hsa-miR-653-3p, hsa-miR-624-3p, hsa-miR-624-3p, hsa-miR-509-3p, hsa-miR-506-5p, hsa-miR-493-3p, hsa-miR-450a-2-3p, hsa-miR-384, hsa-miR-378j, hsa-miR-6839-5p, hsa-miR-329-5p, hsa-miR-210-3p, hsa-miR-204-5p, hsa-miR-211-5p, hsa-miR-183-3p, hsa-miR-27a-5p, hsa-miR-9-5p, hsa-let-7c-3p, hsa-miR-7702, hsa-miR-6857-5p, hsa-miR-6844, hsa-miR-6834-3p, hsa-miR-6740-5p, hsa-miR-4713-3p, hsa-miR-4693-5p, hsa-miR-4680-3p |
| HSALNT0116512 | hsa-miR-4666a-5p, hsa-miR-4643, hsa-miR-4638-3p, hsa-miR-4539, hsa-miR-4528, hsa-miR-4515, hsa-miR-4509, hsa-miR-4495, hsa-miR-4482-3p, hsa-miR-4477a, hsa-miR-4422, hsa-miR-4325, hsa-miR-4310, hsa-miR-7157-5p, hsa-miR-4307, hsa-miR-4308, hsa-miR-4302, hsa-miR-4282, hsa-miR-4277, hsa-miR-4259, hsa-miR-3976, hsa-miR-3941, hsa-miR-3940-5p, hsa-miR-4507, hsa-miR-3925-3p, hsa-miR-3918, hsa-miR-3688-3p, hsa-miR-3686, hsa-miR-3671, hsa-miR-3646, hsa-miR-3614-5p, hsa-miR-3613-3p, hsa-miR-3194-3p, hsa-miR-3191-5p, hsa-miR-3187-3p, hsa-miR-3173-5p, hsa-miR-3169, hsa-miR-3163, hsa-miR-3156-3p, hsa-miR-3152-5p, hsa-miR-3148, hsa-miR-3149, hsa-miR-3147, hsa-miR-3145-3p, hsa-miR-3143, hsa-miR-3125, hsa-miR-3916, hsa-miR-6859-5p, hsa-miR-3121-5p, hsa-miR-3120-3p, hsa-miR-3117-3p, hsa-miR-3064-5p, hsa-miR-2115-5p, hsa-miR-2053, hsa-miR-1537-5p, hsa-miR-1468-3p, hsa-miR-1294, hsa-miR-1291, hsa-miR-6775-3p, hsa-miR-1288-5p, hsa-miR-1283, hsa-miR-1277-5p, hsa-miR-1276, hsa-miR-1256, hsa-miR-1253, hsa-miR-1251-3p, hsa-miR-1244, hsa-miR-1237-3p, hsa-miR-1229-5p, hsa-miR-1228-3p, hsa-miR-1224-5p, hsa-miR-1206, hsa-miR-1200, hsa-miR-944, hsa-miR-942-5p, hsa-miR-892b, hsa-miR-873-5p.2, hsa-miR-760, hsa-miR-656-3p, hsa-miR-649, hsa-miR-648, hsa-miR-634, hsa-miR-629-3p, hsa-miR-617, hsa-miR-616-3p, hsa-miR-614, hsa-miR-607, hsa-miR-600, hsa-miR-595, hsa-miR-580-5p, hsa-miR-579-3p, hsa-miR-568, hsa-miR-555, hsa-miR-548v, hsa-miR-548u, hsa-miR-7161-5p, hsa-miR-548p, hsa-miR-548c-3p, hsa-miR-548az-5p, hsa-miR-548t-5p, hsa-miR-548a-3p, hsa-miR-548ar-3p, hsa-miR-548az-3p, hsa-miR-548e-3p, hsa-miR-548f-3p, hsa-miR-548at-5p, hsa-miR-548as-3p, hsa-miR-548aa, hsa-miR-548ap-3p, hsa-miR-548t-3p, hsa-miR-548ah-5p, hsa-miR-548bb-3p, hsa-miR-548d-3p, hsa-miR-545-5p, hsa-miR-545-3p, hsa-miR-544b, hsa-miR-544a, hsa-miR-539-3p, hsa-miR-532-5p, hsa-miR-532-3p, hsa-miR-524-5p, hsa-miR-522-3p, hsa-miR-518a-5p, hsa-miR-527, hsa-miR-514a-3p, hsa-miR-513a-5p, hsa-miR-3606-3p  hsa-miR-511-5p, hsa-miR-511-3p, hsa-miR-503-3p, hsa-miR-501-5p, hsa-miR-494-3p, hsa-miR-493-5p, hsa-miR-491-3p, hsa-miR-451b, hsa-miR-450b-5p, hsa-miR-421, hsa-miR-410-3p, hsa-miR-380-3p, hsa-miR-378j, hsa-miR-6839-5p, hsa-miR-375, hsa-miR-374a-3p, hsa-miR-371b-5p, hsa-miR-373-5p, hsa-miR-616-5p, hsa-miR-361-5p, hsa-miR-340-5p, hsa-miR-338-5p, hsa-miR-337-3p, hsa-miR-328-3p, hsa-miR-326, hsa-miR-323b-3p, hsa-miR-302a-5p, hsa-miR-223-5p, hsa-miR-222-3p, hsa-miR-217, hsa-miR-216a-5p, hsa-miR-205-3p, hsa-miR-190a-3p, hsa-miR-186-5p, hsa-miR-183-3p, hsa-miR-153-5p, hsa-miR-149-5p, hsa-miR-148b-5p, hsa-miR-147a, hsa-miR-146b-3p, hsa-miR-146a-3p, hsa-miR-144-3p, hsa-miR-142-5p, hsa-miR-138-2-3p, hsa-miR-136-5p, hsa-miR-133a-3p.2, hsa-miR-133b, hsa-miR-128-3p, hsa-miR-126-5p, hsa-miR-105-5p, hsa-miR-105-3p, hsa-miR-103a-2-5p, hsa-miR-101-3p.1, hsa-miR-33a-3p, hsa-miR-32-3p, hsa-miR-26b-3p, hsa-miR-25-3p, hsa-miR-32-5p, hsa-miR-363-3p, hsa-miR-367-3p, hsa-miR-22-5p, hsa-miR-20a-3p, hsa-miR-17-5p, hsa-miR-16-2-3p, hsa-miR-195-3p, hsa-let-7c-3p, hsa-let-7a-3p, hsa-miR-98-3p, hsa-miR-8485, hsa-miR-8087, hsa-miR-8076, hsa-miR-7974, hsa-miR-7854-3p, hsa-miR-7703, hsa-miR-7162-3p, hsa-miR-6895-5p, hsa-miR-6861-5p, hsa-miR-6853-3p, hsa-miR-6847-5p, hsa-miR-6844, hsa-miR-6830-3p, hsa-miR-6828-3p, hsa-miR-6826-5p, hsa-miR-6821-3p, hsa-miR-6813-3p, hsa-miR-6809-3p, hsa-miR-6789-5p, hsa-miR-6779-3p, hsa-miR-6768-5p, hsa-miR-6765-3p, hsa-miR-6739-3p, hsa-miR-6740-3p, hsa-miR-6516-3p, hsa-miR-6509-5p, hsa-miR-6500-3p, hsa-miR-6813-5p, hsa-miR-5700, hsa-miR-5696, hsa-miR-5694, hsa-miR-5692a, hsa-miR-5589-5p, hsa-miR-5589-3p, hsa-miR-5584-3p, hsa-miR-5580-3p, hsa-miR-5197-3p, hsa-miR-5088-3p, hsa-miR-5007-3p, hsa-miR-5004-5p, hsa-miR-4803, hsa-miR-4789-5p, hsa-miR-4789-3p, hsa-miR-4778-5p, hsa-miR-4778-3p, hsa-miR-4777-3p, hsa-miR-4775, hsa-miR-4774-3p, hsa-miR-4766-5p, hsa-miR-8061, hsa-miR-4756-3p, hsa-miR-4753-3p, hsa-miR-4744, hsa-miR-4735-5p, hsa-miR-4729, hsa-miR-4720-3p, hsa-miR-4714-5p, hsa-miR-4713-5p, hsa-miR-4709-3p, hsa-miR-4699-3p, hsa-miR-4699-5p, hsa-miR-4698, hsa-miR-4694-3p, hsa-miR-4688, hsa-miR-6743-5p, hsa-miR-4687-3p, hsa-miR-4672, hsa-miR-4668-3p |
| HSALNT0252034 | hsa-miR-4659a-3p, hsa-miR-4659b-3p, hsa-miR-4655-3p, hsa-miR-4645-5p, hsa-miR-4673, hsa-miR-4632-3p, hsa-miR-4532, hsa-miR-4524a-3p, hsa-miR-4514, hsa-miR-4692, hsa-miR-4508, hsa-miR-4468, hsa-miR-4632-5p, hsa-miR-6735-5p, hsa-miR-6879-5p, hsa-miR-4428, hsa-miR-4326, hsa-miR-4325, hsa-miR-4324, hsa-miR-4314, hsa-miR-4311, hsa-miR-4299, hsa-miR-6791-5p, hsa-miR-4283, hsa-miR-4281, hsa-miR-4275, hsa-miR-4273, hsa-miR-7156-5p, hsa-miR-4271, hsa-miR-4725-3p, hsa-miR-6780b-5p, hsa-miR-4251, hsa-miR-3940-3p, hsa-miR-3937, hsa-miR-3917, hsa-miR-3692-5p, hsa-miR-3663-3p, hsa-miR-3653-5p, hsa-miR-3202, hsa-miR-3192-5p, hsa-miR-3191-5p, hsa-miR-3173-3p, hsa-miR-3171, hsa-miR-3160-3p, hsa-miR-3153, hsa-miR-6739-5p, hsa-miR-3151-5p, hsa-miR-3135b, hsa-miR-3127-3p, hsa-miR-6756-3p, hsa-miR-3916, hsa-miR-3074-3p, hsa-miR-2277-5p, hsa-miR-2117, hsa-miR-2114-5p, hsa-miR-1976, hsa-miR-1910-5p, hsa-miR-6722-3p, hsa-miR-1471, hsa-miR-1468-5p, hsa-miR-1306-5p, hsa-miR-1299, hsa-miR-1298-3p, hsa-miR-1292-5p, hsa-miR-1289, hsa-miR-1286, hsa-miR-1273c, hsa-miR-1248, hsa-miR-1247-3p, hsa-miR-1238-3p, hsa-miR-1237-3p, hsa-miR-1227-5p, hsa-miR-1207-5p, hsa-miR-4763-3p, hsa-miR-1205, hsa-miR-1204, hsa-miR-1200, hsa-miR-1184, hsa-miR-940, hsa-miR-6808-5p, hsa-miR-6893-5p, hsa-miR-766-5p, hsa-miR-765, hsa-miR-744-5p, hsa-miR-668-5p, hsa-miR-663b, hsa-miR-660-3p, hsa-miR-645, hsa-miR-638, hsa-miR-619-5p, hsa-miR-6506-5p, hsa-miR-617, hsa-miR-607, hsa-miR-593-3p, hsa-miR-585-5p, hsa-miR-548v, hsa-miR-539-5p, hsa-miR-519d-5p, hsa-miR-518e-3p, hsa-miR-518c-5p, hsa-miR-515-5p, hsa-miR-519e-5p, hsa-miR-512-5p, hsa-miR-501-3p, hsa-miR-502-3p, hsa-miR-500a-3p, hsa-miR-455-3p.2, hsa-miR-450b-3p, hsa-miR-769-3p, hsa-miR-423-3p, hsa-miR-370-3p, hsa-miR-361-5p, hsa-miR-340-3p, hsa-miR-6827-3p, hsa-miR-331-3p, hsa-miR-324-5p, hsa-miR-324-3p, hsa-miR-1913, hsa-miR-298, hsa-miR-219a-1-3p, hsa-miR-216b-3p, hsa-miR-216a-5p, hsa-miR-210-5p, hsa-miR-204-3p, hsa-miR-4646-5p, hsa-miR-186-5p, hsa-miR-186-3p, hsa-miR-144-5p, hsa-miR-141-5p, hsa-miR-133a-3p.2, hsa-miR-133b, hsa-miR-133a-3p.1, hsa-miR-132-3p, hsa-miR-212-3p, hsa-miR-129-5p, hsa-miR-128-1-5p, hsa-miR-6780a-5p, hsa-miR-28-5p, hsa-miR-708-5p, hsa-miR-22-5p, hsa-miR-10b-3p, hsa-miR-8064, hsa-miR-7706, hsa-miR-7111-3p, hsa-miR-7110-3p, hsa-miR-6895-3p, hsa-miR-6880-3p, hsa-miR-6875-3p, hsa-miR-6873-3p, hsa-miR-6870-3p, hsa-miR-6869-5p, hsa-miR-6851-3p, hsa-miR-6845-3p, hsa-miR-6838-3p, hsa-miR-6830-3p, hsa-miR-6826-3p, hsa-miR-6809-3p, hsa-miR-6803-3p, hsa-miR-6796-5p, hsa-miR-6787-3p, hsa-miR-6784-5p, hsa-miR-6780b-3p, hsa-miR-6777-5p, hsa-miR-6768-5p, hsa-miR-6763-3p, hsa-miR-6761-5p, hsa-miR-6758-5p, hsa-miR-6856-5p, hsa-miR-6757-5p, hsa-miR-6747-3p, hsa-miR-6746-3p, hsa-miR-6738-3p, hsa-miR-6504-3p, hsa-miR-6500-3p, hsa-miR-6125, hsa-miR-6079, hsa-miR-5690, hsa-miR-5587-3p, hsa-miR-5582-3p, hsa-miR-5571-3p, hsa-miR-5197-3p, hsa-miR-5088-3p, hsa-miR-5000-5p, hsa-miR-4783-5p, hsa-miR-4779, hsa-miR-4778-3p, hsa-miR-4768-5p, hsa-miR-6833-3p, hsa-miR-4762-5p, hsa-miR-4749-3p, hsa-miR-4747-5p, hsa-miR-5196-5p, hsa-miR-4746-5p, hsa-miR-4733-5p, hsa-miR-4726-3p, hsa-miR-4722-5p, hsa-miR-4706, hsa-miR-4749-5p, hsa-miR-6743-5p, hsa-miR-4682, hsa-miR-4677-5p, hsa-miR-4668-5p |
| HSALNT0238453 | hsa-miR-4661-3p, hsa-miR-4658, hsa-miR-6790-5p, hsa-miR-4650-5p, hsa-miR-4649-3p, hsa-miR-4536-5p, hsa-miR-4531, hsa-miR-4493, hsa-miR-4476, hsa-miR-6876-5p, hsa-miR-4457, hsa-miR-4437, hsa-miR-4436b-3p, hsa-miR-4632-5p, hsa-miR-6735-5p, hsa-miR-6879-5p, hsa-miR-7843-5p, hsa-miR-4434, hsa-miR-4433b-5p, hsa-miR-4433a-5p, hsa-miR-4424, hsa-miR-4422, hsa-miR-4419a, hsa-miR-4510, hsa-miR-6127, hsa-miR-6129, hsa-miR-6130, hsa-miR-6133, hsa-miR-4330, hsa-miR-4314, hsa-miR-4293, hsa-miR-4291, hsa-miR-4290, hsa-miR-4282, hsa-miR-4283, hsa-miR-4265, hsa-miR-4296, hsa-miR-4322, hsa-miR-4260, hsa-miR-4255, hsa-miR-3936, hsa-miR-3924, hsa-miR-3910, hsa-miR-3692-3p, hsa-miR-3688-5p, hsa-miR-3682-5p, hsa-miR-3671, hsa-miR-3667-3p, hsa-miR-3659, hsa-miR-3616-3p, hsa-miR-3614-5p, hsa-miR-3200-5p, hsa-miR-3194-5p, hsa-miR-3185, hsa-miR-3180-5p, hsa-miR-3179, hsa-miR-3177-5p, hsa-miR-3176, hsa-miR-3922-3p, hsa-miR-3169, hsa-miR-3166, hsa-miR-3164, hsa-miR-3163, hsa-miR-3160-5p, hsa-miR-3154, hsa-miR-3150b-3p, hsa-miR-3148, hsa-miR-3138, hsa-miR-3137, hsa-miR-3123, hsa-miR-2053, hsa-miR-1915-3p, hsa-miR-6764-5p, hsa-miR-1914-5p, hsa-miR-1323, hsa-miR-1302, hsa-miR-4298, hsa-miR-1303, hsa-miR-1298-3p, hsa-miR-1294, hsa-miR-1291, hsa-miR-6775-3p, hsa-miR-1288-5p, hsa-miR-1285-5p, hsa-miR-1277-5p, hsa-miR-1273g-3p, hsa-miR-1272, hsa-miR-1266-5p, hsa-miR-4518, hsa-miR-1260a, hsa-miR-1260b, hsa-miR-1252-5p, hsa-miR-1246, hsa-miR-1245a, hsa-miR-8079, hsa-miR-1231, hsa-miR-1227-5p, hsa-miR-1226-5p, hsa-miR-1226-3p, hsa-miR-1202, hsa-miR-3972, hsa-miR-1182, hsa-miR-1178-5p, hsa-miR-944, hsa-miR-942-3p, hsa-miR-922, hsa-miR-891a-3p, hsa-miR-874-3p, hsa-miR-764, hsa-miR-664a-5p, hsa-miR-4794, hsa-miR-664a-3p, hsa-miR-651-3p, hsa-miR-627-3p, hsa-miR-619-5p, hsa-miR-6506-5p, hsa-miR-3131, hsa-miR-610, hsa-miR-607, hsa-miR-606, hsa-miR-605-5p, hsa-miR-605-3p, hsa-miR-597-5p, hsa-miR-592, hsa-miR-589-5p, hsa-miR-588, hsa-miR-587, hsa-miR-586, hsa-miR-580-3p, hsa-miR-578, hsa-miR-569, hsa-miR-567, hsa-miR-561-3p, hsa-miR-552-5p, hsa-miR-551b-5p, hsa-miR-550b-3p, hsa-miR-548v, hsa-miR-548e-5p, hsa-miR-548c-3p, hsa-miR-548az-5p, hsa-miR-548a-3p, hsa-miR-548ar-3p, hsa-miR-548az-3p, hsa-miR-548e-3p, hsa-miR-548f-3p, hsa-miR-548av-3p, hsa-miR-548an, hsa-miR-544b, hsa-miR-542-3p, hsa-miR-532-5p, hsa-miR-526b-5p, hsa-miR-525-5p, hsa-miR-518a-5p, hsa-miR-527, hsa-miR-515-5p, hsa-miR-519e-5p, hsa-miR-513b-5p, hsa-miR-513a-5p, hsa-miR-512-5p, hsa-miR-510-5p, hsa-miR-505-3p.1, hsa-miR-500a-5p, hsa-miR-495-5p, hsa-miR-495-3p, hsa-miR-493-5p, hsa-miR-491-3p, hsa-miR-484, hsa-miR-449b-3p, hsa-miR-433-3p, hsa-miR-421, hsa-miR-383-5p.2, hsa-miR-383-5p.1, hsa-miR-383-3p, hsa-miR-382-5p, hsa-miR-376c-3p, hsa-miR-376a-5p, hsa-miR-374c-3p, hsa-miR-371b-5p, hsa-miR-373-5p, hsa-miR-616-5p, hsa-miR-372-5p, hsa-miR-339-5p, hsa-miR-330-3p, hsa-miR-302c-5p, hsa-miR-299-3p, hsa-miR-297, hsa-miR-222-5p, hsa-miR-219a-1-3p, hsa-miR-216a-5p, hsa-miR-214-3p, hsa-miR-3619-5p, hsa-miR-198, hsa-miR-194-5p, hsa-miR-192-5p, hsa-miR-215-5p, hsa-miR-190a-3p, hsa-miR-188-3p, hsa-miR-187-5p, hsa-miR-183-5p.1, hsa-miR-153-5p, hsa-miR-150-3p, hsa-miR-146b-3p, hsa-miR-142-3p.2, hsa-miR-142-3p.1, hsa-miR-141-5p, hsa-miR-141-3p, hsa-miR-139-5p, hsa-miR-135b-3p, hsa-miR-132-5p, hsa-miR-128-3p, hsa-miR-127-5p, hsa-miR-125b-2-3p, hsa-miR-124-3p.2, hsa-miR-506-3p, hsa-miR-124-3p.1, hsa-miR-105-5p, hsa-miR-103b, hsa-miR-18b-3p, hsa-miR-18a-3p, hsa-miR-16-1-3p, hsa-miR-10b-3p, hsa-miR-7-5p, hsa-miR-98-5p, hsa-miR-8485, hsa-miR-8084, hsa-miR-8071, hsa-miR-8065, hsa-miR-8055, hsa-miR-7854-3p, hsa-miR-7843-3p, hsa-miR-7705, hsa-miR-7162-3p, hsa-miR-7515, hsa-miR-7114-5p, hsa-miR-6886-3p, hsa-miR-6873-5p, hsa-miR-6865-3p, hsa-miR-6856-3p, hsa-miR-6857-3p, hsa-miR-6854-5p, hsa-miR-6849-3p, hsa-miR-6837-3p, hsa-miR-6834-3p, hsa-miR-6833-5p, hsa-miR-6832-3p, hsa-miR-6826-5p, hsa-miR-6815-3p, hsa-miR-6814-5p, hsa-miR-6804-5p, hsa-miR-6800-5p, hsa-miR-6791-3p, hsa-miR-6782-5p, hsa-miR-6780a-3p, hsa-miR-6776-3p, hsa-miR-6768-3p, hsa-miR-6768-5p, hsa-miR-6760-5p, hsa-miR-6734-3p, hsa-miR-6729-3p, hsa-miR-6723-5p, hsa-miR-6721-5p, hsa-miR-6720-5p, hsa-miR-6509-3p, hsa-miR-6504-3p, hsa-miR-6501-5p, hsa-miR-5693, hsa-miR-5681a, hsa-miR-5586-5p, hsa-miR-5585-3p, hsa-miR-5584-5p, hsa-miR-5581-3p, hsa-miR-5579-5p, hsa-miR-5579-3p, hsa-miR-5196-3p, hsa-miR-5100, hsa-miR-5011-5p, hsa-miR-5008-3p, hsa-miR-6737-3p, hsa-miR-7157-3p, hsa-miR-5008-5p, hsa-miR-5007-3p, hsa-miR-4795-3p, hsa-miR-4789-3p, hsa-miR-4786-3p, hsa-miR-4760-5p, hsa-miR-8061, hsa-miR-4755-5p, hsa-miR-5006-3p, hsa-miR-4755-3p, hsa-miR-4740-5p, hsa-miR-4733-5p, hsa-miR-4731-3p, hsa-miR-4801, hsa-miR-4709-5p, hsa-miR-4708-3p, hsa-miR-4705, hsa-miR-4697-3p, hsa-miR-4698, hsa-miR-4691-3p, hsa-miR-4682, hsa-miR-4668-5p, hsa-miR-4668-3p, hsa-miR-4667-3p |
| HSALNT0017759 | hsa-miR-4509, hsa-miR-4490, hsa-miR-4474-3p, hsa-miR-4473, hsa-miR-4470, hsa-miR-4320, hsa-miR-4263, hsa-miR-3910, hsa-miR-3911, hsa-miR-3690, hsa-miR-3685, hsa-miR-3682-3p, hsa-miR-3198, hsa-miR-4309, hsa-miR-3161, hsa-miR-3136-5p, hsa-miR-4439, hsa-miR-3119, hsa-miR-1909-3p, hsa-miR-6722-3p, hsa-miR-1289, hsa-miR-1272, hsa-miR-1255b-2-3p, hsa-miR-1254, hsa-miR-3116, hsa-miR-1207-5p, hsa-miR-4763-3p, hsa-miR-892a, hsa-miR-660-3p, hsa-miR-644a, hsa-miR-628-5p, hsa-miR-612, hsa-miR-1285-3p, hsa-miR-3187-5p, hsa-miR-5189-5p, hsa-miR-6860, hsa-miR-586, hsa-miR-578, hsa-miR-561-3p, hsa-miR-548g-3p, hsa-miR-548an, hsa-miR-522-3p, hsa-miR-512-3p, hsa-miR-493-3p, hsa-miR-452-3p, hsa-miR-320e, hsa-miR-218-2-3p, hsa-miR-152-5p, hsa-miR-147a, hsa-miR-105-5p, hsa-miR-8060, hsa-miR-7975, hsa-miR-7156-3p, hsa-miR-7150, hsa-miR-6892-5p, hsa-miR-6880-5p, hsa-miR-6878-5p, hsa-miR-6802-5p, hsa-miR-6798-5p, hsa-miR-6792-5p, hsa-miR-6777-3p, hsa-miR-6760-5p, hsa-miR-6752-5p, hsa-miR-6842-5p, hsa-miR-7110-5p, hsa-miR-6732-5p, hsa-miR-6731-5p, hsa-miR-8085, hsa-miR-6080, hsa-miR-5700, hsa-miR-5192, hsa-miR-4785, hsa-miR-4776-5p, hsa-miR-4738-3p, hsa-miR-4736, hsa-miR-4730, hsa-miR-4728-3p, hsa-miR-4724-5p, hsa-miR-4719, hsa-miR-6794-5p |
| HSALNT0015061 | hsa-miR-4643, hsa-miR-4640-3p, hsa-miR-4637, hsa-miR-4536-5p, hsa-miR-4533, hsa-miR-4524a-3p, hsa-miR-4509, hsa-miR-4486, hsa-miR-4461, hsa-miR-4457, hsa-miR-4436b-3p, hsa-miR-4632-5p, hsa-miR-6735-5p, hsa-miR-6879-5p  hsa-miR-7843-5p, hsa-miR-4433b-3p, hsa-miR-4428, hsa-miR-4422, hsa-miR-4289, hsa-miR-4282, hsa-miR-4725-3p, hsa-miR-6780b-5p, hsa-miR-4266, hsa-miR-3976, hsa-miR-3975, hsa-miR-3941, hsa-miR-3936, hsa-miR-6831-5p, hsa-miR-3922-5p, hsa-miR-3682-5p, hsa-miR-3675-5p, hsa-miR-3613-3p, hsa-miR-3202, hsa-miR-3179, hsa-miR-3175, hsa-miR-3173-3p, hsa-miR-6891-5p, hsa-miR-3163, hsa-miR-3162-5p, hsa-miR-3154, hsa-miR-3153, hsa-miR-6733-5p, hsa-miR-6739-5p, hsa-miR-3121-3p, hsa-miR-2115-3p, hsa-miR-2114-3p, hsa-miR-1292-5p, hsa-miR-1277-5p, hsa-miR-1273f, hsa-miR-1249-5p, hsa-miR-6797-5p, hsa-miR-1228-3p, hsa-miR-1224-5p, hsa-miR-1183, hsa-miR-765, hsa-miR-708-3p, hsa-miR-661, hsa-miR-652-5p, hsa-miR-633, hsa-miR-627-3p, hsa-miR-624-3p, hsa-miR-622, hsa-miR-6506-5p, hsa-miR-583, hsa-miR-568, hsa-miR-558, hsa-miR-552-3p, hsa-miR-7161-5p, hsa-miR-548m, hsa-miR-548g-3p, hsa-miR-548e-5p, hsa-miR-548a-3p, hsa-miR-548ar-3p, hsa-miR-548az-3p, hsa-miR-548e-3p, hsa-miR-548f-3p, hsa-miR-548av-3p, hsa-miR-548as-3p, hsa-miR-548ae-3p, hsa-miR-548ah-3p, hsa-miR-548aj-3p, hsa-miR-548am-3p, hsa-miR-548aq-3p, hsa-miR-548j-3p, hsa-miR-548x-3p, hsa-miR-539-3p, hsa-miR-525-5p, hsa-miR-513b-3p, hsa-miR-3606-3p, hsa-miR-510-5p, hsa-miR-507, hsa-miR-557, hsa-miR-497-3p, hsa-miR-495-3p, hsa-miR-494-3p, hsa-miR-493-5p, hsa-miR-490-3p, hsa-miR-486-3p, hsa-miR-466, hsa-miR-423-5p, hsa-miR-329-3p, hsa-miR-362-3p, hsa-miR-324-5p, hsa-miR-323a-5p, hsa-miR-298, hsa-miR-204-3p, hsa-miR-4646-5p, hsa-miR-197-5p, hsa-miR-3132, hsa-miR-197-3p, hsa-miR-192-5p, hsa-miR-215-5p, hsa-miR-191-5p, hsa-miR-190a-3p, hsa-miR-185-5p, hsa-miR-153-5p, hsa-miR-142-5p, hsa-miR-135a-3p, hsa-miR-132-3p, hsa-miR-212-3p, hsa-miR-454-3p, hsa-miR-105-5p, hsa-miR-107, hsa-miR-28-3p, hsa-miR-15b-3p, hsa-miR-10a-3p, hsa-miR-9-3p, hsa-miR-1-3p, hsa-miR-206, hsa-miR-9500, hsa-miR-8485, hsa-miR-8084, hsa-miR-8080, hsa-miR-8078, hsa-miR-8065, hsa-miR-7154-3p, hsa-miR-7152-5p, hsa-miR-7109-3p, hsa-miR-6894-5p, hsa-miR-6890-5p, hsa-miR-6881-5p, hsa-miR-6839-3p, hsa-miR-6832-5p, hsa-miR-6825-5p, hsa-miR-6809-5p, hsa-miR-6802-3p, hsa-miR-6783-5p, hsa-miR-6784-3p, hsa-miR-6862-3p, hsa-miR-6782-5p, hsa-miR-6776-3p, hsa-miR-6773-3p, hsa-miR-6758-5p, hsa-miR-6856-5p, hsa-miR-6731-5p, hsa-miR-8085, hsa-miR-6729-3p, hsa-miR-6715a-3p, hsa-miR-6508-5p, hsa-miR-8067, hsa-miR-5739, hsa-miR-5692a, hsa-miR-5687, hsa-miR-5689, hsa-miR-5680, hsa-miR-5589-3p, hsa-miR-5584-5p, hsa-miR-5583-3p, hsa-miR-5093, hsa-miR-5089-3p, hsa-miR-5011-5p, hsa-miR-5010-5p, hsa-miR-5009-3p, hsa-miR-5007-5p, hsa-miR-5004-5p, hsa-miR-4999-5p, hsa-miR-4799-5p, hsa-miR-4789-5p, hsa-miR-4789-3p, hsa-miR-4779, hsa-miR-4773, hsa-miR-4756-3p, hsa-miR-4753-5p, hsa-miR-4747-5p, hsa-miR-5196-5p, hsa-miR-4744, hsa-miR-4731-3p, hsa-miR-4723-5p, hsa-miR-5698, hsa-miR-6870-5p, hsa-miR-7111-5p, hsa-miR-4719, hsa-miR-4718, hsa-miR-4717-3p, hsa-miR-4716-3p, hsa-miR-6794-5p, hsa-miR-4711-5p, hsa-miR-4712-3p, hsa-miR-4694-3p, hsa-miR-6858-5p |
