# Supplementary material for: Functional lncRNA-miRNA-mRNA Networks in Response to Baicalein Treatment in Hepatocellular Carcinoma
Source: Biomed Res Int. 2021 Jan 14;2021:8844261. doi: 10.1155/2021/8844261 (PMC7825356; doi:10.1155/2021/8844261)
Supplement: Supplementary 3 — Table S2: prediction results for hsa-miR-4443. [file 8844261.f3.docx]

Table S2: Prediction results for hsa-miR-4443

| TargetScan prediction result | miRWalk prediction resul | miRDB prediction result |
| --- | --- | --- |
| FAM83F | ZMAT2 | KDM2A |
| HIF3A | PCDHA1 | AGPAT4 |
| SHISA6 | PCDHA2 | KRTAP4-12 |
| KRTAP4-12 | PCDHA4 | PTPRJ |
| APOBEC3C | PCDHA5 | PIK3C2B |
| IL1RN | PCDHA6 | RGS7 |
| TIMP2 | PCDHA7 | EIF4G3 |
| MOB3A | PCDHA8 | MOGAT3 |
| RP11-520P18.5 | PCDHA9 | EBF3 |
| C20orf26 | PCDHA10 | ST3GAL1 |
| B3GALT5 | PCDHA11 | SYNJ1 |
| CD209 | PCDHA12 | LARP4B |
| LMX1B | PCDHA13 | CAMK1D |
| TMEM95 | PCDHAC1 | SUMF2 |
| LZTR1 | PCDHAC2 | SCAI |
| PRSS33 | PCDHB2 | GGCX |
| PRRG3 | PCDHB3 | CD209 |
| AC016559.1 | PCDHB4 | CACNA2D4 |
| TPRXL | PCDHB5 | AFF3 |
| CLEC4M | PCDHB6 | MITF |
| F2RL2 | PCDHB7 | S100B |
| PFKFB3 | PCDHB8 | ADARB2 |
| CTA-299D3.8 | PCDHB16 | PDE1B |
| TMEM75 | PCDHB9 | MOB3A |
| CD274 | PCDHB10 | TNS1 |
| IMMP2L | PCDHB11 | LZTR1 |
| H6PD | PCDHB12 | FUBP3 |
| PTPN7 | PCDHB13 | FAM168B |
| RP11-276H1.3 | PCDHB14 | IFT80 |
| DNAH17-AS1 | SLC25A2 | C1orf74 |
| ISCA2 | TAF7 | GALK1 |
| AGPAT4 | PCDHGA1 | NBPF1 |
| RPP25 | PCDHGA2 | PDS5A |
| TMEM154 | PCDHGA3 | RFESD |
| RP11-497E19.2 | PCDHGB1 | NCOA2 |
| ACBD7 | PCDHGA4 | LIX1L |
| FLJ00388 | PCDHGB2 | GFRAL |
| COX6B2 | PCDHGA5 | PLCL1 |
| TEX35 | PCDHGB3 | YDJC |
| GBA2 | PCDHGA6 | HEPACAM |
| SLC30A2 | PCDHGA7 | CLEC4M |
| CXXC11 | PCDHGB4 | SOGA1 |
| MOGAT3 | PCDHGA8 | DGKB |
| ZNF488 | PCDHGB5 | WIPF3 |
| NFIC | PCDHGA9 | SPAG17 |
| PRY | PCDHGB6 | CAMK1G |
| LYPLA2 | PCDHGA10 | TMEM170A |
| GINM1 | PCDHGB7 | TRAF4 |
| NCOA1 | PCDHGA11 | WLS |
| PRY2 | PCDHGA12 | DLEU7 |
| MOB3C | PCDHGC3 | TIMP2 |

| METTL7B | PCDHGC4 | EFHC1 |
| --- | --- | --- |
| FLJ00104 | PCDHGC5 | KCNS1 |
| ATP6V0E1 | DIAPH1 | ESYT1 |
| MITF | HDAC3 | TRPC3 |
| AC104667.3 | RELL2 | UNC80 |
| AL049747.1 | FCHSD1 | HOXA9 |
| NR6A1 | ARAP3 | AGT |
| KCNIP3 | PCDH1 | FER1L6 |
| SATL1 | KIAA0141 | KLHL29 |
| NUPR1 | PCDH12 | TSC22D1 |
| PRRT1 | RNF14 | TCF21 |
| AFF3 | GNPDA1 | NMT2 |
| LRRC3 | NDFIP1 | NCOA1 |
| MCAM | FGF1 | XKR4 |
| CITED4 | ARHGAP26 | MUL1 |
| AC004899.1 | NR3C1 | SCN8A |
| RIPK3 | HMHB1 | PLEC |
| EBF3 | KCTD16 | ETNK2 |
| KLLN | PRELID2 | CUL2 |
| C3orf20 | GRXCR2 | NRIP3 |
| VSTM5 | SH3RF2 | TSPAN6 |
| TMEM100 | PLAC8L1 | CAPZB |
| CCDC38 | LARS | EXOG |
| IFI6 | POU4F3 | USP51 |
| CDK2AP1 | TCERG1 | NR6A1 |
| FBXL18 | GPR151 | ERLIN2 |
| CACNG3 | PPP2R2B | GVQW3 |
| CCL22 | STK32A | TRPC5 |
| KRTAP4-4 | DPYSL3 | DIPK2B |
| THY1 | JAKMIP2 | MPI |
| RAB15 | SPINK1 | ALX3 |
| RBM48 | C5orf46 | HIP1 |
| EIF2B3 | SPINK5 | AR |
| SIRPB2 | SPINK14 | TMEM100 |
| CD300LF | SPINK7 | WDHD1 |
| NOVA2 | SPINK9 | KLF6 |
| AGFG2 | FBXO38 | HIF3A |
| FER1L6 | HTR4 | SYT7 |
| NEU3 | ADRB2 | NCOA4 |
| HAVCR1 | SH3TC2 | CD274 |
| SHISA7 | ABLIM3 | RALGAPA2 |
| IRF3 | AFAP1L1 | RELCH |
| KRTAP4-2 | GRPEL2 | SAMD12 |
| C1orf74 | PCYOX1L | DBR1 |
| PVRIG | CSNK1A1 | ALX4 |
| GPX3 | ARHGEF37 | A1CF |
| TRAPPC2 | PPARGC1B | RUSC2 |
| LDB1 | PDE6A | ACPP |
| CSF3 | SLC26A2 | KRT6C |
| RP11-451M19.3 | TIGD6 | RASSF4 |
| SLA2 | HMGXB3 | B3GALT1 |
| KIAA1257 | CSF1R | PRSS22 |
| TMEM214 | PDGFRB | MCAM |

| CYB561D1 | SLC6A7 | KIF1B |
| --- | --- | --- |
| BET1L | CAMK2A | PLPP5 |
| CA13 | ARSI | ACVR1B |
| CLLU1 | TCOF1 | PMP22 |
| SPATA32 | CD74 | RNF4 |
| KNDC1 | NDST1 | PRY |
| RNF223 | SYNPO | SMARCA5 |
| TMEM199 | MYOZ3 | TCHH |
| SSSCA1 | DCTN4 | NTRK3 |
| ZNF469 | SMIM3 | NONO |
| KRT6C | IRGM | SNPH |
| KCTD15 | ZNF300 | FBXO42 |
| EXOSC6 | GPX3 | KRT77 |
| PGLYRP4 | TNIP1 | RAB11FIP4 |
| ITGA2B | ANXA6 | NECTIN4 |
| LIX1L | CCDC69 | KBTBD2 |
| LAPTM5 | GM2A | TMEM26 |
| C11orf44 | SLC36A3 | TACC1 |
| PCK1 | SLC36A2 | LRRC20 |
| SPC24 | SLC36A1 | GJB2 |
| BCKDK | FAT2 | CNTNAP2 |
| PRR3 | SPARC | U2SURP |
| TAPBP | ATOX1 | NTRK2 |
| SPP2 | GLRA1 | PTPN7 |
| FAM86A | NMUR2 | F2RL2 |
| GIPC1 | GRIA1 | TIMM10B |
| MUL1 | FAM114A2 | MOB3C |
| DUSP16 | MFAP3 | LRP6 |
| CD74 | GALNT10 | ST14 |
| TNNC1 | SAP30L | CLMN |
| CYP8B1 | LARP1 | ZNF597 |
| KCNK12 | FAXDC2 | SIRPB1 |
| ATG12 | CNOT8 | PRLR |
| OR11A1 | GEMIN5 | STX1A |
| FGF10 | MRPL22 | PRY2 |
| PRSS22 | KIF4B | PAX5 |
| CSDC2 | SGCD | TMEM95 |
| YDJC | PPP1R2B | TMEM199 |
| CXorf36 | TIMD4 | PPP1R13B |
| MBLAC1 | HAVCR1 | SMO |
| FAM106A | HAVCR2 | DLL4 |
| PLA2G5 | MED7 | C2orf16 |
| DPF2 | FAM71B | ARHGAP31 |
| PATE4 | ITK | PLXDC1 |
| LRRC25 | CYFIP2 | MLLT1 |
| LETM1 | NIPAL4 | NOVA2 |
| CNTD2 | ADAM19 | FKBP1A |
| M1AP | SOX30 | LAPTM5 |
| SERTM1 | THG1L | GCLM |
| EMP2 | LSM11 | KCNC4 |
| HLA-DPB1 | CLINT1 | NBPF3 |
| LHPP | EBF1 | YBX2 |
| SIX5 | RNF145 | FAM120A |

| EXOSC2 | IL12B | ATP6V0E1 |
| --- | --- | --- |
| PRDX2 | ADRA1B | ILF3 |
| SPRN | TTC1 | CHST2 |
| PBOV1 | PWWP2A | MTM1 |
| TESK1 | FABP6 | CREB5 |
| ZNF317 | CCNJL | RERG |
| CMA1 | ZBED8 | NBPF12 |
| LDHAL6A | SLU7 | SETD5 |
| FKBP1A | ATP10B | NBPF15 |
| ADIPOQ | GABRB2 | NBPF9 |
| PCSK7 | GABRA1 | NBPF14 |
| C21orf49 | B4GALNT3 | ERBB4 |
| CHCHD5 | NINJ2 | AKT1 |
| SPAG17 | WNK1 | NBPF20 |
| DNAI2 | RAD52 | NBPF8 |
| MFAP3L | ERC1 | RASSF2 |
| PRPS2 | WNT5B | AMER2 |
| C10orf76 | ADIPOR2 | ITPRID2 |
| TMUB1 | CACNA2D4 | BTN2A2 |
| H2AFY2 | LRTM2 | DGKA |
| GLT1D1 | DCP1B | RARB |
| HABP2 | CACNA1C | FAS |
| SLC25A35 | FKBP4 | IDI2 |
| PCGF3 | ITFG2 | NT5E |
| RGSL1 | NRIP2 | THBS1 |
| SAMD12 | FOXM1 | NBPF11 |
| AC103801.2 | RHNO1 | PRRG3 |
| KLF13 | TULP3 | ZNF540 |
| TMEM170A | TEAD4 | ZNF488 |
| AC105020.1 | TSPAN9 | MRPL42 |
| SLC30A8 | PRMT8 | SLC25A35 |
| CTD-2267D19.3 | CRACR2A | PURB |
| VANGL1 | PARP11 | PAX3 |
| TFRC | CCND2 | RAB12 |
| DDA1 | TIGAR | CUX2 |
| CAMK1G | FGF23 | GUCY1A2 |
| SLC25A36 | FGF6 | DAG1 |
| STX1A | DYRK4 | COL23A1 |
| FAS | AKAP3 | TBCEL |
| PML | GALNT8 | TESMIN |
| LRRC20 | KCNA6 | ZBTB20 |
| CREB5 | KCNA1 | MFAP3L |
| FAM120C | ANO2 | ATRX |
| SOCS3 | VWF | SIRPB2 |
| NOS1 | CD9 | METTL8 |
| KDM2A | PLEKHG6 | GPX3 |
| CYP2C19 | TNFRSF1A | ZNF251 |
| ETNK2 | SCNN1A | H6PD |
| C19orf53 | CD27 | SMLR1 |
| RARB | VAMP1 | QRICH1 |
| HSPB7 | NCAPD2 | TMEM87A |
| TINF2 | GAPDH | USP27X |
| NOG | IFFO1 | SATL1 |

| NPPC | NOP2 | RHD |
| --- | --- | --- |
| NUCB1 | CHD4 | TGM4 |
| CUX2 | LPAR5 | USB1 |
| SUMF2 | ACRBP | NOD1 |
| MPL | ING4 | PBOV1 |
| CLPSL1 | PIANP | PCSK5 |
| NECAB3 | MLF2 | ITGA2B |
| C22orf46 | LAG3 | DDX17 |
| EXOG | CD4 | PRRT1 |
| PLA2G2D | P3H3 | SYDE1 |
| BCL11A | GNB3 | SPC24 |
| LYZL6 | CDCA3 | MAP3K8 |
| USP2 | SPSB2 | RIPK3 |
| ZNF418 | LRRC23 | LYVE1 |
| IDI2 | ENO2 | PBX1 |
| GFRAL | ATN1 | TFRC |
| FAM101B | PTPN6 | TRIM13 |
| ATXN2 | PHB2 | SLTM |
| CASP16 | LPCAT3 | ANGEL1 |
| AC104841.2 | C1S | CUX1 |
| RAB38 | C1R | LOC101928841 |
| AC006486.1 | C1RL | ZNF320 |
| ZDHHC16 | RBP5 | ZNF814 |
| AP000889.3 | CLSTN3 | POU2F2 |
| KCNIP1 | PEX5 | ZNF329 |
| ATP8B3 | ACSM4 | ZC3H12A |
| CLDN18 | CD163L1 | PAX7 |
| IDO2 | CD163 | TYW5 |
| ARL6 | DPPA3 | PCK1 |
| KRT6A | CLEC4C | RNF213 |
| ZNF365 | NANOGNB | CNTD1 |
| ST14 | NANOG | DZIP3 |
| ZNF416 | SLC2A14 | NPPC |
| TTC34 | SLC2A3 | TRIM66 |
| SYT15 | FOXJ2 | CDH24 |
| TRAF4 | C3AR1 | VCAN |
| ZNF460 | NECAP1 | ANGPT4 |
| SH3RF2 | ZNF705A | COX4I1 |
| ALX3 | FAM90A1 | STC1 |
| KCNS1 | CLEC4E | KAT7 |
| RASSF4 | AICDA | EEF2KMT |
| PYDC1 | RIMKLB | EDIL3 |
| STX4 | A2ML1 | COX15 |
| MTHFR | PHC1 | SCGB2B2 |
| CHRNB2 | M6PR | SLC27A4 |
| RP11-422N16.3 | KLRG1 | DHCR24 |
| RPL23A | A2M | CHD6 |
| GRAPL | PZP | STARD4 |
| TAF11 | KLRB1 | SLC30A2 |
| C5orf45 | CLEC2D | BTBD7 |
| MORN4 | CLECL1 | TPRA1 |
| MFAP4 | CLEC2A | EEF1AKMT3 |
| CDK12 | CLEC1B | RUNDC3B |

| RASSF2 | CLEC12B | KCTD15 |
| --- | --- | --- |
| AC110619.2 | CLEC9A | CDK12 |
| TMEM26 | CLEC1A | HLA-DPB1 |
| RABEP2 | CLEC7A | PYROXD2 |
| FTMT | OLR1 | SYT1 |
| CLTCL1 | TMEM52B | FECH |
| AKT2 | KLRD1 | BIN3 |
| NFASC | KLRK1 | PATZ1 |
| ATP6V0D2 | KLRC4 | ABCC12 |
| GJB2 | EIF2S3B | ERC1 |
| PURB | MAGOHB | DGKK |
| KIAA1755 | STYK1 | TAF11 |
| RUNDC3B | YBX3 | GLIPR1 |
| KLHL30 | TAS2R10 | FBXW8 |
| RFESD | PRH1 | IGFBP5 |
| LTA | PRB3 | NMD3 |
| NSG2 | PRB4 | SHISA6 |
| KRTAP17-1 | PRB2 | BAGE2 |
| PYROXD2 | ETV6 | ARL17A |
| C2orf16 | BCL2L14 | CFLAR |
| SLC41A1 | LRP6 | BAK1 |
| FAM168B | MANSC1 | TEX48 |
| HEPACAM | BORCS5 | EXOC6B |
| NRIP3 | DUSP16 | WNT2B |
| PDE1B | CREBL2 | PMP2 |
| RP4-758J18.2 | APOLD1 | ADAMTS17 |
| ZNF346 | GPRC5A | PCNT |
| COX4I1 | GPRC5D | ZNF765 |
| CKMT1A | FAM234B | RNF169 |
| TBX6 | GSG1 | LRRC8C |
| GCLM | GRIN2B | MFN1 |
| TACR3 | ATF7IP | GRM4 |
| KCTD2 | PLBD1 | SLC5A5 |
| PAX7 | GUCY2C | CDK2AP1 |
| POLR2D | H2AFJ | PRKAA2 |
| FUBP3 | WBP11 | DVL3 |
| ALX4 | SMCO3 | PSMD8 |
| EDDM3B | ART4 | DZIP1 |
| NDUFAF2 | ERP27 | PRR3 |
| SASH3 | ARHGDIB | HYAL4 |
| MAML3 | RERG | ACBD7 |
| DGKA | PTPRO | AK4 |
| CIB2 | EPS8 | CRELD1 |
| ADAM15 | DERA | PRDX2 |
| MYLK3 | MGST1 | FOXP4 |
| CKMT1B | LMO3 | ANP32E |
| CASC4 | SDCBP | FOSL1 |
| C16orf98 | NSMAF | PGLYRP4 |
| RP11-467N20.5 | CA8 | JUP |
| PITX2 | RAB2A | MINDY4 |
| RP11-111M22.2 | ZSCAN2 | INSYN1 |
| REL | WDR73 | MAST3 |
| MTMR11 | SEC11A | KRTAP4-4 |

| DHCR24 | ZNF592 | COX18 |
| --- | --- | --- |
| ZNF134 | ALPK3 | RAB2B |
| STK36 | SLC28A1 | N4BP3 |
| SMO | PDE8A | GLI3 |
| TNS1 | AKAP13 | CDH5 |
| AC092850.1 | KLHL25 | PIP5K1A |
| KCNQ5 | AGBL1 | H2AFY2 |
| C15orf52 | NTRK3 | MTA2 |
| RAB36 | MRPS11 | MAPKBP1 |
| DNAJC24 | DET1 | DPP6 |
| CNGA2 | AEN | GAS7 |
| SPN | ISG20 | MCCC2 |
| TXNDC2 | ACAN | CLTCL1 |
| ANXA9 | HAPLN3 | SLC6A11 |
| COX18 | MFGE8 | ORAI2 |
| PPARA | ABHD2 | KIF1A |
| SCAMP4 | RLBP1 | PRKCA |
| NCOA4 | FANCI | NECAB3 |
| HRK | POLG | PSG1 |
| CLCN7 | RHCG | FICD |
| NHLRC4 | TICRR | PSG8 |
| TNR | PLIN1 | LYZL6 |
| CLEC4G | PEX11A | CTTNBP2NL |
| ZNF398 | WDR93 | VNN1 |
| FAM171B | MESP1 | SLC43A2 |
| SPOCD1 | MESP2 | PLXNA4 |
| GFI1 | ANPEP | PDE1C |
| PIP5K1C | C15orf38-AP3S2 | ZNF185 |
| ST8SIA2 | AP3S2 | METTL7B |
| KIAA1644 | ARPIN | SALL2 |
| ZG16 | ZNF710 | LTA |
| C6orf25 | IDH2 | STK38 |
| DLL4 | SEMA4B | SLC41A1 |
| CNR2 | GDPGP1 | WDR1 |
| FSTL4 | ZNF774 | XPO5 |
| C21orf119 | IQGAP1 | RANGAP1 |
| ATG9B | CRTC3 | PIP5K1C |
| SLC7A5 | BLM | AKR1C2 |
| MTM1 | FURIN | ECE1 |
| ZNF33B | FES | C3orf20 |
| ELN | MAN2A2 | NUCB1 |
| CPNE6 | UNC45A | SLC46A3 |
| CDK2 | HDDC3 | NDUFA4 |
| HECTD3 | PRC1 | RGSL1 |
| ZNF329 | VPS33B | LRRC15 |
| TOMM40 | SV2B | DDX6 |
| ONECUT3 | SLCO3A1 | C22orf46 |
| CTC-360G5.1 | ST8SIA2 | HHIPL1 |
| ACPP | FAM174B | EXOSC6 |
| COL23A1 | CHD2 | USP2 |
| TGFA | RGMA | PSG4 |
| DZIP1 | MCTP2 | GLIS3 |
| MAP3K8 | NR2F2 | KLHL20 |

| SERPINA6 | ARRDC4 | PSG7 |
| --- | --- | --- |
| TSNARE1 | FAM169B | STRADB |
| SLC12A3 | PGPEP1L | SLC35C1 |
| DBF4B | SYNM | UNC5B |
| PMP22 | TTC23 | KRTAP4-2 |
| CDH24 | LRRC28 | HECTD3 |
| EXOSC5 | MEF2A | ZNF737 |
| CHID1 | LYSMD4 | SLC13A5 |
| HYI | ADAMTS17 | LDHAL6A |
| AP005482.1 | CERS3 | PRKCE |
| ZNF157 | LINS1 | C1orf198 |
| FKBP1C | ASB7 | NIT2 |
| RCN3 | ALDH1A3 | CASTOR2 |
| NBPF3 | LRRK1 | SLC16A14 |
| FAM43B | CHSY1 | SIX5 |
| DEFB132 | SELENOS | RBM18 |
| RP11-178C3.1 | PCSK6 | OR11A1 |
| LY6K | TM2D3 | IMMP2L |
| USP44 | OR4F6 | TAPBP |
| TM4SF19 | LOC102723502 | ST8SIA2 |
| AC068987.1 | RHBDF1 | C1QTNF9B |
| KIF1B | MPG | FRYL |
| ZC3H12A | HBZ | PML |
| C9orf66 | HBA2 | KNDC1 |
| EPN2 | HBA1 | CCDC34 |
| FAM203B | HBQ1 | IL12RB2 |
| PAX2 | LUC7L | PIK3CD |
| DBR1 | FAM234A | MYBPHL |
| TRPC3 | RGS11 | SLX4 |
| KCNC4 | PDIA2 | LTBP2 |
| P2RX1 | MRPL28 | VANGL2 |
| C11orf34 | TMEM8A | CPNE6 |
| WDR55 | NME4 | RPL23A |
| NCS1 | DECR2 | C5orf63 |
| NDUFA2 | RAB11FIP3 | KCTD2 |
| PIF1 | CAPN15 | FAM129C |
| DOLPP1 | PRR35 | SNX21 |
| MIF4GD | RAB40C | COL4A3 |
| AL590822.1 | WFIKKN1 | TRIM41 |
| GPR37L1 | METTL26 | TMEM63C |
| ZNF557 | MCRIP2 | CKMT1A |
| VNN1 | WDR90 | LETM1 |
| MIEN1 | RHOT2 | PTPN3 |
| SH2D3C | RHBDL1 | IL1RN |
| TSPAN13 | STUB1 | EXOSC5 |
| TMPRSS4 | JMJD8 | BEGAIN |
| AC091801.1 | WDR24 | CLCN2 |
| PIGO | FBXL16 | XKR5 |
| CBL | NARFL | PCDH7 |
| MS4A4A | MSLN | CAP2 |
| STK35 | RPUSD1 | SLC6A15 |
| TBCA | CHTF18 | SREBF1 |
| TRPC5 | SOX8 | PAX2 |

| TMED2 | SSTR5 | PARD3B |
| --- | --- | --- |
| GNAT1 | C1QTNF8 | SLC25A48 |
| AC013269.5 | CACNA1H | SCAMP4 |
| PPP1R13B | TPSG1 | SLAMF6 |
| NEUROG3 | TPSB2 | ZG16 |
| ZNF90 | TPSAB1 | SLC7A5 |
| KIF26B | TPSD1 | BCL11A |
| PARD6G | UBE2I | NKAIN1 |
| FAM180B | BAIAP3 | ARMH3 |
| CERS5 | TSR3 | RMI1 |
| BAK1 | GNPTG | SYNE3 |
| KIAA0247 | UNKL | GIT2 |
| MRGPRF | CCDC154 | CREB1 |
| DNAJC5G | CLCN7 | LDB1 |
| CCDC115 | PTX4 | SEC14L2 |
| SMPD3 | TELO2 | REL |
| S100A7 | IFT140 | MYO1E |
| MAPKBP1 | TMEM204 | ATP6V0D2 |
| C10orf129 | CRAMP1 | PRKAG1 |
| KPRP | JPT2 | POTEM |
| GATSL3 | MAPK8IP3 | TNFAIP8L3 |
| SH2D4A | NME3 | COX10 |
| PPP1R3G | MRPS34 | TRAF1 |
| THBS1 | NUBP2 | NFASC |
| QRICH1 | IGFALS | CENPP |
| PBX1 | FAHD1 | ZNF829 |
| KRT8 | MSRB1 | NOG |
| WDHD1 | RPS2 | CLDN18 |
| CAMK2A | TBL3 | GAB2 |
| RARRES3 | NOXO1 | FTMT |
| C2orf66 | GFER | GALNT10 |
| WWTR1 | SYNGR3 | ZER1 |
| PTPRQ | ZNF598 | MAPK15 |
| ENG | SLC9A3R2 | ZIC5 |
| THTPA | NTHL1 | ZNF557 |
| ATP2A2 | TSC2 | PEG10 |
| AP4S1 | PKD1 | STOML2 |
| PACSIN1 | RAB26 | CTIF |
| RGS7 | TRAF7 | EHD4 |
| GINS4 | MLST8 | SLC46A1 |
| TNFRSF1B | PGP | CACNB1 |
| TMEM119 | E4F1 | TRABD2B |
| COX10 | DNASE1L2 | BCAM |
| FA2H | ECI1 | SOX13 |
| USP51 | RNPS1 | BMP8A |
| CACNA2D4 | CCNF | MTUS1 |
| NDUFS7 | TEDC2 | RANBP17 |
| TSPAN32 | NTN3 | ATXN7L3 |
| TCHH | TBC1D24 | CKMT1B |
| PDE4B | ATP6V0C | RABEP2 |
| SHISA9 | AMDHD2 | CHRDL1 |
| FOXP4 | CEMP1 | HABP2 |
| TNS4 | PDPK1 | KDM4E |

| AC012215.1 | KCTD5 | ENPP6 |
| --- | --- | --- |
| GTPBP1 | PRSS27 | ATP2A2 |
| POLR2H | SRRM2 | GRIP2 |
| FCRLA | PRSS33 | SLC30A8 |
| PEG10 | PRSS41 | SMPD3 |
| ZNF320 | PRSS21 | HSPB7 |
| SOD3 | FLYWCH2 | FAM43B |
| RP1-170O19.20 | FLYWCH1 | CHRNB2 |
| SNX21 | PAQR4 | CALCR |
| LRFN1 | PPIL3 | RPL27A |
| AC010536.1 | NIF3L1 | SERTM1 |
| DCDC2B | FAM126B | MLXIP |
| DGCR6 | NDUFB3 | NKD1 |
| GPR137C | CFLAR | SERPINA6 |
| VANGL2 | CASP10 | XKRX |
| ACRV1 | CASP8 | CALN1 |
| STON2 | TRAK2 | CLEC4G |
| ATRNL1 | STRADB | MFAP4 |
| RAB39A | C2CD6 | PLET1 |
| KLHL20 | TMEM237 | TNFSF8 |
| LILRA1 | MPP4 | SPRED2 |
| STRADB | ALS2 | GLP1R |
| KIAA1671 | CDK15 | MEIS3 |
| NUDT4 | FZD7 | SCP2 |
| FBXL12 | KIAA2012 | ST3GAL2 |
| ZNF251 | BMPR2 | SOCS3 |
| RCOR3 | FAM117B | ERVH48-1 |
| IER3 | ICA1L | GRAPL |
| POLR3D | WDR12 | ANAPC16 |
| BAX | CARF | TTC9 |
| MYADM | NBEAL1 | TNNC1 |
| FAM203A | CYP20A1 | CSNK1G3 |
| HYAL4 | RAPH1 | SHISA9 |
| YBX2 | CD28 | ZKSCAN4 |
| TPRA1 | ICOS | SASH3 |
| FANCD2 | PARD3B | MEP1A |
| CALN1 | NRP2 | AMOT |
| C7orf76 | INO80D | NDEL1 |
| EIF4EBP1 | NDUFS1 | KAZN |
| PIK3CD | EEF1B2 | EXOSC2 |
| EDIL3 | GPR1 | LDLR |
| CD300LB | ZDBF2 | GJA3 |
| EPHA1 | ADAM23 | SARM1 |
| RDH13 | FAM237A | LEPROTL1 |
| FABP1 | DYTN | RAB38 |
| ZNF337 | FASTKD2 | FMNL3 |
| FAM180A | CPO | ZBTB39 |
| IKBKG | KLF7 | KCNIP3 |
| TACC1 | CREB1 | FAM120C |
| EVX1 | METTL21A | SCN1B |
| FHL3 | FZD5 | THY1 |
| APOBEC3F | PLEKHM3 | KCNIP1 |
| PARP11 | CRYGD | TULP4 |

| SYNJ1 | CRYGA |  | ZFHX4 |
| --- | --- | --- | --- |
| MOCOS | C2orf80 |  | ZNF365 |
| LARP4B | PIKFYVE |  | DMTN |
| APOL4 | PTH2R |  | CYP8B1 |
| METTL21B | MAP2 |  | CYP2C19 |
| RNF4 | UNC80 |  | CAPNS1 |
| ZNF597 | KANSL1L |  | URGCP-MRPS24 |
| C1orf111 | ACADL |  | GABRA3 |
| PIANP | MYL1 |  | L3MBTL2 |
| ZNF562 | LANCL1 |  | SGSM1 |
| AASDHPPT | CPS1 |  | RBMS2 |
| CPNE7 | ERBB4 |  | KIAA1671 |
| PIRT | IKZF2 |  | TRAF7 |
| OR12D3 | VWC2L |  | RFLNB |
| NFE2 | BARD1 |  | DGCR8 |
| FAM86C1 | ABCA12 |  | KLF13 |
| NDEL1 | FN1 |  | ALDH3B2 |
| PRODH | MREG |  | ENY2 |
| YPEL4 | PECR |  | CA13 |
| ZNF185 | TMEM169 |  | ACAN |
| ACTL7B | XRCC5 |  | EIF2B3 |
| PHPT1 |  | 4-Mar | MRPL30 |
| GALNT10 | SMARCAL1 |  | NDUFC2-KCTD14 |
| ZNF880 | IGFBP5 |  | ITSN2 |
| ATXN7L3 | TNS1 |  | LMX1B |
| CAP2 | RUFY4 |  | SPIB |
| ILK | CXCR2 |  | MYH14 |
| PIP5K1A | CXCR1 |  | C1QTNF9 |
| MTIF2 | ARPC2 |  | TANC1 |
| PPP1R3D | GPBAR1 |  | KRT6A |
| CMTM7 | AAMP |  | SH3TC2 |
| EHD4 | PNKD |  | ATG14 |
| YWHAE | TMBIM1 |  | NCAPH |
| SNX3 | CATIP |  | MGST2 |
| PCDHB11 | SLC11A1 |  | COX6B2 |
| LETMD1 | CTDSP1 |  | MFHAS1 |
| ZMYND10 | VIL1 |  | ING4 |
| KIF3C | USP37 |  | TESPA1 |
| IFRG15 | PLCD4 |  | ARRDC3 |
| LRRC38 | ZNF142 |  | HHAT |
| CD96 | BCS1L |  | GIPC1 |
| CACNB1 | STK36 |  | PRPS2 |
| ZNF324B | TTLL4 |  | LARP1 |
| BAALC | CYP27A1 |  | SLC12A3 |
| PRUNE2 | PRKAG3 |  | IDO2 |
| NBPF14 | WNT10A |  | RNASEH2C |
| PRKAA2 | CDK5R2 |  | FXN |
| TMEM229B | FEV |  | RSBN1 |
| SLTM | CRYBA2 |  | TEAD3 |
| ZNF852 | CFAP65 |  | PVRIG |
| BPIFB4 | IHH |  | DLAT |
| ADH5 | NHEJ1 |  | C2orf68 |
| XRCC3 | SLC23A3 |  | DGKE |

| TP53RK | CNPPD1 | TMC8 |
| --- | --- | --- |
| RSL1D1 | RETREG2 | NOS1 |
| CCDC120 | ABCB6 | ONECUT2 |
| RAET1L | ATG9A | PACSIN1 |
| CERS1 | ANKZF1 | KIRREL2 |
| NKAIN1 | GLB1L | SERTAD2 |
| METTL6 | STK16 | TBC1D12 |
| PTOV1 | TUBA4A | COL5A1 |
| WDR1 | DNAJB2 | VDR |
| ACACB | PTPRN | MED20 |
| DTL | DNPEP | KIAA0930 |
| SLAMF8 | DES | DGKQ |
| CCDC121 | LOC100996693 | OPA1 |
| TMEM86B | CHPF | RASA2 |
| RP11-204N11.1 | TMEM198 | STOX2 |
| NFAM1 | OBSL1 | EIF2AK3 |
| TTC38 | INHA | C2orf66 |
| AC138655.1 | STK11IP | ADAM15 |
| APBA3 | EPHA4 | IRAK1 |
| MCCC2 | PAX3 | CDV3 |
| CUL2 | SGPP2 | ADIPOQ |
| SLC22A23 | MOGAT1 | KLHL3 |
| CCND3 | ACSL3 | PAPPA |
| LTBP2 | KCNE4 | SMARCC2 |
| LEMD2 | SCG2 | GPR171 |
| CALML5 | AP1S3 | ENTPD1 |
| PIPOX | WDFY1 | EFCAB14 |
| CACNG1 | MRPL44 | PTPN14 |
| UBL7 | SERPINE2 | UBE3B |
| CNR1 | FAM124B | PGR |
| ENPP6 | CUL3 | ZNF714 |
| LDLRAP1 | DOCK10 | TNFSF4 |
| EDN2 | NYAP2 | ALDOB |
| NMD3 | IRS1 | SBNO1 |
| RC3H1 | RHBDD1 | ARMC5 |
| C3orf70 | COL4A4 | ELP1 |
| TNFSF8 | COL4A3 | GLT1D1 |
| NOD1 | TM4SF20 | M1AP |
| HR | AGFG1 | C11orf44 |
| CCDC64 | GABRG2 | DCUN1D1 |
| HOXC12 | CCNG1 | PCDHB11 |
| HOXA9 | NUDCD2 | ZCWPW1 |
| LRRD1 | MAT2B | KIAA0895 |
| MORN5 | TENM2 | FKBP5 |
| ASPA | WWC1 | GFOD1 |
| EDDM3A | PANK3 | ACER3 |
| SLC35C1 | SLIT3 | FLJ45513 |
| TEAD3 | SPDL1 | TTC38 |
| EFCC1 | DOCK2 | DIAPH1 |
| LIMD1 | FAM196B | ZNF346 |
| C17orf82 | FOXI1 | TMEM25 |
| ZNF517 | LCP2 | SAMD9 |
| KAT7 | KCNIP1 | UMPS |

| COLEC10 | KCNMB1 | PLA2G2D |
| --- | --- | --- |
| CEACAM16 | GABRP | GDF11 |
| VGLL2 | RANBP17 | ATG12 |
| MYBPHL | TLX3 | STOM |
| FAM188B | NPM1 | UTP25 |
| SMCP | FGF18 | FAM171B |
| TBXA2R | FBXW11 | BHLHB9 |
| CAPNS1 | SH3PXD2B | MYADM |
| GLTSCR1 | NEURL1B | RHOBTB2 |
| PCYT1B | DUSP1 | ABL2 |
| GDNF | ERGIC1 | TBR1 |
| S100A7A | RPL26L1 | EVC |
| MVB12B | CREBRF | BET1L |
| NDUFC2-KCTD14 | NKX2-5 | ARHGAP32 |
| RAB11FIP5 | STC2 | SEC16B |
| C11orf21 | BOD1 | NUDT4 |
| TNFSF10 | CPEB4 | NDUFA2 |
| PRKAG1 | NSG2 | TNFSF9 |
| MAVS | MSX2 | THRB |
| MUM1L1 | DRD1 | CNTNAP1 |
| PRSS38 | SFXN1 | TMPRSS4 |
| SP7 | HRH2 | DOT1L |
| ANP32E | CPLX2 | SCN4A |
| MB | SIMC1 | GNAO1 |
| ABHD14B | KIAA1191 | SHISAL1 |
| SPATA12 | ARL10 | ZNF776 |
| RP11-382J12.1 | NOP16 | SHC3 |
| TECTA | HIGD2A | NHLRC4 |
| SYDE1 | CLTB | STPG1 |
| C5orf60 | FAF2 | ZDHHC16 |
| NBPF15 | RNF44 | MAML3 |
| GVQW1 | CDHR2 | LDLRAD3 |
| TMEM207 | GPRIN1 | EMP2 |
| C1orf158 | EIF4E1B | SH3RF2 |
| NBPF16 | UNC5A | SZT2 |
| PAK7 | HK3 | S100A7A |
| CCDC103 | UIMC1 | ANKRD42 |
| ZNF696 | ZNF346 | C9orf66 |
| HIP1 | FGFR4 | ZNF619 |
| VAMP7 | NSD1 | EIF4EBP1 |
| FBXL8 | PRELID1 | CD300LF |
| CTDSPL | MXD3 | UCK2 |
| TFCP2L1 | LMAN2 | CCDC9B |
| SAMD10 | SLC34A1 | PCSK7 |
| ZNF664 | GRK6 | ISCA2 |
| ARHGAP44 | PRR7 | ZNF90 |
| ZIK1 | DBN1 | TGFB2 |
| ESYT1 | PDLIM7 | TRA2B |
| SLC26A1 | DOK3 | DND1 |
| TNFSF4 | DDX41 | CCER2 |
| DNAJC9 | FAM193B | BNC2 |
| USB1 | TMED9 | PLOD1 |
| SLC5A5 | FAM153A | HLA-E |

| MTMR7 | PROP1 | SLC25A36 |
| --- | --- | --- |
| KRT77 | N4BP3 | FHL3 |
| CDH5 | GMCL2 | LOC102724265 |
| CTB-186H2.3 | PHYKPL | ARL4C |
| PCBP4 | COL23A1 | DIRAS2 |
| KLF7 | LOC102724657 | TACR3 |
| PABPC5 | ZNF354A | SLMAP |
| KBTBD12 | ZFP2 | CARM1 |
| ADCK1 | ZNF454 | PIANP |
| KNTC1 | GRM6 | THTPA |
| PI4K2A | ADAMTS2 | SLC45A4 |
| SRL | RUFY1 | PMM2 |
| KSR2 | CANX | ZNF134 |
| COQ10A | MAML1 | SBK1 |
| MOGS | LTC4S | PLEKHG4B |
| GNPDA1 | MGAT4B | CDRT4 |
| ZNF782 | MRNIP | SVEP1 |
| AC026310.1 | TBC1D9B | VGLL2 |
| CPN2 | RNF130 | KPRP |
| BNIP1 | RASGEF1C | ASB16 |
| PTPRJ | MAPK9 | TMED7 |
| UGT3A2 | GFPT2 | HTR3E |
| ATP5J2 | SCGB3A1 | EIF4E2 |
| DDX17 | FLT4 | YPEL4 |
| NBPF9 | OR2Y1 | FMO2 |
| PAFAH1B2 | MGAT1 | IQSEC3 |
| KCNE1L | ZFP62 | PCBP4 |
| LONRF3 | BTNL8 | RBBP5 |
| SHOX2 | BTNL9 | ADCK1 |
| B3GNT6 | OR2V2 | TMEM214 |
| LPAR2 | TRIM7 | FBLN1 |
| IKZF5 | TRIM41 | SEMA5A |
| ZNF497 | TRIM52 | ZNF703 |
| FAM171A2 | OR4F3 | HIPK2 |
| LYPD1 | DUSP22 | PPM1A |
| ZNF587 | IRF4 | DLGAP1 |
| HLA-E | EXOC2 | TRIM67 |
| P2RY1 | HUS1B | SEL1L |
| DLGAP2 | FOXQ1 | E2F3 |
| GPR83 | FOXF2 | PPP1R16B |
| SLC6A2 | MYLK4 | MTHFR |
| GLIPR1 | SERPINB1 | RBM20 |
| HGSNAT | SERPINB9 | FNTB |
| MBD6 | SERPINB6 | SERF2 |
| CYP4A11 | NQO2 | CLCN7 |
| TGM4 | BPHL | TMEM135 |
| PLA2G4E | TUBB2A | DEFB132 |
| SMAP2 | TUBB2B | MMP8 |
| APEX1 | PSMG4 | PLXNA2 |
| L3MBTL2 | SLC22A23 | PODN |
| KIAA0895 | PXDC1 | KPNA3 |
| PEX10 | FAM50B | NF2 |
| ZNF764 | PRPF4B | NDST1 |

| HMGN4 | ECI2 | ARHGAP30 |
| --- | --- | --- |
| EIF4E3 | CDYL | PIRT |
| CATSPERG | PPP1R3G | STK32C |
| S100A14 | LYRM4 | TRIOBP |
| UBXN4 | NRN1 | SLC9A5 |
| PAX5 | F13A1 | PDHA1 |
| ARHGAP17 | RREB1 | TVP23C-CDRT4 |
| AHI1 | SSR1 | ATP6V1C1 |
| RBM17 | CAGE1 | PHC2 |
| RGS4 | DSP | TGM2 |
| POMGNT1 | SNRNP48 | KCNB1 |
| LRTOMT | TXNDC5 | CYP20A1 |
| GPR75 | BLOC1S5 | SH3BP2 |
| TMEM204 | TFAP2A | PFKFB3 |
| DGKQ | GCNT2 | NEFL |
| C7orf65 | PAK1IP1 | GNPDA1 |
| MR1 | TMEM14C | HECTD4 |
| ANKRD42 | TMEM14B | PCIF1 |
| SCAI | MAK | GINS4 |
| COL21A1 | GCM2 | WASF2 |
| ING4 | SYCP2L | KAT6A |
| FBXW8 | BFSP2 | DNAI2 |
| NBPF10 | CDV3 | CALR |
| PMP2 | TOPBP1 | CERS1 |
| LTC4S | SLCO2A1 | CCDC121 |
| ZBTB43 | RYK | HIRIP3 |
| EDN1 | AMOTL2 | APOBEC3F |
| ZNF367 | ANAPC13 | VXN |
| UBE3B | CEP63 | MOBP |
| RERG | KY | PPME1 |
| LGALS9 | EPHB1 | HIF1AN |
| NAV1 | PPP2R3A | TLN2 |
| DDIT4L | MSL2 | SUPT16H |
| MDM4 | PCCB | MDM2 |
| SLC6A16 | STAG1 | PKNOX2 |
| CREB1 | NCK1 | C4orf3 |
| FOXE3 | IL20RB | LRRC25 |
| C1orf198 | SOX14 | TXLNG |
| SERPINF2 | CLDN18 | CBX6 |
| MFSD2B | DZIP1L | RIMS3 |
| PSD | A4GNT | SH2D3C |
| TTC1 | DBR1 | MMP11 |
| TSR2 | ARMC8 | NAA60 |
| PDE1C | NME9 | ATG16L1 |
| EPX | MRAS | TGFA |
| OSGEPL1 | ESYT3 | GATAD2A |
| TIMM10B | CEP70 | ZSCAN29 |
| ABHD17A | FAIM | APOBEC3C |
| PGAP3 | PIK3CB | CD96 |
| FBLIM1 | FOXL2NB | ZNF416 |
| RNF165 | PRR23C | MAPK8 |
| AMBRA1 | COPB2 | DCUN1D5 |
| LRRC10B | RBP1 | ZNF418 |

| P2RY8 | NMNAT3 | FAM160A1 |
| --- | --- | --- |
| CXADR | CLSTN2 | APOPT1 |
| SLC27A4 | TRIM42 | TRMT9B |
| STPG1 | SLC25A36 | ATP7B |
| CHRNA3 | PXYLP1 | GIMAP5 |
| E2F3 | RASA2 | DEPDC5 |
| PCNT | RNF7 | MTMR7 |
| RMI1 | GRK7 | RAB39A |
| DPPA3 | TFDP2 | SYT2 |
| ZNF829 | GK5 | GOLGA6L1 |
| MTA2 | XRN1 | CLEC3A |
| FAM222B | ATR | GOLGA6L6 |
| ECE1 | PLS1 | ZNF664 |
| PLIN5 | TRPC1 | MACO1 |
| DLG4 | U2SURP | TP53I11 |
| SLC46A3 | CHST2 | CNP |
| RP11-527L4.2 | SLC9A9 | GIMAP1-GIMAP5 |
| FOSL1 | C3orf58 | CMA1 |
| C7orf13 | PLOD2 | TRAPPC2 |
| RASSF7 | PLSCR4 | BNIP2 |
| C9orf96 | PLSCR2 | MYO1A |
| HDGFL1 | PLSCR1 | PLAGL2 |
| ATP6V1C2 | PLSCR5 | MTF1 |
| CDC42SE1 | ZIC4 | NABP1 |
| ZBTB39 | ZIC1 | GASK1B |
| ARHGEF15 | CPB1 | WASHC4 |
| PNOC | CPA3 | KLHL30 |
| RP11-116D17.1 | GYG1 | PPL |
| SMIM21 | HLTF | PUM1 |
| IL1R1 | CP | PTGIS |
| ZNF540 | TM4SF18 | PAIP2B |
| STXBP1 | TM4SF1 | NFIA |
| SDR9C7 | WWTR1 | FAM222A |
| SLC9A5 | COMMD2 | MON1A |
| PSORS1C2 | ANKUB1 | P2RY8 |
| LRP6 | RNF13 | LRRC59 |
| DRAXIN | TSC22D2 | ATP2B2 |
| LDLRAD3 | EIF2A | HR |
| LGALS9B | ERICH6 | CREBZF |
| GOLGA6L1 | SIAH2 | C6orf132 |
| CCL15 | CLRN1 | SPOUT1 |
| MAP7D2 | MED12L | ARL10 |
| SLC25A15 | GPR171 | BCKDK |
| PNPLA2 | P2RY14 | CTDSPL |
| NSUN7 | GPR87 | AMBRA1 |
| ZDHHC18 | P2RY13 | CCN4 |
| WDR76 | IGSF10 | P2RY1 |
| RNF169 | AADACL2 | HK1 |
| PREPL | AADAC | ZNF629 |
| FAM221B | SUCNR1 | PPARGC1B |
| GATC | MBNL1 | KRT73 |
| ONECUT1 | P2RY1 | PAQR8 |
| FKBP4 | RAP2B | ALDH7A1 |

| SRCRB4D | ARHGEF26 | PPP2R1B |
| --- | --- | --- |
| DCAKD | GPR149 | CD74 |
| TTBK1 | MME | PHF21A |
| BCAS4 | PLCH1 | RC3H1 |
| KRTAP10-1 | C3orf33 | HBP1 |
| PIK3C2B | SLC33A1 | TMOD2 |
| CTB-167G5.5 | GMPS | SSC4D |
| FAM63A | KCNAB1 | PDE4B |
| IL36RN | SSR3 | DBT |
| ERAL1 | TIPARP | C3orf70 |
| PATZ1 | LEKR1 | RXRB |
| MAP1LC3C | CCNL1 | RAP1GAP2 |
| ELMOD3 | VEPH1 | STX17 |
| LRRN4CL | PTX3 | SRL |
| GOLGA6L6 | SHOX2 | MUC13 |
| LGALS9C | RSRC1 | TUBB |
| OR6V1 | LXN | MCHR1 |
| CDCA8 | RARRES1 | PKIA |
| ZBTB8B | IQCJ | SLC38A1 |
| GDF11 | IL12A | HDAC11 |
| LARS2 | IFT80 | PCGF3 |
| GATA1 | SMC4 | UQCRQ |
| ANKRD63 | TRIM59 | ATRNL1 |
| FAM53C | KPNA4 | EPHB2 |
| C2orf15 | PPM1L | LTB4R2 |
| MRPL30 | B3GALNT1 | RORA |
| THEM6 | NMD3 | APPBP2 |
| GSC2 | SPTSSB | IMPDH1 |
| ACTR1A | OTOL1 | SLAMF8 |
| KLF6 | SI | TSNARE1 |
| PRLR | SLITRK3 | XRCC3 |
| ATP6V0A1 | BCHE | ZNF652 |
| DSG3 | ZBBX | MR1 |
| KBTBD2 | PDCD10 | NACC2 |
| ASB16 | GOLIM4 | DBF4B |
| CDKL4 | MECOM | ADIG |
| DCXR | MYNN | STON2 |
| UROC1 | LRRIQ4 | CD34 |
| TMEM151A | LRRC31 | WWOX |
| ARHGAP30 | SAMD7 | COPS2 |
| HLA-DQA1 | SEC62 | GOLGA7B |
| MYO1F | GPR160 | ALDH18A1 |
| EBI3 | PHC3 | MRPS21 |
| UBXN2B | PRKCI | EPN2 |
| PVRL4 | FAM92B | ADAMTSL5 |
| OSCAR | GSE1 | ARSA |
| MAZ | GINS2 | ETFBKMT |
| NBPF12 | EMC8 | VSTM2B |
| ARMC5 | IRF8 | CAMKK1 |
| MT-ATP8 | MTHFSD | P2RY2 |
| HCLS1 | FOXC2 | FGFBP3 |
| FMO2 | FOXL1 | M6PR |
| NCOR2 | FBXO31 | FGFR3 |

| MAPK1 | MAP1LC3B | FKBP4 |
| --- | --- | --- |
| TBCEL | ZCCHC14 | AXIN1 |
| NPR3 | JPH3 | SLC38A10 |
| KRTAP24-1 | KLHDC4 | ZNF33B |
| CYP4A22 | SLC7A5 | BCL2L13 |
| MON1A | BANP | SOGA3 |
| CD27 | ZFPM1 | KIF24 |
| WNT4 | ZC3H18 | PPARA |
| SST | CYBA | CACUL1 |
| HPN | SNAI3 | NBPF4 |
| PRPF38A | RNF166 | EDN2 |
| RXRB | CTU2 | POU3F3 |
| GRAP2 | PIEZO1 | LPO |
| CHAC1 | CDT1 | PREX2 |
| GLP1R | APRT | SH2D4A |
| TPP1 | GALNS | XIRP1 |
| ADPRHL1 | TRAPPC2L | CIAO1 |
| NBPF11 | PABPN1L | NRP1 |
| PTGIS | CBFA2T3 | SPOCK2 |
| DMTN | ACSF3 | CLIC6 |
| TRIM62 | ZNF778 | ADCY1 |
| HMGXB4 | ANKRD11 | GTPBP1 |
| SLC5A10 | SPG7 | STXBP1 |
| SYTL5 | RPL13 | MPP6 |
| SUV39H1 | CPNE7 | RAB17 |
| PAQR8 | DPEP1 | POTEF |
| DBN1 | SPATA33 | PRMT2 |
| IRAK1 | CDK10 | RBFOX2 |
| AC008132.1 | SPATA2L | MTRNR2L4 |
| NAALADL2 | VPS9D1 | DUSP22 |
| C19orf54 | ZNF276 | PCLO |
| C1orf226 | FANCA | GREB1 |
| ALPPL2 | TCF25 | GRSF1 |
| TMEM213 | MC1R | LRFN4 |
| GDAP1 | OR2M4 | MYBBP1A |
| ADAT3 | OR2M7 | UPF3A |
| MYBBP1A | OR2T4 | BBX |
| B4GALNT3 | OR2T6 | UBXN4 |
| TRIM66 | OR2T1 | KREMEN1 |
| BRSK2 | OR2T7 | ZNF436 |
| C21orf2 | OR2T2 | HSPA6 |
| LYNX1 | OR2T3 | NANOGNB |
| FXYD7 | OR2T5 | LEMD2 |
| NBPF1 | OR2T29 |  |
| SYT7 | OR2T34 |  |
| C2CD4C | OR2T10 |  |
| TMEM150A | OR2T27 |  |
| HILPDA | OR14I1 |  |
| CYLC2 | LYPD8 |  |
| AC026703.1 | ZNF672 |  |
| TMEM184A | ZNF692 |  |
| HHIPL1 | LOC102724250 |  |
| COL5A1 | FAM110C |  |

| CDV3 | SH3YL1 |  |
| --- | --- | --- |
| FAM131B | ACP1 |  |
| ERLIN2 | ALKAL2 |  |
| C12orf49 | TMEM18 |  |
| SBK1 | TPO |  |
| NKX6-3 | PXDN |  |
| RARRES2 | MYT1L |  |
| C1QTNF9B | TRAPPC12 |  |
| RRP7A | ADI1 |  |
| VSNL1 | RNASEH1 |  |
| AMOTL1 | COLEC11 |  |
| AL021546.6 | ALLC |  |
| SIX1 | CMPK2 |  |
| KCNE1 | RSAD2 |  |
| RPL3L | RNF144A |  |
| ZNF74 | KIDINS220 |  |
| HIVEP3 | MBOAT2 |  |
| PPP5D1 | ASAP2 |  |
| S100A16 | ADAM17 |  |
| ITPRIP | TAF1B |  |
| TP53I11 | GRHL1 |  |
| HOXC13 | CYS1 |  |
| FAM102B | RRM2 |  |
| SLC4A8 | HPCAL1 |  |
| CHST6 | ODC1 |  |
| MIIP | NOL10 |  |
| ZNF625 | ATP6V1C2 |  |
| ACKR4 | PDIA6 |  |
| FCGRT | KCNF1 |  |
| MAN2A2 | ROCK2 |  |
| KIAA0930 | E2F6 |  |
| CDH1 | GREB1 |  |
| DFFB | NTSR2 |  |
| POMGNT2 | LPIN1 |  |
| RASA2 | TRIB2 |  |
| MED24 | NBAS |  |
| JPH2 | DDX1 |  |
| UQCRFS1 | MYCN |  |
| ZNF33A | FAM49A |  |
| XKR4 | RAD51AP2 |  |
| RPS6KL1 | VSNL1 |  |
| ISCU | SMC6 |  |
| PRELP | GEN1 |  |
| NTRK3 | MSGN1 |  |
| CCRN4L | KCNS3 |  |
| DDX56 | NT5C1B |  |
| KRT73 | OSR1 |  |
| PNPLA1 | WDR35 |  |
| MFAP3 | SDC1 |  |
| PLCL1 | PUM2 |  |
| ACER2 | RHOB |  |
| MRPL35 | HS1BP3 |  |
| GPA33 | GDF7 |  |

| SPHK2 | LDAH |  |
| --- | --- | --- |
| FRYL | APOB |  |
| TGM2 | TDRD15 |  |
| METTL20 | KLHL29 |  |
| CD22 | ATAD2B |  |
| ZNF71 | MFSD2B |  |
| TFAP4 | WDCP |  |
| PPME1 | FKBP1B |  |
| UPK3BL | TP53I3 |  |
| UBXN6 | PFN4 |  |
| MEP1A | FAM228B |  |
| MIXL1 | ITSN2 |  |
| REEP6 | NCOA1 |  |
| DND1 | CENPO |  |
| ATP6V1G2 | ADCY3 |  |
| IL7R | DNAJC27 |  |
| TMEM168 | EFR3B |  |
| DCP2 | POMC |  |
| SLC46A1 | DNMT3A |  |
| MTRNR2L4 | DTNB |  |
| KIAA1614 | ASXL2 |  |
| RAP1GAP2 | KIF3C |  |
| ANK1 | GAREM2 |  |
| ULBP2 | HADHA |  |
| TREML2 | ADGRF3 |  |
| R3HDML | SELENOI |  |
| PLOD1 | DRC1 |  |
| ZNF737 | OTOF |  |
| TNFRSF10A | CIB4 |  |
| LHX3 | KCNK3 |  |
| CCDC24 | SLC35F6 |  |
| POLR3F | CENPA |  |
| TULP4 | DPYSL5 |  |
| DNAJB12 | MAPRE3 |  |
| MTRNR2L11 | TMEM214 |  |
| KLHL3 | AGBL5 |  |
| TMEM80 | EMILIN1 |  |
| CNP | KHK |  |
| MAPK10 | CGREF1 |  |
| ZNF512B | PREB |  |
| TAS1R1 | PRR30 |  |
| ZNF649 | SLC5A6 |  |
| OPA3 | ATRAID |  |
| ZNF619 | CAD |  |
| FLCN | SLC30A3 |  |
| TTC9 | DNAJC5G |  |
| ZNF784 | UCN |  |
| GPR171 | MPV17 |  |
| TMEM151B | GTF3C2 |  |
| C12orf56 | EIF2B4 |  |
| GLIS2 | SNX17 |  |
| ABCD4 | ZNF513 |  |
| ALPL | PPM1G |  |

| PCIF1 | NRBP1 |  |
| --- | --- | --- |
| TRIM40 | KRTCAP3 |  |
| TUBB6 | IFT172 |  |
| SOGA3 | GCKR |  |
| TNF | C2orf16 |  |
| GAL3ST4 | ZNF512 |  |
| FAM120AOS | CCDC121 |  |
| SYNJ2BP | GPN1 |  |
| DHX40 | SUPT7L |  |
| FAM198B | BABAM2 |  |
| TKTL1 | FOSL2 |  |
| TERT | PPP1CB |  |
| HRH4 | SPDYA |  |
| MCCC1 | TRMT61B |  |
| HHAT | WDR43 |  |
| RILPL2 | TOGARAM2 |  |
| ERGIC1 | C2orf71 |  |
| KIAA1456 | CLIP4 |  |
| SNN | ALK |  |
| IRGQ | YPEL5 |  |
| TAF5L | LBH |  |
| NES | LCLAT1 |  |
| C20orf196 | CAPN13 |  |
| DNAJC5 | GALNT14 |  |
| GPR157 | CAPN14 |  |
| RBPMS | EHD3 |  |
| ZNF621 | XDH |  |
| SFSWAP | SRD5A2 |  |
| USP19 | MEMO1 |  |
| FRG2C | SPAST |  |
| CASS4 | SLC30A6 |  |
| KCTD7 | SATB2 |  |
| KCNE3 | FTCDNL1 |  |
| AC137932.1 | CHAF1B |  |
| HOXC10 | CLDN14 |  |
| PRKCA | HLCS |  |
| TRERF1 | RIPPLY3 |  |
| AP3S2 | PIGP |  |
| PVR | TTC3 |  |
| RCVRN | VPS26C |  |
| NEDD1 | DYRK1A |  |
| CABP4 | DHRS7 |  |
| ESPL1 | PPM1A |  |
| TSC22D1 | SIX6 |  |
| CUEDC2 | SIX4 |  |
| PPP2R3B | TRMT5 |  |
| GAB2 | SLC38A6 |  |
| YIF1B | TMEM30B |  |
| SSNA1 | HIF1A |  |
| CLSTN2 | SNAPC1 |  |
| CHRDL1 | RHOJ |  |
| CLU | PPP2R5E |  |
| TIMM22 | WDR89 |  |

| DIXDC1 | SGPP1 |  |
| --- | --- | --- |
| GPRIN2 | SYNE2 |  |
| CDK8 | ESR2 |  |
| PLA2G2F | MTHFD1 |  |
| APLN | ZBTB25 |  |
| WDR5B | AKAP5 |  |
| NOP2 | ZBTB1 |  |
| ZNF3 | PPP1R36 |  |
| ZFP62 | PLEKHG3 |  |
| FADS3 | SPTB |  |
| PAX4 | CHURC1-FNTB |  |
| CCDC25 | CHURC1 |  |
| GLG1 | GPX2 |  |
| NEIL2 | RAB15 |  |
| POTEG | FNTB |  |
| LPAR6 | MAX |  |
| ZCWPW1 | FUT8 |  |
| ZNF502 | GPHN |  |
| FAM26E | FAM71D |  |
| MYH14 | MPP5 |  |
| TMOD3 | ATP6V1D |  |
| METTL9 | EIF2S1 |  |
| ZBTB37 | TMEM229B |  |
| DUSP22 | PLEKHH1 |  |
| SYN3 | ARG2 |  |
| SFRP5 | VTI1B |  |
| EFTUD2 | RDH11 |  |
| RNF185 | RDH12 |  |
| C2orf57 | ZFYVE26 |  |
| HUS1 | RAD51B |  |
| ZNF677 | ZFP36L1 |  |
| REEP2 | ACTN1 |  |
| TXNRD3NB | DCAF5 |  |
| ACER3 | EXD2 |  |
| SV2B | GALNT16 |  |
| HOXB3 | ERH |  |
| RPA2 | SLC39A9 |  |
| LRRC15 | PLEKHD1 |  |
| STK38 | SUSD6 |  |
| SYT2 | SRSF5 |  |
| KIAA1210 | SMOC1 |  |
| RASSF5 | SLC8A3 |  |
| AR | COX16 |  |
| CD6 | SYNJ2BP |  |
| C9orf38 | ADAM20 |  |
| DGCR8 | MED6 |  |
| CBX6 | TTC9 |  |
| CENPP | MAP3K9 |  |
| AACS | PCNX1 |  |
| LDLR | SIPA1L1 |  |
| MEF2B | RGS6 |  |
| LRRC27 | DPF3 |  |
| TGFB1I1 | DCAF4 |  |

| TNIP1 | ZFYVE1 |  |
| --- | --- | --- |
| SIPA1L3 | RBM25 |  |
| PPP1R15B | PSEN1 |  |
| PIGA | PAPLN |  |
| SIPA1 | NUMB |  |
| UST | HEATR4 |  |
| NFIX | RIOX1 |  |
| NRBP2 | ACOT1 |  |
| AKAP5 | ACOT6 |  |
| TRAF1 | DNAL1 |  |
| DCTPP1 | PNMA1 |  |
| RP11-182J1.16 | ELMSAN1 |  |
| HTR3E | PTGR2 |  |
| FAM193B | ZNF410 |  |
| CRYAA | FAM161B |  |
| RPL27A | COQ6 |  |
| GDPGP1 | ENTPD5 |  |
| FAF2 | BBOF1 |  |
| SLC6A4 | ALDH6A1 |  |
| NACC2 | LIN52 |  |
| KCNH1 | VSX2 |  |
| C3orf56 | SYNDIG1L |  |
| SV2C | NPC2 |  |
| NKAIN2 | AREL1 |  |
| KRTAP5-6 | FCF1 |  |
| FAM175B | YLPM1 |  |
| MLLT1 | PROX2 |  |
| CD79B | DLST |  |
| AC016722.1 | RPS6KL1 |  |
| UNC13D | PGF |  |
| OSBPL7 | EIF2B2 |  |
| SELV | MLH3 |  |
| SV2A | ZC2HC1C |  |
| DLGAP1 | NEK9 |  |
| WASF2 | TMED10 |  |
| PROCR | FOS |  |
| POTEM | JDP2 |  |
| ROBO4 | BATF |  |
| PCP4 | FLVCR2 |  |
| NEURL4 | ERG28 |  |
| SLIT3 | TTLL5 |  |
| DGCR2 | TGFB3 |  |
| FZD10 | IFT43 |  |
| SULT1E1 | GPATCH2L |  |
| SPRED2 | ESRRB |  |
| LTBP4 | VASH1 |  |
| GALP | ANGEL1 |  |
| UNC80 | LRRC74A |  |
| SREBF2 | IRF2BPL |  |
| ONECUT2 | CIPC |  |
| POLR2J2 | ZDHHC22 |  |
| GPR4 | TMEM63C |  |
| CDRT4 | POMT2 |  |

| FCGR1A | GSTZ1 |  |
| --- | --- | --- |
| RNF122 | TMED8 |  |
| PSD2 | NOXRED1 |  |
| ZDHHC8 | VIPAS39 |  |
| GPC1 | AHSA1 |  |
| SLC26A6 | ISM2 |  |
| HMOX1 | SPTLC2 |  |
| LBP | ALKBH1 |  |
| PRRT2 | SNW1 |  |
| TDGF1 | ADCK1 |  |
| BRF1 | NRXN3 |  |
| C1QTNF6 | DIO2 |  |
| AMER2 | TSHR |  |
| CTTNBP2NL | GTF2A1 |  |
| WDR91 | STON2 |  |
| GNL3L | FLRT2 |  |
| TCL1B | GALC |  |
| FURIN | GPR65 |  |
| DDX60L | PTPN21 |  |
| SFTPA1 | EML5 |  |
| NYX | FOXN3 |  |
| PPP3R2 | EFCAB11 |  |
| FAM206A | TDP1 |  |
| ARHGAP19-SLIT1 | KCNK13 |  |
| FGFBP3 | NRDE2 |  |
| SLC38A11 | TTC7B |  |
| TMLHE | RPS6KA5 |  |
| GPR162 | DGLUCY |  |
| LZTS1 | GPR68 |  |
| SREBF1 | CCDC88C |  |
| ERAS | PPP4R3A |  |
| DTWD2 | CATSPERB |  |
| SPSB1 | TC2N |  |
| AC024940.1 | FBLN5 |  |
| VOPP1 | TRIP11 |  |
| TMEM63C | ATXN3 |  |
| GLCCI1 | CPSF2 |  |
| C2orf88 | SLC24A4 |  |
| TMC8 | RIN3 |  |
| NTNG2 | LGMN |  |
| ANKRD52 | GOLGA5 |  |
| SLX4 | CHGA |  |
| NUBP2 | TMEM38A |  |
| ZSCAN12 | NWD1 |  |
| USP54 | SIN3B |  |
| KHNYN | F2RL3 |  |
| FAM57A | CPAMD8 |  |
| NDST1 | HAUS8 |  |
| SZT2 | MYO9B |  |
| EPHX1 | OCEL1 |  |
| BTBD7 | USHBP1 |  |
| LTN1 | BABAM1 |  |
| SRXN1 | ABHD8 |  |

| NT5C1A | MRPL34 |  |
| --- | --- | --- |
| ACSS2 | DDA1 |  |
| CNTNAP2 | ANO8 |  |
| RHOJ | GTPBP3 |  |
| UBE2L3 | PLVAP |  |
| AK1 | TMEM221 |  |
| C11orf84 | NXNL1 |  |
| SPNS2 | SLC27A1 |  |
| PRR12 | PGLS |  |
| HAS2 | FAM129C |  |
| RASL10B | COLGALT1 |  |
| FAM222A | UNC13A |  |
| VIPR1 | FCHO1 |  |
| SMYD4 | B3GNT3 |  |
| SPATA8 | JAK3 |  |
| IRAK3 | RPL18A |  |
| XIRP1 | SLC5A5 |  |
| NCOA2 | CCDC124 |  |
| RASL12 | KCNN1 |  |
| TRABD2B | ARRDC2 |  |
| RHOBTB2 | IL12RB1 |  |
| RPH3AL | MAST3 |  |
| FAM155B | PIK3R2 |  |
| PKIG | MPV17L2 |  |
| NUAK2 | PDE4C |  |
| CTD-2162K18.4 | IQCN |  |
| TEF | JUND |  |
| DUSP18 | LSM4 |  |
| C1orf116 | PGPEP1 |  |
| SPZ1 | LRRC25 |  |
| PHACTR1 | SSBP4 |  |
| UBTD1 | ISYNA1 |  |
| FKBP10 | ELL |  |
| PGAM5 | FKBP8 |  |
| APMAP | KXD1 |  |
| VGLL3 | UBA52 |  |
| EPM2A | REX1BD |  |
| SNPH | CRLF1 |  |
| ZNF490 | TMEM59L |  |
| SULT1A3 | KLHL26 |  |
| PPP1R16B | CRTC1 |  |
| APOC4 | UPF1 |  |
| CECR6 | CERS1 |  |
| ST3GAL2 | GDF1 |  |
| SRGAP1 | COPE |  |
| TMEM57 | DDX49 |  |
| RP5-850E9.3 | HOMER3 |  |
| CHST10 | SUGP2 |  |
| CTD-3193O13.9 | ARMC6 |  |
| HS3ST3B1 | SLC25A42 |  |
| CALHM2 | TMEM161A |  |
| DAPK3 | BORCS8 |  |
| PAK6 | RFXANK |  |

| RAB3B | NR2C2AP |  |
| --- | --- | --- |
| C9orf114 | NCAN |  |
| TCP11L2 | HAPLN4 |  |
| NMNAT3 | SUGP1 |  |
| HSPA6 | GATAD2A |  |
| S100PBP | YJEFN3 |  |
| NDRG2 | CILP2 |  |
| RD3 | PBX4 |  |
| INSC | LPAR2 |  |
| ATXN1L | GMIP |  |
| WNT9B | ATP13A1 |  |
| PNPLA3 | ZNF101 |  |
| MIDN | ZNF14 |  |
| OAF | ZNF506 |  |
| NIT2 | ZNF253 |  |
| KCNAB2 | ZNF93 |  |
| USP40 | ZNF682 |  |
| POTEI | ZNF486 |  |
| C12orf36 | ZNF737 |  |
| ACTN1 | ZNF626 |  |
| GOLGA7B | ZNF66 |  |
| CNTN2 | ZNF85 |  |
| AMFR | ZNF714 |  |
| KIF24 | ZNF431 |  |
| FAM89B | ZNF708 |  |
| UBE2S | ZNF738 |  |
| PFDN1 | ZNF493 |  |
| SUFU | ZNF429 |  |
| KCTD17 | ZNF100 |  |
| GLIS3 | ZNF43 |  |
| FAM90A26 | ZNF208 |  |
| SH3PXD2A | ZNF492 |  |
| MTF1 | ZNF99 |  |
| C15orf38-AP3S2 | ZNF728 |  |
| CRHR1 | ZNF730 |  |
| ATP7B | ZNF724 |  |
| SLC39A13 | ZNF681 |  |
| LCN6 | ZNF726 |  |
| TUBGCP6 | ZNF254 |  |
| GDPD5 | UQCRFS1 |  |
| NFE2L1 | VSTM2B |  |
| VPS36 | POP4 |  |
| TMEM248 | C19orf12 |  |
| TMOD2 | CCNE1 |  |
| RAB2B | URI1 |  |
| LEPROTL1 | ZNF536 |  |
| RAP1GAP | TSHZ3 |  |
| FGF7 | ZNF507 |  |
| TNFSF15 | DPY19L3 |  |
| PKNOX2 | PDCD5 |  |
| NUTF2 | ANKRD27 |  |
| TVP23C-CDRT4 | RGS9BP |  |
| SULT1A4 | TDRD12 |  |

| CCDC177 | SLC7A9 |  |
| --- | --- | --- |
| OTOS | CEP89 |  |
| ZNF266 | FAAP24 |  |
| P2RX3 | RHPN2 |  |
| NCDN | GPATCH1 |  |
| WAS | WDR88 |  |
| PKP2 | LRP3 |  |
| CELF2 | SLC7A10 |  |
| WWP2 | CEBPA |  |
| FBXO10 | PEPD |  |
| EPN3 | KCTD15 |  |
| ABCA4 | LSM14A |  |
| PRKCG | KIAA0355 |  |
| GTF3C4 | GPI |  |
| SEC22C | PDCD2L |  |
| ZNF259 | UBA2 |  |
| GLRX3 | WTIP |  |
| SRF | SCGB2B2 |  |
| JMJD1C | ZNF302 |  |
| BHLHA15 | ZNF181 |  |
| FBLN1 | ZNF599 |  |
| RNF38 | ZNF792 |  |
| VCAN | GRAMD1A |  |
| ZNF234 | SCN1B |  |
| LIF | HPN |  |
| ALDH18A1 | FXYD3 |  |
| FAM19A2 | LGI4 |  |
| PABPC1L | FXYD1 |  |
| EMID1 | FXYD5 |  |
| PALM | LSR |  |
| CEP170B | USF2 |  |
| CD99L2 | HAMP |  |
| SALL2 | MAG |  |
| TRIM41 | CD22 |  |
| SH2D6 | FFAR1 |  |
| ZNF853 | KRTDAP |  |
| RP11-192H23.4 | DMKN |  |
| AIF1L | GAPDHS |  |
| CYP2F1 | HAUS5 |  |
| TPPP | RBM42 |  |
| RAB3IL1 | ETV2 |  |
| DDX26B | UPK1A |  |
| SIAH3 | ZBTB32 |  |
| TBL3 | KMT2B |  |
| EMP3 | PSENEN |  |
| TNFSF9 | LIN37 |  |
| BMP8A | PROSER3 |  |
| SMG7 | PRODH2 |  |
| SFXN3 | NPHS1 |  |
| NXPH4 | KIRREL2 |  |
| DAW1 | APLP1 |  |
| ELMSAN1 | NFKBID |  |
| TOM1L2 | HCST |  |

| B3GNT7 | TYROBP |  |
| --- | --- | --- |
| ITPKC | ALKBH6 |  |
| RTEL1 | CLIP3 |  |
| FAM177B | THAP8 |  |
| ACSBG1 | WDR62 |  |
| ANKRD45 | OVOL3 |  |
| TMEM234 | TBCB |  |
| SUPT4H1 | CAPNS1 |  |
| C2orf68 | COX7A1 |  |
| PEX26 | ZNF565 |  |
| AC007040.11 | ZNF146 |  |
| MAP4K4 | ZFP14 |  |
| GRIK3 | ZFP82 |  |
| URGCP-MRPS24 | ZNF566 |  |
| CCR7 | CHD7 |  |
| TMPRSS5 | SSBP2 |  |
| CST8 | ALDH3B2 |  |
| C1QTNF9 | TUBB3 |  |
| F2RL3 | DEF8 |  |
| FNDC8 | RUNX1 |  |
| ASRGL1 | SETD4 |  |
| UPF3A | HECW2 |  |
| BMP8B | CCDC150 |  |
| OSMR | GTF3C3 |  |
| SLC29A4 | C2orf66 |  |
| TBL1X | SPAG9 |  |
| AKT1 | NME1-NME2 |  |
| ERC1 | NME2 |  |
| ATP5E | MBTD1 |  |
| INTS6 | CA10 |  |
| TTYH2 | KIF2B |  |
| KIAA0513 | TOM1L1 |  |
| KLHL21 | COX11 |  |
| MLLT10 | STXBP4 |  |
| TECPR2 | HLF |  |
| HOXB6 | MMD |  |
| CBFA2T2 | TMEM100 |  |
| KXD1 | PCTP |  |
| LCP1 | ANKFN1 |  |
| C9orf57 | NOG |  |
| SRP9 | DGKE |  |
| MS4A14 | TRIM25 |  |
| PRMT8 | COIL |  |
| ATP13A3 | SCPEP1 |  |
| MYCL | AKAP1 |  |
| PDE11A | MSI2 |  |
| ZNF486 | CCDC182 |  |
| FBF1 | MRPS23 |  |
| CELF1 | CUEDC1 |  |
| SH3BP2 | VEZF1 |  |
| DGKD | SRSF1 |  |
| NKX2-5 | OR4D1 |  |
| DOC2B | EPX |  |

| RCSD1 | MKS1 |  |  |
| --- | --- | --- | --- |
| AGAP10 | LPO |  |  |
| CT62 | MPO |  |  |
| ERBB4 | TSPOAP1 |  |  |
| EPB41L4A | SUPT4H1 |  |  |
| UMODL1 | RNF43 |  |  |
| CXCR3 | HSF5 |  |  |
| BEGAIN | MTMR4 |  |  |
| NT5E |  | 4-Sep |  |
| BIN3 | C17orf47 |  |  |
| RP11-302B13.5 | TEX14 |  |  |
| SYT3 | RAD51C |  |  |
| HTR1D | PPM1E |  |  |
| HS3ST4 | TRIM37 |  |  |
| SARM1 | SKA2 |  |  |
| E2F2 | PRR11 |  |  |
| ADRBK1 | SMG8 |  |  |
| KRT6B | GDPD1 |  |  |
| PLEKHG4B | DHX40 |  |  |
| SLC25A51 | CLTC |  |  |
| C9orf47 | PTRH2 |  |  |
| NUDT10 | TUBD1 |  |  |
| CCDC105 | RPS6KB1 |  |  |
| RORB | HEATR6 |  |  |
| MTX3 | CA4 |  |  |
| ARHGEF5 | APPBP2 |  |  |
| GATAD2A | PPM1D |  |  |
| FGD1 | BCAS3 |  |  |
| HLA-DQA2 | TBX2 |  |  |
| UAP1L1 | TBX4 |  |  |
| DNTTIP1 | BRIP1 |  |  |
| VAC14 | INTS2 |  |  |
| GUCA1C | MED13 |  |  |
| ZNF750 | EFCAB3 |  |  |
| KCTD21 | METTL2A |  |  |
| HCAR1 | TLK2 |  |  |
| CD3EAP |  | 10-Mar |  |
| GUCY1A2 | TANC2 |  |  |
| ARL10 | CYB561 |  |  |
| FICD | ACE |  |  |
| DENND3 | KCNH6 |  |  |
| CCSER2 | MAP3K3 |  |  |
| PHF21A | LIMD2 |  |  |
| PRKAR2A | STRADA |  |  |
| RAB22A | CCDC47 |  |  |
| AGAP9 | DDX42 |  |  |
| RTP1 | SMARCD2 |  |  |
| NKAIN4 | CSH2 |  |  |
| HSD3B7 | GH2 |  |  |
| C17orf62 | CSH1 |  |  |
| CLDN2 | CSHL1 |  |  |
| ITGAL | GH1 |  |  |
| C10orf11 | CD79B |  |  |

| PPP2R3A | SCN4A |  |
| --- | --- | --- |
| TCTE1 | PRR29 |  |
| GSS | ICAM2 |  |
| MSRB1 | TEX2 |  |
| SETD5 | PECAM1 |  |
| DAXX | MILR1 |  |
| CYS1 | CEP95 |  |
| EPS8L3 | SMURF2 |  |
| CLEC18A | LRRC37A3 |  |
| STOM | GNA13 |  |
| DDB2 | RGS9 |  |
| FOXP1 | AXIN2 |  |
| GNPNAT1 | CEP112 |  |
| NUP214 | APOH |  |
| KPNA3 | PRKCA |  |
| STON1 | CACNG4 |  |
| KLHDC10 | CACNG1 |  |
| CNKSR1 | HELZ |  |
| MTUS1 | PITPNC1 |  |
| CCDC106 | NOL11 |  |
| TBC1D10A | KPNA2 |  |
| EPN1 | AMZ2 |  |
| FIBCD1 | SLC16A6 |  |
| DMBX1 | ARSG |  |
| KCNS2 | WIPI1 |  |
| CHGA | FAM20A |  |
| PLAC1L | ABCA8 |  |
| SLC25A43 | ABCA9 |  |
| ZNF749 | ABCA10 |  |
| KDSR | ABCA5 |  |
| ST5 | MAP2K6 |  |
| TNKS | KCNJ16 |  |
| GIMAP5 | KCNJ2 |  |
| EVI5L | SOX9 |  |
| ZNF776 | SLC39A11 |  |
| NID1 | COG1 |  |
| TAP1 | FAM104A |  |
| SLC35F2 | CPSF4L |  |
| KMO | SDK2 |  |
| TBC1D29 | RPL38 |  |
| CLIP3 | TTYH2 |  |
| CC2D1B | DNAI2 |  |
| ACRBP | KIF19 |  |
| XBP1 | GPR142 |  |
| ST3GAL1 | GPRC5C |  |
| PMM2 | CD300A |  |
| DUS4L | CD300LB |  |
| TNFRSF11B | CD300LD |  |
| CCDC140 | CD300E |  |
| FP15737 | RAB37 |  |
| INHBB | CD300LF |  |
| ENC1 | SLC9A3R1 |  |
| KIF6 | NAT9 |  |

| NUP205 | TMEM104 |  |  |
| --- | --- | --- | --- |
| MAPK15 | GRIN2C |  |  |
| NOX5 | FDXR |  |  |
| CHTF8 | FADS6 |  |  |
| FRG1B | USH1G |  |  |
| KLF17 | OTOP2 |  |  |
| RBBP4 | OTOP3 |  |  |
| DTX3L | HID1 |  |  |
| CCDC163P | CDR2L |  |  |
| KANK2 | ATP5PD |  |  |
| TBCD | KCTD2 |  |  |
| TSPAN4 | ARMC7 |  |  |
| SYT9 | JPT1 |  |  |
| IGHMBP2 | SUMO2 |  |  |
| GRB2 | NUP85 |  |  |
| PLEKHG5 | GGA3 |  |  |
| SESN2 | MRPS7 |  |  |
| JAKMIP3 | MIF4GD |  |  |
| DLX1 | SLC25A19 |  |  |
| C1orf229 | GRB2 |  |  |
| SIGLEC6 | TMEM94 |  |  |
| KNOP1 | CASKIN2 |  |  |
| RABGEF1 | PPP2R5A |  |  |
| ANGEL1 | TMEM206 |  |  |
| WIPF3 | ATF3 |  |  |
| PELI3 | FAM71A |  |  |
| MPZ | NSL1 |  |  |
| LINC00908 | TATDN3 |  |  |
| ZNF471 | VASH2 |  |  |
| CLCN2 | ANGEL2 |  |  |
| GFOD1 | RPS6KC1 |  |  |
| ABHD6 | PROX1 |  |  |
| LAGE3 | SMYD2 |  |  |
| KCNG1 | PTPN14 |  |  |
| ZNF106 | CENPF |  |  |
| CNTNAP1 | KCNK2 |  |  |
| ADM2 | KCTD3 |  |  |
| NBPF6 | USH2A |  |  |
| CAPN14 | ESRRG |  |  |
| CXCL9 | GPATCH2 |  |  |
| FANCI | RRP15 |  |  |
| PPP1R1C | TGFB2 |  |  |
| COPRS | ZC3H11B |  |  |
| GRK1 | EPRS |  |  |
| LYSMD4 | BPNT1 |  |  |
| GJA3 | IARS2 |  |  |
| COL25A1 | RAB3GAP2 |  |  |
| LIX1 | MARK1 |  |  |
| ITGA6 | C1orf115 |  |  |
| ARFGAP1 |  | 1-Mar |  |
| FUT1 | HLX |  |  |
| NAB2 | DUSP10 |  |  |
| TOR2A | HHIPL2 |  |  |

| ZNF286B | TAF1A |  |
| --- | --- | --- |
| LYRM9 | MIA3 |  |
| RP11-1212A22.4 | AIDA |  |
| HAUS5 | BROX |  |
| SF3A3 | FAM177B |  |
| LUC7L | TLR5 |  |
| ATXN7L3B | SUSD4 |  |
| LTB4R2 | CCDC185 |  |
| DCUN1D1 | CAPN8 |  |
| PLEC | TP53BP2 |  |
| GLYCTK | FBXO28 |  |
| INPP5E | NVL |  |
| CACUL1 | CNIH4 |  |
| RAB5B | WDR26 |  |
| SNAP25 | CNIH3 |  |
| TSPAN6 | LBR |  |
| FZD4 | ENAH |  |
| KCNN3 | EPHX1 |  |
| POLA1 | TMEM63A |  |
| PRICKLE2 | LEFTY1 |  |
| FAM71B | PYCR2 |  |
| TMPRSS6 | LEFTY2 |  |
| EPB41 | SDE2 |  |
| FAM57B | H3F3A |  |
| RGS12 | LIN9 |  |
| NETO2 | ITPKB |  |
| XKRX | PSEN2 |  |
| ARHGAP10 | COQ8A |  |
| PRDM9 | CDC42BPA |  |
| RCC2 | SNAP47 |  |
| FAM90A1 | PRSS38 |  |
| DHDDS | WNT9A |  |
| LIMS2 | ARF1 |  |
| C2orf53 | MRPL55 |  |
| NAP1L6 | GUK1 |  |
| NUDT5 | IBA57 |  |
| SYTL3 | OBSCN |  |
| ZER1 | TRIM11 |  |
| PRDM7 | TRIM17 |  |
| AL583828.1 | HIST3H3 |  |
| TBC1D3F | HIST3H2A |  |
| HOXA7 | RNF187 |  |
| TIMP3 | ACTA1 |  |
| PRKAA1 | NUP133 |  |
| TBC1D3 | ABCB10 |  |
| DOT1L | TAF5L |  |
| HES5 | URB2 |  |
| OLFML2A | GALNT2 |  |
| DIAPH1 | PGBD5 |  |
| CACNB3 | COG2 |  |
| ELF3 | AGT |  |
| KIF4A | CAPN9 |  |
| SUCO | C1orf198 |  |

| ZNF284 | TTC13 |  |
| --- | --- | --- |
| FOXD3 | ARV1 |  |
| PSMG4 | FAM89A |  |
| LHX2 | TRIM67 |  |
| MAP2K2 | C1orf131 |  |
| ZFP36 | EXOC8 |  |
| CHRNA2 | EGLN1 |  |
| PKD2L2 | TSNAX |  |
| CNTNAP5 | DISC1 |  |
| PDZD2 | SIPA1L2 |  |
| GORAB | MAP10 |  |
| ARL5C | NTPCR |  |
| GRIN2D | PCNX2 |  |
| SIGLEC10 | MAP3K21 |  |
| CCDC141 | SLC35F3 |  |
| FBXO48 | COA6 |  |
| TFPI | TARBP1 |  |
| HS6ST3 | IRF2BP2 |  |
| TMEM159 | RBM34 |  |
| HDAC11 | GGPS1 |  |
| CTD-2054N24.2 | B3GALNT2 |  |
| NFIA | GNG4 |  |
| ACTN4 | LYST |  |
| TMEM169 | NID1 |  |
| AL589765.1 | ERO1B |  |
| GCH1 | EDARADD |  |
| MLEC | LGALS8 |  |
| KRTAP19-1 | HEATR1 |  |
| RTTN | ACTN2 |  |
| ANAPC1 | MTR |  |
| KCNV1 | MT1HL1 |  |
| PADI1 | ZP4 |  |
| SCD5 | CHRM3 |  |
| ORM2 | FMN2 |  |
| SDR16C5 | GREM2 |  |
| KIAA1009 | RGS7 |  |
| ADIPOR1 | KMO |  |
| GUF1 | OPN3 |  |
| ZNF391 | CHML |  |
| ORM1 | EXO1 |  |
| IL16 | MAP1LC3C |  |
| CRTC3 | CEP170 |  |
| ZNF195 | SDCCAG8 |  |
| LMBR1 | AKT3 |  |
| RAD23A | ZBTB18 |  |
| CLEC3A | C1orf100 |  |
| PGLYRP2 | CATSPERE |  |
| KCNQ1 | COX20 |  |
| ADAMTSL1 | EFCAB2 |  |
| SLC34A1 | KIF26B |  |
| FAM129C | SMYD3 |  |
| SCN4A | CNST |  |
| SYPL2 | AHCTF1 |  |

| TANC2 | ZNF695 |  |
| --- | --- | --- |
| DUSP28 | ZNF669 |  |
| RNF115 | ZNF124 |  |
| FAM160A1 | ZNF496 |  |
| ACVR1B | NLRP3 |  |
| CTSB | GCSAML |  |
| FBXO31 | OR2G2 |  |
| NCR3LG1 | OR14A2 |  |
| COL4A3 | OR14K1 |  |
| OSBPL5 | OR1C1 |  |
| PDK4 | OR14A16 |  |
| VPS33A | TRIM58 |  |
| ENTPD1 | OR2W3 |  |
| SPOCK2 | OR2L13 |  |
| UNC5B | OR2M5 |  |
| PODN | OR2M2 |  |
| CS | OR2M3 |  |
| C6orf222 | DGKB |  |
| PKLR | MEOX2 |  |
| XKR8 | ISPD |  |
| DNAJC11 | SOSTDC1 |  |
| HES4 | LRRC72 |  |
| POLR2E | ANKMY2 |  |
| FASTK | BZW2 |  |
| SIDT2 | TSPAN13 |  |
| KLHL29 | AHR |  |
| FANCD2OS | SNX13 |  |
| GPR156 | PRPS1L1 |  |
| DENND2C | HDAC9 |  |
| WSB2 | TWIST1 |  |
| PPM1A | FERD3L |  |
| SMC1A | TWISTNB |  |
| UNC5D | TMEM196 |  |
| LSG1 | MACC1 |  |
| XRN1 | ITGB8 |  |
| NANOS2 | ABCB5 |  |
| DCAF4 | SP8 |  |
| DEDD2 | SP4 |  |
| HK1 | DNAH11 |  |
| B4GALT5 | CDCA7L |  |
| CXCR1 | RAPGEF5 |  |
| RIMBP3C | STEAP1B |  |
| LPO | IL6 |  |
| UPK3A | KLHL7 |  |
| DNAH10OS | NUPL2 |  |
| CLEC4E | GPNMB |  |
| FRRS1L | IGF2BP3 |  |
| STARD4 | TRA2A |  |
| EMILIN2 | CCDC126 |  |
| NUDT3 | STK31 |  |
| ITPR2 | MPP6 |  |
| NUDT11 | GSDME |  |
| ESCO2 | OSBPL3 |  |

| ACAD11 | CYCS |  |  |
| --- | --- | --- | --- |
| PAPPA2 | C7orf31 |  |  |
| ANKRD34A | CBX3 |  |  |
| NAGK | SNX10 |  |  |
| PCDH7 | SKAP2 |  |  |
| ZIC3 | HOXA1 |  |  |
| SALL4 | HOXA2 |  |  |
| ACTRT3 | HOXA3 |  |  |
| TMEM110 | HOXA7 |  |  |
| PURA | HOXA9 |  |  |
| BNIP2 | HOXA10 |  |  |
| SRSF11 | HOXA11 |  |  |
| ADAMTS17 | HOXA13 |  |  |
| SYT10 | EVX1 |  |  |
| TBR1 | TAX1BP1 |  |  |
| LPP | JAZF1 |  |  |
| MFN1 | CREB5 |  |  |
| FAM71F2 | TRIL |  |  |
| TMEM182 | CPVL |  |  |
| FGFRL1 | CHN2 |  |  |
| SPTBN2 | PRR15 |  |  |
| AMMECR1L | WIPF3 |  |  |
| DTHD1 | SCRN1 |  |  |
| BSN | FKBP14 |  |  |
| LILRB4 | PLEKHA8 |  |  |
| PPP2R1A | MTURN |  |  |
| PXMP4 | ZNRF2 |  |  |
| PEAR1 | NOD1 |  |  |
| LRRC14 | GGCT |  |  |
| EMP1 | GARS |  |  |
| ZC3H4 | CRHR2 |  |  |
| MCC | INMT |  |  |
| IMPDH1 | MINDY4 |  |  |
| ANO7 | AQP1 |  |  |
| RAB12 | GHRHR |  |  |
| ZNF652 | ADCYAP1R1 |  |  |
| ADAM11 | CCDC129 |  |  |
| ISY1-RAB43 | PPP1R17 |  |  |
| FBXL20 | PDE1C |  |  |
| KCNJ2 | LSM5 |  |  |
| NAV3 | AVL9 |  |  |
| ZIC5 | KBTBD2 |  |  |
| ZNF707 | FKBP9 |  |  |
| C7orf71 | NT5C3A |  |  |
| SETD1B | RP9 |  |  |
| INO80C | BBS9 |  |  |
| CAMK1D | BMPER |  |  |
| CYP20A1 | NPSR1 |  |  |
| SLC30A7 | DPY19L1 |  |  |
| PAPPA | TBX20 |  |  |
| SLC9A2 | HERPUD2 |  |  |
| RPUSD1 |  | 7-Sep |  |
| IYD | EEPD1 |  |  |

| ITPR3 | KIAA0895 |  |
| --- | --- | --- |
| HLF | ANLN |  |
| MARCH9 | AOAH |  |
| SLC13A5 | ELMO1 |  |
| AC074212.3 | GPR141 |  |
| MDM2 | NME8 |  |
| PIP4K2C | SFRP4 |  |
| ZCCHC14 | EPDR1 |  |
| GGA3 | STARD3NL |  |
| DPP8 | AMPH |  |
| MPV17L | VPS41 |  |
| TLR5 | POU6F2 |  |
| GXYLT1 | YAE1D1 |  |
| CAPN5 | CDK13 |  |
| FAM129B | MPLKIP |  |
| PAX8 | SUGCT |  |
| PHLDB1 | INHBA |  |
| CYTH3 | GLI3 |  |
| SRRM4 | PSMA2 |  |
| FOXC1 | HECW1 |  |
| SDC1 | STK17A |  |
| ADCK4 | COA1 |  |
| CUX1 | MRPS24 |  |
| HBP1 | URGCP |  |
| SLC17A9 | UBE2D4 |  |
| CPSF7 | SPDYE1 |  |
| SNAP29 | PGAM2 |  |
| EFCAB14 | POLM |  |
| RIMS3 | AEBP1 |  |
| RPH3A | POLD2 |  |
| NRCAM | MYL7 |  |
| CPLX3 | GCK |  |
| SLC13A4 | YKT6 |  |
| POLR3H | CAMK2B |  |
| MAST3 | NUDCD3 |  |
| ZFP36L1 | NPC1L1 |  |
| ABCB8 | DDX56 |  |
| KIF1A | TMED4 |  |
| SEL1L | OGDH |  |
| DSEL | ZMIZ2 |  |
| EPHA8 | PPIA |  |
| CPLX2 | H2AFV |  |
| IFITM10 | PURB |  |
| PRRG4 | MYO1G |  |
| EMR2 | CCM2 |  |
| ZBTB34 | NACAD |  |
| AQP2 | TBRG4 |  |
| LY86 | RAMP3 |  |
| EXOC6B | ADCY1 |  |
| SAMD1 | IGFBP1 |  |
| SEZ6 | IGFBP3 |  |
| DPP6 | TNS3 |  |
| SAMD9 | PKD1L1 |  |

| TMPPE | SUN3 |  |
| --- | --- | --- |
| ZFAND3 | C7orf57 |  |
| URGCP | UPP1 |  |
| ARHGAP31 | ABCA13 |  |
| RPL7L1 | CDC14C |  |
| SLC16A2 | ZPBP |  |
| MMP11 | SPATA48 |  |
| SLCO2A1 | IKZF1 |  |
| PSKH1 | FIGNL1 |  |
| OAS1 | DDC |  |
| PGM5 | GRB10 |  |
| TAL1 | ATRX |  |
| ZSCAN32 | MAGT1 |  |
| APBB2 | ATP7A |  |
| SFTPB | PGAM4 |  |
| EEF2K | PGK1 |  |
| SPPL2A | TAF9B |  |
| PSMB5 | LPAR4 |  |
| OTUD3 | P2RY10 |  |
| AKR1C2 | GPR174 |  |
| ZNF286A | ITM2A |  |
| C8orf46 | TBX22 |  |
| GABRA4 | BRWD3 |  |
| GAREML | HMGN5 |  |
| ACSM1 | SH3BGRL |  |
| FCHSD1 | POU3F4 |  |
| TJAP1 | RPS6KA6 |  |
| IGFBP5 | HDX |  |
| IGFBP3 | APOOL |  |
| CTD-2207O23.12 | POF1B |  |
| CARM1 | CHM |  |
| KRT80 | DACH2 |  |
| USP33 | KLHL4 |  |
| UBALD1 | TGIF2LX |  |
| CHPF | PABPC5 |  |
| ADCY6 | PCDH11X |  |
| SPEF2 | NAP1L3 |  |
| HAGHL | FAM133A |  |
| TUBB1 | DIAPH2 |  |
| IFITM5 | RPA4 |  |
| PRSS42 | PCDH19 |  |
| IGLL1 | TSPAN6 |  |
| SGK494 | SRPX2 |  |
| C17orf70 | SYTL4 |  |
| RAD54L2 | CSTF2 |  |
| PRKX | NOX1 |  |
| YBX3 | XKRX |  |
| FGF22 | ARL13A |  |
| FLRT3 | TRMT2B |  |
| ZNF75A | TMEM35A |  |
| ABHD4 | CENPI |  |
| SMARCAD1 | DRP2 |  |
| ZDBF2 | TAF7L |  |

| ELOVL5 | BTK |  |
| --- | --- | --- |
| TLR6 | RPL36A |  |
| ANKRD11 | GLA |  |
| FIZ1 | ARMCX4 |  |
| SCN8A | ARMCX1 |  |
| CTCFL | ARMCX6 |  |
| MAPK14 | ARMCX3 |  |
| KAZALD1 | ZMAT1 |  |
| RAP2B | BEX5 |  |
| SNRNP70 | NXF2 |  |
| LIMK2 | NXF2B |  |
| NKX3-2 | TCP11X2 |  |
| FNTB | TMSB15A |  |
| TMED7-TICAM2 | ARMCX5 |  |
| ZNF667 | ARMCX5-GPRASP2 |  |
| RFTN2 | GPRASP2 |  |
| MED17 | BHLHB9 |  |
| TRIM65 | RAB40AL |  |
| FGFR1 | BEX1 |  |
| PACS1 | NXF3 |  |
| SYAP1 | BEX4 |  |
| LUZPP1 | TCEAL8 |  |
| CLHC1 | BEX2 |  |
| PAQR4 | TCEAL7 |  |
| EYA1 | TCEAL4 |  |
| SPARC | TCEAL3 |  |
| TRIM14 | PLP1 |  |
| SLC35F1 | RAB9B |  |
| BHLHB9 | H2BFWT |  |
| OXSR1 | H2BFM |  |
| VDR | SLC25A53 |  |
| SIRPA | ZCCHC18 |  |
| PHF15 | FAM199X |  |
| ZNF92 | TEX13A |  |
| PHC2 | NRK |  |
| EIF3J | SERPINA7 |  |
| SLC5A7 | MUM1L1 |  |
| KIAA1468 | CXorf57 |  |
| HIF1AN | RIPPLY1 |  |
| RRM2B | CLDN2 |  |
| FUT11 | MORC4 |  |
| E2F1 | RBM41 |  |
| SRM | NUP62CL |  |
| SLC6A20 | PIH1D3 |  |
| CLNK | FRMPD3 |  |
| SLFN11 | PRPS1 |  |
| CIC | TSC22D3 |  |
| RASD2 | NCBP2L |  |
| AC025278.1 | MID2 |  |
| CARD8 | VSIG1 |  |
| CRY2 | PSMD10 |  |
| SBF2 | COL4A6 |  |
| SLC22A7 | COL4A5 |  |

| MAMSTR | IRS4 |  |  |
| --- | --- | --- | --- |
| TMEM173 | GUCY2F |  |  |
| COL28A1 | NXT2 |  |  |
| ADARB2 | KCNE5 |  |  |
| CCDC78 | ACSL4 |  |  |
| GCHFR | TMEM164 |  |  |
| ARL4C | AMMECR1 |  |  |
| C9orf163 | RTL9 |  |  |
| SCARF1 | CHRDL1 |  |  |
| CYP2S1 | PAK3 |  |  |
| RAX | CAPN6 |  |  |
| FRS2 | DCX |  |  |
| MXRA7 | SERTM2 |  |  |
| DYM | ALG13 |  |  |
| CDR2 | TRPC5 |  |  |
| ZNF442 | TRPC5OS |  |  |
| C18orf25 | RTL4 |  |  |
| MKL2 | LHFPL1 |  |  |
| GINS2 | AMOT |  |  |
| LMO3 | HTR2C |  |  |
| LRRC3C | IL13RA2 |  |  |
| FRMD8 | LRCH2 |  |  |
| XCR1 | SLC6A14 |  |  |
| ADAL | CT83 |  |  |
| PLEKHA1 | KLHL13 |  |  |
| SYT6 | WDR44 |  |  |
| LRRN1 | DOCK11 |  |  |
| BTBD3 | IL13RA1 |  |  |
| MYLK2 | LONRF3 |  |  |
| C17orf103 | KIAA1210 |  |  |
| TVP23A | PGRMC1 |  |  |
| CYP4F3 | SLC25A43 |  |  |
| DIEXF | SLC25A5 |  |  |
| ORC2 | CXorf56 |  |  |
| CRLF1 | NKRF |  |  |
| PTK2 |  | 6-Sep |  |
| STX5 | SOWAHD |  |  |
| KREMEN2 | UPF3B |  |  |
| ARHGEF39 | RNF113A |  |  |
| KDM4E | NKAP |  |  |
| NIPSNAP3B | RHOXF2B |  |  |
| DUSP7 | RHOXF1 |  |  |
| PAFAH2 | RHOXF2 |  |  |
| SF3A1 | ZBTB33 |  |  |
| DFFA | TMEM255A |  |  |
| SUGT1 | ATP1B4 |  |  |
| C3orf72 | LAMP2 |  |  |
| RAB17 | CUL4B |  |  |
| SLC5A1 | MCTS1 |  |  |
| HTR3D | CT47B1 |  |  |
| RASGRP4 | CT47A12 |  |  |
| SLC7A8 | CT47A11 |  |  |
| CMTM3 | CT47A10 |  |  |

| CIAO1 | CT47A9 |  |
| --- | --- | --- |
| DUSP8 | CT47A8 |  |
| ZFP14 | CT47A7 |  |
| RXFP3 | CT47A6 |  |
| FAM20B | CT47A5 |  |
| PPP4C | CT47A4 |  |
| ANKS1B | CT47A3 |  |
| NEURL1B | CT47A2 |  |
| MYOCD | CT47A1 |  |
| OPRK1 | GLUD2 |  |
| GAL3ST3 | GRIA3 |  |
| GRM6 | THOC2 |  |
| CEMP1 | XIAP |  |
| AKNA | STAG2 |  |
| MFHAS1 | TEX13D |  |
| NPTX1 | TENM1 |  |
| EPG5 | TEX13C |  |
| SHPK | DCAF12L2 |  |
| TNFRSF13C | DCAF12L1 |  |
| TRPV1 | PRR32 |  |
| ESAM | ACTRT1 |  |
| MON1B | SMARCA1 |  |
| A4GNT | OCRL |  |
| HRH2 | XPNPEP2 |  |
| ZNF541 | SASH3 |  |
| GDI2 | ZDHHC9 |  |
| SLC30A4 | BCORL1 |  |
| MUC20 | ELF4 |  |
| SFXN5 | AIFM1 |  |
| PSAPL1 | ZNF280C |  |
| SLC1A3 | SLC25A14 |  |
| CABP7 | GPR119 |  |
| WWOX | ENOX2 |  |
| C21orf128 | ARHGAP36 |  |
| MGRN1 | IGSF1 |  |
| CAV1 | OR13H1 |  |
| HLCS | STK26 |  |
| ACKR2 | FRMD7 |  |
| JOSD1 | RAP2C |  |
| PTPLAD1 | MBNL3 |  |
| PLEKHA7 | HS6ST2 |  |
| TAF4B | USP26 |  |
| SLC22A5 | TFDP3 |  |
| ZC3H6 | GPC4 |  |
| PALD1 | GPC3 |  |
| CCDC137 | CCDC160 |  |
| LDB3 | PHF6 |  |
| IL2RB | INSC |  |
| HSD11B1L | SOX6 |  |
| NBPF20 | C11orf58 |  |
| CCDC180 | PLEKHA7 |  |
| ELL | PIK3C2A |  |
| DLGAP4 | NUCB2 |  |

| RAPGEF1 | NCR3LG1 |  |
| --- | --- | --- |
| ZFP91 | KCNJ11 |  |
| AXIN1 | ABCC8 |  |
| TRABD2A | USH1C |  |
| STEAP2 | OTOG |  |
| P2RY13 | MYOD1 |  |
| FMNL1 | KCNC1 |  |
| HTT | SERGEF |  |
| NBEAL1 | TPH1 |  |
| SLIT1 | SAAL1 |  |
| STC1 | HPS5 |  |
| PTPN14 | GTF2H1 |  |
| H2AFV | LDHA |  |
| PNLIPRP3 | LDHC |  |
| CELF5 | LDHAL6A |  |
| PCDHB6 | UEVLD |  |
| WNK2 | SPTY2D1OS |  |
| ZNF70 | SPTY2D1 |  |
| BSDC1 | TMEM86A |  |
| MYH10 | IGSF22 |  |
| NCOA7 | PTPN5 |  |
| NUGGC | MRGPRX2 |  |
| ASIC2 | ZDHHC13 |  |
| SGSH | CSRP3 |  |
| HIPK2 | NAV2 |  |
| NRF1 | DBX1 |  |
| FBXO28 | HTATIP2 |  |
| TMEM81 | PRMT3 |  |
| ARNTL2 | SLC6A5 |  |
| DCTN5 | NELL1 |  |
| CHST11 | ANO5 |  |
| ALDOB | FANCF |  |
| TMEM63B | SVIP |  |
| BARX1 | LUZP2 |  |
| SALL1 | ANO3 |  |
| TDRD9 | MUC15 |  |
| AXDND1 | SLC5A12 |  |
| C1orf177 | CCDC34 |  |
| TAP2 | LGR4 |  |
| MAFG | LIN7C |  |
| KMT2B | BDNF |  |
| RARG | KIF18A |  |
| SERPINE2 | KCNA4 |  |
| MAP4 | ARL14EP |  |
| TMEM87A | MPPED2 |  |
| PLXDC1 | DCDC1 |  |
| RNASEH2C | DNAJC24 |  |
| GREB1 | IMMP1L |  |
| SNIP1 | ELP4 |  |
| SYCP2L | PAX6 |  |
| THSD7A | RCN1 |  |
| SAMD4A | WT1 |  |
| SEC14L5 | EIF3M |  |

| CLIP2 | PRRG4 |  |
| --- | --- | --- |
| SLC14A1 | QSER1 |  |
| HCCS | DEPDC7 |  |
| SKA3 | TCP11L1 |  |
| HOXC6 | HIPK3 |  |
| SFTPA2 | KIAA1549L |  |
| FMN1 | C11orf91 |  |
| TNRC18 | CD59 |  |
| SLAMF6 | FBXO3 |  |
| MED13L | LMO2 |  |
| HOOK3 | CAPRIN1 |  |
| AP5S1 | ABTB2 |  |
| CREBL2 | ELF5 |  |
| MCPH1 | EHF |  |
| ZBTB38 | APIP |  |
| TYRO3 | CD44 |  |
| RFTN1 | SLC1A2 |  |
| PHYHIP | PAMR1 |  |
| HAND2 | FJX1 |  |
| SMARCC2 | TRIM44 |  |
| ZBTB16 | LDLRAD3 |  |
| MUM1 | COMMD9 |  |
| TMCC1 | PRR5L |  |
| TBC1D24 | TRAF6 |  |
| FAM19A4 | RAG1 |  |
| TATDN2 | RAG2 |  |
| MARK2 | C11orf74 |  |
| PCP4L1 | LRRC4C |  |
| OPA1 | API5 |  |
| SNAPC5 | TTC17 |  |
| ENPP1 | HSD17B12 |  |
| TIRAP | ACCS |  |
| ZNF773 | EXT2 |  |
| ZMIZ1 | ALX4 |  |
| GPR182 | CD82 |  |
| MICALCL | TSPAN18 |  |
| TPM3 | TP53I11 |  |
| TRAM2 | PRDM11 |  |
| ADH6 | SYT13 |  |
| SERPINB9 | CHST1 |  |
| KCNMB1 | SLC35C1 |  |
| PFKFB1 | CRY2 |  |
| SOX13 | MAPK8IP1 |  |
| SNTA1 | C11orf94 |  |
| KCNB1 | LARGE2 |  |
| USP27X | PHF21A |  |
| YOD1 | CREB3L1 |  |
| DLAT | DGKZ |  |
| SGSM2 | MDK |  |
| MMP16 | AMBRA1 |  |
| CYP27B1 | ATG13 |  |
| IL7 | ARHGAP1 |  |
| PLXNB2 | ZNF408 |  |

| FGFR3 | F2 |  |
| --- | --- | --- |
| ESR1 | C11orf49 |  |
| EIF2AK2 | ARFGAP2 |  |
| SLC9A9 | PACSIN3 |  |
| BMP7 | DDB2 |  |
| IPP | ACP2 |  |
| GRB10 | NR1H3 |  |
| C19orf57 | MADD |  |
| HLA-DOA | MYBPC3 |  |
| ZC3H7B | SPI1 |  |
| CHD6 | SLC39A13 |  |
| MMP8 | PSMC3 |  |
| KCTD14 | CELF1 |  |
| ALPP | PTPMT1 |  |
| CALHM3 | FAM180B |  |
| PSG1 | C1QTNF4 |  |
| RFC2 | MTCH2 |  |
| SLC16A4 | AGBL2 |  |
| STIM1 | NUP160 |  |
| ARHGAP26 | PTPRJ |  |
| EMC8 | OR4C5 |  |
| CLIC6 | TRIM49B |  |
| ACAD9 | TRIM64C |  |
| CSNK1G3 | IL11RA |  |
| AGXT | LOC730098 |  |
| LANCL1 | CCL19 |  |
| STARD5 | FAM205A |  |
| SUPT16H | FAM205C |  |
| RGS19 | PHF24 |  |
| KIAA1324 | DNAJB5 |  |
| DNAH11 | C9orf131 |  |
| FAM21D | VCP |  |
| FCN2 | PIGO |  |
| PLEKHG3 | STOML2 |  |
| WLS | FAM214B |  |
| TRIM33 | UNC13B |  |
| SLC8A1 | RUSC2 |  |
| UCK2 | FAM166B |  |
| SCAMP5 | TESK1 |  |
| APCDD1L | SIT1 |  |
| LEPRE1 | ARHGEF39 |  |
| DPF1 | CA9 |  |
| SHC1 | TPM2 |  |
| DENND1C | TLN1 |  |
| RAB8A | CREB3 |  |
| NAA60 | GBA2 |  |
| ARHGEF19 | RGP1 |  |
| KPNA1 | MSMP |  |
| ACP5 | NPR2 |  |
| AGO1 | SPAG8 |  |
| LURAP1 | HINT2 |  |
| CLDN6 | FAM221B |  |
| TSPAN9 | TMEM8B |  |

| SEC16B | OR13J1 |  |
| --- | --- | --- |
| TMEM201 | HRCT1 |  |
| FGD3 | SPAAR |  |
| NBPF4 | OR2S2 |  |
| EDA2R | GLIPR2 |  |
| PDRG1 | CCIN |  |
| RAPGEF2 | CLTA |  |
| CTIF | GNE |  |
| BEAN1 | RNF38 |  |
| SURF4 | MELK |  |
| GAPVD1 | PAX5 |  |
| FLT4 | ZCCHC7 |  |
| MMEL1 | GRHPR |  |
| TPCN1 | ZBTB5 |  |
| CTD-2616J11.4 | POLR1E |  |
| MT1A | FBXO10 |  |
| ZNF23 | FRMPD1 |  |
| ZC3H12D | TRMT10B |  |
| SPOCK1 | EXOSC3 |  |
| POLR1A | DCAF10 |  |
| DNAJB1 | SLC25A51 |  |
| OGFOD2 | SHB |  |
| VSTM2B | ALDH1B1 |  |
| THRB | CNTNAP3 |  |
| ATG16L1 | SPATA31A1 |  |
| GPSM3 | FOXD4L6 |  |
| ADAMTS2 | SPATA31A6 |  |
| FBXL22 | SPATA31A5 |  |
| PNRC2 | SPATA31A7 |  |
| GABRA3 | FOXD4L5 |  |
| PNMAL2 | FOXD4L4 |  |
| UNC119 | ZNF658 |  |
| PLCG1 | SPATA31A3 |  |
| LRFN4 | FOXD4L3 |  |
| DIRAS1 | PGM5 |  |
| BCAP29 | TMEM252 |  |
| GPR68 | PIP5K1B |  |
| TPGS2 | FAM122A |  |
| SCN3B | PRKACG |  |
| DIAPH2 | FXN |  |
| RABIF | TJP2 |  |
| C5orf24 | FAM189A2 |  |
| PVRL1 | APBA1 |  |
| ZNF491 | PTAR1 |  |
| CDH4 | MAMDC2 |  |
| CEACAM6 | SMC5 |  |
| MAL | KLF9 |  |
| DLG3 | TRPM3 |  |
| WSCD1 | TMEM2 |  |
| ZNF514 | ABHD17B |  |
| SGPP2 | C9orf85 |  |
| TADA2B | C9orf57 |  |
| RSBN1 | GDA |  |

| TRIOBP | ZFAND5 |  |
| --- | --- | --- |
| CD84 | TMC1 |  |
| IGF2BP1 | ANXA1 |  |
| PPL | RORB |  |
| FAM124A | TRPM6 |  |
| AMER1 | C9orf40 |  |
| ZNF629 | CARNMT1 |  |
| B3GALT1 | NMRK1 |  |
| ZNF436 | PCSK5 |  |
| ANPEP | GCNT1 |  |
| IRF4 | PRUNE2 |  |
| CCDC40 | VPS13A |  |
| IL21R | GNA14 |  |
| CSPG4 | GNAQ |  |
| RASGEF1C | CEP78 |  |
| PITX1 | PSAT1 |  |
| GAN | TLE4 |  |
| ATOX1 | SPATA31D1 |  |
| KLRC4 | RASEF |  |
| PPP1R7 | FRMD3 |  |
| C20orf112 | IDNK |  |
| ZDHHC24 | UBQLN1 |  |
| CSRNP3 | KIF27 |  |
| MAFF | C9orf64 |  |
| MAMLD1 | HNRNPK |  |
| ADRA2C | RMI1 |  |
| KIAA1033 | SLC28A3 |  |
| ZBTB42 | NTRK2 |  |
| YPEL1 | AGTPBP1 |  |
| ZNF805 | GOLM1 |  |
| RASAL1 | ZCCHC6 |  |
| POU3F3 | DAPK1 |  |
| MTR | CTSL |  |
| AFF2 | SPATA31E1 |  |
| RIMS4 | CDK20 |  |
| LPGAT1 | SPIN1 |  |
| LYVE1 | NXNL2 |  |
| PCBD2 | C9orf47 |  |
| SLC24A2 | S1PR3 |  |
| NFATC1 | SHC3 |  |
| SERPINA1 | SECISBP2 |  |
| AC110781.3 | SEMA4D |  |
| MFAP2 | GADD45G |  |
| GBF1 | DIRAS2 |  |
| ADCY5 | SYK |  |
| ZKSCAN3 | NOL8 |  |
| CCDC61 | CENPP |  |
| DOK6 | OGN |  |
| UNC13A | OMD |  |
| ZNF726 | ECM2 |  |
| TSTD2 | IPPK |  |
| GABPB2 | BICD2 |  |
| KIRREL2 | ZNF484 |  |

| SH2D5 | FGD3 |  |
| --- | --- | --- |
| TMEM259 | SUSD3 |  |
| C1orf200 | CARD19 |  |
| SPRED3 | NINJ1 |  |
| CRTC1 | WNK2 |  |
| C19orf55 | FAM120AOS |  |
| SLC18B1 | FAM120A |  |
| FAM153C | PHF2 |  |
| C15orf27 | BARX1 |  |
| MCHR1 | PTPDC1 |  |
| LPL | NUTM2F |  |
| ZBTB7A | MFSD14B |  |
| EPHB2 | FBP1 |  |
| TMEM25 | C9orf3 |  |
| FYB | FANCC |  |
| CD34 | PTCH1 |  |
| RIN3 | ERCC6L2 |  |
| IQSEC3 | HSD17B3 |  |
| ERG | CDC14B |  |
| TBC1D30 | ZNF510 |  |
| ZFHX4 | ZNF782 |  |
| NFAT5 | NUTM2G |  |
| NLE1 | MFSD14C |  |
| AC007390.5 | CTSV |  |
| PLXNA4 | CCDC180 |  |
| ANKRD34C | TDRD7 |  |
| RNPEPL1 | TMOD1 |  |
| STK11 | VPS53 |  |
| ATP7A | FAM57A |  |
| CYP46A1 | GEMIN4 |  |
| L3MBTL4 | GLOD4 |  |
| AC004466.1 | ABR |  |
| ZNF213 | BHLHA9 |  |
| TMEM55B | TUSC5 |  |
| PLCH2 | CRK |  |
| RNF220 | MYO1C |  |
| CABLES2 | INPP5K |  |
| ATP6V1C1 | TRPV3 |  |
| NAA50 | TRPV1 |  |
| CERS6 | SHPK |  |
| BYSL | CTNS |  |
| ARHGAP32 | TAX1BP3 |  |
| BCORL1 | EMC6 |  |
| RBMX2 | P2RX5 |  |
| FAM168A | ITGAE |  |
| FXN | HASPIN |  |
| SH3BGRL2 | NCBP3 |  |
| ZNF570 | CAMKK1 |  |
| GLI3 | P2RX1 |  |
| FBXO42 | ATP2A3 |  |
| EHD3 | ZZEF1 |  |
| NCMAP | CYB5D2 |  |
| PPM1F | ANKFY1 |  |

| ZFHX2 | UBE2G1 |  |
| --- | --- | --- |
| CBS | SPNS3 |  |
| FRAT1 | MYBBP1A |  |
| IKBKAP | GGT6 |  |
| ZNF765 | ALOX15 |  |
| TNFRSF12A | PELP1 |  |
| OXTR | ARRB2 |  |
| SNX10 | MED11 |  |
| PET112 | CXCL16 |  |
| MOV10 | NOLC1 |  |
| CLIC5 | ELOVL3 |  |
| WDR92 | GBF1 |  |
| SHE | NFKB2 |  |
| RBFOX2 | PSD |  |
| RNF144A | FBXL15 |  |
| IFT80 | CUEDC2 |  |
| ACAN | MFSD13A |  |
| MMP17 | ACTR1A |  |
| SIM1 | SUFU |  |
| METTL15 | TRIM8 |  |
| MSTN | ARL3 |  |
| EIF2AK3 | SFXN2 |  |
| VENTX | WBP1L |  |
| RBM19 | CYP17A1 |  |
| POC1B | BORCS7 |  |
| ADAM12 | CNNM2 |  |
| UNC5C | NT5C2 |  |
| MBNL3 | INA |  |
| RPS15A | PCGF6 |  |
| FAM46B | ATP5MD |  |
| RAD51L3-RFFL | PDCD11 |  |
| DDX6 | CALHM2 |  |
| HMX2 | CALHM1 |  |
| NEK8 | NEURL1 |  |
| ADD1 | SH3PXD2A |  |
| TEX2 | STN1 |  |
| RILPL1 | SLK |  |
| CACNG4 | COL17A1 |  |
| QPCTL | GSTO1 |  |
| KIAA1919 | GSTO2 |  |
| ANKRD12 | ITPRIP |  |
| CD200R1 | SORCS3 |  |
| DAG1 | XPNPEP1 |  |
| FAIM2 | ADD3 |  |
| SYNE1 | MXI1 |  |
| PHF7 | SMNDC1 |  |
| C1QBP | DUSP5 |  |
| TNNI1 | RBM20 |  |
| SLC12A8 | PDCD4 |  |
| LRRC59 | BBIP1 |  |
| C3orf18 | ADRA2A |  |
| RANGRF | GPAM |  |
| HAP1 | TECTB |  |

| KLF12 | ACSL5 |  |
| --- | --- | --- |
| NPY4R | ZDHHC6 |  |
| ZC3H13 | TCF7L2 |  |
| POLR2F | HABP2 |  |
| RND2 | NRAP |  |
| ARF1 | CASP7 |  |
| RAB32 | PLEKHS1 |  |
| RWDD1 | DCLRE1A |  |
| PSMD8 | ADRB1 |  |
| PPCDC | CCDC186 |  |
| PADI2 | TDRD1 |  |
| ANP32A | VWA2 |  |
| GPD1 | AFAP1L2 |  |
| PREX2 | ABLIM1 |  |
| C20orf197 | FAM160B1 |  |
| NUP62 | GFRA1 |  |
| RP11-17M16.1 | CCDC172 |  |
| USP42 | PNLIPRP3 |  |
| SLC35D3 | PNLIP |  |
| GPR114 | C10orf82 |  |
| HELZ2 | HSPA12A |  |
| MARCH5 | ENO4 |  |
| CECR2 | SHTN1 |  |
| PRSS21 | VAX1 |  |
| SULT1C4 | KCNK18 |  |
| TTBK2 | SLC18A2 |  |
| LDOC1L | PDZD8 |  |
| PHF2 | EMX2 |  |
| C17orf107 | RAB11FIP2 |  |
| SLC25A42 | FAM204A |  |
| TEX261 | PRLHR |  |
| PTGES | CACUL1 |  |
| MALL | NANOS1 |  |
| ESPNL | FAM45A |  |
| VAT1L | SFXN4 |  |
| ALG1 | PRDX3 |  |
| TMEM251 | GRK5 |  |
| STOML1 | TIAL1 |  |
| BCL2 | BAG3 |  |
| FZD3 | INPP5F |  |
| RBM8A | SEC23IP |  |
| SLC35B1 | PLPP4 |  |
| IFIT5 | FGFR2 |  |
| HIST1H2AH | ATE1 |  |
| APPBP2 | NSMCE4A |  |
| TBC1D13 | TACC2 |  |
| PTGDR2 | BTBD16 |  |
| TKTL2 | PLEKHA1 |  |
| NYAP2 | DMBT1 |  |
| SLCO1A2 | C10orf120 |  |
| ZBTB4 | CUZD1 |  |
| RAB11FIP4 | C10orf88 |  |
| GOLM1 | PSTK |  |

| EN2 | IKZF5 |  |
| --- | --- | --- |
| TRIB3 | ACADSB |  |
| COX20 | HMX3 |  |
| C15orf57 | HMX2 |  |
| ADCYAP1R1 | BUB3 |  |
| SNX11 | GPR26 |  |
| IL17D | CPXM2 |  |
| PTPRT | CHST15 |  |
| ENDOD1 | NKX1-2 |  |
| FAM124B | LHPP |  |
| DUS1L | FAM53B |  |
| CREB3L2 | EEF1AKMT2 |  |
| MRPS21 | ABRAXAS2 |  |
| CLCN5 | CTBP2 |  |
| ARPP19 | EDRF1 |  |
| PTPN11 | MMP21 |  |
| WDFY4 | UROS |  |
| KAT6A | DHX32 |  |
| ARHGAP11A | ADAM12 |  |
| KCNE4 | C10orf90 |  |
| CYP11B2 | DOCK1 |  |
| TRAF7 | FAM196A |  |
| SLC7A2 | MKI67 |  |
| PIP4K2A | MGMT |  |
| SLC39A10 | EBF3 |  |
| SERBP1 | C10orf143 |  |
| ARSE | GLRX3 |  |
| RFFL | TCERG1L |  |
| POLDIP2 | PPP2R2D |  |
| PTPN3 | JAKMIP3 |  |
| HMGA2 | DPYSL4 |  |
| ZNF512 | STK32C |  |
| ANO5 | LRRC27 |  |
| ARRDC3 | PWWP2B |  |
| GALNT9 | NKX6-2 |  |
| MARCH8 | CFAP46 |  |
| CLDND1 | ADGRA1 |  |
| DNAJC3 | KNDC1 |  |
| EMX1 | UTF1 |  |
| NUP155 | VENTX |  |
| MMP24 | TUBGCP2 |  |
| FOXP2 | ZNF511 |  |
| METTL16 | CALY |  |
| WBSCR27 | PRAP1 |  |
| TMEM92 | FUOM |  |
| ZNF264 | PAOX |  |
| NAA30 | MTG1 |  |
| DNAJB13 | SPRN |  |
| MURC | SYCE1 |  |
| GABRB2 | FRG2B |  |
| RBFOX3 | ODF3 |  |
| C2CD2 | BET1L |  |
| EXPH5 | RIC8A |  |

| SECTM1 | SIRT3 |  |
| --- | --- | --- |
| IFNAR1 | PSMD13 |  |
| NONO | NLRP6 |  |
| AC007375.1 | PGGHG |  |
| TNIK | IFITM5 |  |
| CDK5 | IFITM1 |  |
| SETDB1 | IFITM3 |  |
| TCEAL7 | B4GALNT4 |  |
| PDE5A | PKP3 |  |
| CHURC1-FNTB | SIGIRR |  |
| C21orf91 | ANO9 |  |
| RNASEH1 | RNH1 |  |
| SEMA3F | HRAS |  |
| KPNA6 | LRRC56 |  |
| GBP5 | LMNTD2 |  |
| NUDT16L1 | RASSF7 |  |
| CHKB | PHRF1 |  |
| CAMKK1 | IRF7 |  |
| CTU1 | CDHR5 |  |
| LARP1 | SLCO5A1 |  |
| QSOX1 | PRDM14 |  |
| TLN2 | NCOA2 |  |
| PRICKLE1 | TRAM1 |  |
| POFUT2 | LACTB2 |  |
| PLXNA3 | EYA1 |  |
| ENTPD4 | MSC |  |
| CAPZB | TRPA1 |  |
| AXIN2 | KCNB2 |  |
| PDGFRB | TERF1 |  |
| TRIM67 | SBSPON |  |
| ACADS | RDH10 |  |
| LL22NC03-63E9.3 | STAU2 |  |
| POLR1D | UBE2W |  |
| ZNF431 | TMEM70 |  |
| ATXN3 | JPH1 |  |
| KCNC1 | PI15 |  |
| DCLRE1C | CRISPLD1 |  |
| HOXD3 | HNF4G |  |
| FTO | ZFHX4 |  |
| STARD8 | PEX2 |  |
| DGKG | OR4F29 |  |
| ISM2 | OR4F16 |  |
| ZNF117 | SAMD11 |  |
| GLI4 | PLEKHN1 |  |
| CTBS | PERM1 |  |
| PHACTR2 | HES4 |  |
| RALGAPA2 | ISG15 |  |
| CCDC9 | AGRN |  |
| ACACA | RNF223 |  |
| GALM | C1orf159 |  |
| NTSR1 | TTLL10 |  |
| SLC6A9 | TNFRSF18 |  |
| CACNA2D2 | B3GALT6 |  |

| FSD2 | UBE2J2 |  |
| --- | --- | --- |
| XYLT1 | SCNN1D |  |
| REPIN1 | ACAP3 |  |
| GTF2A1 | PUSL1 |  |
| CES4A | INTS11 |  |
| PAIP2B | TAS1R3 |  |
| PPIG | DVL1 |  |
| KITLG | MXRA8 |  |
| PYCRL | AURKAIP1 |  |
| DOCK5 | CCNL2 |  |
| VPS41 | ANKRD65 |  |
| ZBTB7C | VWA1 |  |
| TESPA1 | ATAD3C |  |
| SPIRE2 | ATAD3B |  |
| COLCA1 | ATAD3A |  |
| KCNK3 | TMEM240 |  |
| TBC1D2B | MIB2 |  |
| AREL1 | MMP23B |  |
| TMEM130 | CDK11B |  |
| ABI2 | SLC35E2B |  |
| STX17 | CDK11A |  |
| LRRC8C | SLC35E2 |  |
| ADCY2 | NADK |  |
| ZC3HAV1 | GNB1 |  |
| SLC25A26 | CALML6 |  |
| POTEE | TMEM52 |  |
| FAM58A | CFAP74 |  |
| KLHL25 | PRKCZ |  |
| SP9 | FAAP20 |  |
| INTS1 | SKI |  |
| TMED1 | MORN1 |  |
| AP5M1 | RER1 |  |
| TMEM185B | PEX10 |  |
| DPYSL5 | PLCH2 |  |
| MYO1A | TNFRSF14 |  |
| ZC3H8 | FAM213B |  |
| NMT2 | MMEL1 |  |
| FBRS | TTC34 |  |
| JMJD4 | ACTRT2 |  |
| MICALL1 | ARHGEF16 |  |
| SLC41A2 | MEGF6 |  |
| FGD5 | TPRG1L |  |
| C17orf96 | TP73 |  |
| APOBEC4 | CCDC27 |  |
| ORAI2 | LRRC47 |  |
| NPLOC4 | CEP104 |  |
| CEP350 | C1orf174 |  |
| GNG4 | AJAP1 |  |
| PPARGC1B | NPHP4 |  |
| ZNF440 | KCNAB2 |  |
| SNX30 | RNF207 |  |
| EXOC7 | ICMT |  |
| FANCA | ACOT7 |  |

| ATRX | HES2 |  |
| --- | --- | --- |
| KLF16 | ESPN |  |
| WNT2B | TNFRSF25 |  |
| CCL25 | PLEKHG5 |  |
| SLC16A14 | NOL9 |  |
| ADAM22 | TAS1R1 |  |
| ITGA2 | ZBTB48 |  |
| TM9SF4 | KLHL21 |  |
| PNP | PHF13 |  |
| CHMP3 | CAMTA1 |  |
| ADAMTS4 | VAMP3 |  |
| MIB1 | PER3 |  |
| ARID3A | UTS2 |  |
| CNNM1 | TNFRSF9 |  |
| MRPS25 | PARK7 |  |
| AC010327.2 | ERRFI1 |  |
| GJA5 | SLC45A1 |  |
| BTBD11 | RERE |  |
| FAM21B | ENO1 |  |
| AL031666.2 | CA6 |  |
| ZNF84 | SLC2A7 |  |
| EPAS1 | SLC2A5 |  |
| SLC22A12 | GPR157 |  |
| CDKN2B | H6PD |  |
| HSD17B12 | SPSB1 |  |
| VPS8 | TMEM201 |  |
| CASP9 | PIK3CD |  |
| SCUBE1 | CLSTN1 |  |
| C11orf88 | CTNNBIP1 |  |
| SYNGAP1 | LZIC |  |
| STK16 | NMNAT1 |  |
| TMPRSS12 | UBE4B |  |
| SEC14L2 | KIF1B |  |
| TMED7 | PGD |  |
| SORBS3 | CENPS-CORT |  |
| POU2F2 | CORT |  |
| SCFD2 | DFFA |  |
| RNF103-CHMP3 | PEX14 |  |
| C11orf87 | CASZ1 |  |
| COLQ | C1orf127 |  |
| XKR7 | TARDBP |  |
| TP53INP2 | MASP2 |  |
| PUM1 | EXOSC10 |  |
| C5 | MTOR |  |
| FAM21A | ANGPTL7 |  |
| FAM21C | UBIAD1 |  |
| HS3ST1 | DISP3 |  |
| BTN2A2 | FBXO2 |  |
| EFHC1 | FBXO44 |  |
| GNPDA2 | FBXO6 |  |
| HOXD4 | MAD2L2 |  |
| PRKCE | AGTRAP |  |
| CDC42BPB | MTHFR |  |

| VAV3 | CLCN6 |  |
| --- | --- | --- |
| TMEM203 | NPPA |  |
| DNALI1 | NPPB |  |
| KIAA0895L | KIAA2013 |  |
| POFUT1 | PLOD1 |  |
| NADK | MFN2 |  |
| PPP1R12B | MIIP |  |
| LAIR1 | TNFRSF8 |  |
| FADS6 | TNFRSF1B |  |
| EP300 | VPS13D |  |
| RP11-625H11.1 | DHRS3 |  |
| RBM20 | AADACL3 |  |
| ANKRD50 | PRAMEF12 |  |
| FAM163A | PRAMEF1 |  |
| NLGN2 | PRAMEF11 |  |
| LIN28B | HNRNPCL1 |  |
| PEBP1 | PRAMEF2 |  |
| HAO2 | PRAMEF4 |  |
| DIRAS2 | PRAMEF10 |  |
| POTEF | PRAMEF7 |  |
| RCAN3 | PRAMEF6 |  |
| TRIM72 | PRAMEF27 |  |
| SUB1 | HNRNPCL3 |  |
| ILDR2 | PRAMEF25 |  |
| FOXC2 | HNRNPCL2 |  |
| ULBP3 | PRAMEF26 |  |
| CBLB | PRAMEF9 |  |
| SMS | PRAMEF13 |  |
| RAB11FIP3 | PRAMEF18 |  |
| TMX2 | PRAMEF5 |  |
| MMP2 | PRAMEF8 |  |
| PLEKHG4 | PRAMEF33 |  |
| SLC7A14 | PRAMEF15 |  |
| PYGB | PRAMEF14 |  |
| ERI2 | PRAMEF19 |  |
| SLC33A1 | PRAMEF17 |  |
| SIRT5 | PRAMEF20 |  |
| SMCR8 | LRRC38 |  |
| MESDC2 | PDPN |  |
| TCF7 | PRDM2 |  |
| MED28 | KAZN |  |
| FAM196B | TMEM51 |  |
| ZNF546 | FHAD1 |  |
| UBR2 | EFHD2 |  |
| PABPN1 | CTRC |  |
| HS3ST2 | CELA2A |  |
| RNF187 | CELA2B |  |
| FAM171A1 | CASP9 |  |
| GRPR | DNAJC16 |  |
| TGFB3 | AGMAT |  |
| HOXB5 | PLEKHM2 |  |
| FZD1 | SLC25A34 |  |
| TMEM63A | FBLIM1 |  |

| TP73 | SPEN |  |
| --- | --- | --- |
| MEX3A | ZBTB17 |  |
| UBE2D3 | ING2 |  |
| FOXK1 | RWDD4 |  |
| CLPB | TRAPPC11 |  |
| ZADH2 | STOX2 |  |
| SIT1 | ENPP6 |  |
| MYH9 | IRF2 |  |
| MBD3 | CASP3 |  |
| APOOL | PRIMPOL |  |
| HIST1H2BJ | CENPU |  |
| AXL | ACSL1 |  |
| ZIC4 | HELT |  |
| SGSM1 | SLC25A4 |  |
| PLXNA2 | CFAP97 |  |
| EVC | SNX25 |  |
| PITPNA | LRP2BP |  |
| MCFD2 | ANKRD37 |  |
| MGAT5 | UFSP2 |  |
| ABI3 | CCDC110 |  |
| B4GALT4 | PDLIM3 |  |
| AP5B1 | SORBS2 |  |
| COL4A1 | TLR3 |  |
| WDR13 | FAM149A |  |
| CSNK1G2 | CYP4V2 |  |
| ALPK3 | KLKB1 |  |
| ZSWIM4 | F11 |  |
| GPR135 | FAT1 |  |
| RPL10A | ZFP42 |  |
| CALR | TRIML2 |  |
| RRP1B | TRIML1 |  |
| UBE2H | FRG1 |  |
| TEX22 | FRG2 |  |
| PDHA1 | PLEKHG4B |  |
| HEYL | LRRC14B |  |
| GALNT13 | SDHA |  |
| POTEJ | PDCD6 |  |
| RNF41 | EXOC3 |  |
| N4BP3 | SLC9A3 |  |
| FZD7 | CEP72 |  |
| SZRD1 | ZDHHC11B |  |
| MARK4 | ZDHHC11 |  |
| HIST1H2BO | BRD9 |  |
| DEPDC1 | NKD2 |  |
| DGKI | SLC12A7 |  |
| SHROOM4 | SLC6A19 |  |
| PHIP | TERT |  |
| ZNF641 | CLPTM1L |  |
| VASH1 | SLC6A3 |  |
| SEMA6C | LPCAT1 |  |
| SYT1 | MRPL36 |  |
| GPR63 | IRX4 |  |
| FAM109A | IRX2 |  |

| UNC45A | C5orf38 |  |  |
| --- | --- | --- | --- |
| APOLD1 | IRX1 |  |  |
| DLEC1 | ADAMTS16 |  |  |
| ARMC10 | ICE1 |  |  |
| PLXDC2 | MED10 |  |  |
| AL590822.2 | UBE2QL1 |  |  |
| GGCX | NSUN2 |  |  |
| PLS1 | SRD5A1 |  |  |
| BCAT1 | PAPD7 |  |  |
| AL354993.1 | ADCY2 |  |  |
| TRAK2 | FASTKD3 |  |  |
| NRG2 | MTRR |  |  |
| ETV3L | SEMA5A |  |  |
| FAM111B | TAS2R1 |  |  |
| NUMBL | CCT5 |  |  |
| GAS7 | CMBL |  |  |
| FGD4 |  | 6-Mar |  |
| POLE | ROPN1L |  |  |
| KCNJ5 | ANKRD33B |  |  |
| MYL9 | DAP |  |  |
| TMEM86A | CTNND2 |  |  |
| PAX9 | DNAH5 |  |  |
| ATF2 | TRIO |  |  |
| MRPS18A | FAM105A |  |  |
| SPTB | OTULIN |  |  |
| GLYR1 | ANKH |  |  |
| ATP11A | FBXL7 |  |  |
| ZNFX1 | ZNF622 |  |  |
| CDK20 | RETREG1 |  |  |
| ZDHHC3 | MYO10 |  |  |
| SCUBE3 | CDH18 |  |  |
| WDR41 | CDH12 |  |  |
| ATG14 | PRDM9 |  |  |
| RANBP17 | CDH10 |  |  |
| DDX19B | CDH6 |  |  |
| C2CD2L | DROSHA |  |  |
| SCRN3 | C5orf22 |  |  |
| GRIN2B | PDZD2 |  |  |
| ADD2 | GOLPH3 |  |  |
| C6orf132 | MTMR12 |  |  |
| C10orf2 | SUB1 |  |  |
| CECR1 | NPR3 |  |  |
| LRRC18 | TARS |  |  |
| AK4 | ADAMTS12 |  |  |
| C1orf210 | RXFP3 |  |  |
| VSIG10L | SLC45A2 |  |  |
| XRCC2 | AMACR |  |  |
| A1CF | C1QTNF3 |  |  |
| ITPK1 | RAI14 |  |  |
| SEMA5A | RAD1 |  |  |
| N4BP1 | DNAJC21 |  |  |
| C5orf63 | AGXT2 |  |  |
| GPR180 | PRLR |  |  |

| TCF21 | SPEF2 |  |
| --- | --- | --- |
| PLB1 | IL7R |  |
| BRPF3 | CAPSL |  |
| ATXN2L | UGT3A1 |  |
| ADAT2 | UGT3A2 |  |
| MCTP2 | LMBRD2 |  |
| MAOA | SKP2 |  |
| PCBD1 | NADK2 |  |
| SLFN5 | RANBP3L |  |
| SLC25A37 | SLC1A3 |  |
| PTPLAD2 | NIPBL |  |
| BTF3L4 | CPLANE1 |  |
| EHD2 | NUP155 |  |
| KIAA1045 | GDNF |  |
| BICC1 | EGFLAM |  |
| ZNF587B | LIFR |  |
| IGFN1 | OSMR |  |
| ADAMTS14 | RICTOR |  |
| BAIAP3 | FYB1 |  |
| TBC1D5 | C9 |  |
| AMT | DAB2 |  |
| KIAA1551 | PTGER4 |  |
| KIAA1958 | TTC33 |  |
| CUL5 | PRKAA1 |  |
| MLPH | CARD6 |  |
| GPN1 | C7 |  |
| UBE4B | MROH2B |  |
| STS | C6 |  |
| CALCR | PLCXD3 |  |
| PODXL | OXCT1 |  |
| RFX6 | C5orf51 |  |
| KIAA1522 | FBXO4 |  |
| STRIP2 | GHR |  |
| KCNC3 | SELENOP |  |
| ZNF324 | ANXA2R |  |
| RELA | ZNF131 |  |
| ZSWIM6 | NIM1K |  |
| LRRC28 | HMGCS1 |  |
| TTC30A | CCL28 |  |
| HSPG2 | TMEM267 |  |
| TIAF1 | NNT |  |
| CBX5 | HCN1 |  |
| ATP1B2 | EMB |  |
| COL27A1 | PARP8 |  |
| LRRTM3 | ISL1 |  |
| TUBB4A | ITGA2 |  |
| SPDYE3 | MOCS2 |  |
| DNMBP | SNX18 |  |
| PARD3B | CDC20B |  |
| SPECC1L | GPX8 |  |
| PTCHD4 | MCIDAS |  |
| DCAF12L1 | CCNO |  |
| SFT2D2 | PLPP1 |  |

| ICOSLG | SLC38A9 |  |
| --- | --- | --- |
| GDF5OS | DDX4 |  |
| WIPF2 | TSTD2 |  |
| QKI | NCBP1 |  |
| TBC1D12 | FOXE1 |  |
| XPNPEP3 | TRMO |  |
| ZMAT3 | NANS |  |
| MCM8 | TRIM14 |  |
| CMIP | CORO2A |  |
| OAS2 | TBC1D2 |  |
| S1PR5 | GABBR2 |  |
| SDK2 | ANKS6 |  |
| PPP4R1L | COL15A1 |  |
| BRD2 | TGFBR1 |  |
| SAMD5 | ALG2 |  |
| GSTM5 | NR4A3 |  |
| NUDT16 | STX17 |  |
| PRR13 | TEX10 |  |
| ZNF561 | MSANTD3 |  |
| CTD-2545M3.6 | TMEFF1 |  |
| GRWD1 | CAVIN4 |  |
| SEMA4G | PLPPR1 |  |
| NHLH1 | BAAT |  |
| ZNF208 | ZNF189 |  |
| UQCC1 | ALDOB |  |
| IL22RA2 | TMEM246 |  |
| FAM153A | RNF20 |  |
| STAT1 | GRIN3A |  |
| MRGBP | PPP3R2 |  |
| SDK1 | SMC2 |  |
| BTBD9 | OR13C4 |  |
| DUSP2 | OR13C8 |  |
| RLIM | OR13D1 |  |
| MXI1 | NIPSNAP3A |  |
| STOX2 | NIPSNAP3B |  |
| INPP4B | ABCA1 |  |
| HAUS8 | SLC44A1 |  |
| SLC38A7 | FSD1L |  |
| YTHDC1 | FKTN |  |
| ENY2 | TMEM38B |  |
| ZNF574 | RAD23B |  |
| CEP104 | KLF4 |  |
| KBTBD11 | ACTL7B |  |
| STXBP5L | ELP1 |  |
| WFIKKN2 | CTNNAL1 |  |
| OR1L8 | TMEM245 |  |
| C2orf48 | FRRS1L |  |
| ZSCAN29 | EPB41L4B |  |
| WDR82 | PTPN3 |  |
| PDPK1 | PALM2 |  |
| TIPRL | PALM2-AKAP2 |  |
| RANGAP1 | AKAP2 |  |
| ILF3 | C9orf152 |  |

| ATG13 | TXNDC8 |  |
| --- | --- | --- |
| ANKRD33B | SVEP1 |  |
| HEMK1 | MUSK |  |
| GOSR1 | LPAR1 |  |
| SPAG6 | ZNF483 |  |
| PSEN1 | PTGR1 |  |
| SMIM13 | DNAJC25-GNG10 |  |
| LANCL2 | DNAJC25 |  |
| DDIT4 | C9orf84 |  |
| DTX3 | UGCG |  |
| RASA4B | SUSD1 |  |
| WDR52 | PTBP3 |  |
| HECTD4 | KIAA1958 |  |
| MAT1A | INIP |  |
| AOC3 | SNX30 |  |
| TTC17 | SLC46A2 |  |
| SLFN12 | ZNF883 |  |
| FADS1 | ZFP37 |  |
| HMX1 | SLC31A2 |  |
| GABRQ | FKBP15 |  |
| DCAF4L1 | SLC31A1 |  |
| PTAFR | PRPF4 |  |
| SH3BP1 | RNF183 |  |
| B3GAT1 | WDR31 |  |
| CACHD1 | BSPRY |  |
| SYK | ALAD |  |
| TSHZ2 | POLE3 |  |
| LYSMD3 | C9orf43 |  |
| BCL2L13 | RGS3 |  |
| STX6 | ZNF618 |  |
| PIP4K2B | KIF12 |  |
| TRAPPC2P1 | COL27A1 |  |
| NRXN2 | ORM1 |  |
| ARHGAP36 | ORM2 |  |
| SLC25A16 | AKNA |  |
| ARNT2 | WHRN |  |
| SCARB1 | ATP6V1G1 |  |
| PCDH20 | TEX53 |  |
| FOXL1 | TEX48 |  |
| DAK | TNFSF15 |  |
| SMAGP | TNFSF8 |  |
| MSRB3 | TNC |  |
| RNF152 | PAPPA |  |
| C17orf85 | ASTN2 |  |
| ABL2 | TLR4 |  |
| HGF | BRINP1 |  |
| ELFN2 | CDK5RAP2 |  |
| CRCP | MEGF9 |  |
| PROCA1 | FBXW2 |  |
| MAPK3 | PSMD5 |  |
| CAMKMT | PHF19 |  |
| GSPT1 | TRAF1 |  |
| RGMA | C5 |  |

| FARSB | CNTRL |  |
| --- | --- | --- |
| DHODH | RAB14 |  |
| GFPT1 | GSN |  |
| SERTAD2 | STOM |  |
| NHLRC2 | DAB2IP |  |
| RBBP5 | TTLL11 |  |
| ARHGEF11 | LHX6 |  |
| ZNF500 | RBM18 |  |
| TRPM2 | MRRF |  |
| USH1G | PTGS1 |  |
| MAP1A | OR1J2 |  |
| ZNF704 | OR1J4 |  |
| AHCYL2 | OR1N1 |  |
| ARAP1 | OR1N2 |  |
| POGZ | OR1L8 |  |
| PSD4 | OR1Q1 |  |
| SLC1A7 | OR1B1 |  |
| TBCK | OR1L3 |  |
| HNF4A | OR1L4 |  |
| FAM73B | OR1L6 |  |
| STX18 | OR5C1 |  |
| ADAMTS1 | OR1K1 |  |
| RP11-383H13.1 | PDCL |  |
| SNTB2 | RC3H2 |  |
| MTL5 | ZBTB6 |  |
| IBA57 | ZBTB26 |  |
| MCIDAS | STRBP |  |
| TRIM45 | CRB2 |  |
| PARP8 | DENND1A |  |
| LCOR | PSMB7 |  |
| PARVA | ADGRD2 |  |
| PEX5L | NR5A1 |  |
| C9orf139 | NR6A1 |  |
| PSCA | OLFML2A |  |
| XXYLT1 | WDR38 |  |
| ARHGAP35 | ARPC5L |  |
| SP1 | GOLGA1 |  |
| MEX3B | SCAI |  |
| RP11-1055B8.7 | PPP6C |  |
| FAM83G | RABEPK |  |
| CYLD | HSPA5 |  |
| HIST3H2BB | GAPVD1 |  |
| BHLHE41 | MAPKAP1 |  |
| INPP5K | PBX3 |  |
| NEGR1 | LMX1B |  |
| AMOT | ZBTB43 |  |
| CYP2W1 | ZBTB34 |  |
| PHC1 | RALGPS1 |  |
| EPB42 | ANGPTL2 |  |
| CTNNBIP1 | GARNL3 |  |
| PIK3R2 | SLC2A8 |  |
| TRIP12 | LRSAM1 |  |
| LHFPL4 | FAM129B |  |

| CCDC88C | CFAP157 |  |
| --- | --- | --- |
| FOSL2 | PTRH1 |  |
| GDAP2 | TTC16 |  |
| PGR | TOR2A |  |
| CERK | SH2D3C |  |
| CCDC77 | CDK9 |  |
| MTA3 | FPGS |  |
| ITPRIPL2 | ENG |  |
| TMEM44 | AK1 |  |
| USP46 | ST6GALNAC6 |  |
| TRAPPC2L | CLPTM1 |  |
| ADAMTSL2 | CLASRP |  |
| UNC93B1 | ZNF296 |  |
| CYP2B6 | GEMIN7 |  |
| AQP10 | TRAPPC6A |  |
| SORCS1 | BLOC1S3 |  |
| CD58 | MARK4 |  |
| PLXNB1 | CKM |  |
| ARFGEF2 | KLC3 |  |
| POLM | ERCC2 |  |
| ALG6 | PPP1R13L |  |
| ASCC1 | CD3EAP |  |
| TXNIP | ERCC1 |  |
| BTD | FOSB |  |
| ST6GAL1 | RTN2 |  |
| SLC7A1 | VASP |  |
| PHF6 | OPA3 |  |
| RNF44 | GPR4 |  |
| TFAP2B | EML2 |  |
| PLA2R1 | GIPR |  |
| FAM69B | SNRPD2 |  |
| C6 | QPCTL |  |
| MAPK8 | FBXO46 |  |
| FANCC | BHMG1 |  |
| NABP1 | SIX5 |  |
| KRR1 | DMPK |  |
| GNG7 | DMWD |  |
| POGK | RSPH6A |  |
| CDK15 | SYMPK |  |
| SIX6 | FOXA3 |  |
| TBC1D10B | NOVA2 |  |
| SLC36A1 | CCDC61 |  |
| TSPAN11 | PGLYRP1 |  |
| XPNPEP1 | IGFL4 |  |
| SCYL3 | HIF3A |  |
| ICMT | PPP5C |  |
| MTMR12 | CCDC8 |  |
| TMEM33 | PNMA8A |  |
| SRCAP | PPP5D1 |  |
| UBE4A | PNMA8B |  |
| RIMKLA | CALM3 |  |
| FAM122A | PTGIR |  |
| DCPS | GNG8 |  |

| SLC6A19 | DACT3 |  |
| --- | --- | --- |
| PLAGL2 | PRKD2 |  |
| ANAPC16 | STRN4 |  |
| CALCOCO1 | FKRP |  |
| GALNT15 | SLC1A5 |  |
| CDT1 | AP2S1 |  |
| LHX4 | ARHGAP35 |  |
| TTC13 | NPAS1 |  |
| RCOR1 | ZC3H4 |  |
| SLMAP | SAE1 |  |
| SEC14L1 | BBC3 |  |
| MXD1 | INAFM1 |  |
| RPL28 | C5AR1 |  |
| PTCD1 | C5AR2 |  |
| RPL4 | DHX34 |  |
| SEPT8 | MEIS3 |  |
| TMEM65 | SLC8A2 |  |
| LRRC3DN | KPTN |  |
| N6AMT1 | NAPA |  |
| CHRND | ZNF541 |  |
| NOL10 | BICRA |  |
| WSB1 | EHD2 |  |
| MAPK1IP1L | NOP53 |  |
| ZNF589 | SELENOW |  |
| RFX1 | TPRX1 |  |
| PLIN4 | CRX |  |
| GPR176 | CABP5 |  |
| NUPL1 | PLA2G4C |  |
| CHST2 | LIG1 |  |
| ERI1 | ZSWIM9 |  |
| OSBPL10 | CARD8 |  |
| RBM18 | ZNF114 |  |
| TMEM135 | CCDC114 |  |
| CSF1 | EMP3 |  |
| CASP14 | TMEM143 |  |
| RASA4 | SYNGR4 |  |
| ARHGEF7 | KDELR1 |  |
| C16orf72 | GRIN2D |  |
| LCN10 | GRWD1 |  |
| MRRF | KCNJ14 |  |
| TMEM120B | CYTH2 |  |
| SLC43A2 | SULT2B1 |  |
| C1orf35 | FAM83E |  |
| CLDN5 | SPACA4 |  |
| FUT9 | SPHK2 |  |
| CD5 | DBP |  |
| PIGM | CA11 |  |
| PIM2 | NTN5 |  |
| ZNF230 | FUT2 |  |
| TIMM50 | MAMSTR |  |
| COL18A1 | RASIP1 |  |
| SNX33 | IZUMO1 |  |
| WDR48 | FUT1 |  |

| GIGYF2 | FGF21 |  |
| --- | --- | --- |
| PTBP2 | BCAT2 |  |
| ZNF708 | HSD17B14 |  |
| PNPLA4 | PLEKHA4 |  |
| M6PR | TULP2 |  |
| SOX8 | NUCB1 |  |
| NR3C2 | DHDH |  |
| RNF150 | BAX |  |
| TYW5 | FTL |  |
| LAYN | GYS1 |  |
| IREB2 | RUVBL2 |  |
| TLE3 | LHB |  |
| NOL6 | CGB3 |  |
| LCORL | CGB2 |  |
| ARSK | CGB1 |  |
| XPO5 | CGB5 |  |
| TNFRSF25 | CGB8 |  |
| IL6ST | CGB7 |  |
| SULF2 | NTF4 |  |
| CD93 | KCNA7 |  |
| CHD5 | C19orf73 |  |
| RGS17 | PPFIA3 |  |
| MAD2L1 | HRC |  |
| TPD52 | TRPM4 |  |
| MFF | SLC6A16 |  |
| SMLR1 | CD37 |  |
| CX3CL1 | TEAD2 |  |
| DTD2 | DKKL1 |  |
| HTRA2 | CCDC155 |  |
| POLL | GFY |  |
| SP2 | SLC17A7 |  |
| DCLRE1B | PIH1D1 |  |
| DBNL | ALDH16A1 |  |
| CRIPT | FLT3LG |  |
| RBM27 | RPL13A |  |
| RHOBTB3 | RCN3 |  |
| ADAMTS13 | PRRG2 |  |
| ING5 | PRR12 |  |
| C16orf87 | RRAS |  |
| NAPG | SCAF1 |  |
| C4orf46 | IRF3 |  |
| EBF1 | BCL2L12 |  |
| TBC1D16 | PRMT1 |  |
| ACADSB | ADM5 |  |
| NAIF1 | CPT1C |  |
| SGCD | TSKS |  |
| MME | AP2A1 |  |
| BSND | FUZ |  |
| SKI | MED25 |  |
| PPP1R1A | PTOV1 |  |
| CCL1 | AKT1S1 |  |
| C5orf15 | TBC1D17 |  |
| PQLC2 | IL4I1 |  |

| INSR | NUP62 |  |
| --- | --- | --- |
| SH3PXD2B | ATF5 |  |
| PPP1CB | SIGLEC11 |  |
| LYRM2 | VRK3 |  |
| ARIH1 | ZNF473 |  |
| TBRG4 | IZUMO2 |  |
| C1orf21 | MYH14 |  |
| CINP | KCNC3 |  |
| HS3ST3A1 | NAPSA |  |
| MSI2 | NR1H2 |  |
| ITGB8 | POLD1 |  |
| CAMK2D | SPIB |  |
| RAD1 | MYBPC2 |  |
| GRPEL2 | FAM71E1 |  |
| WNT9A | JOSD2 |  |
| YPEL2 | ASPDH |  |
| TRIM25 | LRRC4B |  |
| SLC25A44 | SYT3 |  |
| WTIP | C19orf81 |  |
| GID8 | SHANK1 |  |
| LUZP1 | CLEC11A |  |
| ESF1 | GPR32 |  |
| PDK2 | ACP4 |  |
| NDST4 | C19orf48 |  |
| EGFR | KLK1 |  |
| DDX31 | KLK15 |  |
| THSD4 | KLK3 |  |
| ANKRD9 | KLK5 |  |
| KIN | KLK6 |  |
| TUBB | KLK7 |  |
| AGAP1 | KLK9 |  |
| TOMM22 | KLK10 |  |
| KDELR1 | KLK11 |  |
| ZKSCAN4 | KLK13 |  |
| ZNF211 | KLK14 |  |
| TENM4 | SIGLEC9 |  |
| DYNLL2 | SIGLEC7 |  |
| PKIA | CD33 |  |
| DBT | SIGLECL1 |  |
| HEBP2 | IGLON5 |  |
| TMEM242 | CLDND2 |  |
| SYNPO | NKG7 |  |
| CENPL | LIM2 |  |
| TFDP2 | C19orf84 |  |
| MBL2 | SIGLEC10 |  |
| C11orf48 | SIGLEC8 |  |
| C11orf54 | CEACAM18 |  |
| MED22 | SIGLEC12 |  |
| KCTD12 | SIGLEC6 |  |
| PDGFD | SIGLEC5 |  |
| ANKRD40 | SCT |  |
| TRAF3 | DRD4 |  |
| GZF1 | DEAF1 |  |

| FGF5 | TMEM80 |  |
| --- | --- | --- |
| NSL1 | EPS8L2 |  |
| SPATA17 | TALDO1 |  |
| SAMHD1 | GATD1 |  |
| GPR26 | SLC25A22 |  |
| KIAA0753 | PIDD1 |  |
| FAM105A | RPLP2 |  |
| SFT2D3 | PNPLA2 |  |
| FMNL3 | CRACR2B |  |
| TMED8 | CD151 |  |
| TIMM9 | POLR2L |  |
| PPM1L | TSPAN4 |  |
| AGO3 | CHID1 |  |
| PPP5C | AP2A2 |  |
| NINJ1 | MUC6 |  |
| TM4SF1 | MUC5B |  |
| OTUD7B | TOLLIP |  |
| BCL2L11 | BRSK2 |  |
| TMEM239 | MOB2 |  |
| BCL11B | DUSP8 |  |
| LRRC8A | KRTAP5-2 |  |
| SLC25A23 | KRTAP5-3 |  |
| AC174470.1 | KRTAP5-4 |  |
| PXN | KRTAP5-6 |  |
| C18orf21 | IFITM10 |  |
| PRRC2B | SYT8 |  |
| C21orf58 | TNNI2 |  |
| ZNF189 | LSP1 |  |
| PYGO2 | TNNT3 |  |
| SLC19A1 | MRPL23 |  |
| TMEM101 | IGF2 |  |
| TSPAN3 | TH |  |
| NKD1 | ASCL2 |  |
| RHPN1 | C11orf21 |  |
| MYSM1 | TSPAN32 |  |
| RPL32 | CD81 |  |
| MRPS30 | TRPM5 |  |
| ADAMTSL5 | KCNQ1 |  |
| CD109 | CDKN1C |  |
| VWA5A | SLC22A18AS |  |
| PAX3 | SLC22A18 |  |
| NYAP1 | CARS |  |
| ZMIZ2 | OSBPL5 |  |
| SLC6A17 | MRGPRG |  |
| MEAF6 | MRGPRE |  |
| MLXIP | ZNF195 |  |
| RAD18 | ART5 |  |
| COMT | ART1 |  |
| TMED10 | CHRNA10 |  |
| FECH | NUP98 |  |
| SNRPD1 | PGAP2 |  |
| TXLNA | RHOG |  |
| MARCH6 | STIM1 |  |

| METRN | OR52B4 |  |
| --- | --- | --- |
| SMARCC1 | TRIM21 |  |
| GAB1 | OR52K2 |  |
| ITFG1 | OR52K1 |  |
| PLEKHA3 | OR52M1 |  |
| EIF4EBP3 | OR52I2 |  |
| LAD1 | OR52I1 |  |
| GPR20 | TRIM68 |  |
| NDUFAF5 | OR51D1 |  |
| LMTK2 | OR51E1 |  |
| ZNF611 | OR51E2 |  |
| MAU2 | OR51F1 |  |
| INTS3 | OR52R1 |  |
| PHF19 | OR51T1 |  |
| AJAP1 | OR51A7 |  |
| RRM2 | OR51G2 |  |
| RAB43 | OR51G1 |  |
| NTRK2 | OR51A4 |  |
| MAP7 | OR51A2 |  |
| MTDH | MMP26 |  |
| KLF10 | OR51L1 |  |
| CNKSR3 | OR52E1 |  |
| EXOC4 | OR52A1 |  |
| NUDCD1 | OR51V1 |  |
| MOB3B | HBG1 |  |
| MSH6 | HBG2 |  |
| RALY | HBE1 |  |
| DGKE | OR51B4 |  |
| SLC9A4 | OR51B5 |  |
| JPH4 | OR51B6 |  |
| SHROOM3 | OR51M1 |  |
| FOXI2 | OR51Q1 |  |
| VWA3A | OR51I1 |  |
| BCL9L | OR51I2 |  |
| ZNF354B | OR52D1 |  |
| DDN | UBQLN3 |  |
| AKT3 | UBQLNL |  |
| NEK9 | OR52B6 |  |
| TSC1 | TRIM6 |  |
| PDGFA | TRIM34 |  |
| COX15 | TRIM5 |  |
| C16orf45 | TRIM22 |  |
| SPIB | OR52N4 |  |
| KIAA1715 | OR52N5 |  |
| TMEM178B | OR52N2 |  |
| FAM9C | OR52E6 |  |
| CTNNA3 | OR52E8 |  |
| MEGF8 | OR52E5 |  |
| OTOF | OR56A3 |  |
| DTX4 | OR56A5 |  |
| CACNA1E | OR52L1 |  |
| ABLIM2 | OR56A4 |  |
| PREX1 | OR56A1 |  |

| LRRTM2 | OR56B4 |  |
| --- | --- | --- |
| PRX | OR52W1 |  |
| CSMD2 | C11orf42 |  |
| RAX2 | FAM160A2 |  |
| SLX4IP | CNGA4 |  |
| ZNF783 | CCKBR |  |
| KREMEN1 | CAVIN3 |  |
| KIAA1199 | SMPD1 |  |
| LRRC4B | APBB1 |  |
| HS6ST1 | HPX |  |
| SMG6 | TRIM3 |  |
| ZNF646 | TIMM10B |  |
| PLD5 | DNHD1 |  |
| CACNA1C | RRP8 |  |
| MSN | ILK |  |
| ZC3H3 | TAF10 |  |
| ADAMTS15 | TPP1 |  |
| TMEM132B | DCHS1 |  |
| GALNT6 | MRPL17 |  |
| FAM19A5 | OR2AG2 |  |
| IRX4 | OR2AG1 |  |
| NKX6-1 | OR6A2 |  |
| FOXO3 | OR10A5 |  |
| TRPM3 | OR2D3 |  |
| PCYT2 | ZNF215 |  |
| GPSM1 | ZNF214 |  |
| KIF13B | RBMXL2 |  |
| HES2 | SYT9 |  |
| TBX15 | OLFML1 |  |
| TBC1D14 | PPFIBP2 |  |
| DZIP1L | CYB5R2 |  |
| NLRP1 | OVCH2 |  |
| WDR78 | OR5P3 |  |
| ESYT2 | OR10A6 |  |
| EPDR1 | OR10A3 |  |
| ZNF395 | NLRP10 |  |
| HSPA4L | EIF3F |  |
| BACH2 | TUB |  |
| PTCD2 | LMO1 |  |
| MLX | TRIM66 |  |
| WDR26 | RPL27A |  |
| GRAMD4 | ST5 |  |
| MESP1 | AKIP1 |  |
| HCFC2 | C11orf16 |  |
| RGS9BP | TMEM9B |  |
| NMT1 | NRIP3 |  |
| CLASP1 | SCUBE2 |  |
| UPF1 | DENND5A |  |
| SERPINA4 | TMEM41B |  |
| DDI2 | IPO7 |  |
| ZNF354C | ZNF143 |  |
| VPS13A | WEE1 |  |
| ARRB1 | SWAP70 |  |

| TRIM13 | SBF2 |  |
| --- | --- | --- |
| RHOQ | AMPD3 |  |
| SLC6A6 | MTRNR2L8 |  |
| MPP6 | RNF141 |  |
| FSTL3 | LYVE1 |  |
| CLMN | MRVI1 |  |
| ANKFY1 | CTR9 |  |
| SNX8 | ZBED5 |  |
| IER3IP1 | GALNT18 |  |
| RHBDL3 | CSNK2A3 |  |
| CREBZF | USP47 |  |
| GM2A | DKK3 |  |
| MECP2 | MICAL2 |  |
| RBMS3 | MICALCL |  |
| RPL18A | PARVA |  |
| KDM5A | TEAD1 |  |
| RAB30 | RASSF10 |  |
| NSUN5 | ARNTL |  |
| ZFYVE9 | SPON1 |  |
| SLC22A18AS | RRAS2 |  |
| EIF2S2 | COPB1 |  |
| OTUD5 | PSMA1 |  |
| USP36 | CYP2R1 |  |
| MPI | CALCA |  |
| RPL15 | CALCB |  |
| PIK3C3 | MTERF2 |  |
| C4orf29 | CRY1 |  |
| CDHR1 | BTBD11 |  |
| UACA | PWP1 |  |
| GJC1 | ASCL4 |  |
| FGFR1OP | WSCD2 |  |
| ANKIB1 | CMKLR1 |  |
| GFER | ISCU |  |
| ID4 | TMEM119 |  |
| MRPL45 | SELPLG |  |
| NDOR1 | CORO1C |  |
| MYO18A | SSH1 |  |
| MYO9A | DAO |  |
| PLEKHM3 | SVOP |  |
| EYA3 | USP30 |  |
| C1orf95 | ACACB |  |
| GPR144 | FOXN4 |  |
| ST13 | MYO1H |  |
| LIG3 | KCTD10 |  |
| CPM | UBE3B |  |
| PDS5A | MMAB |  |
| DZIP3 | MVK |  |
| GPR107 | FAM222A |  |
| LMLN | TRPV4 |  |
| MRTO4 | GLTP |  |
| TRA2B | TCHP |  |
| DGAT2 | GIT2 |  |
| ALG9 | IFT81 |  |

| GSTO2 | ATP2A2 |  |
| --- | --- | --- |
| POLG | ANAPC7 |  |
| WNT10B | ARPC3 |  |
| CLVS2 | FAM216A |  |
| PSMB2 | VPS29 |  |
| MYO1E | PPTC7 |  |
| TMEM170B | HVCN1 |  |
| CCDC153 | PPP1CC |  |
| STK25 | CCDC63 |  |
| UBXN7 | CUX2 |  |
| AP2B1 | SH2B3 |  |
| ZNF41 | ATXN2 |  |
| TXLNG | BRAP |  |
| SLC4A4 | ACAD10 |  |
| RFC5 | TMEM116 |  |
| MLLT6 | ERP29 |  |
| NRL | NAA25 |  |
| RAPGEF3 | TRAFD1 |  |
| UVSSA | HECTD4 |  |
| ADRBK2 | PTPN11 |  |
| AGTPBP1 | RPH3A |  |
| KLHL31 | OAS1 |  |
| CBX7 | OAS3 |  |
| PELO | OAS2 |  |
| HOXD11 | DTX1 |  |
| BRCC3 | RASAL1 |  |
| CA12 | CFAP73 |  |
| ENGASE | DDX54 |  |
| INTU | RITA1 |  |
| PGK1 | IQCD |  |
| TMEM11 | TPCN1 |  |
| METTL14 | SLC8B1 |  |
| DDX19A | PLBD2 |  |
| NFKBIA | SDS |  |
| FAM179A | SDSL |  |
| SLC44A1 | RBM19 |  |
| TTC39A | TBX5 |  |
| SEMA6A | TBX3 |  |
| SBNO1 | MED13L |  |
| HSP90B1 | MAP1LC3B2 |  |
| DHRS7 | C12orf49 |  |
| LRP2BP | RNFT2 |  |
| UBN2 | HRK |  |
| DDX11 | FBXW8 |  |
| EZH1 | NOS1 |  |
| DYRK2 | KSR2 |  |
| ZFPM1 | RFC5 |  |
| SLBP | WSB2 |  |
| RNF214 | VSIG10 |  |
| ZDHHC14 | PEBP1 |  |
| PPP2R1B | SUDS3 |  |
| MTG1 | SRRM4 |  |
| RP11-108K14.8 | HSPB8 |  |

| FUBP1 | TMEM233 |  |
| --- | --- | --- |
| CCBE1 | PRKAB1 |  |
| TRIM71 | CIT |  |
| GNL1 | BICDL1 |  |
| SUSD1 | RAB35 |  |
| SIGLEC14 | GCN1 |  |
| ASB6 | PXN |  |
| RNMT | SIRT4 |  |
| HGFAC | MSI1 |  |
| ERGIC2 | GATC |  |
| GK | SRSF9 |  |
| MUC3A | RNF10 |  |
| NFE2L3 | POP5 |  |
| POLR2C | CABP1 |  |
| EHD1 | MLEC |  |
| CASC10 | UNC119B |  |
| U2SURP | ACADS |  |
| ZNF703 | SPPL3 |  |
| ZNF334 | HNF1A |  |
| TUBGCP4 | C12orf43 |  |
| MMAB | OASL |  |
| C9orf85 | P2RX7 |  |
| SCIN | P2RX4 |  |
| PDPR | CAMKK2 |  |
| MLH3 | ANAPC5 |  |
| MAPKAPK5 | KDM2B |  |
| SUPT3H | TMEM120B |  |
| FKBP5 | RHOF |  |
| MKRN2 | SETD1B |  |
| ASB1 | HPD |  |
| P2RY2 | PSMD9 |  |
| PLAC8 | WDR66 |  |
| CASKIN1 | BCL7A |  |
| LRRC16A | MLXIP |  |
| MAN2B1 | LRRC43 |  |
| ZNF285 | B3GNT4 |  |
| IGF2R | DIABLO |  |
| ZNF326 | VPS33A |  |
| PHKG2 | CLIP1 |  |
| ANKHD1-EIF4EBP3 | ZCCHC8 |  |
| NAPA | RSRC2 |  |
| CHSY1 | KNTC1 |  |
| RBMS2 | HCAR2 |  |
| HECW2 | HCAR3 |  |
| ZDHHC9 | HCAR1 |  |
| STRIP1 | DENR |  |
| GPRIN3 | CCDC62 |  |
| ZDHHC5 | HIP1R |  |
| GRHPR | VPS37B |  |
| GNB1L | ABCB9 |  |
| NCAPH | OGFOD2 |  |
| LGALS8 | PITPNM2 |  |
| XRCC5 | MPHOSPH9 |  |

| AGPAT6 | C12orf65 |  |  |
| --- | --- | --- | --- |
| B4GALT7 | CDK2AP1 |  |  |
| MAPK8IP2 | SBNO1 |  |  |
| PSPH | KMT5A |  |  |
| LSAMP | RILPL2 |  |  |
| TRMT61A | SNRNP35 |  |  |
| METAP1D | RILPL1 |  |  |
| EFNA5 | TMED2 |  |  |
| VSIG10 | DDX55 |  |  |
| UBE2QL1 | EIF2B1 |  |  |
| FAM49B | GTF2H3 |  |  |
| PODNL1 | TCTN2 |  |  |
| RPS29 | ATP6V0A2 |  |  |
| MRPL19 | DNAH10 |  |  |
| FIBIN | CCDC92 |  |  |
| PLXNA1 | ZNF664-RFLNA |  |  |
| TIA1 | ZNF664 |  |  |
| CPPED1 | RFLNA |  |  |
| BDH1 | NCOR2 |  |  |
| MED20 | SCARB1 |  |  |
| ETV5 | DHX37 |  |  |
| NME6 | BRI3BP |  |  |
| GPKOW | AACS |  |  |
| DNM1L | TMEM132B |  |  |
| RBM23 | TMEM132C |  |  |
| MRPL42 | GLT1D1 |  |  |
| NR1H3 | TMEM132D |  |  |
| GPLD1 | FZD10 |  |  |
| ARSA | PIWIL1 |  |  |
| TRAF6 | RIMBP2 |  |  |
| SLC45A4 | STX2 |  |  |
| RMND5A | RAN |  |  |
| PDE7A | ADGRD1 |  |  |
| DEF6 | SFSWAP |  |  |
| LPHN3 | MMP17 |  |  |
| FKBP15 | EP400 |  |  |
| DGKH | COBL |  |  |
| RAPGEF5 | POM121L12 |  |  |
| TFEC | VSTM2A |  |  |
| SLC38A1 | EGFR |  |  |
| RND3 | LANCL2 |  |  |
| RAB27B | VOPP1 |  |  |
| SCN4B |  | 14-Sep |  |
| BCAS1 | ZNF713 |  |  |
| SLC12A1 | NIPSNAP2 |  |  |
| IKZF1 | PSPH |  |  |
| STAC | SUMF2 |  |  |
| KCNMB4 | PHKG1 |  |  |
| TEKT1 | CHCHD2 |  |  |
| COL8A2 | ZNF479 |  |  |
| SMAD7 | ZNF716 |  |  |
| CPEB1 | ZNF727 |  |  |
| ASAH2 | ZNF679 |  |  |

| MAP3K3 | ZNF736 |  |
| --- | --- | --- |
| MEF2D | ZNF680 |  |
| DCHS1 | ZNF107 |  |
| CTC-349C3.1 | ZNF273 |  |
| CCR5 | ERV3-1-ZNF117 |  |
| KSR1 | ZNF117 |  |
| PHF13 | ERV3-1 |  |
| MYLK | ZNF92 |  |
| CNTFR | VKORC1L1 |  |
| SLC4A1 | GUSB |  |
| IPO7 | TPST1 |  |
| TMEM200B | RABGEF1 |  |
| IQGAP3 | TMEM248 |  |
| GPR124 | TYW1 |  |
| CCDC8 | AUTS2 |  |
| PRKCB | GALNT17 |  |
| FASN | CALN1 |  |
| HIRA | TYW1B |  |
| ABCA13 | POM121 |  |
| HNF4G | TRIM74 |  |
| PRR23A | SPDYE11 |  |
| KMT2A | NSUN5 |  |
| NYNRIN | TRIM50 |  |
| QSOX2 | FZD9 |  |
| TUB | BAZ1B |  |
| PHF12 | BCL7B |  |
| ITGA9 | MLXIPL |  |
| SYNGR1 | VPS37D |  |
| ITK | BUD23 |  |
| RGL1 | STX1A |  |
| TMEM231 | ABHD11 |  |
| EDA | CLDN3 |  |
| ABTB2 | METTL27 |  |
| DNAJA3 | TMEM270 |  |
| KLHDC7A | ELN |  |
| MGAT5B | LIMK1 |  |
| GPR132 | EIF4H |  |
| ADAR | LAT2 |  |
| LRRC2 | RFC2 |  |
| DCLK3 | CLIP2 |  |
| KDM4B | GTF2IRD1 |  |
| PHF21B | GTF2IRD2 |  |
| CALHM1 | CASTOR2 |  |
| DST | RCC1L |  |
| ARRDC2 | GTF2IRD2B |  |
| HAS3 | TRIM73 |  |
| SYVN1 | POM121C |  |
| POU6F2 | SPDYE5 |  |
| CCDC144A | HIP1 |  |
| SLC17A4 | CCL26 |  |
| ADAMTS9 | RHBDD2 |  |
| NRP1 | POR |  |
| PTPRB | TMEM120A |  |

| TIGD5 | STYXL1 |  |
| --- | --- | --- |
| L3MBTL3 | MDH2 |  |
| UMPS | SRRM3 |  |
| PTGR2 | HSPB1 |  |
| MDGA1 | YWHAG |  |
| DERL3 | SSC4D |  |
| MRPS14 | ZP3 |  |
| ALG14 | DTX2 |  |
| PRRG1 | SPDYE16 |  |
| FLI1 | SPDYE17 |  |
| ZNF592 | CCDC146 |  |
| FUT10 | FGL2 |  |
| CCDC90B | GSAP |  |
| MICAL3 | PTPN12 |  |
| RPTOR | PHTF2 |  |
| TMEM98 | MAGI2 |  |
| TTLL12 | CD36 |  |
| AGGF1 | HGF |  |
| NKTR | CACNA2D1 |  |
| GPAM | PCLO |  |
| PHF20L1 | SEMA3E |  |
| TMEM108 | SEMA3A |  |
| PLEKHA8 | SEMA3D |  |
| ZNF518B | GRM3 |  |
| RICTOR | KIAA1324L |  |
| FAM227A | DMTF1 |  |
| CIT | TMEM243 |  |
| PLEKHO1 | CROT |  |
| ABCG8 | ABCB4 |  |
| MCOLN3 | ABCB1 |  |
| MNAT1 | RUNDC3B |  |
| SLC25A33 | SLC25A40 |  |
| ATP9A | DBF4 |  |
| ARIH2 | ADAM22 |  |
| CLCN4 | STEAP4 |  |
| CCDC85C | ZNF804B |  |
| ATP5A1 | TEX47 |  |
| JHDM1D | STEAP2 |  |
| COL4A4 | CFAP69 |  |
| FAM46A | GTPBP10 |  |
| TTL | CLDN12 |  |
| IKZF2 | CDK14 |  |
| TAOK1 | FZD1 |  |
| GRAMD1B | AKAP9 |  |
| POU6F1 | CYP51A1 |  |
| ALX1 | KRIT1 |  |
| PRDM16 | ANKIB1 |  |
| ABCF1 | GATAD1 |  |
| SUDS3 | ERVW-1 |  |
| SH2B3 | PEX1 |  |
| SMNDC1 | RBM48 |  |
| RPAP2 | FAM133B |  |
| VPS72 | CDK6 |  |

| MAP3K9 | SAMD9 |  |
| --- | --- | --- |
| SETBP1 | SAMD9L |  |
| RORA | VPS50 |  |
| CCNYL1 | CALCR |  |
| MUC13 | TFPI2 |  |
| TGS1 | COL1A2 |  |
| FLYWCH2 | CASD1 |  |
| GPR161 | SGCE |  |
| MTAP | PEG10 |  |
| CA5B | PPP1R9A |  |
| MAPK4 | PON1 |  |
| PTPRG | PON2 |  |
| PCYT1A | ASB4 |  |
| PRDM1 | DYNC1I1 |  |
| CABLES1 | DLX5 |  |
| MID2 | SDHAF3 |  |
| INPP4A | TAC1 |  |
| ERCC6L | ASNS |  |
| APEH | OCM2 |  |
| TOB2 | LMTK2 |  |
| SLC22A3 | BRI3 |  |
| SNED1 | BAIAP2L1 |  |
| KCNK1 | NPTX2 |  |
| DLC1 | TMEM130 |  |
| TMEM67 | TRRAP |  |
| DNMT3A | SMURF1 |  |
| SMIM12 | KPNA7 |  |
| PPP1R21 | ARPC1A |  |
| DYNC1LI2 | ARPC1B |  |
| CDX2 | PGAP1 |  |
| FAM49A | ANKRD44 |  |
| ASPSCR1 | SF3B1 |  |
| NAGS | COQ10B |  |
| TMEM167A | MIS12 |  |
| SVEP1 | NLRP1 |  |
| PRPF38B | WSCD1 |  |
| GREB1L | AIPL1 |  |
| MOB1A | PITPNM3 |  |
| COX7A2L | KIAA0753 |  |
| PDE10A | TXNDC17 |  |
| WDR37 | MED31 |  |
| ALDH5A1 | SLC13A5 |  |
| SYNJ2 | XAF1 |  |
| INSIG2 | FBXO39 |  |
| SMG1 | RNASEK |  |
| PTPN4 | BCL6B |  |
| RUSC2 | SLC16A13 |  |
| BBX | SLC16A11 |  |
| XPR1 | CLEC10A |  |
| SVIP | ASGR2 |  |
| ATP5F1 | ASGR1 |  |
| FXYD3 | DLG4 |  |
| CCDC80 | PHF23 |  |

| C3 | EHMT1 |  |
| --- | --- | --- |
| PMEPA1 | TUBB8 |  |
| ALDH3A2 | ZMYND11 |  |
| NOP9 | DIP2C |  |
| ZNF507 | PRR26 |  |
| CNBP | LARP4B |  |
| KIAA0141 | IDI2 |  |
| FAM96A | WDR37 |  |
| PCNX | ADARB2 |  |
| ITPA | PFKP |  |
| PHB2 | PITRM1 |  |
| STOML2 | KLF6 |  |
| POC1A | AKR1E2 |  |
| ATP6V0D1 | AKR1C1 |  |
| PPP3CC | AKR1C2 |  |
| MED6 | AKR1C3 |  |
| ANKS6 | AKR1C4 |  |
| TADA2A | UCN3 |  |
| ACVRL1 | TUBAL3 |  |
| ESYT3 | NET1 |  |
| CHFR | CALML5 |  |
| GATAD2B | ASB13 |  |
| NSD1 | FAM208B |  |
| PANX1 | ANKRD16 |  |
| SDHAF2 | FBH1 |  |
| TMBIM6 | IL15RA |  |
| SCAND3 | RBM17 |  |
| ABCG1 | PFKFB3 |  |
| DCAF17 | PRKCQ |  |
| TTF2 | ITIH5 |  |
| PPFIBP2 | ITIH2 |  |
| EXOSC7 | KIN |  |
| TRIM38 | TAF3 |  |
| SLC38A2 | CELF2 |  |
| RNASEH2B | USP6NL |  |
| STAMBPL1 | ECHDC3 |  |
| STAT6 | UPF2 |  |
| PDGFC | DHTKD1 |  |
| RBFA | SEC61A2 |  |
| WDR53 | NUDT5 |  |
| IL17RB | CDC123 |  |
| FUNDC2 | CAMK1D |  |
| CISD3 | CCDC3 |  |
| RBM15B | OPTN |  |
| USP31 | MCM10 |  |
| PLEKHG1 | UCMA |  |
| KDM5C | PHYH |  |
| DDHD1 | SEPHS1 |  |
| PPIE | BEND7 |  |
| IMP4 | FRMD4A |  |
| TMEM106B | FAM107B |  |
| GPX8 | HSPA14 |  |
| DGCR6L | SUV39H2 |  |

| PAQR3 | DCLRE1C |  |
| --- | --- | --- |
| ANKMY1 | ACBD7 |  |
| ENPEP | RPP38 |  |
| PPIP5K2 | NMT2 |  |
| TRPM7 | FAM171A1 |  |
| ECHDC2 | ITGA8 |  |
| FAM136A | PTER |  |
| MON2 | CUBN |  |
| TGFB2 | TRDMT1 |  |
| SLC2A11 | VIM |  |
| ITGA3 | ST8SIA6 |  |
| ZNF451 | HACD1 |  |
| CDS2 | STAM |  |
| CRELD1 | TMEM236 |  |
| EML4 | MRC1 |  |
| DHX36 | SLC39A12 |  |
| PSD3 | CACNB2 |  |
| GPBP1 | ARL5B |  |
| TRMT5 | MALRD1 |  |
| FRK | PLXDC2 |  |
| NDUFA4 | NEBL |  |
| CCDC149 | SKIDA1 |  |
| TMC6 | MLLT10 |  |
| ABCF3 | DNAJC1 |  |
| C10orf54 | EBLN1 |  |
| FAF1 | COMMD3 |  |
| PPP6C | SPAG6 |  |
| TPCN2 | PIP4K2A |  |
| POLR1E | ARMC3 |  |
| MTUS2 | C10orf67 |  |
| LMBRD1 | OTUD1 |  |
| PRKRIP1 | KIAA1217 |  |
| HIC2 | ARHGAP21 |  |
| GMPPB | PRTFDC1 |  |
| MBD2 | ENKUR |  |
| SPECC1 | THNSL1 |  |
| ALDH1L2 | GPR158 |  |
| EML5 | MYO3A |  |
| HCN4 | GAD2 |  |
| USP37 | APBB1IP |  |
| JDP2 | ABI1 |  |
| GPRC5A | ANKRD26 |  |
| CUL3 | YME1L1 |  |
| TRIP4 | MASTL |  |
| NXPE3 | ACBD5 |  |
| ST7L | PTCHD3 |  |
| MTO1 | MKX |  |
| RSBN1L | ARMC4 |  |
| MKI67 | MPP7 |  |
| ACAD10 | WAC |  |
| SF3B3 | BAMBI |  |
| TGIF1 | LYZL1 |  |
| SSBP2 | SVIL |  |

| TIFA | JCAD |  |
| --- | --- | --- |
| MEA1 | MTPAP |  |
| BRD3 | MAP3K8 |  |
| CD59 | ZNF438 |  |
| RAB3C | ZEB1 |  |
| MED9 | ARHGAP12 |  |
| CNNM2 | EPC1 |  |
| AKR7A2 | CCDC7 |  |
| ZNF559 | NRP1 |  |
| BNC2 | PARD3 |  |
| CDH2 | CUL2 |  |
| SQSTM1 | CREM |  |
| CHKA | CCNY |  |
| NSUN3 | GJD4 |  |
| POMT2 | FZD8 |  |
| RAB6B | MTRNR2L7 |  |
| ATG4B | ZNF248 |  |
| XRRA1 | ZNF33A |  |
| PANK3 | ZNF37A |  |
| SGIP1 | ZNF33B |  |
| RCL1 | BMS1 |  |
| GFRA1 | RET |  |
| CDH7 | RASGEF1A |  |
| TOX | FXYD4 |  |
| MCM3AP | HNRNPF |  |
| DGCR14 | ZNF487 |  |
| NSMCE1 | VMO1 |  |
| COA5 | PLD2 |  |
| PRPS1 | MINK1 |  |
| MCL1 | C17orf107 |  |
| ANXA11 | SLC25A11 |  |
| ZBTB10 | RNF167 |  |
| MRPL12 | PFN1 |  |
| AGT | SPAG7 |  |
| TNFAIP8L3 | CAMTA2 |  |
| IWS1 | INCA1 |  |
| ARNT | KIF1C |  |
| LMF1 | SLC52A1 |  |
| MGAT1 | ZFP3 |  |
| SFXN4 | ZNF232 |  |
| GPSM2 | USP6 |  |
| CAPN7 | ZNF594 |  |
| ARL5A | SCIMP |  |
| CYP4F12 | RABEP1 |  |
| KAZN | NUP88 |  |
| HMGN3 | RPAIN |  |
| TRDMT1 | DHX33 |  |
| IFT74 | RPS23 |  |
| CALCOCO2 | ATP6AP1L |  |
| SNX2 | TMEM167A |  |
| ZNF613 | VCAN |  |
| RELT | HAPLN1 |  |
| STYX | EDIL3 |  |

| MAGI3 | RASA1 |  |
| --- | --- | --- |
| C2orf72 | CCNH |  |
| TMEM8B | MEF2C |  |
| TUSC2 | MBLAC2 |  |
| COBLL1 | POLR3G |  |
| KIF5B | LYSMD3 |  |
| CLCC1 | ADGRV1 |  |
| ADHFE1 | ARRDC3 |  |
| MRPL3 | NR2F1 |  |
| MARCH3 | FAM172A |  |
| PDK1 | POU5F2 |  |
| VPS37A | MCTP1 |  |
| LRIG3 | ARSK |  |
| RFT1 | UPK3A |  |
| SMIM1 | FAM118A |  |
| SHB | RIBC2 |  |
| ZNF607 | ATXN10 |  |
| TSKU | WNT7B |  |
| DVL3 | PPARA |  |
| RAB11FIP1 | CDPF1 |  |
| PIFO | PKDREJ |  |
| SMARCA5 | TTC38 |  |
| ALDH7A1 | GTSE1 |  |
| RHOH | TRMU |  |
| SHOC2 | CELSR1 |  |
| HNRNPD | GRAMD4 |  |
| PTCHD1 | TBC1D22A |  |
| C4orf32 | FAM19A5 |  |
| OGFOD1 | BRD1 |  |
| KLHL18 | ZBED4 |  |
| SARDH | ALG12 |  |
| PACS2 | CRELD2 |  |
| ZNF736 | PIM3 |  |
| POLI | IL17REL |  |
| ORMDL1 | TTLL8 |  |
| CHD7 | MLC1 |  |
| TUBGCP2 | MOV10L1 |  |
| SNAP47 | TRABD |  |
| TRIM11 | SELENOO |  |
| HIST1H2AG | TUBGCP6 |  |
| UBE2I | HDAC10 |  |
| C22orf39 | MAPK12 |  |
| INTS8 | MAPK11 |  |
| PLCG2 | PLXNB2 |  |
| ECHDC3 | DENND6B |  |
| CCDC171 | PPP6R2 |  |
| WIPI2 | SBF1 |  |
| RRM1 | ADM2 |  |
| ATP2B2 | MIOX |  |
| LNPEP | LMF2 |  |
| ZNF780A | SCO2 |  |
| ENSA | TYMP |  |
| CTSS | KLHDC7B |  |

| NRDE2 | CPT1B |  |
| --- | --- | --- |
| ZNF786 | MAPK8IP2 |  |
| FIG4 | ARSA |  |
| RP11-73M18.2 | ACR |  |
| MED16 | RABL2B |  |
| BCL10 | LOC102724151 |  |
| PDLIM4 | PLCXD1 |  |
| GRSF1 | GTPBP6 |  |
| SOD2 | PPP2R3B |  |
| GRK5 | SHOX |  |
| C12orf5 | CRLF2 |  |
| PARL | CSF2RA |  |
| GNPTG | IL3RA |  |
| WDR3 | SLC25A6 |  |
| UQCRB | ASMTL |  |
| TRMU | P2RY8 |  |
| S100A9 | ASMT |  |
| IDH3A | DHRSX |  |
| CLUAP1 | CD99 |  |
| STAM2 | XG |  |
| RPS19 | GYG2 |  |
| PITPNC1 | ARSD |  |
| FAM216A | ARSE |  |
| CDR1as | ARSH |  |
| WDFY2 | ARSF |  |
| CEP120 | MXRA5 |  |
| TMEM43 | PRKX |  |
| NTMT1 | NLGN4X |  |
| NTPCR | VCX3A |  |
| NR2F6 | PUDP |  |
| CDC73 | STS |  |
| SLC25A11 | VCX |  |
| MTHFD1 | PNPLA4 |  |
| RNF126 | VCX2 |  |
| TARDBP | VCX3B |  |
| NFYA | FAM9B |  |
| SLC35F6 | TBL1X |  |
| NUP85 | GPR143 |  |
| HSPA13 | SHROOM2 |  |
| PLA2G16 | WWC3 |  |
| PTCD3 | CLCN4 |  |
| S100B | MID1 |  |
| DCBLD1 | HCCS |  |
| OPTN | ARHGAP6 |  |
| FOXN3 | AMELX |  |
| MTMR3 | MSL3 |  |
| RBP4 | FRMPD4 |  |
| HSBP1 | PRPS2 |  |
| DPY19L1 | TLR7 |  |
| LMOD1 | TLR8 |  |
| NUPL2 | TMSB4X |  |
| UBA6 | FAM9C |  |
| DNAJC10 | ATXN3L |  |

| C19orf47 | EGFL6 |  |
| --- | --- | --- |
| CEP57 | TCEANC |  |
| BAIAP2L1 | RAB9A |  |
| SERINC1 | TRAPPC2 |  |
| COPS2 | OFD1 |  |
| FLYWCH1 | GPM6B |  |
| SOCS7 | GEMIN8 |  |
| ZNF670 | GLRA2 |  |
| AGBL5 | FANCB |  |
| DNLZ | MOSPD2 |  |
| RPS12 | ASB9 |  |
| RAD50 | ASB11 |  |
| GATSL2 | PIGA |  |
| SMAD2 | VEGFD |  |
| FCRL1 | PIR |  |
| MAP4K2 | BMX |  |
| HUWE1 | ACE2 |  |
| EIF4E | CLTRN |  |
| TRIM4 | CA5B |  |
| ACO1 | AP1S2 |  |
| ZNF780B | GRPR |  |
| RTN3 | MAGEB17 |  |
| LRPAP1 | CTPS2 |  |
| PTGS1 | SYAP1 |  |
| DEFB105A | TXLNG |  |
| SCIMP | RBBP7 |  |
| GALNT2 | REPS2 |  |
| ESCO1 | NHS |  |
| LMF2 | SCML1 |  |
| NCKAP1 | RAI2 |  |
| TM4SF5 | BEND2 |  |
| DEFB105B | SCML2 |  |
| SEC23B | CDKL5 |  |
| TTR | RS1 |  |
| PIN1 | PPEF1 |  |
| LTF | PHKA2 |  |
| MAMDC4 | ADGRG2 |  |
| PPIL4 | PDHA1 |  |
| ACOT13 | MAP3K15 |  |
| TIMM10 | SH3KBP1 |  |
| HM13 | BCLAF3 |  |
| TSC22D2 | MAP7D2 |  |
| HIF1A | EIF1AX |  |
| SMIM14 | RPS6KA3 |  |
| CEP128 | CNKSR2 |  |
| ACOT2 | KLHL34 |  |
| C10orf71 | MBTPS2 |  |
| ANGPT4 | YY2 |  |
| CD180 | SMS |  |
| PGLS | PTCHD1 |  |
| ZNF593 | ACOT9 |  |
| NSMCE2 | APOO |  |
| LONP2 | KLHL15 |  |

| WDR73 | EIF2S3 |  |
| --- | --- | --- |
| PAGR1 | ZFX |  |
| ELAVL1 | PDK3 |  |
| ADSL | PCYT1B |  |
| MAGEB10 | POLA1 |  |
| SESN3 | ARX |  |
| CALML4 | MAGEB6 |  |
| MED14 | PPP4R3C |  |
| COL9A2 | DCAF8L2 |  |
| HRASLS5 | MAGEB10 |  |
| KIAA1462 | IL1RAPL1 |  |
| RAPGEF4 | MAGEB2 |  |
| SND1 | MAGEB3 |  |
| KLHL23 | MAGEB1 |  |
| RHD | NR0B1 |  |
| FPR1 | CXorf21 |  |
| UBE2T | TAB3 |  |
| CLNS1A | FTHL17 |  |
| EEA1 | DMD |  |
| CNTD1 | FAM47A |  |
| BEST3 | TMEM47 |  |
| HOXB2 | FAM47B |  |
| THBS2 | MAGEB16 |  |
| EPS8 | CFAP47 |  |
| NLRP9 | FAM47C |  |
| PTCHD3 | PRRG1 |  |
| HDAC2 | LANCL3 |  |
| CASK | XK |  |
| G6PC | CYBB |  |
| CD226 | SYTL5 |  |
| VWA2 | SRPX |  |
| ANKRD28 | OTC |  |
| SAMD15 | TSPAN7 |  |
| GRK6 | MID1IP1 |  |
| EIF2B2 | BCOR |  |
| LILRA2 | CXorf38 |  |
| SLFN12L | MED14 |  |
| FXYD5 | USP9X |  |
| NBPF24 | DDX3X |  |
| TACR2 | NYX |  |
| AZIN1 | CASK |  |
| TAB3 | GPR82 |  |
| SLC25A4 | PPP1R2C |  |
| C9orf3 | PIK3C2G |  |
| UROS | PLCZ1 |  |
| METTL22 | PLEKHA5 |  |
| MARCKS | AEBP2 |  |
| CLEC4C | PDE3A |  |
| KARS | SLCO1B3 |  |
| SEC14L4 | SLCO1B1 |  |
| SF3B14 | IAPP |  |
| EPHX2 | RECQL |  |
| CD3D | GYS2 |  |

| COG6 | LDHB |  |
| --- | --- | --- |
| CAD | KCNJ8 |  |
| SLA | ABCC9 |  |
| C14orf23 | ST8SIA1 |  |
| MYO1G | C2CD5 |  |
| OPN1MW | ETNK1 |  |
| MSH2 | SOX5 |  |
| TBL2 | BCAT1 |  |
| HEATR6 | LRMP |  |
| WISP1 | ETFRF1 |  |
| TP53TG5 | LMNTD1 |  |
| EMC1 | RASSF8 |  |
| BMP10 | BHLHE41 |  |
| WNT1 | SSPN |  |
| SPIC | INTS13 |  |
| SSR1 | FGFR1OP2 |  |
| ZBTB20 | TM7SF3 |  |
| WDR77 | MED21 |  |
| MOBP | ARNTL2 |  |
| OPN1MW2 | SMCO2 |  |
| ZBTB14 | PPFIBP1 |  |
| QDPR | MANSC4 |  |
| TLR8 | KLHL42 |  |
| TMX1 | PTHLH |  |
| DHX30 | FAR2 |  |
| FHL2 | ERGIC2 |  |
| C11orf85 | OVCH1 |  |
| FCGR1B | TMTC1 |  |
| ANAPC11 | IPO8 |  |
| AC004381.6 | CAPRIN2 |  |
| INO80 | TSPAN11 |  |
| MELK | SINHCAF |  |
| KDM2B | DENND5B |  |
| GPR78 | ETFBKMT |  |
| STX7 | AMN1 |  |
| GLYAT | KIAA1551 |  |
| TSHR | BICD1 |  |
| B3GALT6 | FGD4 |  |
| TIMD4 | DNM1L |  |
| FBXL3 | YARS2 |  |
| SLC9A3R1 | PKP2 |  |
| DCUN1D5 | SYT10 |  |
| PYCARD | ALG10 |  |
| GPX5 | ALG10B |  |
| AKAP1 | CPNE8 |  |
| KLHDC8A | ABCD2 |  |
| ITIH5 | SLC2A13 |  |
| ATG2A | LRRK2 |  |
| MTMR8 | CNTN1 |  |
| CHST12 | PDZRN4 |  |
| SHMT1 | GXYLT1 |  |
| CACNG2 | YAF2 |  |
| PMVK | ZCRB1 |  |

| GRM4 | PPHLN1 |  |
| --- | --- | --- |
| FDX1L | PRICKLE1 |  |
| GABRB1 | ADAMTS20 |  |
| PFAS | IRAK4 |  |
| LSM14B | TMEM117 |  |
| HIRIP3 | NELL2 |  |
| FAM207A | DBX2 |  |
| AQP6 | ANO6 |  |
| CTB-54O9.9 | ARID2 |  |
| RP11-210M15.2 | SCAF11 |  |
| TMC2 | SLC38A1 |  |
| SBF1 | SLC38A2 |  |
| TMEM136 | SLC38A4 |  |
| POTED | AMIGO2 |  |
| GBP6 | PCED1B |  |
| SELK | RPAP3 |  |
| MYCBP2 | ENDOU |  |
| EFHC2 | RAPGEF3 |  |
| C14orf164 | SLC48A1 |  |
| IL5RA | HDAC7 |  |
| NDUFB5 | VDR |  |
| DAP | COL2A1 |  |
| USP9X | SENP1 |  |
| DNASE1L3 | PFKM |  |
| TMEM115 | ASB8 |  |
| MKKS | CCDC184 |  |
| SP140L | ZNF641 |  |
| CTSO | C12orf54 |  |
| AVPI1 | CCNT1 |  |
| SPEN | TEX49 |  |
| SLC30A9 | ADCY6 |  |
| TMCO1 | CACNB3 |  |
| TLK2 | RND1 |  |
| BAG1 | CCDC65 |  |
| CD1C | FKBP11 |  |
| SLC22A2 | ARF3 |  |
| MX2 | WNT10B |  |
| AP5Z1 | WNT1 |  |
| GPR65 | DDN |  |
| SLC24A4 | PRKAG1 |  |
| NARS | KMT2D |  |
| DDX4 | RHEBL1 |  |
| PNRC1 | LMBR1L |  |
| PIGP | TUBA1B |  |
| CRLS1 | TUBA1A |  |
| ACP6 | TUBA1C |  |
| RAI14 | TROAP |  |
| GNG11 | DNAJC22 |  |
| MYOM2 | SPATS2 |  |
| ABHD16B | KCNH3 |  |
| TG | MCRS1 |  |
| MARC1 | FAM186B |  |
| C19orf24 | PRPF40B |  |

| NKX2-1 | FMNL3 |  |
| --- | --- | --- |
| CDH18 | TMBIM6 |  |
| GRIN2A | NCKAP5L |  |
| SIGLEC5 | BCDIN3D |  |
| RNF213 | FAIM2 |  |
| CNOT1 | AQP2 |  |
| TGFBR2 | AQP6 |  |
| SHC3 | ASIC1 |  |
| HSPA14 | SMARCD1 |  |
| CAMK2N1 | GPD1 |  |
| NAALAD2 | COX14 |  |
| C17orf72 | LIMA1 |  |
| ZNF8 | FAM186A |  |
| KY | LARP4 |  |
| MRPL22 | DIP2B |  |
| SP8 | ATF1 |  |
| IGFBP4 | TMPRSS12 |  |
| NDUFA10 | METTL7A |  |
| DSTN | SLC11A2 |  |
| MRPL36 | LETMD1 |  |
| ALDOA | CSRNP2 |  |
| RNMTL1 | TFCP2 |  |
| MSH3 | POU6F1 |  |
| VIL1 | RGS8 |  |
| C8A | NPL |  |
| CHST14 | DHX9 |  |
| PPIL2 | SHCBP1L |  |
| RCCD1 | LAMC1 |  |
| APOL3 | LAMC2 |  |
| BCDIN3D | NMNAT2 |  |
| HCN1 | SMG7 |  |
| NKD2 | NCF2 |  |
| DOCK7 | RGL1 |  |
| MBD5 | COLGALT2 |  |
| CKAP5 | TSEN15 |  |
| BTN3A1 | C1orf21 |  |
| ATP12A | EDEM3 |  |
| DUSP27 | FAM129A |  |
| METTL24 | TRMT1L |  |
| AGAP2 | SWT1 |  |
| CLEC14A | IVNS1ABP |  |
| PDHB | HMCN1 |  |
| SH3TC2 | PRG4 |  |
| ITGA1 | TPR |  |
| DMGDH | ODR4 |  |
| C18orf32 | BRINP3 |  |
| VCAM1 | RGS18 |  |
| SF3B1 | RGS21 |  |
| PIK3AP1 | RGS13 |  |
| EGF | UCHL5 |  |
| ATP5J2-PTCD1 | TROVE2 |  |
| MLIP | GLRX2 |  |
| CYSLTR2 | CDC73 |  |

| CTC1 | B3GALT2 |  |
| --- | --- | --- |
| RNF130 | KCNT2 |  |
| FHL5 | CFH |  |
| PAICS | CFHR4 |  |
| CRISPLD2 | CFHR2 |  |
| C4orf3 | CFHR5 |  |
| ENPP5 | ASPM |  |
| CRHR2 | ZBTB41 |  |
| LGR4 | CRB1 |  |
| PCDHB5 | DENND1B |  |
| RYK | C1orf53 |  |
| KNG1 | LHX9 |  |
| EMC3 | PTPRC |  |
| CYB5D2 | NR5A2 |  |
| UBE2J1 | ZNF281 |  |
| PCDHB15 | KIF14 |  |
| NQO2 | DDX59 |  |
| TLR4 | CAMSAP2 |  |
| ASAH2B | GPR25 |  |
| SARS | INAVA |  |
| SYNCRIP | KIF21B |  |
| CCAR2 | CACNA1S |  |
| FAM20A | ASCL5 |  |
| MRPL17 | TMEM9 |  |
| AKR1D1 | IGFN1 |  |
| FUS | PKP1 |  |
| THAP8 | TNNT2 |  |
| USP47 | LAD1 |  |
| RPS24 | TNNI1 |  |
| PTPRF | PHLDA3 |  |
| UQCRQ | CSRP1 |  |
| HINT1 | NAV1 |  |
| FREM2 | IPO9 |  |
| FUCA2 | SHISA4 |  |
| GUCY1A3 | LMOD1 |  |
| AGPS | TIMM17A |  |
| IFT46 | RNPEP |  |
| PRKACB | ELF3 |  |
| TP53BP2 | GPR37L1 |  |
| ARMCX3 | ARL8A |  |
| LAMA4 | PTPN7 |  |
| FGB | LGR6 |  |
| DNAH17 | PPP1R12B |  |
| PDLIM3 | SYT2 |  |
| HOPX | KDM5B |  |
| EBF4 | RABIF |  |
| RSU1 | ADIPOR1 |  |
| KCNC2 | CYB5R1 |  |
| SC5D | TMEM183A |  |
| IFIT1 | PPFIA4 |  |
| BET1 | MYOG |  |
| DENND2D | ADORA1 |  |
| ADAMTS5 | MYBPH |  |

| NFIB | CHI3L1 |  |
| --- | --- | --- |
| FAM64A | CHIT1 |  |
| BLVRA | BTG2 |  |
| FGG | FMOD |  |
| DYRK4 | PRELP |  |
| TROVE2 | OPTC |  |
| SEC23IP | ATP2B4 |  |
| HOXA1 | LAX1 |  |
| ZC4H2 | ZC3H11A |  |
| DENND2A | ZBED6 |  |
| ELMO1 | SOX13 |  |
| SH3BP5 | ETNK2 |  |
| APOB | REN |  |
| BTLA | PLEKHA6 |  |
| PMPCB | PPP1R15B |  |
| KIAA0430 | PIK3C2B |  |
| WT1 | MDM4 |  |
| KCNK5 | LRRN2 |  |
| TNFRSF14 | NFASC |  |
| PRMT7 | CNTN2 |  |
| IER5 | TMEM81 |  |
| YAE1D1 | RBBP5 |  |
| EIF2A | DSTYK |  |
| GIN1 | TMCC2 |  |
| LLPH | NUAK2 |  |
| SMTNL2 | KLHDC8A |  |
| OTC | LEMD1 |  |
| FRRS1 | CDK18 |  |
| TCN2 | MFSD4A |  |
| KLF2 | ELK4 |  |
| INHBC | SLC45A3 |  |
|  | RAB29 |  |
|  | SLC41A1 |  |
|  | PM20D1 |  |
|  | SLC26A9 |  |
|  | RAB7B |  |
|  | CTSE |  |
|  | RHEX |  |
|  | AVPR1B |  |
|  | FAM72A |  |
|  | SRGAP2 |  |
|  | IKBKE |  |
|  | RASSF5 |  |
|  | DYRK3 |  |
|  | MAPKAPK2 |  |
|  | IL10 |  |
|  | IL20 |  |
|  | IL24 |  |
|  | FCMR |  |
|  | PIGR |  |
|  | FCAMR |  |
|  | C1orf116 |  |
|  | YOD1 |  |

|  | PFKFB2 |  |
| --- | --- | --- |
|  | C4BPB |  |
|  | C4BPA |  |
|  | CD55 |  |
|  | CR2 |  |
|  | CR1 |  |
|  | CR1L |  |
|  | CD46 |  |
|  | CD34 |  |
|  | PLXNA2 |  |
|  | CAMK1G |  |
|  | LAMB3 |  |
|  | G0S2 |  |
|  | TRAF3IP3 |  |
|  | C1orf74 |  |
|  | IRF6 |  |
|  | DIEXF |  |
|  | SYT14 |  |
|  | SERTAD4 |  |
|  | HHAT |  |
|  | KCNH1 |  |
|  | RCOR3 |  |
|  | TRAF5 |  |
|  | RD3 |  |
|  | SLC30A1 |  |
|  | NEK2 |  |
|  | LPGAT1 |  |
|  | INTS7 |  |
|  | DTL |  |
|  | PTCH2 |  |
|  | EIF2B3 |  |
|  | HECTD3 |  |
|  | UROD |  |
|  | ZSWIM5 |  |
|  | MUTYH |  |
|  | TOE1 |  |
|  | CCDC163 |  |
|  | MMACHC |  |
|  | PRDX1 |  |
|  | NASP |  |
|  | CCDC17 |  |
|  | GPBP1L1 |  |
|  | TMEM69 |  |
|  | IPP |  |
|  | MAST2 |  |
|  | LOC110117498-PIK3R3 |  |
|  | PIK3R3 |  |
|  | TSPAN1 |  |
|  | POMGNT1 |  |
|  | LURAP1 |  |
|  | RAD54L |  |
|  | LRRC41 |  |
|  | NSUN4 |  |

|  | DMBX1 |  |
| --- | --- | --- |
|  | LOC105378696 |  |
|  | KNCN |  |
|  | MKNK1 |  |
|  | MOB3C |  |
|  | ATPAF1 |  |
|  | TEX38 |  |
|  | EFCAB14 |  |
|  | CYP4B1 |  |
|  | CYP4A11 |  |
|  | CYP4X1 |  |
|  | CYP4Z1 |  |
|  | CYP4A22 |  |
|  | PDZK1IP1 |  |
|  | TAL1 |  |
|  | STIL |  |
|  | CMPK1 |  |
|  | FOXD2 |  |
|  | TRABD2B |  |
|  | SLC5A9 |  |
|  | AGBL4 |  |
|  | ELAVL4 |  |
|  | DMRTA2 |  |
|  | FAF1 |  |
|  | CDKN2C |  |
|  | RNF11 |  |
|  | TTC39A |  |
|  | EPS15 |  |
|  | OSBPL9 |  |
|  | NRDC |  |
|  | RAB3B |  |
|  | TXNDC12 |  |
|  | KTI12 |  |
|  | BTF3L4 |  |
|  | ZFYVE9 |  |
|  | CC2D1B |  |
|  | ORC1 |  |
|  | PRPF38A |  |
|  | ZCCHC11 |  |
|  | GPX7 |  |
|  | SHISAL2A |  |
|  | ZYG11B |  |
|  | ZYG11A |  |
|  | ECHDC2 |  |
|  | SCP2 |  |
|  | PODN |  |
|  | SLC1A7 |  |
|  | CPT2 |  |
|  | LRP8 |  |
|  | DMRTB1 |  |
|  | GLIS1 |  |
|  | NDC1 |  |
|  | YIPF1 |  |

|  | LRRC42 |  |
| --- | --- | --- |
|  | LDLRAD1 |  |
|  | TMEM59 |  |
|  | TCEANC2 |  |
|  | CYB5RL |  |
|  | MRPL37 |  |
|  | SSBP3 |  |
|  | ACOT11 |  |
|  | MROH7 |  |
|  | TTC4 |  |
|  | PARS2 |  |
|  | TTC22 |  |
|  | LEXM |  |
|  | DHCR24 |  |
|  | BSND |  |
|  | PCSK9 |  |
|  | USP24 |  |
|  | PLPP3 |  |
|  | PRKAA2 |  |
|  | FYB2 |  |
|  | C8A |  |
|  | C8B |  |
|  | OMA1 |  |
|  | TACSTD2 |  |
|  | JUN |  |
|  | FGGY |  |
|  | C1orf87 |  |
|  | NFIA |  |
|  | PATJ |  |
|  | L1TD1 |  |
|  | KANK4 |  |
|  | USP1 |  |
|  | DOCK7 |  |
|  | ANGPTL3 |  |
|  | FOXD3 |  |
|  | ALG6 |  |
|  | EFCAB7 |  |
|  | ROR1 |  |
|  | UBE2U |  |
|  | CACHD1 |  |
|  | RAVER2 |  |
|  | JAK1 |  |
|  | AK4 |  |
|  | DNAJC6 |  |
|  | LEPROT |  |
|  | LEPR |  |
|  | PDE4B |  |
|  | SGIP1 |  |
|  | MIER1 |  |
|  | SLC35D1 |  |
|  | C1orf141 |  |
|  | IL23R |  |
|  | IL12RB2 |  |

|  | SERBP1 |  |
| --- | --- | --- |
|  | GNG12 |  |
|  | DIRAS3 |  |
|  | WLS |  |
|  | DEPDC1 |  |
|  | LRRC7 |  |
|  | SRSF11 |  |
|  | ANKRD13C |  |
|  | HHLA3 |  |
|  | CTH |  |
|  | PTGER3 |  |
|  | NEGR1 |  |
|  | LRRIQ3 |  |
|  | FPGT |  |
|  | CRYZ |  |
|  | TTBK1 |  |
|  | SLC22A7 |  |
|  | CRIP3 |  |
|  | ZNF318 |  |
|  | ABCC10 |  |
|  | DLK2 |  |
|  | TJAP1 |  |
|  | YIPF3 |  |
|  | POLR1C |  |
|  | XPO5 |  |
|  | POLH |  |
|  | GTPBP2 |  |
|  | MAD2L1BP |  |
|  | RSPH9 |  |
|  | VEGFA |  |
|  | MRPL14 |  |
|  | TMEM63B |  |
|  | CAPN11 |  |
|  | SLC29A1 |  |
|  | HSP90AB1 |  |
|  | SLC35B2 |  |
|  | NFKBIE |  |
|  | TMEM151B |  |
|  | TCTE1 |  |
|  | AARS2 |  |
|  | CDC5L |  |
|  | SUPT3H |  |
|  | RUNX2 |  |
|  | CLIC5 |  |
|  | ENPP4 |  |
|  | ENPP5 |  |
|  | RCAN2 |  |
|  | TDRD6 |  |
|  | MEP1A |  |
|  | ADGRF5 |  |
|  | ADGRF1 |  |
|  | TNFRSF21 |  |
|  | CD2AP |  |

|  | ADGRF2 |  |
| --- | --- | --- |
|  | ADGRF4 |  |
|  | OPN5 |  |
|  | PTCHD4 |  |
|  | CENPQ |  |
|  | GLYATL3 |  |
|  | C6orf141 |  |
|  | RHAG |  |
|  | CRISP3 |  |
|  | PGK2 |  |
|  | CRISP1 |  |
|  | TFAP2B |  |
|  | PKHD1 |  |
|  | IL17A |  |
|  | IL17F |  |
|  | MCM3 |  |
|  | PAQR8 |  |
|  | EFHC1 |  |
|  | TRAM2 |  |
|  | GSTA2 |  |
|  | GSTA1 |  |
|  | GSTA3 |  |
|  | GSTA4 |  |
|  | ICK |  |
|  | FBXO9 |  |
|  | GCM1 |  |
|  | ELOVL5 |  |
|  | GCLC |  |
|  | KLHL31 |  |
|  | MLIP |  |
|  | TINAG |  |
|  | FAM83B |  |
|  | HCRTR2 |  |
|  | GFRAL |  |
|  | HMGCLL1 |  |
|  | BMP5 |  |
|  | COL21A1 |  |
|  | DST |  |
|  | BEND6 |  |
|  | KIAA1586 |  |
|  | ZNF451 |  |
|  | PRIM2 |  |
|  | KHDRBS2 |  |
|  | LGSN |  |
|  | PHF3 |  |
|  | EYS |  |
|  | ADGRB3 |  |
|  | COL19A1 |  |
|  | COL9A1 |  |
|  | SMAP1 |  |
|  | B3GAT2 |  |
|  | OGFRL1 |  |
|  | RIMS1 |  |

|  | KCNQ5 |  |
| --- | --- | --- |
|  | KHDC1 |  |
|  | KHDC3L |  |
|  | DDX43 |  |
|  | CGAS |  |
|  | EEF1A1 |  |
|  | CD109 |  |
|  | COL12A1 |  |
|  | TMEM30A |  |
|  | FILIP1 |  |
|  | SENP6 |  |
|  | MYO6 |  |
|  | IMPG1 |  |
|  | HTR1B |  |
|  | MEI4 |  |
|  | IRAK1BP1 |  |
|  | PHIP |  |
|  | HMGN3 |  |
|  | LCA5 |  |
|  | SH3BGRL2 |  |
|  | BCKDHB |  |
|  | FAM46A |  |
|  | IBTK |  |
|  | TPBG |  |
|  | UBE3D |  |
|  | DOPEY1 |  |
|  | PGM3 |  |
|  | RWDD2A |  |
|  | PRSS35 |  |
|  | SNAP91 |  |
|  | RIPPLY2 |  |
|  | CYB5R4 |  |
|  | MRAP2 |  |
|  | TBX18 |  |
|  | NT5E |  |
|  | SNX14 |  |
|  | SYNCRIP |  |
|  | HTR1E |  |
|  | CGA |  |
|  | ZNF292 |  |
|  | GJB7 |  |
|  | SMIM8 |  |
|  | C6orf163 |  |
|  | CFAP206 |  |
|  | RARS2 |  |
|  | ORC3 |  |
|  | AKIRIN2 |  |
|  | CNR1 |  |
|  | RNGTT |  |
|  | PNRC1 |  |
|  | SRSF12 |  |
|  | GABRR1 |  |
|  | GABRR2 |  |

|  | RRAGD |  |
| --- | --- | --- |
|  | ANKRD6 |  |
|  | LYRM2 |  |
|  | MDN1 |  |
|  | CASP8AP2 |  |
|  | BACH2 |  |
|  | MAP3K7 |  |
|  | EPHA7 |  |
|  | MANEA |  |
|  | FUT9 |  |
|  | UFL1 |  |
|  | GPR63 |  |
|  | KLHL32 |  |
|  | MMS22L |  |
|  | POU3F2 |  |
|  | FBXL4 |  |
|  | FAXC |  |
|  | COQ3 |  |
|  | PNISR |  |
|  | USP45 |  |
|  | PRDM13 |  |
|  | MCHR2 |  |
|  | SIM1 |  |
|  | ASCC3 |  |
|  | GRIK2 |  |
|  | LIN28B |  |
|  | BVES |  |
|  | ERLIN2 |  |
|  | ADGRA2 |  |
|  | BRF2 |  |
|  | RAB11FIP1 |  |
|  | GOT1L1 |  |
|  | ADRB3 |  |
|  | EIF4EBP1 |  |
|  | STAR |  |
|  | LSM1 |  |
|  | BAG4 |  |
|  | DDHD2 |  |
|  | PLPP5 |  |
|  | NSD3 |  |
|  | LETM2 |  |
|  | FGFR1 |  |
|  | C8orf86 |  |
|  | TACC1 |  |
|  | PLEKHA2 |  |
|  | TM2D2 |  |
|  | ADAM32 |  |
|  | ADAM18 |  |
|  | ADAM2 |  |
|  | IDO1 |  |
|  | IDO2 |  |
|  | TCIM |  |
|  | ZMAT4 |  |

|  | SFRP1 |  |
| --- | --- | --- |
|  | GOLGA7 |  |
|  | GINS4 |  |
|  | GPAT4 |  |
|  | ANK1 |  |
|  | KAT6A |  |
|  | AP3M2 |  |
|  | PLAT |  |
|  | IKBKB |  |
|  | DKK4 |  |
|  | VDAC3 |  |
|  | SLC20A2 |  |
|  | SMIM19 |  |
|  | CHRNB3 |  |
|  | CHRNA6 |  |
|  | THAP1 |  |
|  | RNF170 |  |
|  | HOOK3 |  |
|  | FNTA |  |
|  | POMK |  |
|  | HGSNAT |  |
|  | POTEA |  |
|  | SPIDR |  |
|  | CEBPD |  |
|  | PRKDC |  |
|  | MCM4 |  |
|  | SNAI2 |  |
|  | PPDPFL |  |
|  | SNTG1 |  |
|  | PXDNL |  |
|  | PCMTD1 |  |
|  | ALKAL1 |  |
|  | RB1CC1 |  |
|  | NPBWR1 |  |
|  | OPRK1 |  |
|  | ATP6V1H |  |
|  | RGS20 |  |
|  | LYPLA1 |  |
|  | MRPL15 |  |
|  | SOX17 |  |
|  | RP1 |  |
|  | XKR4 |  |
|  | TMEM68 |  |
|  | TGS1 |  |
|  | RPS20 |  |
|  | MOS |  |
|  | PLAG1 |  |
|  | CHCHD7 |  |
|  | SDR16C5 |  |
|  | PENK |  |
|  | IMPAD1 |  |
|  | FAM110B |  |
|  | ARRB1 |  |

|  | RPS3 |  |
| --- | --- | --- |
|  | KLHL35 |  |
|  | GDPD5 |  |
|  | SERPINH1 |  |
|  | MAP6 |  |
|  | MOGAT2 |  |
|  | DGAT2 |  |
|  | UVRAG |  |
|  | WNT11 |  |
|  | THAP12 |  |
|  | GVQW3 |  |
|  | EMSY |  |
|  | LRRC32 |  |
|  | TSKU |  |
|  | ACER3 |  |
|  | B3GNT6 |  |
|  | CAPN5 |  |
|  | MYO7A |  |
|  | GDPD4 |  |
|  | PAK1 |  |
|  | AQP11 |  |
|  | RSF1 |  |
|  | INTS4 |  |
|  | NDUFC2-KCTD14 |  |
|  | KCTD14 |  |
|  | THRSP |  |
|  | NDUFC2 |  |
|  | KCTD21 |  |
|  | USP35 |  |
|  | GAB2 |  |
|  | TENM4 |  |
|  | PRCP |  |
|  | DDIAS |  |
|  | RAB30 |  |
|  | PCF11 |  |
|  | ANKRD42 |  |
|  | CCDC90B |  |
|  | DLG2 |  |
|  | CREBZF |  |
|  | CCDC89 |  |
|  | SYTL2 |  |
|  | CCDC83 |  |
|  | EED |  |
|  | CCDC81 |  |
|  | ME3 |  |
|  | PRSS23 |  |
|  | FZD4 |  |
|  | TMEM135 |  |
|  | RAB38 |  |
|  | CTSC |  |
|  | GRM5 |  |
|  | TYR |  |
|  | NOX4 |  |

|  | TRIM77 |  |
| --- | --- | --- |
|  | TRIM49 |  |
|  | TRIM64B |  |
|  | TRIM49D1 |  |
|  | TRIM49D2 |  |
|  | TRIM64 |  |
|  | TRIM49C |  |
|  | UBTFL1 |  |
|  | NAALAD2 |  |
|  | FAT3 |  |
|  | MTNR1B |  |
|  | DEUP1 |  |
|  | SMCO4 |  |
|  | CEP295 |  |
|  | C11orf54 |  |
|  | MED17 |  |
|  | HEPHL1 |  |
|  | PANX1 |  |
|  | GPR83 |  |
|  | ANKRD49 |  |
|  | FUT4 |  |
|  | PIWIL4 |  |
|  | AMOTL1 |  |
|  | CWC15 |  |
|  | KDM4D |  |
|  | SRSF8 |  |
|  | ENDOD1 |  |
|  | SESN3 |  |
|  | FAM76B |  |
|  | CEP57 |  |
|  | MTMR2 |  |
|  | MAML2 |  |
|  | CCDC82 |  |
|  | JRKL |  |
|  | CNTN5 |  |
|  | ARHGAP42 |  |
|  | PGR |  |
|  | TRPC6 |  |
|  | CEP126 |  |
|  | CFAP300 |  |
|  | YAP1 |  |
|  | BIRC3 |  |
|  | BIRC2 |  |
|  | MMP20 |  |
|  | MMP27 |  |
|  | MMP8 |  |
|  | MMP10 |  |
|  | MMP12 |  |
|  | DCUN1D5 |  |
|  | DYNC2H1 |  |
|  | DDI1 |  |
|  | CASP4 |  |
|  | CASP5 |  |

|  | CASP1 |  |
| --- | --- | --- |
|  | CARD16 |  |
|  | GRIA4 |  |
|  | MSANTD4 |  |
|  | KBTBD3 |  |
|  | AASDHPPT |  |
|  | CWF19L2 |  |
|  | ELMOD1 |  |
|  | SLC35F2 |  |
|  | RAB39A |  |
|  | CUL5 |  |
|  | ACAT1 |  |
|  | NPAT |  |
|  | ATM |  |
|  | KDELC2 |  |
|  | EXPH5 |  |
|  | DDX10 |  |
|  | C11orf87 |  |
|  | ZC3H12C |  |
|  | RDX |  |
|  | FDX1 |  |
|  | ARHGAP20 |  |
|  | C11orf53 |  |
|  | COLCA2 |  |
|  | POU2AF1 |  |
|  | C11orf88 |  |
|  | LAYN |  |
|  | SIK2 |  |
|  | PPP2R1B |  |
|  | ALG9 |  |
|  | FDXACB1 |  |
|  | C11orf1 |  |
|  | CRYAB |  |
|  | HSPB2 |  |
|  | C11orf52 |  |
|  | DIXDC1 |  |
|  | DLAT |  |
|  | PIH1D2 |  |
|  | NKAPD1 |  |
|  | IL18 |  |
|  | BCO2 |  |
|  | PLET1 |  |
|  | NCAM1 |  |
|  | TTC12 |  |
|  | IVL |  |
|  | SPRR4 |  |
|  | SPRR3 |  |
|  | SPRR2D |  |
|  | SPRR2B |  |
|  | SPRR2F |  |
|  | SPRR2G |  |
|  | LELP1 |  |
|  | PRR9 |  |

|  | LOR |  |
| --- | --- | --- |
|  | PGLYRP3 |  |
|  | PGLYRP4 |  |
|  | S100A9 |  |
|  | S100A12 |  |
|  | S100A7A |  |
|  | S100A7 |  |
|  | S100A5 |  |
|  | S100A3 |  |
|  | S100A2 |  |
|  | S100A16 |  |
|  | S100A14 |  |
|  | S100A1 |  |
|  | CHTOP |  |
|  | SNAPIN |  |
|  | INTS3 |  |
|  | SLC27A3 |  |
|  | GATAD2B |  |
|  | DENND4B |  |
|  | CRTC2 |  |
|  | SLC39A1 |  |
|  | CREB3L4 |  |
|  | RAB13 |  |
|  | NUP210L |  |
|  | TPM3 |  |
|  | C1orf43 |  |
|  | UBAP2L |  |
|  | HAX1 |  |
|  | AQP10 |  |
|  | IL6R |  |
|  | SHE |  |
|  | CHRNB2 |  |
|  | ADAR |  |
|  | KCNN3 |  |
|  | PBXIP1 |  |
|  | SHC1 |  |
|  | CKS1B |  |
|  | FLAD1 |  |
|  | LENEP |  |
|  | ZBTB7B |  |
|  | DCST2 |  |
|  | DCST1 |  |
|  | ADAM15 |  |
|  | EFNA3 |  |
|  | EFNA1 |  |
|  | SLC50A1 |  |
|  | DPM3 |  |
|  | KRTCAP2 |  |
|  | TRIM46 |  |
|  | MUC1 |  |
|  | THBS3 |  |
|  | MTX1 |  |
|  | GBA |  |

|  | FAM189B |  |
| --- | --- | --- |
|  | SCAMP3 |  |
|  | PKLR |  |
|  | FDPS |  |
|  | RUSC1 |  |
|  | ASH1L |  |
|  | MSTO1 |  |
|  | YY1AP1 |  |
|  | DAP3 |  |
|  | GON4L |  |
|  | SYT11 |  |
|  | RIT1 |  |
|  | RXFP4 |  |
|  | ARHGEF2 |  |
|  | SSR2 |  |
|  | UBQLN4 |  |
|  | LAMTOR2 |  |
|  | MEX3A |  |
|  | LMNA |  |
|  | SEMA4A |  |
|  | SLC25A44 |  |
|  | PMF1 |  |
|  | BGLAP |  |
|  | PAQR6 |  |
|  | SMG5 |  |
|  | GLMP |  |
|  | RHBG |  |
|  | C1orf61 |  |
|  | MEF2D |  |
|  | IQGAP3 |  |
|  | TTC24 |  |
|  | NAXE |  |
|  | GPATCH4 |  |
|  | BCAN |  |
|  | NES |  |
|  | CRABP2 |  |
|  | RRNAD1 |  |
|  | HDGF |  |
|  | PRCC |  |
|  | SH2D2A |  |
|  | NTRK1 |  |
|  | INSRR |  |
|  | PEAR1 |  |
|  | LRRC71 |  |
|  | ARHGEF11 |  |
|  | ETV3L |  |
|  | ETV3 |  |
|  | FCRL5 |  |
|  | FCRL4 |  |
|  | FCRL3 |  |
|  | FCRL1 |  |
|  | CD5L |  |
|  | KIRREL1 |  |

|  | CD1D |  |
| --- | --- | --- |
|  | CD1A |  |
|  | CD1C |  |
|  | CD1B |  |
|  | CD1E |  |
|  | OR10K1 |  |
|  | OR10R2 |  |
|  | OR6Y1 |  |
|  | OR6P1 |  |
|  | OR10X1 |  |
|  | OR10Z1 |  |
|  | SPTA1 |  |
|  | OR6K2 |  |
|  | OR6N1 |  |
|  | OR6N2 |  |
|  | PYHIN1 |  |
|  | IFI16 |  |
|  | CADM3 |  |
|  | ACKR1 |  |
|  | FCER1A |  |
|  | OR10J3 |  |
|  | OR10J4 |  |
|  | APCS |  |
|  | CRP |  |
|  | FCRL6 |  |
|  | SLAMF8 |  |
|  | VSIG8 |  |
|  | CFAP45 |  |
|  | ZNF32 |  |
|  | CXCL12 |  |
|  | TMEM72 |  |
|  | ZNF22 |  |
|  | OR13A1 |  |
|  | ALOX5 |  |
|  | 8-Mar |  |
|  | WASHC2C |  |
|  | AGAP4 |  |
|  | NCOA4 |  |
|  | ANXA8L1 |  |
|  | NPY4R |  |
|  | GPRIN2 |  |
|  | SYT15 |  |
|  | GDF2 |  |
|  | RBP3 |  |
|  | ANXA8 |  |
|  | AGAP9 |  |
|  | NPY4R2 |  |
|  | FRMPD2 |  |
|  | MAPK8 |  |
|  | ARHGAP22 |  |
|  | WDFY4 |  |
|  | LRRC18 |  |
|  | VSTM4 |  |

|  | FAM170B |  |
| --- | --- | --- |
|  | C10orf128 |  |
|  | C10orf71 |  |
|  | DRGX |  |
|  | ERCC6 |  |
|  | CHAT |  |
|  | SLC18A3 |  |
|  | C10orf53 |  |
|  | OGDHL |  |
|  | TIMM23B |  |
|  | AGAP6 |  |
|  | WASHC2A |  |
|  | ASAH2 |  |
|  | SGMS1 |  |
|  | A1CF |  |
|  | PRKG1 |  |
|  | CSTF2T |  |
|  | DKK1 |  |
|  | MBL2 |  |
|  | PCDH15 |  |
|  | ZWINT |  |
|  | TFAM |  |
|  | PHYHIPL |  |
|  | FAM13C |  |
|  | SLC16A9 |  |
|  | MRLN |  |
|  | CCDC6 |  |
|  | ANK3 |  |
|  | RHOBTB1 |  |
|  | TMEM26 |  |
|  | CABCOCO1 |  |
|  | ARID5B |  |
|  | RTKN2 |  |
|  | ZNF365 |  |
|  | ADO |  |
|  | EGR2 |  |
|  | JMJD1C |  |
|  | REEP3 |  |
|  | CTNNA3 |  |
|  | LRRTM3 |  |
|  | DNAJC12 |  |
|  | SIRT1 |  |
|  | HERC4 |  |
|  | MYPN |  |
|  | PBLD |  |
|  | HNRNPH3 |  |
|  | SLC25A16 |  |
|  | TET1 |  |
|  | DDX50 |  |
|  | DDX21 |  |
|  | KIF1BP |  |
|  | VPS26A |  |
|  | SUPV3L1 |  |

|  | HKDC1 |  |
| --- | --- | --- |
|  | HK1 |  |
|  | TSPAN15 |  |
|  | NEUROG3 |  |
|  | FAM241B |  |
|  | COL13A1 |  |
|  | AIFM2 |  |
|  | TYSND1 |  |
|  | SAR1A |  |
|  | NPFFR1 |  |
|  | LRRC20 |  |
|  | EIF4EBP2 |  |
|  | PRF1 |  |
|  | TBATA |  |
|  | SGPL1 |  |
|  | PCBD1 |  |
|  | UNC5B |  |
|  | SLC29A3 |  |
|  | CDH23 |  |
|  | C10orf105 |  |
|  | VSIR |  |
|  | PSAP |  |
|  | CHST3 |  |
|  | SPOCK2 |  |
|  | ASCC1 |  |
|  | ANAPC16 |  |
|  | DNAJB12 |  |
|  | MICU1 |  |
|  | MCU |  |
|  | OIT3 |  |
|  | P4HA1 |  |
|  | ECD |  |
|  | FAM149B1 |  |
|  | DNAJC9 |  |
|  | MRPS16 |  |
|  | ANXA7 |  |
|  | MSS51 |  |
|  | USP54 |  |
|  | MYOZ1 |  |
|  | SYNPO2L |  |
|  | AGAP5 |  |
|  | SEC24C |  |
|  | CHCHD1 |  |
|  | ZSWIM8 |  |
|  | NDST2 |  |
|  | CAMK2G |  |
|  | PLAU |  |
|  | VCL |  |
|  | AP3M1 |  |
|  | ADK |  |
|  | KAT6B |  |
|  | DUSP13 |  |
|  | SAMD8 |  |

|  | VDAC2 |  |
| --- | --- | --- |
|  | COMTD1 |  |
|  | ZNF503 |  |
|  | LRMDA |  |
|  | KCNMA1 |  |
|  | IRF5 |  |
|  | TNPO3 |  |
|  | TSPAN33 |  |
|  | SMO |  |
|  | AHCYL2 |  |
|  | STRIP2 |  |
|  | NRF1 |  |
|  | UBE2H |  |
|  | ZC3HC1 |  |
|  | KLHDC10 |  |
|  | TMEM209 |  |
|  | CPA2 |  |
|  | CPA4 |  |
|  | CPA5 |  |
|  | CEP41 |  |
|  | MEST |  |
|  | COPG2 |  |
|  | TSGA13 |  |
|  | KLF14 |  |
|  | MKLN1 |  |
|  | PODXL |  |
|  | PLXNA4 |  |
|  | LRGUK |  |
|  | AKR1B1 |  |
|  | AKR1B15 |  |
|  | BPGM |  |
|  | CALD1 |  |
|  | AGBL3 |  |
|  | TMEM140 |  |
|  | CYREN |  |
|  | WDR91 |  |
|  | STRA8 |  |
|  | CNOT4 |  |
|  | NUP205 |  |
|  | STMP1 |  |
|  | SLC13A4 |  |
|  | FAM180A |  |
|  | MTPN |  |
|  | LUZP6 |  |
|  | CHRM2 |  |
|  | PTN |  |
|  | DGKI |  |
|  | CREB3L2 |  |
|  | AKR1D1 |  |
|  | TRIM24 |  |
|  | SVOPL |  |
|  | ATP6V0A4 |  |
|  | TMEM213 |  |

|  | KIAA1549 |  |
| --- | --- | --- |
|  | ZC3HAV1L |  |
|  | ZC3HAV1 |  |
|  | TTC26 |  |
|  | UBN2 |  |
|  | LUC7L2 |  |
|  | C7orf55-LUC7L2 |  |
|  | FMC1 |  |
|  | KLRG2 |  |
|  | CLEC2L |  |
|  | HIPK2 |  |
|  | TBXAS1 |  |
|  | PARP12 |  |
|  | KDM7A |  |
|  | SLC37A3 |  |
|  | RAB19 |  |
|  | MKRN1 |  |
|  | DENND2A |  |
|  | NDUFB2 |  |
|  | BRAF |  |
|  | TMEM178B |  |
|  | AGK |  |
|  | KIAA1147 |  |
|  | WEE2 |  |
|  | SSBP1 |  |
|  | TAS2R38 |  |
|  | MGAM |  |
|  | MGAM2 |  |
|  | MTRNR2L6 |  |
|  | PRSS1 |  |
|  | PRSS2 |  |
|  | EPHB6 |  |
|  | TRPV6 |  |
|  | TRPV5 |  |
|  | LLCFC1 |  |
|  | KEL |  |
|  | TAS2R39 |  |
|  | GSTK1 |  |
|  | TMEM139 |  |
|  | CASP2 |  |
|  | FAM131B |  |
|  | ZYX |  |
|  | EPHA1 |  |
|  | CTAGE15 |  |
|  | TCAF2 |  |
|  | CTAGE6 |  |
|  | TCAF1 |  |
|  | OR2F1 |  |
|  | OR6B1 |  |
|  | OR2A5 |  |
|  | OR2A2 |  |
|  | OR2A14 |  |
|  | CTAGE4 |  |

|  | ARHGEF35 |  |
| --- | --- | --- |
|  | OR2A42 |  |
|  | OR2A7 |  |
|  | CTAGE8 |  |
|  | ARHGEF5 |  |
|  | NOBOX |  |
|  | TPK1 |  |
|  | CNTNAP2 |  |
|  | PDIA4 |  |
|  | ZNF398 |  |
|  | ZNF212 |  |
|  | ZNF777 |  |
|  | ZNF746 |  |
|  | KRBA1 |  |
|  | ZNF467 |  |
|  | ZNF862 |  |
|  | ATP6V0E2 |  |
|  | ACTR3C |  |
|  | LRRC61 |  |
|  | ZBED6CL |  |
|  | REPIN1 |  |
|  | ZNF775 |  |
|  | GIMAP8 |  |
|  | GIMAP1-GIMAP5 |  |
|  | GIMAP1 |  |
|  | TMEM176B |  |
|  | TMEM176A |  |
|  | AOC1 |  |
|  | KCNH2 |  |
|  | NOS3 |  |
|  | ATG9B |  |
|  | ABCB8 |  |
|  | ASIC3 |  |
|  | CDK5 |  |
|  | SLC4A2 |  |
|  | FASTK |  |
|  | TMUB1 |  |
|  | AGAP3 |  |
|  | GBX1 |  |
|  | ASB10 |  |
|  | IQCA1L |  |
|  | ABCF2 |  |
|  | CHPF2 |  |
|  | NUB1 |  |
|  | CRYGN |  |
|  | PRKAG2 |  |
|  | GALNTL5 |  |
|  | GALNT11 |  |
|  | KMT2C |  |
|  | ACTR3B |  |
|  | DPP6 |  |
|  | HTR5A |  |
|  | INSIG1 |  |

|  | RBM33 |  |
| --- | --- | --- |
|  | RNF32 |  |
|  | LMBR1 |  |
|  | NOM1 |  |
|  | MNX1 |  |
|  | UBE3C |  |
|  | DNAJB6 |  |
|  | PTPRN2 |  |
|  | NCAPG2 |  |
|  | ESYT2 |  |
|  | VIPR2 |  |
|  | OR4F21 |  |
|  | ZNF596 |  |
|  | HPRT1 |  |
|  | PLAC1 |  |
|  | FAM122B |  |
|  | FAM122C |  |
|  | MOSPD1 |  |
|  | SMIM10 |  |
|  | RTL8B |  |
|  | RTL8C |  |
|  | RTL8A |  |
|  | SMIM10L2B |  |
|  | CT55 |  |
|  | ZNF75D |  |
|  | ZNF449 |  |
|  | SMIM10L2A |  |
|  | INTS6L |  |
|  | CT45A10 |  |
|  | MMGT1 |  |
|  | SLC9A6 |  |
|  | FHL1 |  |
|  | MAP7D3 |  |
|  | ADGRG4 |  |
|  | HTATSF1 |  |
|  | VGLL1 |  |
|  | CD40LG |  |
|  | ARHGEF6 |  |
|  | ZIC3 |  |
|  | FGF13 |  |
|  | F9 |  |
|  | MCF2 |  |
|  | ATP11C |  |
|  | SOX3 |  |
|  | LDOC1 |  |
|  | SPANXC |  |
|  | SPANXA1 |  |
|  | SPANXA2 |  |
|  | SPANXD |  |
|  | MAGEC3 |  |
|  | MAGEC1 |  |
|  | MAGEC2 |  |
|  | SLITRK4 |  |

|  | SPANXN1 |  |
| --- | --- | --- |
|  | FMR1 |  |
|  | FMR1NB |  |
|  | AFF2 |  |
|  | IDS |  |
|  | CXorf40A |  |
|  | HSFX3 |  |
|  | MAGEA9B |  |
|  | HSFX2 |  |
|  | TMEM185A |  |
|  | MAGEA11 |  |
|  | HSFX1 |  |
|  | MAGEA9 |  |
|  | MAGEA8 |  |
|  | CXorf40B |  |
|  | HSFX4 |  |
|  | MAMLD1 |  |
|  | MTM1 |  |
|  | MTMR1 |  |
|  | CD99L2 |  |
|  | HMGB3 |  |
|  | GPR50 |  |
|  | VMA21 |  |
|  | PASD1 |  |
|  | PRRG3 |  |
|  | CNGA2 |  |
|  | MAGEA4 |  |
|  | GABRE |  |
|  | MAGEA10 |  |
|  | GABRA3 |  |
|  | GABRQ |  |
|  | MAGEA3 |  |
|  | CSAG2 |  |
|  | MAGEA2B |  |
|  | CSAG1 |  |
|  | MAGEA12 |  |
|  | MAGEA2 |  |
|  | CSAG3 |  |
|  | MAGEA6 |  |
|  | CETN2 |  |
|  | ZNF185 |  |
|  | PNMA5 |  |
|  | PNMA3 |  |
|  | PNMA6F |  |
|  | ZNF275 |  |
|  | PNMA6E |  |
|  | BGN |  |
|  | ATP2B3 |  |
|  | DUSP9 |  |
|  | PNCK |  |
|  | SLC6A8 |  |
|  | BCAP31 |  |
|  | ABCD1 |  |

|  | PLXNB3 |  |
| --- | --- | --- |
|  | SRPK3 |  |
|  | IDH3G |  |
|  | SSR4 |  |
|  | PDZD4 |  |
|  | L1CAM |  |
|  | AVPR2 |  |
|  | ARHGAP4 |  |
|  | NAA10 |  |
|  | RENBP |  |
|  | HCFC1 |  |
|  | TMEM187 |  |
|  | IRAK1 |  |
|  | MECP2 |  |
|  | OPN1LW |  |
|  | OPN1MW |  |
|  | OPN1MW2 |  |
|  | OPN1MW3 |  |
|  | TEX28 |  |
|  | TKTL1 |  |
|  | FLNA |  |
|  | EMD |  |
|  | RPL10 |  |
|  | DNASE1L1 |  |
|  | TAZ |  |
|  | ATP6AP1 |  |
|  | GDI1 |  |
|  | FAM50A |  |
|  | PLXNA3 |  |
|  | SLC10A3 |  |
|  | FAM3A |  |
|  | G6PD |  |
|  | CTAG1A |  |
|  | CTAG1B |  |
|  | CTAG2 |  |
|  | GAB3 |  |
|  | DKC1 |  |
|  | MPP1 |  |
|  | F8 |  |
|  | H2AFB1 |  |
|  | FUNDC2 |  |
|  | CMC4 |  |
|  | MTCP1 |  |
|  | BRCC3 |  |
|  | RAB39B |  |
|  | H2AFB2 |  |
|  | H2AFB3 |  |
|  | TMLHE |  |
|  | SPRY3 |  |
|  | VAMP7 |  |
|  | IL9R |  |
|  | SRY |  |
|  | ZFY |  |

|  | TGIF2LY |  |
| --- | --- | --- |
|  | PCDH11Y |  |
|  | TSPY2 |  |
|  | AMELY |  |
|  | TBL1Y |  |
|  | TSPY4 |  |
|  | TSPY8 |  |
|  | TSPY3 |  |
|  | TSPY1 |  |
|  | TSPY10 |  |
|  | USP9Y |  |
|  | UTY |  |
|  | NLGN4Y |  |
|  | KDM5D |  |
|  | PRY2 |  |
|  | PRY |  |
|  | BPY2 |  |
|  | DAZ1 |  |
|  | DAZ2 |  |
|  | BPY2B |  |
|  | DAZ3 |  |
|  | DAZ4 |  |
|  | BPY2C |  |
|  | LOC389831 |  |
|  | LOC100288966 |  |
|  | UBXN2B |  |
|  | CYP7A1 |  |
|  | CNBD1 |  |
|  | DCAF4L2 |  |
|  | MMP16 |  |
|  | NBN |  |
|  | DECR1 |  |
|  | TMEM64 |  |
|  | NECAB1 |  |
|  | PIP4P2 |  |
|  | LRRC69 |  |
|  | SLC26A7 |  |
|  | RUNX1T1 |  |
|  | TRIQK |  |
|  | FAM92A |  |
|  | RBM12B |  |
|  | TMEM67 |  |
|  | PDP1 |  |
|  | CDH17 |  |
|  | RAD54B |  |
|  | FSBP |  |
|  | VIRMA |  |
|  | ESRP1 |  |
|  | DPY19L4 |  |
|  | INTS8 |  |
|  | CCNE2 |  |
|  | NDUFAF6 |  |
|  | PLEKHF2 |  |

|  | C8orf37 |  |
| --- | --- | --- |
|  | GDF6 |  |
|  | UQCRB |  |
|  | PTDSS1 |  |
|  | SDC2 |  |
|  | CPQ |  |
|  | TSPYL5 |  |
|  | MTDH |  |
|  | LAPTM4B |  |
|  | MATN2 |  |
|  | ERICH5 |  |
|  | POP1 |  |
|  | NIPAL2 |  |
|  | KCNS2 |  |
|  | STK3 |  |
|  | OSR2 |  |
|  | VPS13B |  |
|  | COX6C |  |
|  | RGS22 |  |
|  | FBXO43 |  |
|  | RNF19A |  |
|  | ANKRD46 |  |
|  | PABPC1 |  |
|  | YWHAZ |  |
|  | ZNF706 |  |
|  | GRHL2 |  |
|  | NCALD |  |
|  | RRM2B |  |
|  | UBR5 |  |
|  | ODF1 |  |
|  | KLF10 |  |
|  | AZIN1 |  |
|  | ATP6V1C1 |  |
|  | BAALC |  |
|  | FZD6 |  |
|  | CTHRC1 |  |
|  | SLC25A32 |  |
|  | DCAF13 |  |
|  | RIMS2 |  |
|  | DCSTAMP |  |
|  | DPYS |  |
|  | LRP12 |  |
|  | ZFPM2 |  |
|  | OXR1 |  |
|  | ABRA |  |
|  | ANGPT1 |  |
|  | RSPO2 |  |
|  | EMC2 |  |
|  | TMEM74 |  |
|  | NUDCD1 |  |
|  | ENY2 |  |
|  | PKHD1L1 |  |
|  | EBAG9 |  |

|  | SYBU |  |
| --- | --- | --- |
|  | KCNV1 |  |
|  | CSMD3 |  |
|  | TRPS1 |  |
|  | EIF3H |  |
|  | RAD21 |  |
|  | AARD |  |
|  | SLC30A8 |  |
|  | MED30 |  |
|  | EXT1 |  |
|  | SAMD12 |  |
|  | TNFRSF11B |  |
|  | ENPP2 |  |
|  | TAF2 |  |
|  | DSCC1 |  |
|  | DEPTOR |  |
|  | COL14A1 |  |
|  | MTBP |  |
|  | SNTB1 |  |
|  | HAS2 |  |
|  | ZHX2 |  |
|  | TBC1D31 |  |
|  | FAM83A |  |
|  | C8orf76 |  |
|  | ATAD2 |  |
|  | WDYHV1 |  |
|  | FBXO32 |  |
|  | KLHL38 |  |
|  | ANXA13 |  |
|  | FER1L6 |  |
|  | TRMT12 |  |
|  | MTSS1 |  |
|  | SQLE |  |
|  | WASHC5 |  |
|  | TRIB1 |  |
|  | FAM84B |  |
|  | POU5F1B |  |
|  | MYC |  |
|  | GSDMC |  |
|  | FAM49B |  |
|  | ASAP1 |  |
|  | ADCY8 |  |
|  | EFR3A |  |
|  | OC90 |  |
|  | HHLA1 |  |
|  | KCNQ3 |  |
|  | LRRC6 |  |
|  | PHF20L1 |  |
|  | TG |  |
|  | SLA |  |
|  | WISP1 |  |
|  | NDRG1 |  |
|  | ST3GAL1 |  |

|  | ZFAT |  |
| --- | --- | --- |
|  | KHDRBS3 |  |
|  | FAM135B |  |
|  | COL22A1 |  |
|  | KCNK9 |  |
|  | TRAPPC9 |  |
|  | CHRAC1 |  |
|  | AGO2 |  |
|  | PTK2 |  |
|  | DENND3 |  |
|  | SLC45A4 |  |
|  | GPR20 |  |
|  | PTP4A3 |  |
|  | TSNARE1 |  |
|  | ADGRB1 |  |
|  | ARC |  |
|  | JRK |  |
|  | PSCA |  |
|  | LY6K |  |
|  | THEM6 |  |
|  | LYNX1-SLURP2 |  |
|  | LYNX1 |  |
|  | HPSE |  |
|  | ABRAXAS1 |  |
|  | GPAT3 |  |
|  | NKX6-1 |  |
|  | WDFY3 |  |
|  | ARHGAP24 |  |
|  | MAPK10 |  |
|  | PTPN13 |  |
|  | AFF1 |  |
|  | KLHL8 |  |
|  | HSD17B13 |  |
|  | SPARCL1 |  |
|  | DMP1 |  |
|  | MEPE |  |
|  | SPP1 |  |
|  | PKD2 |  |
|  | ABCG2 |  |
|  | PPM1K |  |
|  | HERC6 |  |
|  | PYURF |  |
|  | PIGY |  |
|  | HERC3 |  |
|  | NAP1L5 |  |
|  | FAM13A |  |
|  | GPRIN3 |  |
|  | SNCA |  |
|  | MMRN1 |  |
|  | CCSER1 |  |
|  | GRID2 |  |
|  | ATOH1 |  |
|  | SMARCAD1 |  |

|  | PDLIM5 |  |
| --- | --- | --- |
|  | BMPR1B |  |
|  | UNC5C |  |
|  | STPG2 |  |
|  | RAP1GDS1 |  |
|  | EIF4E |  |
|  | METAP1 |  |
|  | ADH5 |  |
|  | ADH6 |  |
|  | ADH1A |  |
|  | ADH1C |  |
|  | C4orf17 |  |
|  | TRMT10A |  |
|  | MTTP |  |
|  | C4orf54 |  |
|  | LAMTOR3 |  |
|  | DNAJB14 |  |
|  | DDIT4L |  |
|  | EMCN |  |
|  | PPP3CA |  |
|  | BANK1 |  |
|  | NFKB1 |  |
|  | MANBA |  |
|  | UBE2D3 |  |
|  | CISD2 |  |
|  | SLC9B2 |  |
|  | BDH2 |  |
|  | CENPE |  |
|  | TACR3 |  |
|  | CXXC4 |  |
|  | TET2 |  |
|  | PPA2 |  |
|  | ARHGEF38 |  |
|  | INTS12 |  |
|  | GSTCD |  |
|  | TBCK |  |
|  | AIMP1 |  |
|  | DKK2 |  |
|  | SGMS2 |  |
|  | LEF1 |  |
|  | OSTC |  |
|  | ETNPPL |  |
|  | COL25A1 |  |
|  | SEC24B |  |
|  | CASP6 |  |
|  | PLA2G12A |  |
|  | CFI |  |
|  | RRH |  |
|  | LRIT3 |  |
|  | EGF |  |
|  | ELOVL6 |  |
|  | PITX2 |  |
|  | AP1AR |  |

|  | TIFA |  |
| --- | --- | --- |
|  | NEUROG2 |  |
|  | ZGRF1 |  |
|  | LARP7 |  |
|  | ANK2 |  |
|  | CAMK2D |  |
|  | ARSJ |  |
|  | UGT8 |  |
|  | NDST4 |  |
|  | TRAM1L1 |  |
|  | NDST3 |  |
|  | METTL14 |  |
|  | SEC24D |  |
|  | SYNPO2 |  |
|  | C4orf3 |  |
|  | FABP2 |  |
|  | PDE5A |  |
|  | PRDM5 |  |
|  | NDNF |  |
|  | TNIP3 |  |
|  | QRFPR |  |
|  | ANXA5 |  |
|  | EXOSC9 |  |
|  | CCNA2 |  |
|  | BBS7 |  |
|  | XRRA1 |  |
|  | NEU3 |  |
|  | OR2AT4 |  |
|  | SLCO2B1 |  |
|  | PSMD14 |  |
|  | SLC4A10 |  |
|  | DPP4 |  |
|  | FAP |  |
|  | GCA |  |
|  | KCNH7 |  |
|  | FIGN |  |
|  | GRB14 |  |
|  | COBLL1 |  |
|  | SLC38A11 |  |
|  | SCN3A |  |
|  | SCN2A |  |
|  | CSRNP3 |  |
|  | GALNT3 |  |
|  | SCN1A |  |
|  | SCN9A |  |
|  | SCN7A |  |
|  | XIRP2 |  |
|  | B3GALT1 |  |
|  | CERS6 |  |
|  | NOSTRIN |  |
|  | G6PC2 |  |
|  | ABCB11 |  |
|  | DHRS9 |  |

|  | LRP2 |  |
| --- | --- | --- |
|  | BBS5 |  |
|  | KLHL41 |  |
|  | FASTKD1 |  |
|  | CCDC173 |  |
|  | PHOSPHO2-KLHL23 |  |
|  | SSB |  |
|  | METTL5 |  |
|  | UBR3 |  |
|  | MYO3B |  |
|  | SP5 |  |
|  | GAD1 |  |
|  | GORASP2 |  |
|  | TLK1 |  |
|  | METTL8 |  |
|  | DCAF17 |  |
|  | CYBRD1 |  |
|  | DYNC1I2 |  |
|  | SLC25A12 |  |
|  | DLX1 |  |
|  | DLX2 |  |
|  | RAPGEF4 |  |
|  | MAP3K20 |  |
|  | SP3 |  |
|  | OLA1 |  |
|  | SP9 |  |
|  | CIR1 |  |
|  | SCRN3 |  |
|  | GPR155 |  |
|  | WIPF1 |  |
|  | CHRNA1 |  |
|  | CHN1 |  |
|  | ATF2 |  |
|  | ATP5MC3 |  |
|  | LNPK |  |
|  | HOXD13 |  |
|  | HOXD12 |  |
|  | HOXD11 |  |
|  | HOXD10 |  |
|  | HOXD9 |  |
|  | HOXD8 |  |
|  | HOXD3 |  |
|  | HOXD1 |  |
|  | MTX2 |  |
|  | HNRNPA3 |  |
|  | NFE2L2 |  |
|  | AGPS |  |
|  | TTC30B |  |
|  | TTC30A |  |
|  | PDE11A |  |
|  | OSBPL6 |  |
|  | PJVK |  |
|  | FKBP7 |  |

|  | TTN |  |
| --- | --- | --- |
|  | CCDC141 |  |
|  | SESTD1 |  |
|  | ZNF385B |  |
|  | CWC22 |  |
|  | UBE2E3 |  |
|  | ITGA4 |  |
|  | NEUROD1 |  |
|  | SSFA2 |  |
|  | PPP1R1C |  |
|  | PDE1A |  |
|  | DNAJC10 |  |
|  | NCKAP1 |  |
|  | DUSP19 |  |
|  | NUP35 |  |
|  | ZNF804A |  |
|  | FSIP2 |  |
|  | ITGAV |  |
|  | FAM171B |  |
|  | ZSWIM2 |  |
|  | CALCRL |  |
|  | GULP1 |  |
|  | COL3A1 |  |
|  | WDR75 |  |
|  | SLC40A1 |  |
|  | ASNSD1 |  |
|  | ASDURF |  |
|  | ANKAR |  |
|  | OSGEPL1 |  |
|  | ORMDL1 |  |
|  | PMS1 |  |
|  | MSTN |  |
|  | C2orf88 |  |
|  | HIBCH |  |
|  | INPP1 |  |
|  | MFSD6 |  |
|  | NEMP2 |  |
|  | NAB1 |  |
|  | STAT1 |  |
|  | STAT4 |  |
|  | MYO1B |  |
|  | NABP1 |  |
|  | CAVIN2 |  |
|  | TMEFF2 |  |
|  | SLC39A10 |  |
|  | DNAH7 |  |
|  | STK17B |  |
|  | WWP1 |  |
|  | RMDN1 |  |
|  | CPNE3 |  |
|  | CNGB3 |  |
|  | DDX51 |  |
|  | NOC4L |  |

|  | GALNT9 |  |
| --- | --- | --- |
|  | FBRSL1 |  |
|  | P2RX2 |  |
|  | POLE |  |
|  | PXMP2 |  |
|  | PGAM5 |  |
|  | ANKLE2 |  |
|  | GOLGA3 |  |
|  | ZNF605 |  |
|  | ZNF26 |  |
|  | ZNF84 |  |
|  | ZNF140 |  |
|  | ZNF268 |  |
|  | ANHX |  |
|  | TUBA3C |  |
|  | TPTE2 |  |
|  | MPHOSPH8 |  |
|  | PSPC1 |  |
|  | ZMYM5 |  |
|  | ZMYM2 |  |
|  | GJA3 |  |
|  | GJB6 |  |
|  | CRYL1 |  |
|  | IFT88 |  |
|  | EEF1AKMT1 |  |
|  | XPO4 |  |
|  | SAP18 |  |
|  | SKA3 |  |
|  | MRPL57 |  |
|  | ZDHHC20 |  |
|  | SGCG |  |
|  | SACS |  |
|  | TNFRSF19 |  |
|  | MIPEP |  |
|  | SPATA13 |  |
|  | PARP4 |  |
|  | RNF17 |  |
|  | CENPJ |  |
|  | PABPC3 |  |
|  | AMER2 |  |
|  | MTMR6 |  |
|  | NUP58 |  |
|  | ATP8A2 |  |
|  | SHISA2 |  |
|  | RNF6 |  |
|  | CDK8 |  |
|  | WASF3 |  |
|  | GPR12 |  |
|  | USP12 |  |
|  | RASL11A |  |
|  | GTF3A |  |
|  | MTIF3 |  |
|  | LNX2 |  |

|  | POLR1D |  |
| --- | --- | --- |
|  | GSX1 |  |
|  | PDX1 |  |
|  | CDX2 |  |
|  | URAD |  |
|  | PAN3 |  |
|  | FLT1 |  |
|  | POMP |  |
|  | SLC46A3 |  |
|  | MTUS2 |  |
|  | SLC7A1 |  |
|  | UBL3 |  |
|  | KATNAL1 |  |
|  | UBE2L5 |  |
|  | HMGB1 |  |
|  | USPL1 |  |
|  | HSPH1 |  |
|  | B3GLCT |  |
|  | RXFP2 |  |
|  | FRY |  |
|  | ZAR1L |  |
|  | BRCA2 |  |
|  | N4BP2L1 |  |
|  | N4BP2L2 |  |
|  | KL |  |
|  | STARD13 |  |
|  | RFC3 |  |
|  | NBEA |  |
|  | DCLK1 |  |
|  | CCDC169-SOHLH2 |  |
|  | SOHLH2 |  |
|  | CCDC169 |  |
|  | SPART |  |
|  | CCNA1 |  |
|  | SERTM1 |  |
|  | RFXAP |  |
|  | EXOSC8 |  |
|  | SUPT20H |  |
|  | CSNK1A1L |  |
|  | POSTN |  |
|  | TRPC4 |  |
|  | UFM1 |  |
|  | FREM2 |  |
|  | STOML3 |  |
|  | PROSER1 |  |
|  | NHLRC3 |  |
|  | LHFPL6 |  |
|  | COG6 |  |
|  | FOXO1 |  |
|  | SLC25A15 |  |
|  | ELF1 |  |
|  | KBTBD6 |  |
|  | KBTBD7 |  |

|  | MTRF1 |  |
| --- | --- | --- |
|  | NAA16 |  |
|  | RGCC |  |
|  | VWA8 |  |
|  | DGKH |  |
|  | AKAP11 |  |
|  | TNFSF11 |  |
|  | EPSTI1 |  |
|  | ENOX1 |  |
|  | LACC1 |  |
|  | SERP2 |  |
|  | TSC22D1 |  |
|  | NUFIP1 |  |
|  | GPALPP1 |  |
|  | GTF2F2 |  |
|  | TPT1 |  |
|  | SLC25A30 |  |
|  | SPERT |  |
|  | SIAH3 |  |
|  | ZC3H13 |  |
|  | CPB2 |  |
|  | LCP1 |  |
|  | RUBCNL |  |
|  | LRCH1 |  |
|  | ESD |  |
|  | HTR2A |  |
|  | NUDT15 |  |
|  | LPAR6 |  |
|  | RCBTB2 |  |
|  | CYSLTR2 |  |
|  | FNDC3A |  |
|  | CDADC1 |  |
|  | CAB39L |  |
|  | SETDB2 |  |
|  | PHF11 |  |
|  | RCBTB1 |  |
|  | SPRYD7 |  |
|  | TRIM13 |  |
|  | KCNRG |  |
|  | DLEU7 |  |
|  | RNASEH2B |  |
|  | FAM124A |  |
|  | SERPINE3 |  |
|  | INTS6 |  |
|  | DHRS12 |  |
|  | CCDC70 |  |
|  | ATP7B |  |
|  | UTP14C |  |
|  | NEK5 |  |
|  | THSD1 |  |
|  | VPS36 |  |
|  | ZNF529 |  |
|  | ZNF382 |  |

|  | ZNF461 |  |
| --- | --- | --- |
|  | ZNF850 |  |
|  | ZNF345 |  |
|  | ZNF829 |  |
|  | ZNF585A |  |
|  | ZNF585B |  |
|  | ZNF383 |  |
|  | HKR1 |  |
|  | ZNF527 |  |
|  | ZNF569 |  |
|  | ZNF570 |  |
|  | ZNF793 |  |
|  | ZNF540 |  |
|  | ZNF571 |  |
|  | ZFP30 |  |
|  | ZNF607 |  |
|  | ZNF573 |  |
|  | WDR87 |  |
|  | SIPA1L3 |  |
|  | DPF1 |  |
|  | SPINT2 |  |
|  | YIF1B |  |
|  | C19orf33 |  |
|  | KCNK6 |  |
|  | CATSPERG |  |
|  | PSMD8 |  |
|  | SPRED3 |  |
|  | FAM98C |  |
|  | RASGRP4 |  |
|  | RYR1 |  |
|  | MAP4K1 |  |
|  | EIF3K |  |
|  | ACTN4 |  |
|  | CAPN12 |  |
|  | LGALS4 |  |
|  | ECH1 |  |
|  | HNRNPL |  |
|  | SIRT2 |  |
|  | CCER2 |  |
|  | SARS2 |  |
|  | MRPS12 |  |
|  | FBXO17 |  |
|  | FBXO27 |  |
|  | ACP7 |  |
|  | PAK4 |  |
|  | NCCRP1 |  |
|  | SYCN |  |
|  | IFNL3 |  |
|  | IFNL1 |  |
|  | LRFN1 |  |
|  | GMFG |  |
|  | SAMD4B |  |
|  | PAF1 |  |

|  | MED29 |  |
| --- | --- | --- |
|  | ZFP36 |  |
|  | PLEKHG2 |  |
|  | TIMM50 |  |
|  | DLL3 |  |
|  | SELENOV |  |
|  | EID2B |  |
|  | LGALS13 |  |
|  | LEUTX |  |
|  | DYRK1B |  |
|  | FBL |  |
|  | FCGBP |  |
|  | PSMC4 |  |
|  | ZNF546 |  |
|  | ZNF780B |  |
|  | ZNF780A |  |
|  | MAP3K10 |  |
|  | CNTD2 |  |
|  | AKT2 |  |
|  | C19orf47 |  |
|  | PLD3 |  |
|  | HIPK4 |  |
|  | PRX |  |
|  | SERTAD1 |  |
|  | SERTAD3 |  |
|  | BLVRB |  |
|  | SPTBN4 |  |
|  | SHKBP1 |  |
|  | NUMBL |  |
|  | COQ8B |  |
|  | ITPKC |  |
|  | C19orf54 |  |
|  | SNRPA |  |
|  | RAB4B |  |
|  | EGLN2 |  |
|  | CYP2A6 |  |
|  | CYP2A7 |  |
|  | CYP2B6 |  |
|  | CYP2A13 |  |
|  | CYP2F1 |  |
|  | AXL |  |
|  | HNRNPUL1 |  |
|  | CCDC97 |  |
|  | TGFB1 |  |
|  | B9D2 |  |
|  | TMEM91 |  |
|  | EXOSC5 |  |
|  | BCKDHA |  |
|  | B3GNT8 |  |
|  | DMAC2 |  |
|  | CEACAM21 |  |
|  | CEACAM4 |  |
|  | CEACAM7 |  |

|  | CEACAM5 |  |
| --- | --- | --- |
|  | CEACAM6 |  |
|  | CEACAM3 |  |
|  | LYPD4 |  |
|  | DMRTC2 |  |
|  | CD79A |  |
|  | ARHGEF1 |  |
|  | RABAC1 |  |
|  | ATP1A3 |  |
|  | GRIK5 |  |
|  | ZNF574 |  |
|  | POU2F2 |  |
|  | ZNF526 |  |
|  | GSK3A |  |
|  | ERF |  |
|  | CIC |  |
|  | PAFAH1B3 |  |
|  | PRR19 |  |
|  | TMEM145 |  |
|  | MEGF8 |  |
|  | CNFN |  |
|  | LIPE |  |
|  | CXCL17 |  |
|  | CEACAM1 |  |
|  | CEACAM8 |  |
|  | PSG8 |  |
|  | PSG1 |  |
|  | PSG6 |  |
|  | PSG7 |  |
|  | PSG11 |  |
|  | PSG2 |  |
|  | PSG4 |  |
|  | PSG9 |  |
|  | CD177 |  |
|  | TEX101 |  |
|  | LYPD3 |  |
|  | PHLDB3 |  |
|  | ZNF575 |  |
|  | XRCC1 |  |
|  | PINLYP |  |
|  | IRGQ |  |
|  | ZNF576 |  |
|  | ZNF428 |  |
|  | SRRM5 |  |
|  | CADM4 |  |
|  | PLAUR |  |
|  | IRGC |  |
|  | SMG9 |  |
|  | LYPD5 |  |
|  | ZNF283 |  |
|  | ZNF45 |  |
|  | ZNF221 |  |
|  | ZNF155 |  |

|  | ZNF230 |  |
| --- | --- | --- |
|  | ZNF222 |  |
|  | ZNF223 |  |
|  | ZNF284 |  |
|  | ZNF224 |  |
|  | ZNF225 |  |
|  | ZNF234 |  |
|  | ZNF226 |  |
|  | ZNF227 |  |
|  | ZNF233 |  |
|  | ZNF235 |  |
|  | ZNF112 |  |
|  | ZNF285 |  |
|  | ZNF229 |  |
|  | CEACAM20 |  |
|  | IGSF23 |  |
|  | PVR |  |
|  | CEACAM19 |  |
|  | BCL3 |  |
|  | CBLC |  |
|  | BCAM |  |
|  | TOMM40 |  |
|  | APOE |  |
|  | APOC1 |  |
|  | APOC2 |  |
|  | PPM1H |  |
|  | AVPR1A |  |
|  | DPY19L2 |  |
|  | RXYLT1 |  |
|  | SRGAP1 |  |
|  | C12orf66 |  |
|  | C12orf56 |  |
|  | TBK1 |  |
|  | RASSF3 |  |
|  | GNS |  |
|  | TBC1D30 |  |
|  | WIF1 |  |
|  | LEMD3 |  |
|  | MSRB3 |  |
|  | HMGA2 |  |
|  | TMBIM4 |  |
|  | IRAK3 |  |
|  | GRIP1 |  |
|  | CAND1 |  |
|  | DYRK2 |  |
|  | NUP107 |  |
|  | SLC35E3 |  |
|  | MDM2 |  |
|  | CPM |  |
|  | LYZ |  |
|  | YEATS4 |  |
|  | FRS2 |  |
|  | CCT2 |  |

|  | LRRC10 |  |
| --- | --- | --- |
|  | BEST3 |  |
|  | RAB3IP |  |
|  | MYRFL |  |
|  | CNOT2 |  |
|  | KCNMB4 |  |
|  | PTPRB |  |
|  | PTPRR |  |
|  | LGR5 |  |
|  | ZFC3H1 |  |
|  | THAP2 |  |
|  | TMEM19 |  |
|  | RAB21 |  |
|  | TBC1D15 |  |
|  | TPH2 |  |
|  | TRHDE |  |
|  | ATXN7L3B |  |
|  | KCNC2 |  |
|  | GLIPR1 |  |
|  | KRR1 |  |
|  | PHLDA1 |  |
|  | NAP1L1 |  |
|  | OSBPL8 |  |
|  | CSRP2 |  |
|  | E2F7 |  |
|  | NAV3 |  |
|  | SYT1 |  |
|  | PAWR |  |
|  | PPP1R12A |  |
|  | OTOGL |  |
|  | PTPRQ |  |
|  | MYF5 |  |
|  | LIN7A |  |
|  | ACSS3 |  |
|  | PPFIA2 |  |
|  | TMTC2 |  |
|  | SLC6A15 |  |
|  | LRRIQ1 |  |
|  | NTS |  |
|  | MGAT4C |  |
|  | C12orf50 |  |
|  | C12orf29 |  |
|  | CEP290 |  |
|  | TMTC3 |  |
|  | KITLG |  |
|  | DUSP6 |  |
|  | POC1B |  |
|  | POC1B-GALNT4 |  |
|  | GALNT4 |  |
|  | ATP2B1 |  |
|  | CCER1 |  |
|  | EPYC |  |
|  | KERA |  |

|  | DCN |  |
| --- | --- | --- |
|  | BTG1 |  |
|  | C12orf74 |  |
|  | NUDT4 |  |
|  | MRPL42 |  |
|  | SOCS2 |  |
|  | CRADD |  |
|  | PLXNC1 |  |
|  | CEP83 |  |
|  | TMCC3 |  |
|  | NR2C1 |  |
|  | FGD6 |  |
|  | VEZT |  |
|  | USP44 |  |
|  | NTN4 |  |
|  | CCDC38 |  |
|  | HAL |  |
|  | LTA4H |  |
|  | CDK17 |  |
|  | CFAP54 |  |
|  | NEDD1 |  |
|  | TMPO |  |
|  | SLC25A3 |  |
|  | IKBIP |  |
|  | APAF1 |  |
|  | ANKS1B |  |
|  | FAM71C |  |
|  | UHRF1BP1L |  |
|  | ACTR6 |  |
|  | DEPDC4 |  |
|  | SCYL2 |  |
|  | SLC17A8 |  |
|  | GAS2L3 |  |
|  | ANO4 |  |
|  | UTP20 |  |
|  | SPIC |  |
|  | MYBPC1 |  |
|  | DRAM1 |  |
|  | NUP37 |  |
|  | PARPBP |  |
|  | IGF1 |  |
|  | PAH |  |
|  | C12orf42 |  |
|  | STAB2 |  |
|  | NT5DC3 |  |
|  | C12orf73 |  |
|  | TDG |  |
|  | HCFC2 |  |
|  | NFYB |  |
|  | TXNRD1 |  |
|  | CHST11 |  |
|  | SLC41A2 |  |
|  | ALDH1L2 |  |

|  | APPL2 |  |
| --- | --- | --- |
|  | NUAK1 |  |
|  | CKAP4 |  |
|  | TCP11L2 |  |
|  | POLR3B |  |
|  | RIC8B |  |
|  | TMEM263 |  |
|  | PPIP5K2 |  |
|  | C5orf30 |  |
|  | NUDT12 |  |
|  | EFNA5 |  |
|  | FER |  |
|  | PJA2 |  |
|  | TMEM232 |  |
|  | SLC25A46 |  |
|  | WDR36 |  |
|  | CAMK4 |  |
|  | STARD4 |  |
|  | NREP |  |
|  | EPB41L4A |  |
|  | APC |  |
|  | SRP19 |  |
|  | DCP2 |  |
|  | MCC |  |
|  | TSSK1B |  |
|  | YTHDC2 |  |
|  | KCNN2 |  |
|  | TRIM36 |  |
|  | CCDC112 |  |
|  | FEM1C |  |
|  | TMED7-TICAM2 |  |
|  | TMED7 |  |
|  | CDO1 |  |
|  | ATG12 |  |
|  | AP3S1 |  |
|  | ARL14EPL |  |
|  | COMMD10 |  |
|  | SEMA6A |  |
|  | DTWD2 |  |
|  | DMXL1 |  |
|  | TNFAIP8 |  |
|  | HSD17B4 |  |
|  | PRR16 |  |
|  | SRFBP1 |  |
|  | SNCAIP |  |
|  | SNX2 |  |
|  | PRDM6 |  |
|  | CEP120 |  |
|  | CSNK1G3 |  |
|  | ZNF608 |  |
|  | GRAMD2B |  |
|  | ALDH7A1 |  |
|  | PHAX |  |

|  | TEX43 |  |
| --- | --- | --- |
|  | LMNB1 |  |
|  | 3-Mar |  |
|  | C5orf63 |  |
|  | MEGF10 |  |
|  | CTXN3 |  |
|  | CCDC192 |  |
|  | SLC12A2 |  |
|  | SLC27A6 |  |
|  | ISOC1 |  |
|  | CHSY3 |  |
|  | LYRM7 |  |
|  | RAPGEF6 |  |
|  | FNIP1 |  |
|  | MEIKIN |  |
|  | ACSL6 |  |
|  | IL3 |  |
|  | CSF2 |  |
|  | P4HA2 |  |
|  | PDLIM4 |  |
|  | SLC22A5 |  |
|  | IRF1 |  |
|  | RAD50 |  |
|  | IL13 |  |
|  | IL4 |  |
|  | KIF3A |  |
|  | CCNI2 |  |
|  | 8-Sep |  |
|  | SOWAHA |  |
|  | SHROOM1 |  |
|  | GDF9 |  |
|  | UQCRQ |  |
|  | AFF4 |  |
|  | ZCCHC10 |  |
|  | HSPA4 |  |
|  | FSTL4 |  |
|  | C5orf15 |  |
|  | VDAC1 |  |
|  | TCF7 |  |
|  | SKP1 |  |
|  | PPP2CA |  |
|  | CDKL3 |  |
|  | UBE2B |  |
|  | CDKN2AIPNL |  |
|  | JADE2 |  |
|  | SAR1B |  |
|  | SEC24A |  |
|  | CAMLG |  |
|  | DDX46 |  |
|  | PCBD2 |  |
|  | PITX1 |  |
|  | H2AFY |  |
|  | DCANP1 |  |

|  | TIFAB |  |
| --- | --- | --- |
|  | NEUROG1 |  |
|  | CXCL14 |  |
|  | SLC25A48 |  |
|  | IL9 |  |
|  | SMAD5 |  |
|  | TRPC7 |  |
|  | SPOCK1 |  |
|  | KLHL3 |  |
|  | HNRNPA0 |  |
|  | MYOT |  |
|  | PKD2L2 |  |
|  | FAM13B |  |
|  | WNT8A |  |
|  | NME5 |  |
|  | BRD8 |  |
|  | KIF20A |  |
|  | CDC23 |  |
|  | GFRA3 |  |
|  | CDC25C |  |
|  | FAM53C |  |
|  | KDM3B |  |
|  | REEP2 |  |
|  | EGR1 |  |
|  | ETF1 |  |
|  | HSPA9 |  |
|  | LRRTM2 |  |
|  | SIL1 |  |
|  | MATR3 |  |
|  | PAIP2 |  |
|  | SLC23A1 |  |
|  | SPATA24 |  |
|  | DNAJC18 |  |
|  | ECSCR |  |
|  | TMEM173 |  |
|  | UBE2D2 |  |
|  | CXXC5 |  |
|  | PSD2 |  |
|  | NRG2 |  |
|  | PURA |  |
|  | PFDN1 |  |
|  | HBEGF |  |
|  | ANKHD1-EIF4EBP3 |  |
|  | ANKHD1 |  |
|  | EIF4EBP3 |  |
|  | SRA1 |  |
|  | SLC35A4 |  |
|  | CD14 |  |
|  | TMCO6 |  |
|  | NDUFA2 |  |
|  | WDR55 |  |
|  | DND1 |  |
|  | HARS |  |

|  | HARS2 |  |
| --- | --- | --- |
|  | LHX8 |  |
|  | SLC44A5 |  |
|  | ACADM |  |
|  | MSH4 |  |
|  | ST6GALNAC5 |  |
|  | PIGK |  |
|  | AK5 |  |
|  | ZZZ3 |  |
|  | USP33 |  |
|  | MIGA1 |  |
|  | FUBP1 |  |
|  | DNAJB4 |  |
|  | GIPC2 |  |
|  | PTGFR |  |
|  | IFI44L |  |
|  | ADGRL4 |  |
|  | ADGRL2 |  |
|  | TTLL7 |  |
|  | PRKACB |  |
|  | SAMD13 |  |
|  | DNASE2B |  |
|  | RPF1 |  |
|  | GNG5 |  |
|  | CTBS |  |
|  | SSX2IP |  |
|  | MCOLN3 |  |
|  | WDR63 |  |
|  | C1orf52 |  |
|  | BCL10 |  |
|  | DDAH1 |  |
|  | CYR61 |  |
|  | ZNHIT6 |  |
|  | COL24A1 |  |
|  | ODF2L |  |
|  | CLCA2 |  |
|  | CLCA1 |  |
|  | CLCA4 |  |
|  | SH3GLB1 |  |
|  | HS2ST1 |  |
|  | LMO4 |  |
|  | PKN2 |  |
|  | KYAT3 |  |
|  | RBMXL1 |  |
|  | GBP3 |  |
|  | GBP1 |  |
|  | GBP2 |  |
|  | GBP7 |  |
|  | GBP4 |  |
|  | GBP5 |  |
|  | GBP6 |  |
|  | LRRC8B |  |
|  | LRRC8C |  |

|  | ZNF326 |  |
| --- | --- | --- |
|  | BARHL2 |  |
|  | ZNF644 |  |
|  | TGFBR3 |  |
|  | BRDT |  |
|  | EPHX4 |  |
|  | SETSIP |  |
|  | KIAA1107 |  |
|  | GLMN |  |
|  | RPAP2 |  |
|  | GFI1 |  |
|  | EVI5 |  |
|  | FAM69A |  |
|  | MTF2 |  |
|  | TMED5 |  |
|  | BCAR3 |  |
|  | ABCA4 |  |
|  | ARHGAP29 |  |
|  | ABCD3 |  |
|  | F3 |  |
|  | CNN3 |  |
|  | TMEM56 |  |
|  | PTBP2 |  |
|  | DPYD |  |
|  | SNX7 |  |
|  | PLPPR5 |  |
|  | PLPPR4 |  |
|  | PALMD |  |
|  | FRRS1 |  |
|  | AGL |  |
|  | SLC35A3 |  |
|  | MFSD14A |  |
|  | SASS6 |  |
|  | TRMT13 |  |
|  | LRRC39 |  |
|  | DBT |  |
|  | CDC14A |  |
|  | VCAM1 |  |
|  | EXTL2 |  |
|  | SLC30A7 |  |
|  | DPH5 |  |
|  | OLFM3 |  |
|  | RNPC3 |  |
|  | AMY1A |  |
|  | AMY1B |  |
|  | AMY1C |  |
|  | PRMT6 |  |
|  | NTNG1 |  |
|  | VAV3 |  |
|  | NBPF4 |  |
|  | NBPF6 |  |
|  | FAM102B |  |
|  | HENMT1 |  |

|  | PRPF38B |  |
| --- | --- | --- |
|  | FNDC7 |  |
|  | AKNAD1 |  |
|  | GPSM2 |  |
|  | CLCC1 |  |
|  | WDR47 |  |
|  | TMEM167B |  |
|  | C1orf194 |  |
|  | KIAA1324 |  |
|  | SARS |  |
|  | CELSR2 |  |
|  | PSRC1 |  |
|  | MYBPHL |  |
|  | SORT1 |  |
|  | PSMA5 |  |
|  | SYPL2 |  |
|  | ATXN7L2 |  |
|  | CYB561D1 |  |
|  | AMIGO1 |  |
|  | GPR61 |  |
|  | GNAI3 |  |
|  | GNAT2 |  |
|  | AMPD2 |  |
|  | GSTM4 |  |
|  | GSTM2 |  |
|  | GSTM5 |  |
|  | GSTM3 |  |
|  | EPS8L3 |  |
|  | CSF1 |  |
|  | AHCYL1 |  |
|  | STRIP1 |  |
|  | ALX3 |  |
|  | UBL4B |  |
|  | SLC6A17 |  |
|  | KCNC4 |  |
|  | RBM15 |  |
|  | SLC16A4 |  |
|  | LAMTOR5 |  |
|  | PROK1 |  |
|  | KCNA10 |  |
|  | KCNA2 |  |
|  | KCNA3 |  |
|  | CD53 |  |
|  | LRIF1 |  |
|  | DRAM2 |  |
|  | DENND2D |  |
|  | CHI3L2 |  |
|  | CHIA |  |
|  | PIFO |  |
|  | OVGP1 |  |
|  | WDR77 |  |
|  | ATP5PB |  |
|  | C1orf162 |  |

|  | CCND1 |  |
| --- | --- | --- |
|  | ORAOV1 |  |
|  | FGF19 |  |
|  | FGF4 |  |
|  | FGF3 |  |
|  | ANO1 |  |
|  | FADD |  |
|  | PPFIA1 |  |
|  | CTTN |  |
|  | KCNJ15 |  |
|  | ERG |  |
|  | ETS2 |  |
|  | BRWD1 |  |
|  | LCA5L |  |
|  | SH3BGR |  |
|  | B3GALT5 |  |
|  | IGSF5 |  |
|  | DSCAM |  |
|  | BACE2 |  |
|  | MX1 |  |
|  | TMPRSS2 |  |
|  | RIPK4 |  |
|  | PRDM15 |  |
|  | GAS8 |  |
|  | PRDM7 |  |
|  | ANKK1 |  |
|  | DRD2 |  |
|  | TMPRSS5 |  |
|  | ZW10 |  |
|  | CLDN25 |  |
|  | USP28 |  |
|  | HTR3A |  |
|  | ZBTB16 |  |
|  | NNMT |  |
|  | RBM7 |  |
|  | NXPE1 |  |
|  | NXPE4 |  |
|  | CADM1 |  |
|  | BUD13 |  |
|  | ZPR1 |  |
|  | APOA5 |  |
|  | APOA1 |  |
|  | SIK3 |  |
|  | PAFAH1B2 |  |
|  | SIDT2 |  |
|  | TAGLN |  |
|  | PCSK7 |  |
|  | RNF214 |  |
|  | BACE1 |  |
|  | CEP164 |  |
|  | DSCAML1 |  |
|  | FXYD6-FXYD2 |  |
|  | FXYD2 |  |

|  | FXYD6 |  |
| --- | --- | --- |
|  | TMPRSS13 |  |
|  | IL10RA |  |
|  | TMPRSS4 |  |
|  | SCN4B |  |
|  | JAML |  |
|  | MPZL3 |  |
|  | CD3D |  |
|  | CD3G |  |
|  | UBE4A |  |
|  | KMT2A |  |
|  | TMEM25 |  |
|  | IFT46 |  |
|  | ARCN1 |  |
|  | PHLDB1 |  |
|  | TREH |  |
|  | DDX6 |  |
|  | CXCR5 |  |
|  | BCL9L |  |
|  | UPK2 |  |
|  | FOXR1 |  |
|  | CCDC84 |  |
|  | SLC37A4 |  |
|  | HYOU1 |  |
|  | VPS11 |  |
|  | HMBS |  |
|  | H2AFX |  |
|  | DPAGT1 |  |
|  | C2CD2L |  |
|  | HINFP |  |
|  | ABCG4 |  |
|  | NLRX1 |  |
|  | PDZD3 |  |
|  | CCDC153 |  |
|  | CBL |  |
|  | MCAM |  |
|  | RNF26 |  |
|  | USP2 |  |
|  | THY1 |  |
|  | NECTIN1 |  |
|  | TRIM29 |  |
|  | OAF |  |
|  | POU2F3 |  |
|  | TMEM136 |  |
|  | ARHGEF12 |  |
|  | GRIK4 |  |
|  | TBCEL |  |
|  | TECTA |  |
|  | SC5D |  |
|  | SORL1 |  |
|  | BLID |  |
|  | UBASH3B |  |
|  | CRTAM |  |

|  | JHY |  |
| --- | --- | --- |
|  | BSX |  |
|  | HSPA8 |  |
|  | CLMP |  |
|  | LOC100128242 |  |
|  | GRAMD1B |  |
|  | SCN3B |  |
|  | ZNF202 |  |
|  | OR6X1 |  |
|  | OR4D5 |  |
|  | OR6T1 |  |
|  | OR10S1 |  |
|  | OR10G9 |  |
|  | OR10G7 |  |
|  | VWA5A |  |
|  | OR8D1 |  |
|  | OR8B2 |  |
|  | OR8B3 |  |
|  | OR8B8 |  |
|  | PANX3 |  |
|  | TBRG1 |  |
|  | SIAE |  |
|  | SPA17 |  |
|  | NRGN |  |
|  | MSANTD2 |  |
|  | ROBO3 |  |
|  | ROBO4 |  |
|  | HEPACAM |  |
|  | HEPN1 |  |
|  | SLC37A2 |  |
|  | TMEM218 |  |
|  | PKNOX2 |  |
|  | FEZ1 |  |
|  | EI24 |  |
|  | STT3A |  |
|  | CHEK1 |  |
|  | PATE1 |  |
|  | PATE3 |  |
|  | PATE4 |  |
|  | HYLS1 |  |
|  | PUS3 |  |
|  | DDX25 |  |
|  | CDON |  |
|  | RPUSD4 |  |
|  | FAM118B |  |
|  | SRPRA |  |
|  | FOXRED1 |  |
|  | TIRAP |  |
|  | DCPS |  |
|  | ST3GAL4 |  |
|  | KIRREL3 |  |
|  | ETS1 |  |
|  | FLI1 |  |

|  | KCNJ1 |  |
| --- | --- | --- |
|  | KCNJ5 |  |
|  | C11orf45 |  |
|  | TP53AIP1 |  |
|  | ARHGAP32 |  |
|  | BARX2 |  |
|  | TMEM45B |  |
|  | NFRKB |  |
|  | PRDM10 |  |
|  | APLP2 |  |
|  | ST14 |  |
|  | ZBTB44 |  |
|  | ADAMTS8 |  |
|  | ADAMTS15 |  |
|  | SNX19 |  |
|  | NTM |  |
|  | OPCML |  |
|  | IGSF9B |  |
|  | JAM3 |  |
|  | NCAPD3 |  |
|  | VPS26B |  |
|  | ACAD8 |  |
|  | GLB1L3 |  |
|  | B3GAT1 |  |
|  | LOC107987373 |  |
|  | IQSEC3 |  |
|  | SLC6A12 |  |
|  | SLC6A13 |  |
|  | KDM5A |  |
|  | CCDC77 |  |
|  | DAZAP2 |  |
|  | SMAGP |  |
|  | BIN2 |  |
|  | CELA1 |  |
|  | GALNT6 |  |
|  | SLC4A8 |  |
|  | SCN8A |  |
|  | FIGNL2 |  |
|  | ANKRD33 |  |
|  | ACVR1B |  |
|  | GRASP |  |
|  | NR4A1 |  |
|  | KRT80 |  |
|  | KRT7 |  |
|  | KRT86 |  |
|  | KRT81 |  |
|  | KRT83 |  |
|  | KRT85 |  |
|  | KRT84 |  |
|  | KRT82 |  |
|  | KRT75 |  |
|  | KRT6B |  |
|  | KRT6C |  |

|  | KRT6A |  |
| --- | --- | --- |
|  | KRT71 |  |
|  | KRT74 |  |
|  | KRT72 |  |
|  | KRT73 |  |
|  | KRT2 |  |
|  | KRT77 |  |
|  | KRT76 |  |
|  | KRT3 |  |
|  | KRT4 |  |
|  | KRT79 |  |
|  | KRT78 |  |
|  | KRT8 |  |
|  | EIF4B |  |
|  | TNS2 |  |
|  | SPRYD3 |  |
|  | IGFBP6 |  |
|  | SOAT2 |  |
|  | CSAD |  |
|  | ZNF740 |  |
|  | ITGB7 |  |
|  | RARG |  |
|  | MFSD5 |  |
|  | ESPL1 |  |
|  | AAAS |  |
|  | SP7 |  |
|  | SP1 |  |
|  | AMHR2 |  |
|  | PRR13 |  |
|  | PCBP2 |  |
|  | MAP3K12 |  |
|  | TARBP2 |  |
|  | ATF7 |  |
|  | ATP5MC2 |  |
|  | CALCOCO1 |  |
|  | HOXC13 |  |
|  | HOXC12 |  |
|  | HOXC11 |  |
|  | HOXC10 |  |
|  | HOXC8 |  |
|  | HOXC4 |  |
|  | HOXC6 |  |
|  | HOXC5 |  |
|  | SMUG1 |  |
|  | CBX5 |  |
|  | NFE2 |  |
|  | COPZ1 |  |
|  | GPR84 |  |
|  | ZNF385A |  |
|  | ITGA5 |  |
|  | GTSF1 |  |
|  | PDE1B |  |
|  | PPP1R1A |  |

|  | LACRT |  |
| --- | --- | --- |
|  | TESPA1 |  |
|  | NEUROD4 |  |
|  | OR9K2 |  |
|  | OR6C74 |  |
|  | OR6C6 |  |
|  | OR6C3 |  |
|  | OR6C75 |  |
|  | OR6C2 |  |
|  | OR6C68 |  |
|  | OR6C4 |  |
|  | OR10P1 |  |
|  | METTL7B |  |
|  | ITGA7 |  |
|  | BLOC1S1 |  |
|  | RDH5 |  |
|  | CD63 |  |
|  | GDF11 |  |
|  | SARNP |  |
|  | MMP19 |  |
|  | PYM1 |  |
|  | DGKA |  |
|  | PMEL |  |
|  | CDK2 |  |
|  | RAB5B |  |
|  | SUOX |  |
|  | IKZF4 |  |
|  | RPS26 |  |
|  | ERBB3 |  |
|  | PA2G4 |  |
|  | RPL41 |  |
|  | ZC3H10 |  |
|  | ESYT1 |  |
|  | MYL6B |  |
|  | SMARCC2 |  |
|  | RNF41 |  |
|  | SLC39A5 |  |
|  | ANKRD52 |  |
|  | COQ10A |  |
|  | CS |  |
|  | CNPY2 |  |
|  | PAN2 |  |
|  | IL23A |  |
|  | STAT2 |  |
|  | APOF |  |
|  | TIMELESS |  |
|  | MIP |  |
|  | SPRYD4 |  |
|  | GLS2 |  |
|  | RBMS2 |  |
|  | BAZ2A |  |
|  | PTGES3 |  |
|  | NACA |  |

|  | HSD17B6 |  |
| --- | --- | --- |
|  | SDR9C7 |  |
|  | RDH16 |  |
|  | GPR182 |  |
|  | ZBTB39 |  |
|  | MYO1A |  |
|  | NEMP1 |  |
|  | NAB2 |  |
|  | STAT6 |  |
|  | LRP1 |  |
|  | NXPH4 |  |
|  | SHMT2 |  |
|  | NDUFA4L2 |  |
|  | R3HDM2 |  |
|  | INHBC |  |
|  | INHBE |  |
|  | GLI1 |  |
|  | ARHGAP9 |  |
|  | MARS |  |
|  | DDIT3 |  |
|  | MBD6 |  |
|  | DCTN2 |  |
|  | KIF5A |  |
|  | PIP4K2C |  |
|  | DTX3 |  |
|  | ARHGEF25 |  |
|  | SLC26A10 |  |
|  | B4GALNT1 |  |
|  | OS9 |  |
|  | AGAP2 |  |
|  | TSPAN31 |  |
|  | CDK4 |  |
|  | CYP27B1 |  |
|  | METTL1 |  |
|  | EEF1AKMT3 |  |
|  | TSFM |  |
|  | AVIL |  |
|  | LRIG3 |  |
|  | SLC16A7 |  |
|  | FAM19A2 |  |
|  | USP15 |  |
|  | MON2 |  |
|  | ERVFRD-1 |  |
|  | NEDD9 |  |
|  | TMEM170B |  |
|  | ADTRP |  |
|  | HIVEP1 |  |
|  | EDN1 |  |
|  | PHACTR1 |  |
|  | TBC1D7-LOC100130357 |  |
|  | TBC1D7 |  |
|  | GFOD1 |  |
|  | SIRT5 |  |

|  | RANBP9 |  |
| --- | --- | --- |
|  | MCUR1 |  |
|  | RNF182 |  |
|  | JARID2 |  |
|  | DTNBP1 |  |
|  | MYLIP |  |
|  | ATXN1 |  |
|  | STMND1 |  |
|  | RBM24 |  |
|  | CAP2 |  |
|  | NUP153 |  |
|  | KIF13A |  |
|  | NHLRC1 |  |
|  | TPMT |  |
|  | KDM1B |  |
|  | DEK |  |
|  | RNF144B |  |
|  | MBOAT1 |  |
|  | E2F3 |  |
|  | CDKAL1 |  |
|  | PRL |  |
|  | HDGFL1 |  |
|  | NRSN1 |  |
|  | DCDC2 |  |
|  | KAAG1 |  |
|  | MRS2 |  |
|  | GPLD1 |  |
|  | ALDH5A1 |  |
|  | KIAA0319 |  |
|  | TDP2 |  |
|  | ACOT13 |  |
|  | C6orf62 |  |
|  | GMNN |  |
|  | ARMH2 |  |
|  | RIPOR2 |  |
|  | CARMIL1 |  |
|  | SCGN |  |
|  | SLC17A1 |  |
|  | SLC17A3 |  |
|  | SLC17A2 |  |
|  | TRIM38 |  |
|  | HIST1H1A |  |
|  | HIST1H3A |  |
|  | HIST1H3B |  |
|  | HIST1H2AB |  |
|  | HIST1H2BB |  |
|  | HIST1H3C |  |
|  | HIST1H1C |  |
|  | HFE |  |
|  | HIST1H1E |  |
|  | HIST1H3E |  |
|  | HIST1H3F |  |
|  | BTN3A2 |  |

|  | BTN2A2 |  |
| --- | --- | --- |
|  | BTN3A1 |  |
|  | BTN3A3 |  |
|  | BTN2A1 |  |
|  | BTN1A1 |  |
|  | HMGN4 |  |
|  | ABT1 |  |
|  | HIST1H2AH |  |
|  | PRSS16 |  |
|  | POM121L2 |  |
|  | ZNF391 |  |
|  | ZNF184 |  |
|  | HIST1H2AI |  |
|  | HIST1H3H |  |
|  | HIST1H2AJ |  |
|  | HIST1H2BM |  |
|  | HIST1H2BN |  |
|  | HIST1H3I |  |
|  | HIST1H3J |  |
|  | HIST1H2AM |  |
|  | OR2B2 |  |
|  | OR2B6 |  |
|  | ZKSCAN8 |  |
|  | ZSCAN9 |  |
|  | ZKSCAN4 |  |
|  | ZSCAN26 |  |
|  | ZSCAN31 |  |
|  | ZKSCAN3 |  |
|  | ZSCAN12 |  |
|  | ZSCAN23 |  |
|  | GPX6 |  |
|  | GPX5 |  |
|  | ZBED9 |  |
|  | OR11A1 |  |
|  | OR2H1 |  |
|  | MAS1L |  |
|  | UBD |  |
|  | OR2H2 |  |
|  | GABBR1 |  |
|  | HLA-F |  |
|  | HLA-G |  |
|  | HLA-A |  |
|  | PPP1R11 |  |
|  | RNF39 |  |
|  | TRIM40 |  |
|  | TRIM10 |  |
|  | TRIM26 |  |
|  | TRIM39 |  |
|  | TRIM39-RPP21 |  |
|  | RPP21 |  |
|  | HLA-E |  |
|  | GNL1 |  |
|  | PRR3 |  |

|  | ABCF1 |  |
| --- | --- | --- |
|  | PPP1R10 |  |
|  | MRPS18B |  |
|  | ATAT1 |  |
|  | C6orf136 |  |
|  | DHX16 |  |
|  | PPP1R18 |  |
|  | NRM |  |
|  | MDC1 |  |
|  | TUBB |  |
|  | FLOT1 |  |
|  | IER3 |  |
|  | DDR1 |  |
|  | VARS2 |  |
|  | SFTA2 |  |
|  | DPCR1 |  |
|  | MUC21 |  |
|  | MUC22 |  |
|  | C6orf15 |  |
|  | PSORS1C1 |  |
|  | CDSN |  |
|  | PSORS1C2 |  |
|  | CCHCR1 |  |
|  | TCF19 |  |
|  | POU5F1 |  |
|  | HLA-B |  |
|  | MICA |  |
|  | MICB |  |
|  | MCCD1 |  |
|  | DDX39B |  |
|  | NFKBIL1 |  |
|  | LTA |  |
|  | TNF |  |
|  | LST1 |  |
|  | NCR3 |  |
|  | AIF1 |  |
|  | PRRC2A |  |
|  | BAG6 |  |
|  | APOM |  |
|  | C6orf47 |  |
|  | GPANK1 |  |
|  | CSNK2B |  |
|  | LY6G5B |  |
|  | GPR150 |  |
|  | RFESD |  |
|  | SPATA9 |  |
|  | RHOBTB3 |  |
|  | GLRX |  |
|  | ELL2 |  |
|  | PCSK1 |  |
|  | CAST |  |
|  | ERAP1 |  |
|  | ERAP2 |  |

|  | LNPEP |  |
| --- | --- | --- |
|  | LIX1 |  |
|  | CHD1 |  |
|  | ST8SIA4 |  |
|  | SLCO4C1 |  |
|  | SLCO6A1 |  |
|  | PAM |  |
|  | GIN1 |  |
|  | FABP3 |  |
|  | TINAGL1 |  |
|  | HCRTR1 |  |
|  | PEF1 |  |
|  | COL16A1 |  |
|  | ADGRB2 |  |
|  | SPOCD1 |  |
|  | KHDRBS1 |  |
|  | TMEM39B |  |
|  | KPNA6 |  |
|  | CCDC28B |  |
|  | IQCC |  |
|  | DCDC2B |  |
|  | TMEM234 |  |
|  | EIF3I |  |
|  | LCK |  |
|  | FAM229A |  |
|  | TSSK3 |  |
|  | BSDC1 |  |
|  | ZBTB8B |  |
|  | ZBTB8A |  |
|  | ZBTB8OS |  |
|  | RBBP4 |  |
|  | SYNC |  |
|  | KIAA1522 |  |
|  | YARS |  |
|  | S100PBP |  |
|  | FNDC5 |  |
|  | HPCA |  |
|  | TMEM54 |  |
|  | RNF19B |  |
|  | AK2 |  |
|  | AZIN2 |  |
|  | TRIM62 |  |
|  | PHC2 |  |
|  | ZSCAN20 |  |
|  | HMGB4 |  |
|  | C1orf94 |  |
|  | GJB5 |  |
|  | GJB4 |  |
|  | GJB3 |  |
|  | GJA4 |  |
|  | SMIM12 |  |
|  | DLGAP3 |  |
|  | ZMYM6 |  |

|  | ZMYM1 |  |
| --- | --- | --- |
|  | ZMYM4 |  |
|  | KIAA0319L |  |
|  | NCDN |  |
|  | PSMB2 |  |
|  | C1orf216 |  |
|  | CLSPN |  |
|  | AGO4 |  |
|  | AGO1 |  |
|  | TEKT2 |  |
|  | ADPRHL2 |  |
|  | COL8A2 |  |
|  | TRAPPC3 |  |
|  | MAP7D1 |  |
|  | THRAP3 |  |
|  | EVA1B |  |
|  | STK40 |  |
|  | OSCP1 |  |
|  | MRPS15 |  |
|  | CSF3R |  |
|  | GRIK3 |  |
|  | ZC3H12A |  |
|  | MEAF6 |  |
|  | SNIP1 |  |
|  | DNALI1 |  |
|  | GNL2 |  |
|  | RSPO1 |  |
|  | C1orf109 |  |
|  | CDCA8 |  |
|  | MANEAL |  |
|  | YRDC |  |
|  | C1orf122 |  |
|  | MTF1 |  |
|  | INPP5B |  |
|  | SF3A3 |  |
|  | UTP11 |  |
|  | POU3F1 |  |
|  | RRAGC |  |
|  | RHBDL2 |  |
|  | MACF1 |  |
|  | KIAA0754 |  |
|  | BMP8A |  |
|  | PABPC4 |  |
|  | HEYL |  |
|  | HPCAL4 |  |
|  | TRIT1 |  |
|  | MYCL |  |
|  | MFSD2A |  |
|  | CAP1 |  |
|  | PPT1 |  |
|  | RLF |  |
|  | TMCO2 |  |
|  | COL9A2 |  |

|  | SMAP2 |  |
| --- | --- | --- |
|  | ZFP69B |  |
|  | ZFP69 |  |
|  | EXO5 |  |
|  | RIMS3 |  |
|  | NFYC |  |
|  | KCNQ4 |  |
|  | CTPS1 |  |
|  | SLFNL1 |  |
|  | SCMH1 |  |
|  | EDN2 |  |
|  | HIVEP3 |  |
|  | GUCA2A |  |
|  | FOXJ3 |  |
|  | RIMKLA |  |
|  | ZMYND12 |  |
|  | PPCS |  |
|  | CCDC30 |  |
|  | YBX1 |  |
|  | CLDN19 |  |
|  | C1orf50 |  |
|  | TMEM269 |  |
|  | ERMAP |  |
|  | ZNF691 |  |
|  | SLC2A1 |  |
|  | FAM183A |  |
|  | EBNA1BP2 |  |
|  | CFAP57 |  |
|  | C1orf210 |  |
|  | TIE1 |  |
|  | MPL |  |
|  | CDC20 |  |
|  | ELOVL1 |  |
|  | MED8 |  |
|  | SZT2 |  |
|  | HYI |  |
|  | PTPRF |  |
|  | KDM4A |  |
|  | ST3GAL3 |  |
|  | ARTN |  |
|  | IPO13 |  |
|  | DPH2 |  |
|  | ATP6V0B |  |
|  | B4GALT2 |  |
|  | CCDC24 |  |
|  | SLC6A9 |  |
|  | KLF18 |  |
|  | DMAP1 |  |
|  | ERI3 |  |
|  | RNF220 |  |
|  | TMEM53 |  |
|  | ARMH1 |  |
|  | KIF2C |  |

|  | RPS8 |  |
| --- | --- | --- |
|  | CKAP2 |  |
|  | HNRNPA1L2 |  |
|  | SUGT1 |  |
|  | PRR20A |  |
|  | PRR20B |  |
|  | PRR20C |  |
|  | PRR20D |  |
|  | PRR20E |  |
|  | PCDH17 |  |
|  | DIAPH3 |  |
|  | TDRD3 |  |
|  | PCDH20 |  |
|  | LOC112267897 |  |
|  | PCDH9 |  |
|  | KLHL1 |  |
|  | DACH1 |  |
|  | MZT1 |  |
|  | DIS3 |  |
|  | PIBF1 |  |
|  | KLF5 |  |
|  | KLF12 |  |
|  | TBC1D4 |  |
|  | COMMD6 |  |
|  | LMO7 |  |
|  | KCTD12 |  |
|  | ACOD1 |  |
|  | MYCBP2 |  |
|  | SCEL |  |
|  | SLAIN1 |  |
|  | EDNRB |  |
|  | POU4F1 |  |
|  | RNF219 |  |
|  | RBM26 |  |
|  | SPRY2 |  |
|  | SLITRK1 |  |
|  | SLITRK6 |  |
|  | SLITRK5 |  |
|  | GPC5 |  |
|  | GPC6 |  |
|  | DCT |  |
|  | TGDS |  |
|  | GPR180 |  |
|  | SOX21 |  |
|  | ABCC4 |  |
|  | DZIP1 |  |
|  | DNAJC3 |  |
|  | UGGT2 |  |
|  | HS6ST3 |  |
|  | MBNL2 |  |
|  | RAP2A |  |
|  | IPO5 |  |
|  | FARP1 |  |

|  | STK24 |  |
| --- | --- | --- |
|  | DOCK9 |  |
|  | UBAC2 |  |
|  | TM9SF2 |  |
|  | ZIC5 |  |
|  | ZIC2 |  |
|  | PCCA |  |
|  | GGACT |  |
|  | TMTC4 |  |
|  | NALCN |  |
|  | ITGBL1 |  |
|  | FGF14 |  |
|  | TPP2 |  |
|  | CCDC168 |  |
|  | KDELC1 |  |
|  | BIVM |  |
|  | EFNB2 |  |
|  | ARGLU1 |  |
|  | FAM155A |  |
|  | LIG4 |  |
|  | ABHD13 |  |
|  | TNFSF13B |  |
|  | MYO16 |  |
|  | COL4A1 |  |
|  | COL4A2 |  |
|  | NAXD |  |
|  | CARS2 |  |
|  | ING1 |  |
|  | ANKRD10 |  |
|  | ARHGEF7 |  |
|  | SOX1 |  |
|  | SPACA7 |  |
|  | TUBGCP3 |  |
|  | ATP11A |  |
|  | MCF2L |  |
|  | F7 |  |
|  | F10 |  |
|  | PROZ |  |
|  | PCID2 |  |
|  | CUL4A |  |
|  | LAMP1 |  |
|  | GRTP1 |  |
|  | ADPRHL1 |  |
|  | DCUN1D2 |  |
|  | TMCO3 |  |
|  | ATP4B |  |
|  | TMEM255B |  |
|  | GAS6 |  |
|  | RASA3 |  |
|  | CFAP97D2 |  |
|  | CDC16 |  |
|  | CHAMP1 |  |
|  | OR11H12 |  |

|  | POTEM |  |
| --- | --- | --- |
|  | OR11H2 |  |
|  | OR4Q3 |  |
|  | OR4M1 |  |
|  | OR4N2 |  |
|  | OR4K3 |  |
|  | OR4K15 |  |
|  | OR11G2 |  |
|  | TTC5 |  |
|  | CCNB1IP1 |  |
|  | PARP2 |  |
|  | TEP1 |  |
|  | KLHL33 |  |
|  | OSGEP |  |
|  | APEX1 |  |
|  | PIP4P1 |  |
|  | RNASE9 |  |
|  | OR6S1 |  |
|  | ANG |  |
|  | RNASE4 |  |
|  | EDDM3B |  |
|  | RNASE1 |  |
|  | RNASE3 |  |
|  | METTL17 |  |
|  | NDRG2 |  |
|  | TPPP2 |  |
|  | RNASE7 |  |
|  | RNASE8 |  |
|  | ARHGEF40 |  |
|  | ZNF219 |  |
|  | TMEM253 |  |
|  | OR5AU1 |  |
|  | HNRNPC |  |
|  | RPGRIP1 |  |
|  | SUPT16H |  |
|  | CHD8 |  |
|  | RAB2B |  |
|  | TOX4 |  |
|  | METTL3 |  |
|  | SALL2 |  |
|  | OR10G3 |  |
|  | OR10G2 |  |
|  | OR4E1 |  |
|  | OR6J1 |  |
|  | OXA1L |  |
|  | SLC7A7 |  |
|  | MRPL52 |  |
|  | MMP14 |  |
|  | LRP10 |  |
|  | REM2 |  |
|  | RBM23 |  |
|  | HAUS4 |  |
|  | AJUBA |  |

|  | C14orf93 |  |
| --- | --- | --- |
|  | PSMB5 |  |
|  | PSMB11 |  |
|  | CDH24 |  |
|  | ACIN1 |  |
|  | RAB5C |  |
|  | KCNH4 |  |
|  | STAT5A |  |
|  | STAT3 |  |
|  | CAVIN1 |  |
|  | ATP6V0A1 |  |
|  | NAGLU |  |
|  | HSD17B1 |  |
|  | COASY |  |
|  | MLX |  |
|  | PSMC3IP |  |
|  | RETREG3 |  |
|  | TUBG2 |  |
|  | PLEKHH3 |  |
|  | CCR10 |  |
|  | CNTNAP1 |  |
|  | EZH1 |  |
|  | RAMP2 |  |
|  | WNK4 |  |
|  | CNTD1 |  |
|  | BECN1 |  |
|  | PSME3 |  |
|  | AOC2 |  |
|  | AOC3 |  |
|  | G6PC |  |
|  | PTGES3L-AARSD1 |  |
|  | AARSD1 |  |
|  | PTGES3L |  |
|  | RUNDC1 |  |
|  | VAT1 |  |
|  | RND2 |  |
|  | BRCA1 |  |
|  | NBR1 |  |
|  | TMEM106A |  |
|  | ARL4D |  |
|  | DHX8 |  |
|  | ETV4 |  |
|  | MEOX1 |  |
|  | SOST |  |
|  | DUSP3 |  |
|  | CFAP97D1 |  |
|  | MPP3 |  |
|  | CD300LG |  |
|  | MPP2 |  |
|  | PPY |  |
|  | PYY |  |
|  | NAGS |  |
|  | TMEM101 |  |

|  | LSM12 |  |
| --- | --- | --- |
|  | G6PC3 |  |
|  | HDAC5 |  |
|  | C17orf53 |  |
|  | ASB16 |  |
|  | TMUB2 |  |
|  | ATXN7L3 |  |
|  | UBTF |  |
|  | SLC4A1 |  |
|  | SLC25A39 |  |
|  | GRN |  |
|  | ITGA2B |  |
|  | GPATCH8 |  |
|  | FZD2 |  |
|  | MEIOC |  |
|  | DBF4B |  |
|  | ADAM11 |  |
|  | GJC1 |  |
|  | EFTUD2 |  |
|  | CCDC103 |  |
|  | FAM187A |  |
|  | GFAP |  |
|  | KIF18B |  |
|  | C1QL1 |  |
|  | DCAKD |  |
|  | NMT1 |  |
|  | PLCD3 |  |
|  | ACBD4 |  |
|  | HEXIM1 |  |
|  | HEXIM2 |  |
|  | FMNL1 |  |
|  | SPATA32 |  |
|  | MAP3K14 |  |
|  | ARHGAP27 |  |
|  | PLEKHM1 |  |
|  | LINC02210-CRHR1 |  |
|  | CRHR1 |  |
|  | SPPL2C |  |
|  | MAPT |  |
|  | KANSL1 |  |
|  | ARL17B |  |
|  | LRRC37A |  |
|  | LRRC37A2 |  |
|  | ARL17A |  |
|  | WNT9B |  |
|  | GOSR2 |  |
|  | RPRML |  |
|  | MYL4 |  |
|  | ITGB3 |  |
|  | EFCAB13 |  |
|  | NPEPPS |  |
|  | KPNB1 |  |
|  | TBKBP1 |  |

|  | TBX21 |  |
| --- | --- | --- |
|  | OSBPL7 |  |
|  | MRPL10 |  |
|  | LRRC46 |  |
|  | SCRN2 |  |
|  | SP6 |  |
|  | SP2 |  |
|  | PNPO |  |
|  | PRR15L |  |
|  | CDK5RAP3 |  |
|  | NFE2L1 |  |
|  | CBX1 |  |
|  | SNX11 |  |
|  | SKAP1 |  |
|  | HOXB1 |  |
|  | HOXB3 |  |
|  | HOXB4 |  |
|  | HOXB5 |  |
|  | HOXB6 |  |
|  | HOXB8 |  |
|  | HOXB9 |  |
|  | PRAC2 |  |
|  | HOXB13 |  |
|  | TTLL6 |  |
|  | CALCOCO2 |  |
|  | UBE2Z |  |
|  | IGF2BP1 |  |
|  | B4GALNT2 |  |
|  | GNGT2 |  |
|  | ABI3 |  |
|  | PHOSPHO1 |  |
|  | ZNF652 |  |
|  | NXPH3 |  |
|  | SPOP |  |
|  | FAM117A |  |
|  | KAT7 |  |
|  | TAC4 |  |
|  | DLX4 |  |
|  | DLX3 |  |
|  | ITGA3 |  |
|  | PDK2 |  |
|  | SAMD14 |  |
|  | SGCA |  |
|  | TMEM92 |  |
|  | XYLT2 |  |
|  | EME1 |  |
|  | LRRC59 |  |
|  | ACSF2 |  |
|  | CHAD |  |
|  | RSAD1 |  |
|  | MYCBPAP |  |
|  | SPATA20 |  |
|  | CACNA1G |  |

|  | ABCC3 |  |
| --- | --- | --- |
|  | ANKRD40 |  |
|  | LUC7L3 |  |
|  | LINC00483 |  |
|  | WFIKKN2 |  |
|  | TOB1 |  |
|  | PDAP1 |  |
|  | BUD31 |  |
|  | ATP5MF-PTCD1 |  |
|  | PTCD1 |  |
|  | CPSF4 |  |
|  | ZNF789 |  |
|  | ZNF394 |  |
|  | ZKSCAN5 |  |
|  | FAM200A |  |
|  | ZNF655 |  |
|  | TMEM225B |  |
|  | ZSCAN25 |  |
|  | CYP3A5 |  |
|  | CYP3A7-CYP3A51P |  |
|  | CYP3A7 |  |
|  | CYP3A4 |  |
|  | CYP3A43 |  |
|  | OR2AE1 |  |
|  | TRIM4 |  |
|  | GJC3 |  |
|  | AZGP1 |  |
|  | ZKSCAN1 |  |
|  | ZSCAN21 |  |
|  | ZNF3 |  |
|  | MCM7 |  |
|  | AP4M1 |  |
|  | TAF6 |  |
|  | CNPY4 |  |
|  | MBLAC1 |  |
|  | C7orf43 |  |
|  | GAL3ST4 |  |
|  | GPC2 |  |
|  | STAG3 |  |
|  | CASTOR3 |  |
|  | PVRIG |  |
|  | SPDYE3 |  |
|  | PILRA |  |
|  | ZCWPW1 |  |
|  | MEPCE |  |
|  | PPP1R35 |  |
|  | TSC22D4 |  |
|  | NYAP1 |  |
|  | AGFG2 |  |
|  | SAP25 |  |
|  | LRCH4 |  |
|  | FBXO24 |  |
|  | PCOLCE |  |

|  | MOSPD3 |  |
| --- | --- | --- |
|  | TFR2 |  |
|  | ACTL6B |  |
|  | EPO |  |
|  | ZAN |  |
|  | EPHB4 |  |
|  | SLC12A9 |  |
|  | TRIP6 |  |
|  | SRRT |  |
|  | ACHE |  |
|  | MUC3A |  |
|  | MUC12 |  |
|  | MUC17 |  |
|  | SERPINE1 |  |
|  | AP1S1 |  |
|  | VGF |  |
|  | MOGAT3 |  |
|  | PLOD3 |  |
|  | ZNHIT1 |  |
|  | CLDN15 |  |
|  | IFT22 |  |
|  | COL26A1 |  |
|  | MYL10 |  |
|  | CUX1 |  |
|  | SPDYE6 |  |
|  | PRKRIP1 |  |
|  | ORAI2 |  |
|  | ALKBH4 |  |
|  | LRWD1 |  |
|  | POLR2J |  |
|  | RASA4B |  |
|  | POLR2J3 |  |
|  | SPDYE2 |  |
|  | RASA4 |  |
|  | POLR2J2 |  |
|  | UPK3BL1 |  |
|  | SPDYE2B |  |
|  | FAM185A |  |
|  | FBXL13 |  |
|  | LRRC17 |  |
|  | NFE4 |  |
|  | ARMC10 |  |
|  | NAPEPLD |  |
|  | PMPCB |  |
|  | DNAJC2 |  |
|  | SLC26A5 |  |
|  | RELN |  |
|  | ORC5 |  |
|  | LHFPL3 |  |
|  | KMT2E |  |
|  | SRPK2 |  |
|  | PUS7 |  |
|  | RINT1 |  |

|  | EFCAB10 |  |
| --- | --- | --- |
|  | ATXN7L1 |  |
|  | CDHR3 |  |
|  | SYPL1 |  |
|  | NAMPT |  |
|  | CCDC71L |  |
|  | PIK3CG |  |
|  | PRKAR2B |  |
|  | HBP1 |  |
|  | COG5 |  |
|  | GPR22 |  |
|  | DUS4L |  |
|  | BCAP29 |  |
|  | SLC26A4 |  |
|  | CBLL1 |  |
|  | SLC26A3 |  |
|  | LAMB1 |  |
|  | LAMB4 |  |
|  | NRCAM |  |
|  | PNPLA8 |  |
|  | THAP5 |  |
|  | IMMP2L |  |
|  | DOCK4 |  |
|  | ZNF277 |  |
|  | TMEM168 |  |
|  | GPR85 |  |
|  | SMIM30 |  |
|  | FOXP2 |  |
|  | TFEC |  |
|  | TES |  |
|  | CAV2 |  |
|  | CAV1 |  |
|  | MET |  |
|  | CAPZA2 |  |
|  | ST7 |  |
|  | CFTR |  |
|  | CTTNBP2 |  |
|  | LSM8 |  |
|  | ANKRD7 |  |
|  | KCND2 |  |
|  | TSPAN12 |  |
|  | WNT16 |  |
|  | FAM3C |  |
|  | PTPRZ1 |  |
|  | AASS |  |
|  | FEZF1 |  |
|  | CADPS2 |  |
|  | RNF148 |  |
|  | TAS2R16 |  |
|  | SLC13A1 |  |
|  | IQUB |  |
|  | NDUFA5 |  |
|  | ASB15 |  |

|  | WASL |  |
| --- | --- | --- |
|  | HYAL4 |  |
|  | GPR37 |  |
|  | POT1 |  |
|  | GRM8 |  |
|  | ZNF800 |  |
|  | GCC1 |  |
|  | FSCN3 |  |
|  | LRRC4 |  |
|  | LEP |  |
|  | RBM28 |  |
|  | PRRT4 |  |
|  | IMPDH1 |  |
|  | HILPDA |  |
|  | METTL2B |  |
|  | FAM71F2 |  |
|  | FAM71F1 |  |
|  | CALU |  |
|  | OPN1SW |  |
|  | CCDC136 |  |
|  | FLNC |  |
|  | ATP6V1F |  |
|  | LOC100130705 |  |
|  | KCP |  |
|  | CDH19 |  |
|  | DOK6 |  |
|  | CD226 |  |
|  | RTTN |  |
|  | SOCS6 |  |
|  | CBLN2 |  |
|  | FBXO15 |  |
|  | FAM69C |  |
|  | CNDP2 |  |
|  | CNDP1 |  |
|  | ZNF407 |  |
|  | ZADH2 |  |
|  | TSHZ1 |  |
|  | SMIM21 |  |
|  | ZNF516 |  |
|  | ZNF236 |  |
|  | MBP |  |
|  | GALR1 |  |
|  | SALL3 |  |
|  | ATP9B |  |
|  | NFATC1 |  |
|  | HSBP1L1 |  |
|  | RBFA |  |
|  | ADNP2 |  |
|  | PARD6G |  |
|  | PLPP2 |  |
|  | MIER2 |  |
|  | THEG |  |
|  | C2CD4C |  |

|  | SHC2 |  |
| --- | --- | --- |
|  | ODF3L2 |  |
|  | MADCAM1 |  |
|  | CDC34 |  |
|  | BSG |  |
|  | HCN2 |  |
|  | POLRMT |  |
|  | FGF22 |  |
|  | RNF126 |  |
|  | FSTL3 |  |
|  | PRSS57 |  |
|  | MISP |  |
|  | PTBP1 |  |
|  | PLPPR3 |  |
|  | ELANE |  |
|  | CFD |  |
|  | MED16 |  |
|  | R3HDM4 |  |
|  | KISS1R |  |
|  | ARID3A |  |
|  | WDR18 |  |
|  | GRIN3B |  |
|  | CNN2 |  |
|  | ABCA7 |  |
|  | ARHGAP45 |  |
|  | POLR2E |  |
|  | SBNO2 |  |
|  | STK11 |  |
|  | CBARP |  |
|  | ATP5F1D |  |
|  | MIDN |  |
|  | C19orf24 |  |
|  | EFNA2 |  |
|  | MUM1 |  |
|  | NDUFS7 |  |
|  | GAMT |  |
|  | DAZAP1 |  |
|  | APC2 |  |
|  | C19orf25 |  |
|  | PCSK4 |  |
|  | REEP6 |  |
|  | ADAMTSL5 |  |
|  | PLK5 |  |
|  | MBD3 |  |
|  | UQCR11 |  |
|  | TCF3 |  |
|  | ONECUT3 |  |
|  | REXO1 |  |
|  | KLF16 |  |
|  | ABHD17A |  |
|  | SCAMP4 |  |
|  | CSNK1G2 |  |
|  | BTBD2 |  |

|  | MKNK2 |  |
| --- | --- | --- |
|  | MOB3A |  |
|  | AP3D1 |  |
|  | DOT1L |  |
|  | PLEKHJ1 |  |
|  | SF3A2 |  |
|  | AMH |  |
|  | JSRP1 |  |
|  | OAZ1 |  |
|  | PEAK3 |  |
|  | LSM7 |  |
|  | TIMM13 |  |
|  | LMNB2 |  |
|  | GADD45B |  |
|  | GNG7 |  |
|  | DIRAS1 |  |
|  | SLC39A3 |  |
|  | SGTA |  |
|  | THOP1 |  |
|  | ZNF554 |  |
|  | ZNF555 |  |
|  | ZNF556 |  |
|  | TLE6 |  |
|  | TLE2 |  |
|  | AES |  |
|  | GNA11 |  |
|  | S1PR4 |  |
|  | NCLN |  |
|  | CELF5 |  |
|  | NFIC |  |
|  | SMIM24 |  |
|  | DOHH |  |
|  | FZR1 |  |
|  | C19orf71 |  |
|  | MFSD12 |  |
|  | HMG20B |  |
|  | GIPC3 |  |
|  | TBXA2R |  |
|  | CACTIN |  |
|  | PIP5K1C |  |
|  | TJP3 |  |
|  | APBA3 |  |
|  | MRPL54 |  |
|  | RAX2 |  |
|  | MATK |  |
|  | ZFR2 |  |
|  | ATCAY |  |
|  | NMRK2 |  |
|  | DAPK3 |  |
|  | EEF2 |  |
|  | PIAS4 |  |
|  | ZBTB7A |  |
|  | MAP2K2 |  |

|  | CREB3L3 |  |
| --- | --- | --- |
|  | SIRT6 |  |
|  | ANKRD24 |  |
|  | EBI3 |  |
|  | SHD |  |
|  | TMIGD2 |  |
|  | FSD1 |  |
|  | STAP2 |  |
|  | MPND |  |
|  | SH3GL1 |  |
|  | CHAF1A |  |
|  | UBXN6 |  |
|  | HDGFL2 |  |
|  | PLIN4 |  |
|  | PLIN5 |  |
|  | LRG1 |  |
|  | SEMA6B |  |
|  | TNFAIP8L1 |  |
|  | MYDGF |  |
|  | DPP9 |  |
|  | FEM1A |  |
|  | TICAM1 |  |
|  | PLIN3 |  |
|  | ARRDC5 |  |
|  | UHRF1 |  |
|  | KDM4B |  |
|  | PTPRS |  |
|  | ZNRF4 |  |
|  | C19orf70 |  |
|  | HSD11B1L |  |
|  | RPL36 |  |
|  | LONP1 |  |
|  | PRR22 |  |
|  | DUS3L |  |
|  | FUT6 |  |
|  | FUT3 |  |
|  | FUT5 |  |
|  | NDUFA11 |  |
|  | VMAC |  |
|  | CAPS |  |
|  | RANBP3 |  |
|  | RFX2 |  |
|  | ACSBG2 |  |
|  | MLLT1 |  |
|  | ACER1 |  |
|  | CLPP |  |
|  | ALKBH7 |  |
|  | GTF2F1 |  |
|  | SLC25A41 |  |
|  | SLC25A23 |  |
|  | CRB3 |  |
|  | DENND1C |  |
|  | TNFSF9 |  |

|  | CD70 |  |
| --- | --- | --- |
|  | TNFSF14 |  |
|  | C3 |  |
|  | GPR108 |  |
|  | TRIP10 |  |
|  | SH2D3A |  |
|  | VAV1 |  |
|  | ADGRE1 |  |
|  | ZNF557 |  |
|  | INSR |  |
|  | ARHGEF18 |  |
|  | ZNF358 |  |
|  | MCOLN1 |  |
|  | PNPLA6 |  |
|  | CBR1 |  |
|  | CBR3 |  |
|  | ITPK1 |  |
|  | MOAP1 |  |
|  | GON7 |  |
|  | UBR7 |  |
|  | BTBD7 |  |
|  | UNC79 |  |
|  | FAM181A |  |
|  | ASB2 |  |
|  | CCDC197 |  |
|  | OTUB2 |  |
|  | DDX24 |  |
|  | IFI27L2 |  |
|  | SERPINA10 |  |
|  | SERPINA1 |  |
|  | SERPINA11 |  |
|  | SERPINA9 |  |
|  | SERPINA12 |  |
|  | SERPINA4 |  |
|  | SERPINA5 |  |
|  | GSC |  |
|  | DICER1 |  |
|  | CLMN |  |
|  | SYNE3 |  |
|  | TCL1B |  |
|  | TCL1A |  |
|  | C14orf132 |  |
|  | BDKRB2 |  |
|  | BDKRB1 |  |
|  | ATG2B |  |
|  | GSKIP |  |
|  | AK7 |  |
|  | PAPOLA |  |
|  | SETD3 |  |
|  | CCNK |  |
|  | HHIPL1 |  |
|  | EML1 |  |
|  | EVL |  |

|  | DEGS2 |  |
| --- | --- | --- |
|  | SLC25A29 |  |
|  | SLC25A47 |  |
|  | WARS |  |
|  | WDR25 |  |
|  | BEGAIN |  |
|  | DLK1 |  |
|  | RTL1 |  |
|  | DIO3 |  |
|  | PPP2R5C |  |
|  | DYNC1H1 |  |
|  | HSP90AA1 |  |
|  | WDR20 |  |
|  | MOK |  |
|  | ZNF839 |  |
|  | CINP |  |
|  | TECPR2 |  |
|  | TRAF3 |  |
|  | AMN |  |
|  | CDC42BPB |  |
|  | TNFAIP2 |  |
|  | EIF5 |  |
|  | MARK3 |  |
|  | CKB |  |
|  | TRMT61A |  |
|  | BAG5 |  |
|  | APOPT1 |  |
|  | XRCC3 |  |
|  | ZFYVE21 |  |
|  | PPP1R13B |  |
|  | RD3L |  |
|  | ASPG |  |
|  | KIF26A |  |
|  | TMEM179 |  |
|  | INF2 |  |
|  | ADSSL1 |  |
|  | SIVA1 |  |
|  | AKT1 |  |
|  | CEP170B |  |
|  | PLD4 |  |
|  | AHNAK2 |  |
|  | CDCA4 |  |
|  | GPR132 |  |
|  | JAG2 |  |
|  | BRF1 |  |
|  | BTBD6 |  |
|  | PACS2 |  |
|  | CRIP2 |  |
|  | CRIP1 |  |
|  | TEDC1 |  |
|  | GOLGA6L6 |  |
|  | POTEB2 |  |
|  | POTEB3 |  |

|  | POTEB |  |
| --- | --- | --- |
|  | OR4M2 |  |
|  | OR4N4 |  |
|  | NIPA1 |  |
|  | CYFIP1 |  |
|  | TUBGCP5 |  |
|  | GOLGA6L1 |  |
|  | GOLGA6L2 |  |
|  | MAGEL2 |  |
|  | NPAP1 |  |
|  | SNRPN |  |
|  | SNURF |  |
|  | UBE3A |  |
|  | ATP10A |  |
|  | GABRB3 |  |
|  | GABRA5 |  |
|  | GABRG3 |  |
|  | OCA2 |  |
|  | HERC2 |  |
|  | GOLGA8F |  |
|  | GOLGA8G |  |
|  | GOLGA8M |  |
|  | APBA2 |  |
|  | FAM189A1 |  |
|  | NSMCE3 |  |
|  | TJP1 |  |
|  | GOLGA8J |  |
|  | GOLGA8T |  |
|  | CHRFAM7A |  |
|  | GOLGA8R |  |
|  | GOLGA8Q |  |
|  | GOLGA8H |  |
|  | ARHGAP11B |  |
|  | FAN1 |  |
|  | MTMR10 |  |
|  | TRPM1 |  |
|  | KLF13 |  |
|  | OTUD7A |  |
|  | CHRNA7 |  |
|  | GOLGA8K |  |
|  | GOLGA8O |  |
|  | GOLGA8N |  |
|  | ARHGAP11A |  |
|  | SCG5 |  |
|  | FMN1 |  |
|  | RYR3 |  |
|  | AVEN |  |
|  | CHRM5 |  |
|  | EMC7 |  |
|  | PGBD4 |  |
|  | KATNBL1 |  |
|  | EMC4 |  |
|  | SLC12A6 |  |

|  | NUTM1 |  |
| --- | --- | --- |
|  | LPCAT4 |  |
|  | GOLGA8A |  |
|  | GOLGA8B |  |
|  | GJD2 |  |
|  | ZNF770 |  |
|  | NANOGP8 |  |
|  | C15orf41 |  |
|  | MEIS2 |  |
|  | SPRED1 |  |
|  | FAM98B |  |
|  | RASGRP1 |  |
|  | THBS1 |  |
|  | FSIP1 |  |
|  | GPR176 |  |
|  | EIF2AK4 |  |
|  | BMF |  |
|  | EIF5A |  |
|  | NEURL4 |  |
|  | ACAP1 |  |
|  | KCTD11 |  |
|  | TMEM95 |  |
|  | TNK1 |  |
|  | PLSCR3 |  |
|  | NLGN2 |  |
|  | SPEM1 |  |
|  | SPEM2 |  |
|  | TMEM102 |  |
|  | CHRNB1 |  |
|  | ZBTB4 |  |
|  | SLC35G6 |  |
|  | TNFSF12-TNFSF13 |  |
|  | TNFSF12 |  |
|  | TNFSF13 |  |
|  | SENP3 |  |
|  | CD68 |  |
|  | MPDU1 |  |
|  | SOX15 |  |
|  | FXR2 |  |
|  | SHBG |  |
|  | SAT2 |  |
|  | ATP1B2 |  |
|  | TP53 |  |
|  | WRAP53 |  |
|  | EFNB3 |  |
|  | DNAH2 |  |
|  | KDM6B |  |
|  | TMEM88 |  |
|  | CYB5D1 |  |
|  | RNF227 |  |
|  | KCNAB3 |  |
|  | CNTROB |  |
|  | GUCY2D |  |

|  | ALOX12B |  |
| --- | --- | --- |
|  | ALOXE3 |  |
|  | PER1 |  |
|  | VAMP2 |  |
|  | TMEM107 |  |
|  | BORCS6 |  |
|  | AURKB |  |
|  | CTC1 |  |
|  | PFAS |  |
|  | SLC25A35 |  |
|  | RANGRF |  |
|  | ARHGEF15 |  |
|  | KRBA2 |  |
|  | RPL26 |  |
|  | RNF222 |  |
|  | NDEL1 |  |
|  | MYH10 |  |
|  | SPDYE4 |  |
|  | MFSD6L |  |
|  | PIK3R6 |  |
|  | PIK3R5 |  |
|  | NTN1 |  |
|  | CFAP52 |  |
|  | USP43 |  |
|  | DHRS7C |  |
|  | GLP2R |  |
|  | RCVRN |  |
|  | GAS7 |  |
|  | MYH13 |  |
|  | MYH8 |  |
|  | MYH2 |  |
|  | MYH3 |  |
|  | SCO1 |  |
|  | ADPRM |  |
|  | TMEM220 |  |
|  | PIRT |  |
|  | SHISA6 |  |
|  | DNAH9 |  |
|  | ZNF18 |  |
|  | MYOCD |  |
|  | ARHGAP44 |  |
|  | HS3ST3A1 |  |
|  | COX10 |  |
|  | CDRT15 |  |
|  | HS3ST3B1 |  |
|  | PMP22 |  |
|  | TEKT3 |  |
|  | TVP23C |  |
|  | CDRT1 |  |
|  | TRIM16 |  |
|  | ZNF286A |  |
|  | TBC1D26 |  |
|  | ZSWIM7 |  |

|  | TTC19 |  |
| --- | --- | --- |
|  | NCOR1 |  |
|  | PIGL |  |
|  | CENPV |  |
|  | TRPV2 |  |
|  | LRRC75A |  |
|  | ZNF287 |  |
|  | CCDC144A |  |
|  | MPRIP |  |
|  | PLD6 |  |
|  | FLCN |  |
|  | COPS3 |  |
|  | NT5M |  |
|  | MED9 |  |
|  | RAI1 |  |
|  | SREBF1 |  |
|  | TOM1L2 |  |
|  | DRC3 |  |
|  | GID4 |  |
|  | MYO15A |  |
|  | LLGL1 |  |
|  | FLII |  |
|  | MIEF2 |  |
|  | TOP3A |  |
|  | SMCR8 |  |
|  | SHMT1 |  |
|  | LGALS9C |  |
|  | TBC1D28 |  |
|  | TRIM16L |  |
|  | FBXW10 |  |
|  | TVP23B |  |
|  | PRPSAP2 |  |
|  | SLC5A10 |  |
|  | FAM83G |  |
|  | GRAP |  |
|  | GRAPL |  |
|  | EPN2 |  |
|  | B9D1 |  |
|  | MAPK7 |  |
|  | MFAP4 |  |
|  | RNF112 |  |
|  | ALDH3A2 |  |
|  | SLC47A2 |  |
|  | ALDH3A1 |  |
|  | ULK2 |  |
|  | SPECC1 |  |
|  | LGALS9B |  |
|  | USP22 |  |
|  | DHRS7B |  |
|  | TMEM11 |  |
|  | NATD1 |  |
|  | MAP2K3 |  |
|  | KCNJ12 |  |

|  | WSB1 |  |
| --- | --- | --- |
|  | KSR1 |  |
|  | LGALS9 |  |
|  | NOS2 |  |
|  | NLK |  |
|  | TMEM97 |  |
|  | TNFAIP1 |  |
|  | POLDIP2 |  |
|  | TMEM199 |  |
|  | SEBOX |  |
|  | VTN |  |
|  | SARM1 |  |
|  | SLC46A1 |  |
|  | FOXN1 |  |
|  | UNC119 |  |
|  | PIGS |  |
|  | ALDOC |  |
|  | SPAG5 |  |
|  | SGK494 |  |
|  | KIAA0100 |  |
|  | SUPT6H |  |
|  | PROCA1 |  |
|  | RAB34 |  |
|  | RPL23A |  |
|  | TLCD1 |  |
|  | NEK8 |  |
|  | TRAF4 |  |
|  | FAM222B |  |
|  | CTNNB1 |  |
|  | TRAK1 |  |
|  | CCK |  |
|  | VIPR1 |  |
|  | SEC22C |  |
|  | SS18L2 |  |
|  | ZBTB47 |  |
|  | KLHL40 |  |
|  | HHATL |  |
|  | CCDC13 |  |
|  | HIGD1A |  |
|  | ACKR2 |  |
|  | CYP8B1 |  |
|  | ZNF662 |  |
|  | KRBOX1 |  |
|  | POMGNT2 |  |
|  | SNRK |  |
|  | ABHD5 |  |
|  | TOPAZ1 |  |
|  | TCAIM |  |
|  | ZNF445 |  |
|  | ZNF852 |  |
|  | ZNF660 |  |
|  | ZNF197 |  |
|  | ZNF35 |  |

|  | ZNF501 |  |
| --- | --- | --- |
|  | KIAA1143 |  |
|  | KIF15 |  |
|  | TGM4 |  |
|  | ZDHHC3 |  |
|  | EXOSC7 |  |
|  | CLEC3B |  |
|  | CDCP1 |  |
|  | TMEM158 |  |
|  | LARS2 |  |
|  | LIMD1 |  |
|  | SACM1L |  |
|  | SLC6A20 |  |
|  | LZTFL1 |  |
|  | CCR9 |  |
|  | FYCO1 |  |
|  | CXCR6 |  |
|  | XCR1 |  |
|  | CCR1 |  |
|  | CCR3 |  |
|  | CCR2 |  |
|  | CCR5 |  |
|  | CCRL2 |  |
|  | LTF |  |
|  | LRRC2 |  |
|  | TDGF1 |  |
|  | FAM240A |  |
|  | ALS2CL |  |
|  | PRSS50 |  |
|  | PTH1R |  |
|  | CCDC12 |  |
|  | NBEAL2 |  |
|  | SETD2 |  |
|  | KIF9 |  |
|  | KLHL18 |  |
|  | PTPN23 |  |
|  | SCAP |  |
|  | ELP6 |  |
|  | SMARCC1 |  |
|  | DHX30 |  |
|  | MAP4 |  |
|  | CAMP |  |
|  | ZNF589 |  |
|  | NME6 |  |
|  | FBXW12 |  |
|  | PLXNB1 |  |
|  | CCDC51 |  |
|  | ATRIP |  |
|  | SHISA5 |  |
|  | PFKFB4 |  |
|  | UCN2 |  |
|  | COL7A1 |  |
|  | UQCRC1 |  |

|  | TMEM89 |  |
| --- | --- | --- |
|  | SLC26A6 |  |
|  | CELSR3 |  |
|  | NCKIPSD |  |
|  | IP6K2 |  |
|  | PRKAR2A |  |
|  | ARIH2 |  |
|  | WDR6 |  |
|  | DALRD3 |  |
|  | NDUFAF3 |  |
|  | IMPDH2 |  |
|  | QRICH1 |  |
|  | QARS |  |
|  | USP19 |  |
|  | LAMB2 |  |
|  | CCDC71 |  |
|  | C3orf84 |  |
|  | CCDC36 |  |
|  | C3orf62 |  |
|  | USP4 |  |
|  | GPX1 |  |
|  | RHOA |  |
|  | AMT |  |
|  | NICN1 |  |
|  | DAG1 |  |
|  | BSN |  |
|  | APEH |  |
|  | MST1 |  |
|  | RNF123 |  |
|  | AMIGO3 |  |
|  | IP6K1 |  |
|  | CDHR4 |  |
|  | FAM212A |  |
|  | UBA7 |  |
|  | TRAIP |  |
|  | CAMKV |  |
|  | MON1A |  |
|  | RBM6 |  |
|  | RBM5 |  |
|  | SEMA3F |  |
|  | GNAT1 |  |
|  | SLC38A3 |  |
|  | GNAI2 |  |
|  | SEMA3B |  |
|  | IFRD2 |  |
|  | HYAL3 |  |
|  | HYAL1 |  |
|  | HYAL2 |  |
|  | TUSC2 |  |
|  | RASSF1 |  |
|  | ZMYND10 |  |
|  | NPRL2 |  |
|  | CACNA2D2 |  |

|  | C3orf18 |  |
| --- | --- | --- |
|  | HEMK1 |  |
|  | CISH |  |
|  | DOCK3 |  |
|  | RBM15B |  |
|  | DCAF1 |  |
|  | RAD54L2 |  |
|  | TEX264 |  |
|  | GRM2 |  |
|  | MOB4 |  |
|  | RFTN2 |  |
|  | MARS2 |  |
|  | BOLL |  |
|  | PLCL1 |  |
|  | C14orf119 |  |
|  | LMLN2 |  |
|  | CEBPE |  |
|  | SLC7A8 |  |
|  | RNF212B |  |
|  | HOMEZ |  |
|  | PPP1R3E |  |
|  | BCL2L2-PABPN1 |  |
|  | BCL2L2 |  |
|  | PABPN1 |  |
|  | SLC22A17 |  |
|  | EFS |  |
|  | CMTM5 |  |
|  | MYH6 |  |
|  | MYH7 |  |
|  | NGDN |  |
|  | ZFHX2 |  |
|  | THTPA |  |
|  | AP1G2 |  |
|  | JPH4 |  |
|  | DHRS2 |  |
|  | DHRS4 |  |
|  | CARMIL3 |  |
|  | CPNE6 |  |
|  | NRL |  |
|  | PCK2 |  |
|  | DCAF11 |  |
|  | FITM1 |  |
|  | PSME1 |  |
|  | PSME2 |  |
|  | RNF31 |  |
|  | IRF9 |  |
|  | REC8 |  |
|  | IPO4 |  |
|  | TM9SF1 |  |
|  | TSSK4 |  |
|  | GMPR2 |  |
|  | TINF2 |  |
|  | TGM1 |  |

|  | RABGGTA |  |
| --- | --- | --- |
|  | DHRS1 |  |
|  | NOP9 |  |
|  | CIDEB |  |
|  | LTB4R2 |  |
|  | LTB4R |  |
|  | ADCY4 |  |
|  | RIPK3 |  |
|  | NFATC4 |  |
|  | NYNRIN |  |
|  | KHNYN |  |
|  | SDR39U1 |  |
|  | CMA1 |  |
|  | GZMH |  |
|  | GZMB |  |
|  | STXBP6 |  |
|  | NOVA1 |  |
|  | FOXG1 |  |
|  | PRKD1 |  |
|  | G2E3 |  |
|  | COCH |  |
|  | AP4S1 |  |
|  | HECTD1 |  |
|  | HEATR5A |  |
|  | DTD2 |  |
|  | GPR33 |  |
|  | NUBPL |  |
|  | AKAP6 |  |
|  | NPAS3 |  |
|  | EGLN3 |  |
|  | SPTSSA |  |
|  | CFL2 |  |
|  | BAZ1A |  |
|  | FAM177A1 |  |
|  | KIAA0391 |  |
|  | PSMA6 |  |
|  | RALGAPA1 |  |
|  | NKX2-1 |  |
|  | NKX2-8 |  |
|  | PAX9 |  |
|  | SLC25A21 |  |
|  | MIPOL1 |  |
|  | FOXA1 |  |
|  | TTC6 |  |
|  | SSTR1 |  |
|  | CLEC14A |  |
|  | SEC23A |  |
|  | MIA2 |  |
|  | FBXO33 |  |
|  | LRFN5 |  |
|  | C14orf28 |  |
|  | KLHL28 |  |
|  | TOGARAM1 |  |

|  | FANCM |  |
| --- | --- | --- |
|  | MDGA2 |  |
|  | RPS29 |  |
|  | DNAAF2 |  |
|  | KLHDC1 |  |
|  | KLHDC2 |  |
|  | NEMF |  |
|  | ARF6 |  |
|  | L2HGDH |  |
|  | ATP5S |  |
|  | CDKL1 |  |
|  | SAV1 |  |
|  | NIN |  |
|  | PYGL |  |
|  | TRIM9 |  |
|  | TMX1 |  |
|  | FRMD6 |  |
|  | GNG2 |  |
|  | NID2 |  |
|  | PTGDR |  |
|  | PTGER2 |  |
|  | TXNDC16 |  |
|  | GPR137C |  |
|  | ERO1A |  |
|  | STYX |  |
|  | GNPNAT1 |  |
|  | FERMT2 |  |
|  | DDHD1 |  |
|  | CDKN3 |  |
|  | CNIH1 |  |
|  | SAMD4A |  |
|  | GCH1 |  |
|  | WDHD1 |  |
|  | MAPK1IP1L |  |
|  | LGALS3 |  |
|  | DLGAP5 |  |
|  | FBXO34 |  |
|  | TBPL2 |  |
|  | KTN1 |  |
|  | PELI2 |  |
|  | TMEM260 |  |
|  | OTX2 |  |
|  | AP5M1 |  |
|  | CCDC198 |  |
|  | SLC35F4 |  |
|  | ACTR10 |  |
|  | ARID4A |  |
|  | KIAA0586 |  |
|  | DACT1 |  |
|  | DAAM1 |  |
|  | GPR135 |  |
|  | JKAMP |  |
|  | CCDC175 |  |

|  | RTN1 |  |
| --- | --- | --- |
|  | LRRC9 |  |
|  | TMIGD3 |  |
|  | ADORA3 |  |
|  | RAP1A |  |
|  | FAM212B |  |
|  | DDX20 |  |
|  | KCND3 |  |
|  | CTTNBP2NL |  |
|  | WNT2B |  |
|  | ST7L |  |
|  | CAPZA1 |  |
|  | MOV10 |  |
|  | RHOC |  |
|  | PPM1J |  |
|  | FAM19A3 |  |
|  | LRIG2 |  |
|  | MAGI3 |  |
|  | RSBN1 |  |
|  | PTPN22 |  |
|  | BCL2L15 |  |
|  | HIPK1 |  |
|  | OLFML3 |  |
|  | SYT6 |  |
|  | TRIM33 |  |
|  | DENND2C |  |
|  | AMPD1 |  |
|  | NRAS |  |
|  | SIKE1 |  |
|  | SYCP1 |  |
|  | NGF |  |
|  | VANGL1 |  |
|  | CASQ2 |  |
|  | NHLH2 |  |
|  | SLC22A15 |  |
|  | ATP1A1 |  |
|  | IGSF3 |  |
|  | CD2 |  |
|  | PTGFRN |  |
|  | CD101 |  |
|  | TTF2 |  |
|  | TRIM45 |  |
|  | VTCN1 |  |
|  | MAN1A2 |  |
|  | FAM46C |  |
|  | GDAP2 |  |
|  | WDR3 |  |
|  | SPAG17 |  |
|  | TBX15 |  |
|  | HAO2 |  |
|  | ZNF697 |  |
|  | PHGDH |  |
|  | HMGCS2 |  |

|  | REG4 |  |
| --- | --- | --- |
|  | ADAM30 |  |
|  | NOTCH2 |  |
|  | SEC22B |  |
|  | PPIAL4A |  |
|  | FAM72B |  |
|  | HIST2H3PS2 |  |
|  | FAM72C |  |
|  | PPIAL4E |  |
|  | NBPF15 |  |
|  | PPIAL4F |  |
|  | SRGAP2B |  |
|  | FAM72D |  |
|  | PPIAL4D |  |
|  | NBPF20 |  |
|  | PDZK1 |  |
|  | RNF115 |  |
|  | POLR3C |  |
|  | NUDT17 |  |
|  | PIAS3 |  |
|  | ANKRD35 |  |
|  | ITGA10 |  |
|  | PEX11B |  |
|  | LIX1L |  |
|  | TXNIP |  |
|  | NBPF10 |  |
|  | NOTCH2NL |  |
|  | PRKAB2 |  |
|  | FMO5 |  |
|  | CHD1L |  |
|  | BCL9 |  |
|  | ACP6 |  |
|  | GJA5 |  |
|  | GJA8 |  |
|  | NBPF11 |  |
|  | PPIAL4G |  |
|  | NBPF14 |  |
|  | NUDT4B |  |
|  | PDE4DIP |  |
|  | NBPF9 |  |
|  | NBPF19 |  |
|  | PPIAL4C |  |
|  | FCGR1A |  |
|  | HIST2H3D |  |
|  | HIST2H2AA3 |  |
|  | HIST2H2AA4 |  |
|  | HIST2H2BE |  |
|  | HIST2H2AC |  |
|  | HIST2H2AB |  |
|  | SV2A |  |
|  | SF3B4 |  |
|  | MTMR11 |  |
|  | OTUD7B |  |

|  | VPS45 |  |
| --- | --- | --- |
|  | PLEKHO1 |  |
|  | ANP32E |  |
|  | APH1A |  |
|  | CIART |  |
|  | MRPS21 |  |
|  | PRPF3 |  |
|  | RPRD2 |  |
|  | TARS2 |  |
|  | ECM1 |  |
|  | ADAMTSL4 |  |
|  | MCL1 |  |
|  | ENSA |  |
|  | GOLPH3L |  |
|  | HORMAD1 |  |
|  | CTSS |  |
|  | CTSK |  |
|  | ARNT |  |
|  | SETDB1 |  |
|  | CERS2 |  |
|  | ANXA9 |  |
|  | MINDY1 |  |
|  | PRUNE1 |  |
|  | BNIPL |  |
|  | GABPB2 |  |
|  | SEMA6C |  |
|  | TNFAIP8L2-SCNM1 |  |
|  | LYSMD1 |  |
|  | SCNM1 |  |
|  | VPS72 |  |
|  | PIP5K1A |  |
|  | PSMD4 |  |
|  | ZNF687 |  |
|  | PI4KB |  |
|  | RFX5 |  |
|  | SELENBP1 |  |
|  | PSMB4 |  |
|  | POGZ |  |
|  | CGN |  |
|  | TUFT1 |  |
|  | SNX27 |  |
|  | CELF3 |  |
|  | RIIAD1 |  |
|  | MRPL9 |  |
|  | OAZ3 |  |
|  | TDRKH |  |
|  | LINGO4 |  |
|  | RORC |  |
|  | THEM4 |  |
|  | S100A10 |  |
|  | S100A11 |  |
|  | TCHHL1 |  |
|  | TCHH |  |

|  | RPTN |  |
| --- | --- | --- |
|  | FLG |  |
|  | FLG2 |  |
|  | CRNN |  |
|  | LCE5A |  |
|  | LCE3E |  |
|  | LCE3A |  |
|  | LCE2C |  |
|  | LCE2A |  |
|  | LCE4A |  |
|  | C1orf68 |  |
|  | KPRP |  |
|  | LCE1C |  |
|  | LCE1A |  |
|  | NSUN7 |  |
|  | APBB2 |  |
|  | UCHL1 |  |
|  | LIMCH1 |  |
|  | TMEM33 |  |
|  | DCAF4L1 |  |
|  | SLC30A9 |  |
|  | BEND4 |  |
|  | SHISA3 |  |
|  | ATP8A1 |  |
|  | KCTD8 |  |
|  | YIPF7 |  |
|  | GUF1 |  |
|  | GNPDA2 |  |
|  | GABRG1 |  |
|  | GABRA2 |  |
|  | GABRB1 |  |
|  | COMMD8 |  |
|  | ATP10D |  |
|  | CORIN |  |
|  | NFXL1 |  |
|  | CNGA1 |  |
|  | NIPAL1 |  |
|  | TXK |  |
|  | TEC |  |
|  | SLAIN2 |  |
|  | SLC10A4 |  |
|  | ZAR1 |  |
|  | FRYL |  |
|  | OCIAD1 |  |
|  | OCIAD2 |  |
|  | CWH43 |  |
|  | LRRC66 |  |
|  | SGCB |  |
|  | SPATA18 |  |
|  | USP46 |  |
|  | ERVMER34-1 |  |
|  | FIP1L1 |  |
|  | LNX1 |  |

|  | CHIC2 |  |
| --- | --- | --- |
|  | GSX2 |  |
|  | PDGFRA |  |
|  | KIT |  |
|  | KDR |  |
|  | SRD5A3 |  |
|  | TMEM165 |  |
|  | CLOCK |  |
|  | EXOC1 |  |
|  | CEP135 |  |
|  | KIAA1211 |  |
|  | AASDH |  |
|  | PPAT |  |
|  | PAICS |  |
|  | SRP72 |  |
|  | THEGL |  |
|  | HOPX |  |
|  | SPINK2 |  |
|  | ADGRL3 |  |
|  | TECRL |  |
|  | EPHA5 |  |
|  | CENPC |  |
|  | STAP1 |  |
|  | GNRHR |  |
|  | TMPRSS11D |  |
|  | TMPRSS11F |  |
|  | TMPRSS11B |  |
|  | YTHDC1 |  |
|  | UGT2A3 |  |
|  | UGT2B7 |  |
|  | UGT2B11 |  |
|  | UGT2B4 |  |
|  | UGT2A1 |  |
|  | UGT2A2 |  |
|  | SULT1E1 |  |
|  | CSN1S1 |  |
|  | CSN2 |  |
|  | PRR27 |  |
|  | ODAM |  |
|  | CABS1 |  |
|  | SMR3A |  |
|  | SMR3B |  |
|  | OPRPN |  |
|  | MUC7 |  |
|  | AMBN |  |
|  | ENAM |  |
|  | RUFY3 |  |
|  | GRSF1 |  |
|  | MOB1B |  |
|  | DCK |  |
|  | SLC4A4 |  |
|  | GC |  |
|  | NPFFR2 |  |

|  | COX18 |  |
| --- | --- | --- |
|  | ANKRD17 |  |
|  | ALB |  |
|  | AFM |  |
|  | RASSF6 |  |
|  | CXCL6 |  |
|  | PF4V1 |  |
|  | PPBP |  |
|  | CXCL5 |  |
|  | CXCL2 |  |
|  | MTHFD2L |  |
|  | EPGN |  |
|  | EREG |  |
|  | PARM1 |  |
|  | THAP6 |  |
|  | ODAPH |  |
|  | CDKL2 |  |
|  | G3BP2 |  |
|  | USO1 |  |
|  | PPEF2 |  |
|  | NAAA |  |
|  | SDAD1 |  |
|  | CXCL10 |  |
|  | ART3 |  |
|  | SCARB2 |  |
|  | FAM47E |  |
|  | FAM47E-STBD1 |  |
|  | CCDC158 |  |
|  | SHROOM3 |  |
|  | 11-Sep |  |
|  | CCNI |  |
|  | CNOT6L |  |
|  | MRPL1 |  |
|  | FRAS1 |  |
|  | ANXA3 |  |
|  | BMP2K |  |
|  | NAA11 |  |
|  | ANTXR2 |  |
|  | PRDM8 |  |
|  | FGF5 |  |
|  | PRKG2 |  |
|  | RASGEF1B |  |
|  | HNRNPD |  |
|  | TMEM150C |  |
|  | SCD5 |  |
|  | SEC31A |  |
|  | THAP9 |  |
|  | LIN54 |  |
|  | COPS4 |  |
|  | COQ2 |  |
|  | CYP11B1 |  |
|  | CYP11B2 |  |
|  | LY6E |  |

|  | LY6H |  |
| --- | --- | --- |
|  | GPIHBP1 |  |
|  | ZFP41 |  |
|  | GLI4 |  |
|  | RHPN1 |  |
|  | MAFA |  |
|  | ZC3H3 |  |
|  | GSDMD |  |
|  | MROH6 |  |
|  | NAPRT |  |
|  | EEF1D |  |
|  | TIGD5 |  |
|  | PYCR3 |  |
|  | TSTA3 |  |
|  | ZNF623 |  |
|  | ZNF707 |  |
|  | MAPK15 |  |
|  | FAM83H |  |
|  | SCRIB |  |
|  | NRBP2 |  |
|  | EPPK1 |  |
|  | PLEC |  |
|  | PARP10 |  |
|  | GRINA |  |
|  | SPATC1 |  |
|  | OPLAH |  |
|  | EXOSC4 |  |
|  | GPAA1 |  |
|  | CYC1 |  |
|  | SHARPIN |  |
|  | WDR97 |  |
|  | HGH1 |  |
|  | MROH1 |  |
|  | BOP1 |  |
|  | HSF1 |  |
|  | DGAT1 |  |
|  | SCRT1 |  |
|  | TMEM249 |  |
|  | FBXL6 |  |
|  | SLC52A2 |  |
|  | CPSF1 |  |
|  | SLC39A4 |  |
|  | VPS28 |  |
|  | TONSL |  |
|  | CYHR1 |  |
|  | KIFC2 |  |
|  | FOXH1 |  |
|  | PPP1R16A |  |
|  | GPT |  |
|  | MFSD3 |  |
|  | RECQL4 |  |
|  | LRRC14 |  |
|  | LRRC24 |  |

|  | C8orf82 |  |
| --- | --- | --- |
|  | ARHGAP39 |  |
|  | ZNF251 |  |
|  | ZNF34 |  |
|  | ZNF7 |  |
|  | COMMD5 |  |
|  | ZNF250 |  |
|  | ZNF16 |  |
|  | WASHC1 |  |
|  | FOXD4 |  |
|  | DOCK8 |  |
|  | KANK1 |  |
|  | DMRT1 |  |
|  | DMRT3 |  |
|  | DMRT2 |  |
|  | SMARCA2 |  |
|  | VLDLR |  |
|  | PUM3 |  |
|  | RFX3 |  |
|  | GLIS3 |  |
|  | SLC1A1 |  |
|  | SPATA6L |  |
|  | PLPP6 |  |
|  | AK3 |  |
|  | RCL1 |  |
|  | INSL6 |  |
|  | RLN1 |  |
|  | PLGRKT |  |
|  | CD274 |  |
|  | RIC1 |  |
|  | ERMP1 |  |
|  | MLANA |  |
|  | KIAA2026 |  |
|  | RANBP6 |  |
|  | IL33 |  |
|  | TPD52L3 |  |
|  | UHRF2 |  |
|  | GLDC |  |
|  | KDM4C |  |
|  | DMAC1 |  |
|  | PTPRD |  |
|  | TYRP1 |  |
|  | MPDZ |  |
|  | NFIB |  |
|  | ZDHHC21 |  |
|  | CER1 |  |
|  | FREM1 |  |
|  | TTC39B |  |
|  | SNAPC3 |  |
|  | PSIP1 |  |
|  | CCDC171 |  |
|  | C9orf92 |  |
|  | BNC2 |  |

|  | CNTLN |  |
| --- | --- | --- |
|  | SH3GL2 |  |
|  | ADAMTSL1 |  |
|  | SAXO1 |  |
|  | RRAGA |  |
|  | HAUS6 |  |
|  | PLIN2 |  |
|  | DENND4C |  |
|  | SLC24A2 |  |
|  | MLLT3 |  |
|  | FOCAD |  |
|  | HACD4 |  |
|  | IFNA10 |  |
|  | IFNA17 |  |
|  | IFNA14 |  |
|  | IFNA5 |  |
|  | KLHL9 |  |
|  | IFNA13 |  |
|  | IFNA2 |  |
|  | IFNA1 |  |
|  | MTAP |  |
|  | CDKN2A |  |
|  | DMRTA1 |  |
|  | ELAVL2 |  |
|  | TUSC1 |  |
|  | CAAP1 |  |
|  | PLAA |  |
|  | IFT74 |  |
|  | LRRC19 |  |
|  | TEK |  |
|  | MOB3B |  |
|  | IFNK |  |
|  | C9orf72 |  |
|  | LINGO2 |  |
|  | ACO1 |  |
|  | DDX58 |  |
|  | TOPORS |  |
|  | SMIM27 |  |
|  | TAF1L |  |
|  | TMEM215 |  |
|  | APTX |  |
|  | SMU1 |  |
|  | B4GALT1 |  |
|  | BAG1 |  |
|  | CHMP5 |  |
|  | AQP7 |  |
|  | AQP3 |  |
|  | NOL6 |  |
|  | PRSS3 |  |
|  | UBE2R2 |  |
|  | UBAP2 |  |
|  | DCAF12 |  |
|  | UBAP1 |  |

|  | KIF24 |  |
| --- | --- | --- |
|  | NUDT2 |  |
|  | MYORG |  |
|  | C9orf24 |  |
|  | FAM219A |  |
|  | CNTFR |  |
|  | RPP25L |  |
|  | DCTN3 |  |
|  | ARID3C |  |
|  | GALT |  |
|  | ASPH |  |
|  | GGH |  |
|  | TTPA |  |
|  | YTHDF3 |  |
|  | BHLHE22 |  |
|  | CYP7B1 |  |
|  | MTFR1 |  |
|  | PDE7A |  |
|  | DNAJC5B |  |
|  | TRIM55 |  |
|  | RRS1 |  |
|  | ADHFE1 |  |
|  | C8orf46 |  |
|  | MYBL1 |  |
|  | VCPIP1 |  |
|  | C8orf44-SGK3 |  |
|  | SGK3 |  |
|  | MCMDC2 |  |
|  | TCF24 |  |
|  | CSPP1 |  |
|  | ARFGEF1 |  |
|  | PREX2 |  |
|  | C8orf34 |  |
|  | SULF1 |  |
|  | FAM96A |  |
|  | SNX1 |  |
|  | SNX22 |  |
|  | PPIB |  |
|  | CSNK1G1 |  |
|  | PCLAF |  |
|  | TRIP4 |  |
|  | ZNF609 |  |
|  | OAZ2 |  |
|  | RBPMS2 |  |
|  | PLEKHO2 |  |
|  | ANKDD1A |  |
|  | MTFMT |  |
|  | RASL12 |  |
|  | UBAP1L |  |
|  | PDCD7 |  |
|  | CILP |  |
|  | IGDCC3 |  |
|  | IGDCC4 |  |

|  | DPP8 |  |
| --- | --- | --- |
|  | HACD3 |  |
|  | INTS14 |  |
|  | SLC24A1 |  |
|  | DENND4A |  |
|  | MEGF11 |  |
|  | DIS3L |  |
|  | TIPIN |  |
|  | MAP2K1 |  |
|  | SNAPC5 |  |
|  | ZWILCH |  |
|  | LCTL |  |
|  | AAGAB |  |
|  | IQCH |  |
|  | MAP2K5 |  |
|  | PIAS1 |  |
|  | CALML4 |  |
|  | ITGA11 |  |
|  | CORO2B |  |
|  | GLCE |  |
|  | PAQR5 |  |
|  | KIF23 |  |
|  | RPLP1 |  |
|  | TLE3 |  |
|  | LRRC49 |  |
|  | THAP10 |  |
|  | CT62 |  |
|  | THSD4 |  |
|  | NR2E3 |  |
|  | MYO9A |  |
|  | SENP8 |  |
|  | PKM |  |
|  | PARP6 |  |
|  | CELF6 |  |
|  | HEXA |  |
|  | ARIH1 |  |
|  | GOLGA6B |  |
|  | BBS4 |  |
|  | ADPGK |  |
|  | NEO1 |  |
|  | HCN4 |  |
|  | NPTN |  |
|  | CD276 |  |
|  | C15orf59 |  |
|  | TBC1D21 |  |
|  | LOXL1 |  |
|  | STOML1 |  |
|  | PML |  |
|  | GOLGA6A |  |
|  | ISLR2 |  |
|  | ISLR |  |
|  | STRA6 |  |
|  | CCDC33 |  |

|  | CYP11A1 |  |
| --- | --- | --- |
|  | SEMA7A |  |
|  | UBL7 |  |
|  | ARID3B |  |
|  | EDC3 |  |
|  | CYP1A1 |  |
|  | CYP1A2 |  |
|  | CSK |  |
|  | LMAN1L |  |
|  | CPLX3 |  |
|  | ULK3 |  |
|  | SCAMP2 |  |
|  | MPI |  |
|  | FAM219B |  |
|  | COX5A |  |
|  | RPP25 |  |
|  | SCAMP5 |  |
|  | PPCDC |  |
|  | C15orf39 |  |
|  | GOLGA6C |  |
|  | GOLGA6D |  |
|  | COMMD4 |  |
|  | NEIL1 |  |
|  | MAN2C1 |  |
|  | SIN3A |  |
|  | SNUPN |  |
|  | IMP3 |  |
|  | CSPG4 |  |
|  | ODF3L1 |  |
|  | UBE2Q2 |  |
|  | NRG4 |  |
|  | TMEM266 |  |
|  | ETFA |  |
|  | ISL2 |  |
|  | SCAPER |  |
|  | RCN2 |  |
|  | PSTPIP1 |  |
|  | TSPAN3 |  |
|  | PEAK1 |  |
|  | HMG20A |  |
|  | LINGO1 |  |
|  | TBC1D2B |  |
|  | SH2D7 |  |
|  | CIB2 |  |
|  | IDH3A |  |
|  | ACSBG1 |  |
|  | DNAJA4 |  |
|  | WDR61 |  |
|  | IREB2 |  |
|  | HYKK |  |
|  | PSMA4 |  |
|  | CHRNA5 |  |
|  | CHRNA3 |  |

|  | CHRNB4 |  |
| --- | --- | --- |
|  | ADAMTS7 |  |
|  | MORF4L1 |  |
|  | CTSH |  |
|  | RASGRF1 |  |
|  | ANKRD34C |  |
|  | TMED3 |  |
|  | KIAA1024 |  |
|  | BCL2A1 |  |
|  | ZFAND6 |  |
|  | FAH |  |
|  | CTXND1 |  |
|  | ARNT2 |  |
|  | ABHD17C |  |
|  | CEMIP |  |
|  | MESD |  |
|  | CFAP161 |  |
|  | IL16 |  |
|  | STARD5 |  |
|  | TMC3 |  |
|  | MEX3B |  |
|  | SAXO2 |  |
|  | GOLGA6L10 |  |
|  | GOLGA6L9 |  |
|  | CPEB1 |  |
|  | AP3B2 |  |
|  | FSD2 |  |
|  | WHAMM |  |
|  | HOMER2 |  |
|  | FAM103A1 |  |
|  | C15orf40 |  |
|  | BTBD1 |  |
|  | TM6SF1 |  |
|  | BNC1 |  |
|  | SH3GL3 |  |
|  | ADAMTSL3 |  |
|  | UNC93B1 |  |
|  | ALDH3B1 |  |
|  | NDUFS8 |  |
|  | TCIRG1 |  |
|  | KMT5B |  |
|  | C11orf24 |  |
|  | LRP5 |  |
|  | PPP6R3 |  |
|  | GAL |  |
|  | TESMIN |  |
|  | CPT1A |  |
|  | IGHMBP2 |  |
|  | MRGPRD |  |
|  | MRGPRF |  |
|  | TPCN2 |  |
|  | MYEOV |  |
|  | CAMSAP3 |  |

|  | XAB2 |  |
| --- | --- | --- |
|  | PCP2 |  |
|  | RETN |  |
|  | MCEMP1 |  |
|  | FCER2 |  |
|  | CLEC4G |  |
|  | CD209 |  |
|  | CLEC4M |  |
|  | EVI5L |  |
|  | PRR36 |  |
|  | LRRC8E |  |
|  | MAP2K7 |  |
|  | TGFBR3L |  |
|  | SNAPC2 |  |
|  | CTXN1 |  |
|  | TIMM44 |  |
|  | ELAVL1 |  |
|  | CCL25 |  |
|  | FBN3 |  |
|  | CERS4 |  |
|  | CD320 |  |
|  | NDUFA7 |  |
|  | RPS28 |  |
|  | KANK3 |  |
|  | ANGPTL4 |  |
|  | RAB11B |  |
|  | 2-Mar |  |
|  | HNRNPM |  |
|  | PRAM1 |  |
|  | ZNF414 |  |
|  | MYO1F |  |
|  | ADAMTS10 |  |
|  | ACTL9 |  |
|  | OR2Z1 |  |
|  | ZNF558 |  |
|  | MBD3L1 |  |
|  | MUC16 |  |
|  | OR1M1 |  |
|  | OR7G2 |  |
|  | OR7G1 |  |
|  | OR7G3 |  |
|  | ZNF317 |  |
|  | OR7D2 |  |
|  | OR7D4 |  |
|  | OR7E24 |  |
|  | ZNF559 |  |
|  | ZNF559-ZNF177 |  |
|  | ZNF177 |  |
|  | ZNF266 |  |
|  | ZNF560 |  |
|  | ZNF426 |  |
|  | ZNF121 |  |
|  | ZNF561 |  |

|  | ZNF562 |  |
| --- | --- | --- |
|  | ZNF846 |  |
|  | FBXL12 |  |
|  | UBL5 |  |
|  | OLFM2 |  |
|  | COL5A3 |  |
|  | RDH8 |  |
|  | C19orf66 |  |
|  | ANGPTL6 |  |
|  | PPAN |  |
|  | EIF3G |  |
|  | DNMT1 |  |
|  | S1PR2 |  |
|  | MRPL4 |  |
|  | ICAM1 |  |
|  | ICAM4 |  |
|  | ICAM5 |  |
|  | ZGLP1 |  |
|  | RAVER1 |  |
|  | ICAM3 |  |
|  | TYK2 |  |
|  | CDC37 |  |
|  | PDE4A |  |
|  | KEAP1 |  |
|  | S1PR5 |  |
|  | ATG4D |  |
|  | KRI1 |  |
|  | CDKN2D |  |
|  | AP1M2 |  |
|  | SLC44A2 |  |
|  | QTRT1 |  |
|  | DNM2 |  |
|  | TMED1 |  |
|  | C19orf38 |  |
|  | CARM1 |  |
|  | YIPF2 |  |
|  | SMARCA4 |  |
|  | LDLR |  |
|  | SPC24 |  |
|  | KANK2 |  |
|  | DOCK6 |  |
|  | ANGPTL8 |  |
|  | TSPAN16 |  |
|  | RAB3D |  |
|  | TMEM205 |  |
|  | CCDC159 |  |
|  | PLPPR2 |  |
|  | SWSAP1 |  |
|  | EPOR |  |
|  | RGL3 |  |
|  | CCDC151 |  |
|  | PRKCSH |  |
|  | ELAVL3 |  |

|  | ZNF653 |  |
| --- | --- | --- |
|  | ECSIT |  |
|  | CNN1 |  |
|  | ELOF1 |  |
|  | ACP5 |  |
|  | ZNF627 |  |
|  | ZNF823 |  |
|  | ZNF441 |  |
|  | ZNF440 |  |
|  | ZNF439 |  |
|  | ZNF69 |  |
|  | ZNF763 |  |
|  | ZNF433 |  |
|  | ZNF844 |  |
|  | ZNF625 |  |
|  | ZNF136 |  |
|  | ZNF44 |  |
|  | ZNF563 |  |
|  | ZNF442 |  |
|  | ZNF799 |  |
|  | ZNF443 |  |
|  | ZNF709 |  |
|  | ZNF564 |  |
|  | ZNF490 |  |
|  | ZNF791 |  |
|  | MAN2B1 |  |
|  | WDR83 |  |
|  | WDR83OS |  |
|  | DHPS |  |
|  | GNG14 |  |
|  | TNPO2 |  |
|  | BEST2 |  |
|  | HOOK2 |  |
|  | PRDX2 |  |
|  | RTBDN |  |
|  | MAST1 |  |
|  | DNASE2 |  |
|  | KLF1 |  |
|  | GCDH |  |
|  | SYCE2 |  |
|  | RAD23A |  |
|  | DAND5 |  |
|  | NFIX |  |
|  | LYL1 |  |
|  | TRMT1 |  |
|  | NACC1 |  |
|  | IER2 |  |
|  | CACNA1A |  |
|  | CCDC130 |  |
|  | MRI1 |  |
|  | C19orf53 |  |
|  | ZSWIM4 |  |
|  | NANOS3 |  |

|  | C19orf57 |  |
| --- | --- | --- |
|  | CC2D1A |  |
|  | PODNL1 |  |
|  | DCAF15 |  |
|  | RFX1 |  |
|  | RLN3 |  |
|  | IL27RA |  |
|  | PALM3 |  |
|  | MISP3 |  |
|  | C19orf67 |  |
|  | PRKACA |  |
|  | ASF1B |  |
|  | ADGRL1 |  |
|  | ADGRE5 |  |
|  | DDX39A |  |
|  | PKN1 |  |
|  | GIPC1 |  |
|  | DNAJB1 |  |
|  | TECR |  |
|  | NDUFB7 |  |
|  | CLEC17A |  |
|  | ADGRE3 |  |
|  | ZNF333 |  |
|  | ADGRE2 |  |
|  | OR7A17 |  |
|  | OR7C2 |  |
|  | SLC1A6 |  |
|  | CCDC105 |  |
|  | CASP14 |  |
|  | OR1I1 |  |
|  | SYDE1 |  |
|  | ILVBL |  |
|  | NOTCH3 |  |
|  | EPHX3 |  |
|  | BRD4 |  |
|  | AKAP8 |  |
|  | AKAP8L |  |
|  | WIZ |  |
|  | RASAL3 |  |
|  | PGLYRP2 |  |
|  | CYP4F22 |  |
|  | CYP4F8 |  |
|  | CYP4F3 |  |
|  | CYP4F12 |  |
|  | OR10H2 |  |
|  | OR10H3 |  |
|  | OR10H5 |  |
|  | OR10H1 |  |
|  | CYP4F2 |  |
|  | CYP4F11 |  |
|  | OR10H4 |  |
|  | TPM4 |  |
|  | RAB8A |  |

|  | HSH2D |  |
| --- | --- | --- |
|  | AP1M1 |  |
|  | EPS15L1 |  |
|  | CALR3 |  |
|  | C19orf44 |  |
|  | CHERP |  |
|  | SLC35E1 |  |
|  | MED26 |  |
|  | PITPNA |  |
|  | SLC43A2 |  |
|  | SCARF1 |  |
|  | RILP |  |
|  | PRPF8 |  |
|  | TLCD2 |  |
|  | WDR81 |  |
|  | SERPINF2 |  |
|  | SMYD4 |  |
|  | RPA1 |  |
|  | DPH1 |  |
|  | OVCA2 |  |
|  | SMG6 |  |
|  | SRR |  |
|  | TSR1 |  |
|  | SGSM2 |  |
|  | MNT |  |
|  | METTL16 |  |
|  | PAFAH1B1 |  |
|  | CLUH |  |
|  | RAP1GAP2 |  |
|  | OR1D5 |  |
|  | OR1D2 |  |
|  | OR3A2 |  |
|  | OR3A1 |  |
|  | OR3A3 |  |
|  | OR1E2 |  |
|  | SPATA22 |  |
|  | RPH3AL |  |
|  | C17orf97 |  |
|  | SPR |  |
|  | SFXN5 |  |
|  | RAB11FIP5 |  |
|  | NOTO |  |
|  | SMYD5 |  |
|  | PRADC1 |  |
|  | EGR4 |  |
|  | ALMS1 |  |
|  | TPRKB |  |
|  | C2orf78 |  |
|  | STAMBP |  |
|  | ACTG2 |  |
|  | TET3 |  |
|  | BOLA3 |  |
|  | MOB1A |  |

|  | MTHFD2 |  |
| --- | --- | --- |
|  | SLC4A5 |  |
|  | DCTN1 |  |
|  | C2orf81 |  |
|  | RTKN |  |
|  | INO80B |  |
|  | WBP1 |  |
|  | MOGS |  |
|  | MRPL53 |  |
|  | CCDC142 |  |
|  | TTC31 |  |
|  | LBX2 |  |
|  | DQX1 |  |
|  | AUP1 |  |
|  | HTRA2 |  |
|  | LOXL3 |  |
|  | DOK1 |  |
|  | M1AP |  |
|  | SEMA4F |  |
|  | HK2 |  |
|  | TACR1 |  |
|  | MRPL19 |  |
|  | LRRTM4 |  |
|  | REG3G |  |
|  | REG3A |  |
|  | CTNNA2 |  |
|  | LRRTM1 |  |
|  | SUCLG1 |  |
|  | DNAH6 |  |
|  | TRABD2A |  |
|  | TCF7L1 |  |
|  | TGOLN2 |  |
|  | RETSAT |  |
|  | ELMOD3 |  |
|  | CAPG |  |
|  | SH2D6 |  |
|  | MAT2A |  |
|  | GGCX |  |
|  | RNF181 |  |
|  | TMEM150A |  |
|  | C2orf68 |  |
|  | SFTPB |  |
|  | GNLY |  |
|  | ATOH8 |  |
|  | ST3GAL5 |  |
|  | POLR1A |  |
|  | PTCD3 |  |
|  | IMMT |  |
|  | MRPL35 |  |
|  | REEP1 |  |
|  | KDM3A |  |
|  | RNF103-CHMP3 |  |
|  | CHMP3 |  |

|  | RNF103 |  |
| --- | --- | --- |
|  | RMND5A |  |
|  | CD8A |  |
|  | CD8B |  |
|  | PLGLB1 |  |
|  | PLGLB2 |  |
|  | KRCC1 |  |
|  | SMYD1 |  |
|  | THNSL2 |  |
|  | FOXI3 |  |
|  | LOC100509620 |  |
|  | TEKT4 |  |
|  | MRPS5 |  |
|  | ZNF514 |  |
|  | ZNF2 |  |
|  | KCNIP3 |  |
|  | FAHD2A |  |
|  | ANKRD36C |  |
|  | GPAT2 |  |
|  | ADRA2B |  |
|  | ASTL |  |
|  | DUSP2 |  |
|  | STARD7 |  |
|  | TMEM127 |  |
|  | CIAO1 |  |
|  | SNRNP200 |  |
|  | ITPRIPL1 |  |
|  | NCAPH |  |
|  | NEURL3 |  |
|  | ARID5A |  |
|  | KANSL3 |  |
|  | FER1L5 |  |
|  | LMAN2L |  |
|  | CNNM4 |  |
|  | CNNM3 |  |
|  | ANKRD23 |  |
|  | ANKRD39 |  |
|  | SEMA4C |  |
|  | FAM178B |  |
|  | FAHD2B |  |
|  | ANKRD36 |  |
|  | ANKRD36B |  |
|  | ACTR1B |  |
|  | TMEM131 |  |
|  | VWA3B |  |
|  | CNGA3 |  |
|  | INPP4A |  |
|  | MGAT4A |  |
|  | KIAA1211L |  |
|  | TSGA10 |  |
|  | LIPT1 |  |
|  | MRPL30 |  |
|  | LYG2 |  |

|  | EIF5B |  |
| --- | --- | --- |
|  | REV1 |  |
|  | AFF3 |  |
|  | LONRF2 |  |
|  | CHST10 |  |
|  | NPAS2 |  |
|  | RPL31 |  |
|  | TBC1D8 |  |
|  | CNOT11 |  |
|  | RNF149 |  |
|  | CREG2 |  |
|  | RFX8 |  |
|  | MAP4K4 |  |
|  | IL1R2 |  |
|  | IL1R1 |  |
|  | IL1RL2 |  |
|  | IL1RL1 |  |
|  | IL18R1 |  |
|  | IL18RAP |  |
|  | SLC9A4 |  |
|  | MFSD9 |  |
|  | POU3F3 |  |
|  | GPR45 |  |
|  | TGFBRAP1 |  |
|  | C2orf49 |  |
|  | FHL2 |  |
|  | NCK2 |  |
|  | C2orf40 |  |
|  | UXS1 |  |
|  | CD8B2 |  |
|  | ST6GAL2 |  |
|  | RGPD4 |  |
|  | SLC5A7 |  |
|  | SULT1C2 |  |
|  | GCC2 |  |
|  | LIMS1 |  |
|  | RANBP2 |  |
|  | CCDC138 |  |
|  | EDAR |  |
|  | SH3RF3 |  |
|  | GABARAP |  |
|  | ELP5 |  |
|  | SLC2A4 |  |
|  | YBX2 |  |
|  | GNAS |  |
|  | NELFCD |  |
|  | CTSZ |  |
|  | TUBB1 |  |
|  | ZNF831 |  |
|  | PHACTR3 |  |
|  | SYCP2 |  |
|  | FAM217B |  |
|  | PPP1R3D |  |

|  | CDH26 |  |
| --- | --- | --- |
|  | CDH4 |  |
|  | LSM14B |  |
|  | SS18L1 |  |
|  | MTG2 |  |
|  | HRH3 |  |
|  | OSBPL2 |  |
|  | ADRM1 |  |
|  | LAMA5 |  |
|  | RPS21 |  |
|  | CABLES2 |  |
|  | SLCO4A1 |  |
|  | NTSR1 |  |
|  | TCFL5 |  |
|  | DIDO1 |  |
|  | GID8 |  |
|  | SLC17A9 |  |
|  | YTHDF1 |  |
|  | NKAIN4 |  |
|  | COL20A1 |  |
|  | KCNQ2 |  |
|  | PTK6 |  |
|  | SRMS |  |
|  | HELZ2 |  |
|  | GMEB2 |  |
|  | STMN3 |  |
|  | RTEL1 |  |
|  | ARFRP1 |  |
|  | LIME1 |  |
|  | SLC2A4RG |  |
|  | ZBTB46 |  |
|  | TPD52L2 |  |
|  | DNAJC5 |  |
|  | UCKL1 |  |
|  | ZNF512B |  |
|  | SAMD10 |  |
|  | PRPF6 |  |
|  | TCEA2 |  |
|  | RGS19 |  |
|  | OPRL1 |  |
|  | LKAAEAR1 |  |
|  | MYT1 |  |
|  | PCMTD2 |  |
|  | LOC102723996 |  |
|  | SIK1B |  |
|  | CBSL |  |
|  | U2AF1L5 |  |
|  | CRYAA2 |  |
|  | SMIM11B |  |
|  | KCNE1B |  |
|  | POTED |  |
|  | LIPI |  |
|  | HSPA13 |  |

|  | USP25 |  |
| --- | --- | --- |
|  | CXADR |  |
|  | BTG3 |  |
|  | C21orf91 |  |
|  | CHODL |  |
|  | NCAM2 |  |
|  | MRPL39 |  |
|  | GABPA |  |
|  | APP |  |
|  | CYYR1 |  |
|  | ADAMTS1 |  |
|  | ADAMTS5 |  |
|  | N6AMT1 |  |
|  | LTN1 |  |
|  | USP16 |  |
|  | CCT8 |  |
|  | BACH1 |  |
|  | GRIK1 |  |
|  | CLDN17 |  |
|  | CLDN8 |  |
|  | KRTAP13-2 |  |
|  | KRTAP13-1 |  |
|  | KRTAP13-3 |  |
|  | KRTAP13-4 |  |
|  | KRTAP8-1 |  |
|  | KRTAP11-1 |  |
|  | TIAM1 |  |
|  | SCAF4 |  |
|  | HUNK |  |
|  | MRAP |  |
|  | C21orf59-TCP10L |  |
|  | CFAP298 |  |
|  | SYNJ1 |  |
|  | C21orf62 |  |
|  | OLIG1 |  |
|  | IL10RB |  |
|  | IFNAR1 |  |
|  | IFNGR2 |  |
|  | TMEM50B |  |
|  | GART |  |
|  | SON |  |
|  | DONSON |  |
|  | CRYZL1 |  |
|  | ITSN1 |  |
|  | SLC5A3 |  |
|  | SMIM11A |  |
|  | C21orf140 |  |
|  | KCNE1 |  |
|  | RCAN1 |  |
|  | C2CD2 |  |
|  | ZBTB21 |  |
|  | UMODL1 |  |
|  | ABCG1 |  |

|  | TFF3 |  |
| --- | --- | --- |
|  | UBASH3A |  |
|  | RSPH1 |  |
|  | SLC37A1 |  |
|  | PDE9A |  |
|  | NDUFV3 |  |
|  | PKNOX1 |  |
|  | CBS |  |
|  | U2AF1 |  |
|  | SIK1 |  |
|  | HSF2BP |  |
|  | RRP1B |  |
|  | PDXK |  |
|  | CSTB |  |
|  | RRP1 |  |
|  | AGPAT3 |  |
|  | TRAPPC10 |  |
|  | PWP2 |  |
|  | C21orf33 |  |
|  | ICOSLG |  |
|  | DNMT3L |  |
|  | AIRE |  |
|  | PFKL |  |
|  | C21orf2 |  |
|  | TRPM2 |  |
|  | LRRC3 |  |
|  | TSPEAR |  |
|  | KRTAP10-1 |  |
|  | KRTAP10-2 |  |
|  | KRTAP10-3 |  |
|  | KRTAP10-4 |  |
|  | KRTAP10-5 |  |
|  | KRTAP10-6 |  |
|  | KRTAP10-7 |  |
|  | KRTAP10-8 |  |
|  | KRTAP10-9 |  |
|  | KRTAP10-10 |  |
|  | KRTAP12-3 |  |
|  | KRTAP12-2 |  |
|  | KRTAP12-1 |  |
|  | KRTAP10-12 |  |
|  | UBE2G2 |  |
|  | SUMO3 |  |
|  | PTTG1IP |  |
|  | ITGB2 |  |
|  | ADARB1 |  |
|  | POFUT2 |  |
|  | COL18A1 |  |
|  | SLC19A1 |  |
|  | PCBP3 |  |
|  | COL6A1 |  |
|  | FTCD |  |
|  | SPATC1L |  |

|  | LSS |  |
| --- | --- | --- |
|  | MCM3AP |  |
|  | C21orf58 |  |
|  | PCNT |  |
|  | DIP2A |  |
|  | S100B |  |
|  | OR11H1 |  |
|  | POTEH |  |
|  | CCT8L2 |  |
|  | GAB4 |  |
|  | TMEM121B |  |
|  | HDHD5 |  |
|  | ADA2 |  |
|  | CECR2 |  |
|  | SLC25A18 |  |
|  | ATP6V1E1 |  |
|  | BCL2L13 |  |
|  | BID |  |
|  | MICAL3 |  |
|  | PEX26 |  |
|  | TUBA8 |  |
|  | USP18 |  |
|  | TMEM191B |  |
|  | RIMBP3 |  |
|  | DGCR6 |  |
|  | PRODH |  |
|  | DGCR2 |  |
|  | ESS2 |  |
|  | TSSK2 |  |
|  | SLC25A1 |  |
|  | CLTCL1 |  |
|  | HIRA |  |
|  | C22orf39 |  |
|  | UFD1 |  |
|  | CDC45 |  |
|  | CLDN5 |  |
|  | 5-Sep |  |
|  | GP1BB |  |
|  | TBX1 |  |
|  | GNB1L |  |
|  | RTL10 |  |
|  | TXNRD2 |  |
|  | COMT |  |
|  | ARVCF |  |
|  | TANGO2 |  |
|  | DGCR8 |  |
|  | TRMT2A |  |
|  | RANBP1 |  |
|  | ZDHHC8 |  |
|  | DGCR6L |  |
|  | USP41 |  |
|  | ZNF74 |  |
|  | KLHL22 |  |

|  | MED15 |  |
| --- | --- | --- |
|  | SERPIND1 |  |
|  | SNAP29 |  |
|  | CRKL |  |
|  | AIFM3 |  |
|  | LZTR1 |  |
|  | THAP7 |  |
|  | P2RX6 |  |
|  | SLC7A4 |  |
|  | LRRC74B |  |
|  | GGT2 |  |
|  | RIMBP3B |  |
|  | HIC2 |  |
|  | TMEM191C |  |
|  | RIMBP3C |  |
|  | UBE2L3 |  |
|  | YDJC |  |
|  | CCDC116 |  |
|  | PPIL2 |  |
|  | YPEL1 |  |
|  | MAPK1 |  |
|  | PPM1F |  |
|  | TOP3B |  |
|  | VPREB1 |  |
|  | ZNF280B |  |
|  | PRAME |  |
|  | GGTLC2 |  |
|  | IGLL5 |  |
|  | RSPH14 |  |
|  | GNAZ |  |
|  | BCR |  |
|  | IGLL1 |  |
|  | RGL4 |  |
|  | ZNF70 |  |
|  | VPREB3 |  |
|  | C22orf15 |  |
|  | CHCHD10 |  |
|  | MMP11 |  |
|  | SMARCB1 |  |
|  | SLC2A11 |  |
|  | MIF |  |
|  | DDTL |  |
|  | DDT |  |
|  | GSTT4 |  |
|  | CABIN1 |  |
|  | SUSD2 |  |
|  | GGT5 |  |
|  | SPECC1L |  |
|  | GUCD1 |  |
|  | SNRPD3 |  |
|  | GGT1 |  |
|  | LRRC75B |  |
|  | PIWIL3 |  |

|  | SGSM1 |  |
| --- | --- | --- |
|  | TMEM211 |  |
|  | KIAA1671 |  |
|  | CRYBB3 |  |
|  | CRYBB2 |  |
|  | LRP5L |  |
|  | GRK3 |  |
|  | MYO18B |  |
|  | HPS4 |  |
|  | TFIP11 |  |
|  | TPST2 |  |
|  | MN1 |  |
|  | PITPNB |  |
|  | TTC28 |  |
|  | CHEK2 |  |
|  | HSCB |  |
|  | CCDC117 |  |
|  | XBP1 |  |
|  | MAOA |  |
|  | NDP |  |
|  | EFHC2 |  |
|  | FUNDC1 |  |
|  | DUSP21 |  |
|  | KDM6A |  |
|  | CXorf36 |  |
|  | KRBOX4 |  |
|  | ZNF674 |  |
|  | CHST7 |  |
|  | SLC9A7 |  |
|  | JADE3 |  |
|  | NDUFB11 |  |
|  | RBM10 |  |
|  | UBA1 |  |
|  | CDK16 |  |
|  | ZNF41 |  |
|  | ARAF |  |
|  | TIMP1 |  |
|  | CFP |  |
|  | ELK1 |  |
|  | UXT |  |
|  | ZNF81 |  |
|  | ZNF182 |  |
|  | ZNF630 |  |
|  | SSX5 |  |
|  | SSX1 |  |
|  | SSX3 |  |
|  | SLC38A5 |  |
|  | FTSJ1 |  |
|  | PORCN |  |
|  | TBC1D25 |  |
|  | RBM3 |  |
|  | WAS |  |
|  | SUV39H1 |  |

|  | GLOD5 |  |
| --- | --- | --- |
|  | GATA1 |  |
|  | HDAC6 |  |
|  | ERAS |  |
|  | PCSK1N |  |
|  | SLC35A2 |  |
|  | PIM2 |  |
|  | OTUD5 |  |
|  | KCND1 |  |
|  | GRIPAP1 |  |
|  | CCDC120 |  |
|  | PRAF2 |  |
|  | WDR45 |  |
|  | GPKOW |  |
|  | MAGIX |  |
|  | PRICKLE3 |  |
|  | SYP |  |
|  | CACNA1F |  |
|  | CCDC22 |  |
|  | FOXP3 |  |
|  | PPP1R3F |  |
|  | GAGE12J |  |
|  | GAGE1 |  |
|  | USP27X |  |
|  | CLCN5 |  |
|  | AKAP4 |  |
|  | CCNB3 |  |
|  | DGKK |  |
|  | SHROOM4 |  |
|  | NUDT10 |  |
|  | CXorf67 |  |
|  | NUDT11 |  |
|  | CENPVL2 |  |
|  | CENPVL1 |  |
|  | GSPT2 |  |
|  | MAGED1 |  |
|  | MAGED4B |  |
|  | MAGED4 |  |
|  | SSX7 |  |
|  | SSX2 |  |
|  | SSX2B |  |
|  | FAM156B |  |
|  | FAM156A |  |
|  | GPR173 |  |
|  | KDM5C |  |
|  | IQSEC2 |  |
|  | SMC1A |  |
|  | RIBC1 |  |
|  | HSD17B10 |  |
|  | HUWE1 |  |
|  | PHF8 |  |
|  | FAM120C |  |
|  | WNK3 |  |

|  | TSR2 |  |
| --- | --- | --- |
|  | FGD1 |  |
|  | GNL3L |  |
|  | ITIH6 |  |
|  | TRO |  |
|  | PFKFB1 |  |
|  | APEX2 |  |
|  | ALAS2 |  |
|  | FAM104B |  |
|  | MTRNR2L10 |  |
|  | PAGE3 |  |
|  | MAGEH1 |  |
|  | USP51 |  |
|  | RRAGB |  |
|  | KLF8 |  |
|  | UBQLN2 |  |
|  | NBDY |  |
|  | ZXDB |  |
|  | NLRP2B |  |
|  | SPIN4 |  |
|  | ARHGEF9 |  |
|  | AMER1 |  |
|  | ASB12 |  |
|  | MTMR8 |  |
|  | ZC4H2 |  |
|  | ZC3H12B |  |
|  | LAS1L |  |
|  | MSN |  |
|  | VSIG4 |  |
|  | HEPH |  |
|  | AR |  |
|  | OPHN1 |  |
|  | STARD8 |  |
|  | EFNB1 |  |
|  | PJA1 |  |
|  | FAM155B |  |
|  | EDA |  |
|  | OTUD6A |  |
|  | IGBP1 |  |
|  | DGAT2L6 |  |
|  | AWAT1 |  |
|  | P2RY4 |  |
|  | ARR3 |  |
|  | RAB41 |  |
|  | KIF4A |  |
|  | GDPD2 |  |
|  | DLG3 |  |
|  | TEX11 |  |
|  | SLC7A3 |  |
|  | SNX12 |  |
|  | CXorf65 |  |
|  | IL2RG |  |
|  | NLGN3 |  |

|  | ZMYM3 |  |
| --- | --- | --- |
|  | NONO |  |
|  | ITGB1BP2 |  |
|  | TAF1 |  |
|  | OGT |  |
|  | GCNA |  |
|  | CXCR3 |  |
|  | CXorf49 |  |
|  | CXorf49B |  |
|  | NHSL2 |  |
|  | RTL5 |  |
|  | HDAC8 |  |
|  | PHKA1 |  |
|  | DMRTC1B |  |
|  | DMRTC1 |  |
|  | PABPC1L2B |  |
|  | PABPC1L2A |  |
|  | NAP1L2 |  |
|  | SLC16A2 |  |
|  | RLIM |  |
|  | NEXMIF |  |
|  | ABCB7 |  |
|  | UPRT |  |
|  | ZDHHC15 |  |
|  | MAGEE2 |  |
|  | PBDC1 |  |
|  | MAGEE1 |  |
|  | FGF16 |  |
|  | LCMT1 |  |
|  | AQP8 |  |
|  | ZKSCAN2 |  |
|  | KDM8 |  |
|  | NSMCE1 |  |
|  | IL4R |  |
|  | IL21R |  |
|  | GTF3C1 |  |
|  | KIAA0556 |  |
|  | GSG1L |  |
|  | XPO6 |  |
|  | SBK1 |  |
|  | NPIPB6 |  |
|  | EIF3CL |  |
|  | CLN3 |  |
|  | IL27 |  |
|  | NUPR1 |  |
|  | SULT1A1 |  |
|  | NPIPB8 |  |
|  | EIF3C |  |
|  | NPIPB9 |  |
|  | ATXN2L |  |
|  | TUFM |  |
|  | SH2B1 |  |
|  | ATP2A1 |  |

|  | RABEP2 |  |
| --- | --- | --- |
|  | CD19 |  |
|  | NFATC2IP |  |
|  | SPNS1 |  |
|  | LAT |  |
|  | NPIPB11 |  |
|  | BOLA2-SMG1P6 |  |
|  | BOLA2 |  |
|  | SLX1B |  |
|  | SULT1A4 |  |
|  | SPN |  |
|  | QPRT |  |
|  | C16orf54 |  |
|  | ZG16 |  |
|  | KIF22 |  |
|  | MAZ |  |
|  | PRRT2 |  |
|  | PAGR1 |  |
|  | MVP |  |
|  | CDIPT |  |
|  | SEZ6L2 |  |
|  | KCTD13 |  |
|  | TMEM219 |  |
|  | TAOK2 |  |
|  | HIRIP3 |  |
|  | INO80E |  |
|  | DOC2A |  |
|  | C16orf92 |  |
|  | FAM57B |  |
|  | ALDOA |  |
|  | TBX6 |  |
|  | YPEL3 |  |
|  | GDPD3 |  |
|  | MAPK3 |  |
|  | CORO1A |  |
|  | BOLA2B |  |
|  | SLX1A |  |
|  | SULT1A3 |  |
|  | NPIPB13 |  |
|  | CD2BP2 |  |
|  | 1-Sep |  |
|  | ZNF48 |  |
|  | ZNF771 |  |
|  | DCTPP1 |  |
|  | SEPHS2 |  |
|  | ZNF768 |  |
|  | ZNF747 |  |
|  | ZNF764 |  |
|  | ZNF688 |  |
|  | ZNF785 |  |
|  | ZNF689 |  |
|  | PRR14 |  |
|  | FBRS |  |

|  | SRCAP |  |
| --- | --- | --- |
|  | TMEM265 |  |
|  | CCDC189 |  |
|  | RNF40 |  |
|  | ZNF629 |  |
|  | BCL7C |  |
|  | CTF1 |  |
|  | ORAI3 |  |
|  | SETD1A |  |
|  | HSD3B7 |  |
|  | STX1B |  |
|  | STX4 |  |
|  | ZNF668 |  |
|  | ZNF646 |  |
|  | PRSS53 |  |
|  | VKORC1 |  |
|  | BCKDK |  |
|  | KAT8 |  |
|  | PRSS8 |  |
|  | PRSS36 |  |
|  | FUS |  |
|  | PYCARD |  |
|  | PYDC1 |  |
|  | ITGAM |  |
|  | ITGAX |  |
|  | ITGAD |  |
|  | COX6A2 |  |
|  | ZNF843 |  |
|  | ARMC5 |  |
|  | TGFB1I1 |  |
|  | SLC5A2 |  |
|  | C16orf58 |  |
|  | AHSP |  |
|  | ZNF720 |  |
|  | LOC107983990 |  |
|  | ZNF267 |  |
|  | TP53TG3D |  |
|  | TP53TG3 |  |
|  | TP53TG3C |  |
|  | TP53TG3E |  |
|  | TP53TG3B |  |
|  | TP53TG3F |  |
|  | SHCBP1 |  |
|  | VPS35 |  |
|  | MYLK3 |  |
|  | C16orf87 |  |
|  | GPT2 |  |
|  | NETO2 |  |
|  | ITFG1 |  |
|  | PHKB |  |
|  | ABCC12 |  |
|  | ABCC11 |  |
|  | LONP2 |  |

|  | SIAH1 |  |
| --- | --- | --- |
|  | N4BP1 |  |
|  | C16orf78 |  |
|  | ZNF423 |  |
|  | CNEP1R1 |  |
|  | HEATR3 |  |
|  | PAPD5 |  |
|  | ADCY7 |  |
|  | BRD7 |  |
|  | NKD1 |  |
|  | SNX20 |  |
|  | NOD2 |  |
|  | CYLD |  |
|  | SALL1 |  |
|  | TOX3 |  |
|  | CHD9 |  |
|  | RBL2 |  |
|  | AKTIP |  |
|  | RPGRIP1L |  |
|  | FTO |  |
|  | IRX3 |  |
|  | IRX5 |  |
|  | IRX6 |  |
|  | MMP2 |  |
|  | LPCAT2 |  |
|  | CAPNS2 |  |
|  | SLC6A2 |  |
|  | CES5A |  |
|  | GNAO1 |  |
|  | AMFR |  |
|  | NUDT21 |  |
|  | OGFOD1 |  |
|  | MT4 |  |
|  | MT3 |  |
|  | MT2A |  |
|  | MT1M |  |
|  | MT1G |  |
|  | MT1H |  |
|  | MT1X |  |
|  | NUP93 |  |
|  | SLC12A3 |  |
|  | CETP |  |
|  | NLRC5 |  |
|  | CPNE2 |  |
|  | FAM192A |  |
|  | RSPRY1 |  |
|  | ARL2BP |  |
|  | PLLP |  |
|  | CCL22 |  |
|  | CX3CL1 |  |
|  | CIAPIN1 |  |
|  | COQ9 |  |
|  | POLR2C |  |

|  | DOK4 |  |
| --- | --- | --- |
|  | ADGRG5 |  |
|  | ADGRG1 |  |
|  | ADGRG3 |  |
|  | DRC7 |  |
|  | KATNB1 |  |
|  | KIFC3 |  |
|  | CNGB1 |  |
|  | TEPP |  |
|  | ZNF319 |  |
|  | USB1 |  |
|  | IL31RA |  |
|  | IL6ST |  |
|  | ANKRD55 |  |
|  | SETD9 |  |
|  | MIER3 |  |
|  | GPBP1 |  |
|  | ACTBL2 |  |
|  | PLK2 |  |
|  | RAB3C |  |
|  | PDE4D |  |
|  | ELOVL7 |  |
|  | ERCC8 |  |
|  | NDUFAF2 |  |
|  | IPO11 |  |
|  | RGS7BP |  |
|  | SREK1IP1 |  |
|  | ADAMTS6 |  |
|  | CENPK |  |
|  | TRIM23 |  |
|  | TRAPPC13 |  |
|  | SGTB |  |
|  | NLN |  |
|  | ERBIN |  |
|  | SREK1 |  |
|  | MAST4 |  |
|  | CD180 |  |
|  | PIK3R1 |  |
|  | SLC30A5 |  |
|  | CCNB1 |  |
|  | CENPH |  |
|  | MRPS36 |  |
|  | CCDC125 |  |
|  | AK6 |  |
|  | TAF9 |  |
|  | RAD17 |  |
|  | MARVELD2 |  |
|  | OCLN |  |
|  | SERF1B |  |
|  | SMN2 |  |
|  | SERF1A |  |
|  | SMN1 |  |
|  | NAIP |  |

|  | GTF2H2 |  |
| --- | --- | --- |
|  | BDP1 |  |
|  | MCCC2 |  |
|  | MAP1B |  |
|  | MRPS27 |  |
|  | ZNF366 |  |
|  | TNPO1 |  |
|  | TMEM171 |  |
|  | BTF3 |  |
|  | UTP15 |  |
|  | ARHGEF28 |  |
|  | ENC1 |  |
|  | HEXB |  |
|  | GFM2 |  |
|  | NSA2 |  |
|  | FAM169A |  |
|  | ANKRD31 |  |
|  | HMGCR |  |
|  | COL4A3BP |  |
|  | POLK |  |
|  | ANKDD1B |  |
|  | SV2C |  |
|  | IQGAP2 |  |
|  | F2RL2 |  |
|  | F2R |  |
|  | S100Z |  |
|  | CRHBP |  |
|  | ZBED3 |  |
|  | PDE8B |  |
|  | WDR41 |  |
|  | TBCA |  |
|  | AP3B1 |  |
|  | LHFPL2 |  |
|  | ARSB |  |
|  | DMGDH |  |
|  | BHMT2 |  |
|  | JMY |  |
|  | HOMER1 |  |
|  | PAPD4 |  |
|  | CMYA5 |  |
|  | MTX3 |  |
|  | THBS4 |  |
|  | SERINC5 |  |
|  | SPZ1 |  |
|  | ZFYVE16 |  |
|  | ANKRD34B |  |
|  | DHFR |  |
|  | MSH3 |  |
|  | RASGRF2 |  |
|  | CKMT2 |  |
|  | ZCCHC9 |  |
|  | ACOT12 |  |
|  | HSPD1 |  |

|  | HSPE1-MOB4 |  |
| --- | --- | --- |
|  | HSPE1 |  |
|  | C2orf83 |  |
|  | SLC19A3 |  |
|  | DAW1 |  |
|  | SPHKAP |  |
|  | PID1 |  |
|  | DNER |  |
|  | TRIP12 |  |
|  | FBXO36 |  |
|  | SLC16A14 |  |
|  | SP110 |  |
|  | SP140 |  |
|  | SP140L |  |
|  | SP100 |  |
|  | CAB39 |  |
|  | ITM2C |  |
|  | GPR55 |  |
|  | SPATA3 |  |
|  | C2orf72 |  |
|  | PSMD1 |  |
|  | ARMC9 |  |
|  | NMUR1 |  |
|  | COPS7B |  |
|  | DIS3L2 |  |
|  | ALPP |  |
|  | ALPPL2 |  |
|  | ALPI |  |
|  | ECEL1 |  |
|  | CHRND |  |
|  | CHRNG |  |
|  | TIGD1 |  |
|  | EIF4E2 |  |
|  | GIGYF2 |  |
|  | KCNJ13 |  |
|  | SNORC |  |
|  | NGEF |  |
|  | NEU2 |  |
|  | INPP5D |  |
|  | ATG16L1 |  |
|  | SAG |  |
|  | USP40 |  |
|  | UGT1A8 |  |
|  | UGT1A10 |  |
|  | UGT1A9 |  |
|  | UGT1A7 |  |
|  | UGT1A6 |  |
|  | UGT1A5 |  |
|  | UGT1A4 |  |
|  | UGT1A3 |  |
|  | UGT1A1 |  |
|  | MROH2A |  |
|  | HJURP |  |

|  | TRPM8 |  |
| --- | --- | --- |
|  | SPP2 |  |
|  | ARL4C |  |
|  | SH3BP4 |  |
|  | AGAP1 |  |
|  | GBX2 |  |
|  | ASB18 |  |
|  | IQCA1 |  |
|  | COL6A3 |  |
|  | MLPH |  |
|  | LRRFIP1 |  |
|  | RBM44 |  |
|  | RAMP1 |  |
|  | ESPNL |  |
|  | ERFE |  |
|  | ILKAP |  |
|  | HES6 |  |
|  | PER2 |  |
|  | TRAF3IP1 |  |
|  | ASB1 |  |
|  | TWIST2 |  |
|  | HDAC4 |  |
|  | NDUFA10 |  |
|  | OR6B2 |  |
|  | OR6B3 |  |
|  | OTOS |  |
|  | GPC1 |  |
|  | ANKMY1 |  |
|  | DUSP28 |  |
|  | RNPEPL1 |  |
|  | CAPN10 |  |
|  | GPR35 |  |
|  | AQP12B |  |
|  | AGXT |  |
|  | C2orf54 |  |
|  | CROCC2 |  |
|  | SNED1 |  |
|  | MTERF4 |  |
|  | PPP1R7 |  |
|  | ANO7 |  |
|  | HDLBP |  |
|  | 2-Sep |  |
|  | FARP2 |  |
|  | STK25 |  |
|  | THAP4 |  |
|  | ATG4B |  |
|  | ING5 |  |
|  | GAL3ST2 |  |
|  | NEU4 |  |
|  | PDCD1 |  |
|  | RTP5 |  |
|  | CHL1 |  |
|  | CNTN6 |  |

|  | CNTN4 |  |
| --- | --- | --- |
|  | IL5RA |  |
|  | TRNT1 |  |
|  | LRRN1 |  |
|  | SETMAR |  |
|  | SUMF1 |  |
|  | ITPR1 |  |
|  | BHLHE40 |  |
|  | EDEM1 |  |
|  | GRM7 |  |
|  | SSUH2 |  |
|  | OXTR |  |
|  | RAD18 |  |
|  | SRGAP3 |  |
|  | THUMPD3 |  |
|  | SETD5 |  |
|  | LHFPL4 |  |
|  | CPNE9 |  |
|  | BRPF1 |  |
|  | OGG1 |  |
|  | CAMK1 |  |
|  | TADA3 |  |
|  | ARPC4 |  |
|  | ARPC4-TTLL3 |  |
|  | TTLL3 |  |
|  | RPUSD3 |  |
|  | CIDEC |  |
|  | CRELD1 |  |
|  | PRRT3 |  |
|  | EMC3 |  |
|  | FANCD2 |  |
|  | SLC49A3 |  |
|  | PCGF3 |  |
|  | CPLX1 |  |
|  | GAK |  |
|  | TMEM175 |  |
|  | DGKQ |  |
|  | SLC26A1 |  |
|  | IDUA |  |
|  | FGFRL1 |  |
|  | SPON2 |  |
|  | CTBP1 |  |
|  | MAEA |  |
|  | UVSSA |  |
|  | FAM53A |  |
|  | TMEM129 |  |
|  | TACC3 |  |
|  | FGFR3 |  |
|  | NSD2 |  |
|  | NAT8L |  |
|  | POLN |  |
|  | HAUS3 |  |
|  | MXD4 |  |

|  | ZFYVE28 |  |
| --- | --- | --- |
|  | CFAP99 |  |
|  | RNF4 |  |
|  | FAM193A |  |
|  | TNIP2 |  |
|  | SH3BP2 |  |
|  | ADD1 |  |
|  | MFSD10 |  |
|  | NOP14 |  |
|  | GRK4 |  |
|  | HTT |  |
|  | MSANTD1 |  |
|  | RGS12 |  |
|  | HGFAC |  |
|  | DOK7 |  |
|  | LRPAP1 |  |
|  | OTOP1 |  |
|  | TMEM128 |  |
|  | LYAR |  |
|  | ZBTB49 |  |
|  | NSG1 |  |
|  | STX18 |  |
|  | MSX1 |  |
|  | CYTL1 |  |
|  | STK32B |  |
|  | EVC2 |  |
|  | EVC |  |
|  | CRMP1 |  |
|  | JAKMIP1 |  |
|  | WFS1 |  |
|  | PPP2R2C |  |
|  | MAN2B2 |  |
|  | MRFAP1 |  |
|  | S100P |  |
|  | KIAA0232 |  |
|  | TBC1D14 |  |
|  | CCDC96 |  |
|  | TADA2B |  |
|  | SORCS2 |  |
|  | PSAPL1 |  |
|  | AFAP1 |  |
|  | ABLIM2 |  |
|  | SH3TC1 |  |
|  | ACOX3 |  |
|  | TRMT44 |  |
|  | GPR78 |  |
|  | CPZ |  |
|  | HMX1 |  |
|  | FAM90A26 |  |
|  | USP17L10 |  |
|  | USP17L11 |  |
|  | USP17L12 |  |
|  | USP17L13 |  |

|  | USP17L15 |  |
| --- | --- | --- |
|  | USP17L17 |  |
|  | USP17L18 |  |
|  | USP17L19 |  |
|  | USP17L20 |  |
|  | USP17L21 |  |
|  | USP17L22 |  |
|  | USP17L24 |  |
|  | USP17L25 |  |
|  | USP17L26 |  |
|  | USP17L5 |  |
|  | USP17L27 |  |
|  | USP17L28 |  |
|  | USP17L29 |  |
|  | USP17L30 |  |
|  | DRD5 |  |
|  | SLC2A9 |  |
|  | WDR1 |  |
|  | ZNF518B |  |
|  | CLNK |  |
|  | RAB28 |  |
|  | BOD1L1 |  |
|  | CPEB2 |  |
|  | CC2D2A |  |
|  | FBXL5 |  |
|  | BST1 |  |
|  | CD38 |  |
|  | FGFBP2 |  |
|  | PROM1 |  |
|  | TAPT1 |  |
|  | LDB2 |  |
|  | CLRN2 |  |
|  | LAP3 |  |
|  | FAM184B |  |
|  | DCAF16 |  |
|  | LCORL |  |
|  | SLIT2 |  |
|  | PACRGL |  |
|  | PPARGC1A |  |
|  | DHX15 |  |
|  | SOD3 |  |
|  | CCDC149 |  |
|  | LGI2 |  |
|  | PI4K2B |  |
|  | ZCCHC4 |  |
|  | SLC34A2 |  |
|  | SEL1L3 |  |
|  | CCKAR |  |
|  | TBC1D19 |  |
|  | STIM2 |  |
|  | PCDH7 |  |
|  | ARAP2 |  |
|  | DTHD1 |  |

|  | NWD2 |  |
| --- | --- | --- |
|  | C4orf19 |  |
|  | RELL1 |  |
|  | TBC1D1 |  |
|  | KLF3 |  |
|  | TLR10 |  |
|  | TLR1 |  |
|  | TLR6 |  |
|  | FAM114A1 |  |
|  | TMEM156 |  |
|  | KLHL5 |  |
|  | WDR19 |  |
|  | RFC1 |  |
|  | KLB |  |
|  | LIAS |  |
|  | UGDH |  |
|  | SMIM14 |  |
|  | PDS5A |  |
|  | N4BP2 |  |
|  | RHOH |  |
|  | CHRNA9 |  |
|  | RBM47 |  |
|  | HSPB7 |  |
|  | CLCNKA |  |
|  | CLCNKB |  |
|  | FAM131C |  |
|  | EPHA2 |  |
|  | ARHGEF19 |  |
|  | RSG1 |  |
|  | FBXO42 |  |
|  | SZRD1 |  |
|  | SPATA21 |  |
|  | NECAP2 |  |
|  | NBPF1 |  |
|  | MFAP2 |  |
|  | ATP13A2 |  |
|  | PADI2 |  |
|  | PADI1 |  |
|  | PADI3 |  |
|  | PADI4 |  |
|  | PADI6 |  |
|  | RCC2 |  |
|  | ARHGEF10L |  |
|  | ACTL8 |  |
|  | IGSF21 |  |
|  | KLHDC7A |  |
|  | PAX7 |  |
|  | TAS1R2 |  |
|  | ALDH4A1 |  |
|  | IFFO2 |  |
|  | UBR4 |  |
|  | EMC1 |  |
|  | MRTO4 |  |

|  | AKR7A2 |  |
| --- | --- | --- |
|  | PQLC2 |  |
|  | CAPZB |  |
|  | MINOS1-NBL1 |  |
|  | MINOS1 |  |
|  | NBL1 |  |
|  | HTR6 |  |
|  | TMCO4 |  |
|  | RNF186 |  |
|  | OTUD3 |  |
|  | PLA2G2A |  |
|  | PLA2G5 |  |
|  | PLA2G2D |  |
|  | PLA2G2F |  |
|  | UBXN10 |  |
|  | CAMK2N1 |  |
|  | MUL1 |  |
|  | FAM43B |  |
|  | PINK1 |  |
|  | DDOST |  |
|  | KIF17 |  |
|  | HP1BP3 |  |
|  | EIF4G3 |  |
|  | ECE1 |  |
|  | NBPF3 |  |
|  | ALPL |  |
|  | RAP1GAP |  |
|  | LDLRAD2 |  |
|  | HSPG2 |  |
|  | CELA3B |  |
|  | CELA3A |  |
|  | CDC42 |  |
|  | WNT4 |  |
|  | ZBTB40 |  |
|  | EPHA8 |  |
|  | C1QA |  |
|  | C1QC |  |
|  | EPHB2 |  |
|  | LACTBL1 |  |
|  | TEX46 |  |
|  | LUZP1 |  |
|  | HTR1D |  |
|  | HNRNPR |  |
|  | ZNF436 |  |
|  | ASAP3 |  |
|  | E2F2 |  |
|  | ID3 |  |
|  | RPL11 |  |
|  | ELOA |  |
|  | PITHD1 |  |
|  | LYPLA2 |  |
|  | GALE |  |
|  | FUCA1 |  |

|  | CNR2 |  |
| --- | --- | --- |
|  | PNRC2 |  |
|  | SRSF10 |  |
|  | MYOM3 |  |
|  | IL22RA1 |  |
|  | IFNLR1 |  |
|  | GRHL3 |  |
|  | STPG1 |  |
|  | NIPAL3 |  |
|  | RCAN3 |  |
|  | NCMAP |  |
|  | SRRM1 |  |
|  | CLIC4 |  |
|  | RUNX3 |  |
|  | SYF2 |  |
|  | RSRP1 |  |
|  | RHD |  |
|  | MACO1 |  |
|  | LDLRAP1 |  |
|  | MAN1C1 |  |
|  | SELENON |  |
|  | MTFR1L |  |
|  | AUNIP |  |
|  | PAQR7 |  |
|  | STMN1 |  |
|  | PAFAH2 |  |
|  | EXTL1 |  |
|  | SLC30A2 |  |
|  | PDIK1L |  |
|  | ZNF593 |  |
|  | CNKSR1 |  |
|  | CATSPER4 |  |
|  | CEP85 |  |
|  | SH3BGRL3 |  |
|  | UBXN11 |  |
|  | CRYBG2 |  |
|  | ZNF683 |  |
|  | LIN28A |  |
|  | DHDDS |  |
|  | HMGN2 |  |
|  | RPS6KA1 |  |
|  | ARID1A |  |
|  | ZDHHC18 |  |
|  | SFN |  |
|  | GPN2 |  |
|  | GPATCH3 |  |
|  | NR0B2 |  |
|  | NUDC |  |
|  | TRNP1 |  |
|  | SLC9A1 |  |
|  | WDTC1 |  |
|  | TMEM222 |  |
|  | MAP3K6 |  |

|  | FCN3 |  |
| --- | --- | --- |
|  | CD164L2 |  |
|  | GPR3 |  |
|  | WASF2 |  |
|  | AHDC1 |  |
|  | IFI6 |  |
|  | FAM76A |  |
|  | STX12 |  |
|  | PPP1R8 |  |
|  | THEMIS2 |  |
|  | RPA2 |  |
|  | SMPDL3B |  |
|  | XKR8 |  |
|  | EYA3 |  |
|  | PTAFR |  |
|  | DNAJC8 |  |
|  | ATP5IF1 |  |
|  | SESN2 |  |
|  | MED18 |  |
|  | PHACTR4 |  |
|  | RCC1 |  |
|  | RAB42 |  |
|  | GMEB1 |  |
|  | YTHDF2 |  |
|  | OPRD1 |  |
|  | EPB41 |  |
|  | TMEM200B |  |
|  | SRSF4 |  |
|  | MECR |  |
|  | MATN1 |  |
|  | SDC3 |  |
|  | PUM1 |  |
|  | NKAIN1 |  |
|  | SNRNP40 |  |
|  | ERAL1 |  |
|  | FLOT2 |  |
|  | DHRS13 |  |
|  | PHF12 |  |
|  | SEZ6 |  |
|  | PIPOX |  |
|  | MYO18A |  |
|  | TIAF1 |  |
|  | NUFIP2 |  |
|  | TAOK1 |  |
|  | ABHD15 |  |
|  | GIT1 |  |
|  | SSH2 |  |
|  | EFCAB5 |  |
|  | NSRP1 |  |
|  | BLMH |  |
|  | CPD |  |
|  | GOSR1 |  |
|  | ATAD5 |  |

|  | ADAP2 |  |
| --- | --- | --- |
|  | RNF135 |  |
|  | NF1 |  |
|  | OMG |  |
|  | EVI2A |  |
|  | RAB11FIP4 |  |
|  | COPRS |  |
|  | LRRC37B |  |
|  | RHOT1 |  |
|  | RHBDL3 |  |
|  | C17orf75 |  |
|  | ZNF207 |  |
|  | PSMD11 |  |
|  | MYO1D |  |
|  | TMEM98 |  |
|  | ASIC2 |  |
|  | CCL2 |  |
|  | CCL7 |  |
|  | CCL11 |  |
|  | CCL8 |  |
|  | CCL1 |  |
|  | TMEM132E |  |
|  | ZNF830 |  |
|  | LIG3 |  |
|  | RFFL |  |
|  | RAD51D |  |
|  | FNDC8 |  |
|  | NLE1 |  |
|  | UNC45B |  |
|  | SLC35G3 |  |
|  | SLFN5 |  |
|  | SLFN11 |  |
|  | SLFN12 |  |
|  | SLFN13 |  |
|  | SLFN12L |  |
|  | SLFN14 |  |
|  | PEX12 |  |
|  | AP2B1 |  |
|  | RASL10B |  |
|  | GAS2L2 |  |
|  | MMP28 |  |
|  | TAF15 |  |
|  | HEATR9 |  |
|  | CCL5 |  |
|  | RDM1 |  |
|  | LYZL6 |  |
|  | CCL16 |  |
|  | CCL14 |  |
|  | CCL15 |  |
|  | CCL23 |  |
|  | CCL18 |  |
|  | CCL4 |  |
|  | TBC1D3B |  |

|  | TBC1D3I |  |
| --- | --- | --- |
|  | TBC1D3G |  |
|  | TBC1D3H |  |
|  | TBC1D3F |  |
|  | ZNHIT3 |  |
|  | MYO19 |  |
|  | GGNBP2 |  |
|  | AATF |  |
|  | ACACA |  |
|  | C17orf78 |  |
|  | TADA2A |  |
|  | DUSP14 |  |
|  | SYNRG |  |
|  | DDX52 |  |
|  | HNF1B |  |
|  | TBC1D3K |  |
|  | TBC1D3L |  |
|  | TBC1D3D |  |
|  | TBC1D3C |  |
|  | TBC1D3E |  |
|  | TBC1D3 |  |
|  | MRPL45 |  |
|  | GPR179 |  |
|  | SOCS7 |  |
|  | ARHGAP23 |  |
|  | SRCIN1 |  |
|  | EPOP |  |
|  | MLLT6 |  |
|  | CISD3 |  |
|  | PCGF2 |  |
|  | PIP4K2B |  |
|  | CWC25 |  |
|  | LASP1 |  |
|  | FBXO47 |  |
|  | PLXDC1 |  |
|  | ARL5C |  |
|  | CACNB1 |  |
|  | RPL19 |  |
|  | STAC2 |  |
|  | FBXL20 |  |
|  | MED1 |  |
|  | CDK12 |  |
|  | NEUROD2 |  |
|  | PPP1R1B |  |
|  | STARD3 |  |
|  | PGAP3 |  |
|  | ERBB2 |  |
|  | MIEN1 |  |
|  | GRB7 |  |
|  | IKZF3 |  |
|  | ZPBP2 |  |
|  | GSDMB |  |
|  | ORMDL3 |  |

|  | GSDMA |  |
| --- | --- | --- |
|  | CSF3 |  |
|  | MED24 |  |
|  | THRA |  |
|  | NR1D1 |  |
|  | MSL1 |  |
|  | CASC3 |  |
|  | RAPGEFL1 |  |
|  | WIPF2 |  |
|  | CDC6 |  |
|  | RARA |  |
|  | GJD3 |  |
|  | TOP2A |  |
|  | IGFBP4 |  |
|  | TNS4 |  |
|  | SMARCE1 |  |
|  | KRT24 |  |
|  | KRT25 |  |
|  | KRT27 |  |
|  | KRT28 |  |
|  | KRT12 |  |
|  | KRT23 |  |
|  | KRT39 |  |
|  | KRT40 |  |
|  | KRTAP3-3 |  |
|  | KRTAP3-2 |  |
|  | KRTAP3-1 |  |
|  | KRTAP1-5 |  |
|  | KRTAP1-4 |  |
|  | KRTAP1-3 |  |
|  | KRTAP2-1 |  |
|  | KRTAP2-2 |  |
|  | KRTAP2-4 |  |
|  | KRTAP4-7 |  |
|  | KRTAP4-8 |  |
|  | KRTAP4-9 |  |
|  | KRTAP4-11 |  |
|  | KRTAP4-12 |  |
|  | KRTAP4-6 |  |
|  | KRTAP4-5 |  |
|  | KRTAP4-4 |  |
|  | KRTAP4-2 |  |
|  | KRTAP9-2 |  |
|  | KRTAP9-3 |  |
|  | KRTAP9-4 |  |
|  | KRTAP9-7 |  |
|  | KRTAP29-1 |  |
|  | KRTAP16-1 |  |
|  | KRTAP17-1 |  |
|  | KRT34 |  |
|  | KRT31 |  |
|  | KRT37 |  |
|  | KRT32 |  |

|  | KRT35 |  |
| --- | --- | --- |
|  | KRT13 |  |
|  | KRT15 |  |
|  | KRT19 |  |
|  | KRT9 |  |
|  | KRT14 |  |
|  | KRT16 |  |
|  | KRT17 |  |
|  | HAP1 |  |
|  | JUP |  |
|  | FKBP10 |  |
|  | NT5C3B |  |
|  | KLHL10 |  |
|  | KLHL11 |  |
|  | ACLY |  |
|  | TTC25 |  |
|  | CNP |  |
|  | DNAJC7 |  |
|  | NKIRAS2 |  |
|  | C17orf113 |  |
|  | DHX58 |  |
|  | HSPB9 |  |
|  | FBXO25 |  |
|  | TDRP |  |
|  | ERICH1 |  |
|  | DLGAP2 |  |
|  | CLN8 |  |
|  | ARHGEF10 |  |
|  | KBTBD11 |  |
|  | MYOM2 |  |
|  | CSMD1 |  |
|  | MCPH1 |  |
|  | ANGPT2 |  |
|  | XKR5 |  |
|  | DEFB1 |  |
|  | DEFA1 |  |
|  | DEFA1B |  |
|  | DEFA3 |  |
|  | USP17L1 |  |
|  | USP17L4 |  |
|  | SPAG11B |  |
|  | PRR23D1 |  |
|  | PRR23D2 |  |
|  | SPAG11A |  |
|  | USP17L8 |  |
|  | USP17L3 |  |
|  | PRAG1 |  |
|  | MFHAS1 |  |
|  | ERI1 |  |
|  | PPP1R3B |  |
|  | TNKS |  |
|  | MSRA |  |
|  | PRSS55 |  |

|  | RP1L1 |  |
| --- | --- | --- |
|  | C8orf74 |  |
|  | SOX7 |  |
|  | PINX1 |  |
|  | XKR6 |  |
|  | MTMR9 |  |
|  | FAM167A |  |
|  | BLK |  |
|  | GATA4 |  |
|  | NEIL2 |  |
|  | FDFT1 |  |
|  | CTSB |  |
|  | DEFB134 |  |
|  | ZNF705D |  |
|  | USP17L7 |  |
|  | USP17L2 |  |
|  | FAM86B1 |  |
|  | FAM86B2 |  |
|  | TRMT9B |  |
|  | DLC1 |  |
|  | C8orf48 |  |
|  | SGCZ |  |
|  | TUSC3 |  |
|  | MSR1 |  |
|  | ZDHHC2 |  |
|  | VPS37A |  |
|  | MTMR7 |  |
|  | SLC7A2 |  |
|  | PDGFRL |  |
|  | MTUS1 |  |
|  | FGL1 |  |
|  | PCM1 |  |
|  | ASAH1 |  |
|  | NAT1 |  |
|  | NAT2 |  |
|  | PSD3 |  |
|  | SH2D4A |  |
|  | CSGALNACT1 |  |
|  | INTS10 |  |
|  | LPL |  |
|  | SLC18A1 |  |
|  | ATP6V1B2 |  |
|  | LZTS1 |  |
|  | GFRA2 |  |
|  | DOK2 |  |
|  | XPO7 |  |
|  | NPM2 |  |
|  | DMTN |  |
|  | FAM160B2 |  |
|  | HR |  |
|  | REEP4 |  |
|  | LGI3 |  |
|  | SFTPC |  |

|  | BMP1 |  |
| --- | --- | --- |
|  | PHYHIP |  |
|  | POLR3D |  |
|  | PIWIL2 |  |
|  | SLC39A14 |  |
|  | PPP3CC |  |
|  | SORBS3 |  |
|  | PDLIM2 |  |
|  | CCAR2 |  |
|  | BIN3 |  |
|  | EGR3 |  |
|  | RHOBTB2 |  |
|  | TNFRSF10B |  |
|  | TNFRSF10C |  |
|  | TNFRSF10D |  |
|  | TNFRSF10A |  |
|  | CHMP7 |  |
|  | R3HCC1 |  |
|  | LOXL2 |  |
|  | ENTPD4 |  |
|  | SLC25A37 |  |
|  | NKX3-1 |  |
|  | NKX2-6 |  |
|  | STC1 |  |
|  | ADAM28 |  |
|  | NEFM |  |
|  | NEFL |  |
|  | DOCK5 |  |
|  | GNRH1 |  |
|  | KCTD9 |  |
|  | CDCA2 |  |
|  | EBF2 |  |
|  | PPP2R2A |  |
|  | PNMA2 |  |
|  | DPYSL2 |  |
|  | ADRA1A |  |
|  | STMN4 |  |
|  | TRIM35 |  |
|  | PTK2B |  |
|  | CHRNA2 |  |
|  | EPHX2 |  |
|  | CLU |  |
|  | SCARA3 |  |
|  | CCDC25 |  |
|  | ESCO2 |  |
|  | PBK |  |
|  | SCARA5 |  |
|  | NUGGC |  |
|  | ELP3 |  |
|  | PNOC |  |
|  | ZNF395 |  |
|  | FBXO16 |  |
|  | FZD3 |  |

|  | INTS9 |  |
| --- | --- | --- |
|  | HMBOX1 |  |
|  | KIF13B |  |
|  | DUSP4 |  |
|  | SARAF |  |
|  | LEPROTL1 |  |
|  | DCTN6 |  |
|  | RBPMS |  |
|  | SMIM18 |  |
|  | UBXN8 |  |
|  | PPP2CB |  |
|  | PURG |  |
|  | WRN |  |
|  | NRG1 |  |
|  | FUT10 |  |
|  | MAK16 |  |
|  | TTI2 |  |
|  | RNF122 |  |
|  | UNC5D |  |
|  | KCNU1 |  |
|  | ZNF703 |  |
|  | RFLNB |  |
|  | TNFRSF12A |  |
|  | HCFC1R1 |  |
|  | BICDL2 |  |
|  | MMP25 |  |
|  | ZSCAN10 |  |
|  | ZNF205 |  |
|  | ZNF213 |  |
|  | ZNF200 |  |
|  | MEFV |  |
|  | ZNF263 |  |
|  | TIGD7 |  |
|  | ZNF75A |  |
|  | MTRNR2L4 |  |
|  | ZSCAN32 |  |
|  | ZNF174 |  |
|  | NAA60 |  |
|  | C16orf90 |  |
|  | CLUAP1 |  |
|  | NLRC3 |  |
|  | SLX4 |  |
|  | DNASE1 |  |
|  | TRAP1 |  |
|  | CREBBP |  |
|  | ADCY9 |  |
|  | SRL |  |
|  | GLIS2 |  |
|  | PAM16 |  |
|  | CORO7-PAM16 |  |
|  | CORO7 |  |
|  | VASN |  |
|  | NMRAL1 |  |

|  | HMOX2 |  |
| --- | --- | --- |
|  | CDIP1 |  |
|  | C16orf96 |  |
|  | UBALD1 |  |
|  | MGRN1 |  |
|  | NUDT16L1 |  |
|  | ANKS3 |  |
|  | C16orf71 |  |
|  | ZNF500 |  |
|  | 12-Sep |  |
|  | SMIM22 |  |
|  | ROGDI |  |
|  | GLYR1 |  |
|  | UBN1 |  |
|  | SEC14L5 |  |
|  | C16orf89 |  |
|  | ALG1 |  |
|  | EEF2KMT |  |
|  | RBFOX1 |  |
|  | TMEM114 |  |
|  | ABAT |  |
|  | TMEM186 |  |
|  | PMM2 |  |
|  | CARHSP1 |  |
|  | C16orf72 |  |
|  | GRIN2A |  |
|  | EMP2 |  |
|  | TEKT5 |  |
|  | TVP23A |  |
|  | CIITA |  |
|  | DEXI |  |
|  | CLEC16A |  |
|  | SOCS1 |  |
|  | TNP2 |  |
|  | PRM2 |  |
|  | PRM1 |  |
|  | RMI2 |  |
|  | LITAF |  |
|  | SNN |  |
|  | TXNDC11 |  |
|  | RSL1D1 |  |
|  | GSPT1 |  |
|  | NPIPB2 |  |
|  | SNX29 |  |
|  | CPPED1 |  |
|  | SHISA9 |  |
|  | ERCC4 |  |
|  | MKL2 |  |
|  | PARN |  |
|  | PLA2G10 |  |
|  | NPIPA3 |  |
|  | NPIPA2 |  |
|  | NOMO1 |  |

|  | PDXDC1 |  |
| --- | --- | --- |
|  | NTAN1 |  |
|  | RRN3 |  |
|  | NPIPA5 |  |
|  | MPV17L |  |
|  | MARF1 |  |
|  | NDE1 |  |
|  | MYH11 |  |
|  | FOPNL |  |
|  | ABCC1 |  |
|  | ABCC6 |  |
|  | NOMO3 |  |
|  | NPIPA7 |  |
|  | XYLT1 |  |
|  | LOC102723728 |  |
|  | NOMO2 |  |
|  | ARL6IP1 |  |
|  | SMG1 |  |
|  | TMC7 |  |
|  | COQ7 |  |
|  | SYT17 |  |
|  | TMC5 |  |
|  | VPS35L |  |
|  | KNOP1 |  |
|  | IQCK |  |
|  | GPRC5B |  |
|  | GP2 |  |
|  | UMOD |  |
|  | ACSM2A |  |
|  | ACSM2B |  |
|  | THUMPD1 |  |
|  | ACSM3 |  |
|  | ERI2 |  |
|  | REXO5 |  |
|  | LYRM1 |  |
|  | DNAH3 |  |
|  | TMEM159 |  |
|  | ZP2 |  |
|  | ANKS4B |  |
|  | CRYM |  |
|  | METTL9 |  |
|  | OTOA |  |
|  | NPIPB4 |  |
|  | MOSMO |  |
|  | VWA3A |  |
|  | EEF2K |  |
|  | POLR3E |  |
|  | CDR2 |  |
|  | NPIPB5 |  |
|  | USP31 |  |
|  | SCNN1G |  |
|  | SCNN1B |  |
|  | COG7 |  |

|  | GGA2 |  |
| --- | --- | --- |
|  | EARS2 |  |
|  | NDUFAB1 |  |
|  | PALB2 |  |
|  | DCTN5 |  |
|  | PLK1 |  |
|  | ERN2 |  |
|  | CHP2 |  |
|  | PRKCB |  |
|  | CACNG3 |  |
|  | RBBP6 |  |
|  | TNRC6A |  |
|  | SLC5A11 |  |
|  | ARHGAP17 |  |
|  | PDE2A |  |
|  | ARAP1 |  |
|  | FCHSD2 |  |
|  | P2RY2 |  |
|  | P2RY6 |  |
|  | ARHGEF17 |  |
|  | RELT |  |
|  | FAM168A |  |
|  | PLEKHB1 |  |
|  | RAB6A |  |
|  | COA4 |  |
|  | PAAF1 |  |
|  | DNAJB13 |  |
|  | UCP2 |  |
|  | UCP3 |  |
|  | C2CD3 |  |
|  | PPME1 |  |
|  | P4HA3 |  |
|  | PGM2L1 |  |
|  | KCNE3 |  |
|  | POLD3 |  |
|  | CHRDL2 |  |
|  | RNF169 |  |
|  | IL7 |  |
|  | STMN2 |  |
|  | HEY1 |  |
|  | TPD52 |  |
|  | ZBTB10 |  |
|  | PAG1 |  |
|  | PMP2 |  |
|  | IMPA1 |  |
|  | ZFAND1 |  |
|  | CHMP4C |  |
|  | SNX16 |  |
|  | RALYL |  |
|  | LRRCC1 |  |
|  | E2F5 |  |
|  | CA13 |  |
|  | CA1 |  |

|  | CA3 |  |
| --- | --- | --- |
|  | ATP6V0D2 |  |
|  | MMP15 |  |
|  | CFAP20 |  |
|  | CSNK2A2 |  |
|  | CCDC113 |  |
|  | GINS3 |  |
|  | NDRG4 |  |
|  | SETD6 |  |
|  | CNOT1 |  |
|  | SLC38A7 |  |
|  | GOT2 |  |
|  | CDH8 |  |
|  | CDH11 |  |
|  | CDH5 |  |
|  | BEAN1 |  |
|  | TK2 |  |
|  | CKLF-CMTM1 |  |
|  | CMTM1 |  |
|  | CMTM2 |  |
|  | CMTM3 |  |
|  | CMTM4 |  |
|  | DYNC1LI2 |  |
|  | TERB1 |  |
|  | PDP2 |  |
|  | CDH16 |  |
|  | FAM96B |  |
|  | CES2 |  |
|  | CES3 |  |
|  | CES4A |  |
|  | CBFB |  |
|  | C16orf70 |  |
|  | B3GNT9 |  |
|  | TRADD |  |
|  | FBXL8 |  |
|  | NOL3 |  |
|  | KIAA0895L |  |
|  | E2F4 |  |
|  | ELMO3 |  |
|  | LRRC29 |  |
|  | FHOD1 |  |
|  | SLC9A5 |  |
|  | PLEKHG4 |  |
|  | KCTD19 |  |
|  | TPPP3 |  |
|  | ZDHHC1 |  |
|  | HSD11B2 |  |
|  | ATP6V0D1 |  |
|  | AGRP |  |
|  | RIPOR1 |  |
|  | CTCF |  |
|  | CARMIL2 |  |
|  | ACD |  |

|  | PARD6A |  |
| --- | --- | --- |
|  | C16orf86 |  |
|  | GFOD2 |  |
|  | RANBP10 |  |
|  | TSNAXIP1 |  |
|  | CENPT |  |
|  | THAP11 |  |
|  | NUTF2 |  |
|  | EDC4 |  |
|  | NRN1L |  |
|  | PSKH1 |  |
|  | CTRL |  |
|  | LCAT |  |
|  | SLC12A4 |  |
|  | DPEP2 |  |
|  | DPEP2NB |  |
|  | DDX28 |  |
|  | NFATC3 |  |
|  | ESRP2 |  |
|  | SLC7A6 |  |
|  | SLC7A6OS |  |
|  | PRMT7 |  |
|  | SMPD3 |  |
|  | ZFP90 |  |
|  | CDH3 |  |
|  | CDH1 |  |
|  | TANGO6 |  |
|  | HAS3 |  |
|  | CHTF8 |  |
|  | UTP4 |  |
|  | SNTB2 |  |
|  | VPS4A |  |
|  | COG8 |  |
|  | NIP7 |  |
|  | TERF2 |  |
|  | CYB5B |  |
|  | NFAT5 |  |
|  | NQO1 |  |
|  | NOB1 |  |
|  | WWP2 |  |
|  | PDPR |  |
|  | CLEC18C |  |
|  | EXOSC6 |  |
|  | DDX19B |  |
|  | DDX19A |  |
|  | ST3GAL2 |  |
|  | FUK |  |
|  | COG4 |  |
|  | SF3B3 |  |
|  | IL34 |  |
|  | MTSS1L |  |
|  | VAC14 |  |
|  | HYDIN |  |

|  | CMTR2 |  |
| --- | --- | --- |
|  | CALB2 |  |
|  | ZNF23 |  |
|  | ZNF19 |  |
|  | CHST4 |  |
|  | TAT |  |
|  | MARVELD3 |  |
|  | PHLPP2 |  |
|  | AP1G1 |  |
|  | ATXN1L |  |
|  | IST1 |  |
|  | PKD1L3 |  |
|  | DHODH |  |
|  | HP |  |
|  | HPR |  |
|  | TXNL4B |  |
|  | DHX38 |  |
|  | PSMD7 |  |
|  | NPIPB15 |  |
|  | CLEC18B |  |
|  | GLG1 |  |
|  | RFWD3 |  |
|  | MLKL |  |
|  | FA2H |  |
|  | WDR59 |  |
|  | ZNRF1 |  |
|  | LDHD |  |
|  | ZFP1 |  |
|  | CTRB2 |  |
|  | CTRB1 |  |
|  | BCAR1 |  |
|  | TMEM170A |  |
|  | CHST6 |  |
|  | CHST5 |  |
|  | TMEM231 |  |
|  | GABARAPL2 |  |
|  | ADAT1 |  |
|  | CNTNAP4 |  |
|  | MON1B |  |
|  | SYCE1L |  |
|  | ADAMTS18 |  |
|  | NUDT7 |  |
|  | VAT1L |  |
|  | CLEC3A |  |
|  | WWOX |  |
|  | MAF |  |
|  | CDYL2 |  |
|  | CENPN |  |
|  | ATMIN |  |
|  | C16orf46 |  |
|  | GCSH |  |
|  | PKD1L2 |  |
|  | BCO1 |  |

|  | GAN |  |
| --- | --- | --- |
|  | CMIP |  |
|  | PLCG2 |  |
|  | SDR42E1 |  |
|  | HSD17B2 |  |
|  | CDH13 |  |
|  | HSBP1 |  |
|  | MLYCD |  |
|  | OSGIN1 |  |
|  | NECAB2 |  |
|  | SLC38A8 |  |
|  | HSDL1 |  |
|  | TAF1C |  |
|  | KCNG4 |  |
|  | WFDC1 |  |
|  | TLDC1 |  |
|  | COTL1 |  |
|  | KLHL36 |  |
|  | USP10 |  |
|  | CRISPLD2 |  |
|  | ZDHHC7 |  |
|  | KIAA0513 |  |
|  | TAGLN2 |  |
|  | IGSF9 |  |
|  | SLAMF9 |  |
|  | PIGM |  |
|  | KCNJ10 |  |
|  | KCNJ9 |  |
|  | IGSF8 |  |
|  | ATP1A2 |  |
|  | ATP1A4 |  |
|  | CASQ1 |  |
|  | PEA15 |  |
|  | DCAF8 |  |
|  | PEX19 |  |
|  | COPA |  |
|  | NCSTN |  |
|  | NHLH1 |  |
|  | VANGL2 |  |
|  | SLAMF6 |  |
|  | CD84 |  |
|  | SLAMF1 |  |
|  | SLAMF7 |  |
|  | LY9 |  |
|  | CD244 |  |
|  | ITLN1 |  |
|  | ITLN2 |  |
|  | F11R |  |
|  | ARHGAP30 |  |
|  | NECTIN4 |  |
|  | KLHDC9 |  |
|  | NIT1 |  |
|  | DEDD |  |

|  | UFC1 |  |
| --- | --- | --- |
|  | USP21 |  |
|  | PPOX |  |
|  | B4GALT3 |  |
|  | ADAMTS4 |  |
|  | NDUFS2 |  |
|  | FCER1G |  |
|  | TOMM40L |  |
|  | NR1I3 |  |
|  | SDHC |  |
|  | CFAP126 |  |
|  | HSPA6 |  |
|  | FCGR3A |  |
|  | FCGR3B |  |
|  | FCGR2B |  |
|  | FCRLA |  |
|  | FCRLB |  |
|  | ATF6 |  |
|  | OLFML2B |  |
|  | NOS1AP |  |
|  | SPATA46 |  |
|  | C1orf226 |  |
|  | SH2D1B |  |
|  | UHMK1 |  |
|  | UAP1 |  |
|  | DDR2 |  |
|  | HSD17B7 |  |
|  | CCDC190 |  |
|  | RGS4 |  |
|  | RGS5 |  |
|  | PBX1 |  |
|  | LMX1A |  |
|  | RXRG |  |
|  | MGST3 |  |
|  | ALDH9A1 |  |
|  | TMCO1 |  |
|  | UCK2 |  |
|  | FAM78B |  |
|  | POGK |  |
|  | ILDR2 |  |
|  | MAEL |  |
|  | GPA33 |  |
|  | DUSP27 |  |
|  | POU2F1 |  |
|  | CD247 |  |
|  | CREG1 |  |
|  | RCSD1 |  |
|  | MPZL1 |  |
|  | ADCY10 |  |
|  | MPC2 |  |
|  | GPR161 |  |
|  | TBX19 |  |
|  | XCL2 |  |

|  | XCL1 |  |
| --- | --- | --- |
|  | DPT |  |
|  | NME7 |  |
|  | BLZF1 |  |
|  | CCDC181 |  |
|  | SLC19A2 |  |
|  | F5 |  |
|  | SELP |  |
|  | SELL |  |
|  | SELE |  |
|  | METTL18 |  |
|  | C1orf112 |  |
|  | SCYL3 |  |
|  | KIFAP3 |  |
|  | METTL11B |  |
|  | GORAB |  |
|  | PRRX1 |  |
|  | MROH9 |  |
|  | FMO3 |  |
|  | FMO2 |  |
|  | FMO4 |  |
|  | PRRC2C |  |
|  | MYOC |  |
|  | DNM3 |  |
|  | C1orf105 |  |
|  | PIGC |  |
|  | SUCO |  |
|  | FASLG |  |
|  | TNFSF4 |  |
|  | PRDX6 |  |
|  | SLC9C2 |  |
|  | ANKRD45 |  |
|  | KLHL20 |  |
|  | CENPL |  |
|  | DARS2 |  |
|  | ZBTB37 |  |
|  | SERPINC1 |  |
|  | RC3H1 |  |
|  | RABGAP1L |  |
|  | GPR52 |  |
|  | CACYBP |  |
|  | TNN |  |
|  | KIAA0040 |  |
|  | TNR |  |
|  | COP1 |  |
|  | PAPPA2 |  |
|  | ASTN1 |  |
|  | BRINP2 |  |
|  | SEC16B |  |
|  | RASAL2 |  |
|  | TEX35 |  |
|  | RALGPS2 |  |
|  | FAM20B |  |

|  | ABL2 |  |
| --- | --- | --- |
|  | SOAT1 |  |
|  | AXDND1 |  |
|  | NPHS2 |  |
|  | TDRD5 |  |
|  | TOR1AIP2 |  |
|  | TOR1AIP1 |  |
|  | CEP350 |  |
|  | QSOX1 |  |
|  | LHX4 |  |
|  | ACBD6 |  |
|  | XPR1 |  |
|  | KIAA1614 |  |
|  | STX6 |  |
|  | MR1 |  |
|  | IER5 |  |
|  | CACNA1E |  |
|  | ZNF648 |  |
|  | GLUL |  |
|  | TEDDM1 |  |
|  | RGSL1 |  |
|  | RNASEL |  |
|  | RGS16 |  |
|  | ZNRF3 |  |
|  | KREMEN1 |  |
|  | EMID1 |  |
|  | RHBDD3 |  |
|  | EWSR1 |  |
|  | GAS2L1 |  |
|  | RASL10A |  |
|  | THOC5 |  |
|  | NIPSNAP1 |  |
|  | NF2 |  |
|  | CABP7 |  |
|  | ZMAT5 |  |
|  | UQCR10 |  |
|  | ASCC2 |  |
|  | MTMR3 |  |
|  | HORMAD2 |  |
|  | LIF |  |
|  | OSM |  |
|  | CASTOR1 |  |
|  | TBC1D10A |  |
|  | SF3A1 |  |
|  | CCDC157 |  |
|  | RNF215 |  |
|  | SEC14L2 |  |
|  | MTFP1 |  |
|  | SEC14L3 |  |
|  | SEC14L4 |  |
|  | SEC14L6 |  |
|  | GAL3ST1 |  |
|  | PES1 |  |

|  | TCN2 |  |
| --- | --- | --- |
|  | SLC35E4 |  |
|  | DUSP18 |  |
|  | OSBP2 |  |
|  | MORC2 |  |
|  | SMTN |  |
|  | INPP5J |  |
|  | PLA2G3 |  |
|  | RNF185 |  |
|  | LIMK2 |  |
|  | PIK3IP1 |  |
|  | PATZ1 |  |
|  | EIF4ENIF1 |  |
|  | SFI1 |  |
|  | PISD |  |
|  | PRR14L |  |
|  | DEPDC5 |  |
|  | YWHAH |  |
|  | SLC5A1 |  |
|  | C22orf42 |  |
|  | RFPL2 |  |
|  | SLC5A4 |  |
|  | RTCB |  |
|  | BPIFC |  |
|  | FBXO7 |  |
|  | SYN3 |  |
|  | TIMP3 |  |
|  | LARGE1 |  |
|  | ISX |  |
|  | HMGXB4 |  |
|  | TOM1 |  |
|  | MCM5 |  |
|  | RASD2 |  |
|  | MB |  |
|  | APOL6 |  |
|  | RBFOX2 |  |
|  | APOL4 |  |
|  | APOL2 |  |
|  | APOL1 |  |
|  | MYH9 |  |
|  | TXN2 |  |
|  | FOXRED2 |  |
|  | EIF3D |  |
|  | CACNG2 |  |
|  | IFT27 |  |
|  | NCF4 |  |
|  | CSF2RB |  |
|  | TST |  |
|  | MPST |  |
|  | KCTD17 |  |
|  | TMPRSS6 |  |
|  | IL2RB |  |
|  | C1QTNF6 |  |

|  | SSTR3 |  |
| --- | --- | --- |
|  | RAC2 |  |
|  | CYTH4 |  |
|  | ELFN2 |  |
|  | MFNG |  |
|  | CDC42EP1 |  |
|  | LGALS2 |  |
|  | GGA1 |  |
|  | SH3BP1 |  |
|  | PDXP |  |
|  | LGALS1 |  |
|  | NOL12 |  |
|  | TRIOBP |  |
|  | H1F0 |  |
|  | GCAT |  |
|  | GALR3 |  |
|  | ANKRD54 |  |
|  | MICALL1 |  |
|  | C22orf23 |  |
|  | POLR2F |  |
|  | SOX10 |  |
|  | PICK1 |  |
|  | SLC16A8 |  |
|  | BAIAP2L2 |  |
|  | PLA2G6 |  |
|  | TMEM184B |  |
|  | LOC400927-CSNK1E |  |
|  | CSNK1E |  |
|  | KCNJ4 |  |
|  | KDELR3 |  |
|  | DDX17 |  |
|  | DMC1 |  |
|  | FAM227A |  |
|  | JOSD1 |  |
|  | GTPBP1 |  |
|  | SUN2 |  |
|  | DNAL4 |  |
|  | NPTXR |  |
|  | CBX6 |  |
|  | APOBEC3A |  |
|  | APOBEC3B |  |
|  | APOBEC3C |  |
|  | APOBEC3D |  |
|  | APOBEC3F |  |
|  | APOBEC3H |  |
|  | CBX7 |  |
|  | PDGFB |  |
|  | RPL3 |  |
|  | SYNGR1 |  |
|  | TAB1 |  |
|  | MGAT3 |  |
|  | MIEF1 |  |
|  | ATF4 |  |

|  | RPS19BP1 |  |
| --- | --- | --- |
|  | CACNA1I |  |
|  | ENTHD1 |  |
|  | GRAP2 |  |
|  | FAM83F |  |
|  | TNRC6B |  |
|  | ADSL |  |
|  | SGSM3 |  |
|  | MKL1 |  |
|  | MCHR1 |  |
|  | SLC25A17 |  |
|  | ST13 |  |
|  | XPNPEP3 |  |
|  | DNAJB7 |  |
|  | EP300 |  |
|  | L3MBTL2 |  |
|  | CHADL |  |
|  | RANGAP1 |  |
|  | ZC3H7B |  |
|  | TEF |  |
|  | TOB2 |  |
|  | ACO2 |  |
|  | POLR3H |  |
|  | CSDC2 |  |
|  | PMM1 |  |
|  | DESI1 |  |
|  | XRCC6 |  |
|  | MEI1 |  |
|  | SREBF2 |  |
|  | SHISA8 |  |
|  | TNFRSF13C |  |
|  | CENPM |  |
|  | 3-Sep |  |
|  | WBP2NL |  |
|  | NAGA |  |
|  | SMDT1 |  |
|  | CYP2D6 |  |
|  | CYP2D7 |  |
|  | TCF20 |  |
|  | NFAM1 |  |
|  | RRP7A |  |
|  | SERHL2 |  |
|  | POLDIP3 |  |
|  | CYB5R3 |  |
|  | A4GALT |  |
|  | ARFGAP3 |  |
|  | PACSIN2 |  |
|  | TTLL1 |  |
|  | BIK |  |
|  | MCAT |  |
|  | TSPO |  |
|  | TTLL12 |  |
|  | SCUBE1 |  |

|  | MPPED1 |  |
| --- | --- | --- |
|  | EFCAB6 |  |
|  | SULT4A1 |  |
|  | PNPLA3 |  |
|  | SAMM50 |  |
|  | PARVB |  |
|  | PARVG |  |
|  | SHISAL1 |  |
|  | RTL6 |  |
|  | PRR5 |  |
|  | PRR5-ARHGAP8 |  |
|  | ARHGAP8 |  |
|  | PHF21B |  |
|  | NUP50 |  |
|  | KIAA0930 |  |
|  | DEFB132 |  |
|  | C20orf96 |  |
|  | ZCCHC3 |  |
|  | SOX12 |  |
|  | NRSN2 |  |
|  | RBCK1 |  |
|  | TBC1D20 |  |
|  | CSNK2A1 |  |
|  | TCF15 |  |
|  | SRXN1 |  |
|  | SCRT2 |  |
|  | SLC52A3 |  |
|  | FAM110A |  |
|  | ANGPT4 |  |
|  | RSPO4 |  |
|  | PSMF1 |  |
|  | TMEM74B |  |
|  | C20orf202 |  |
|  | RAD21L1 |  |
|  | SNPH |  |
|  | SDCBP2 |  |
|  | FKBP1A |  |
|  | NSFL1C |  |
|  | SIRPB2 |  |
|  | SIRPB1 |  |
|  | SIRPG |  |
|  | SIRPA |  |
|  | PDYN |  |
|  | STK35 |  |
|  | TGM3 |  |
|  | TGM6 |  |
|  | SNRPB |  |
|  | ZNF343 |  |
|  | TMC2 |  |
|  | NOP56 |  |
|  | IDH3B |  |
|  | EBF4 |  |
|  | C20orf141 |  |

|  | TMEM239 |  |
| --- | --- | --- |
|  | PCED1A |  |
|  | VPS16 |  |
|  | PTPRA |  |
|  | GNRH2 |  |
|  | UBOX5 |  |
|  | FASTKD5 |  |
|  | LZTS3 |  |
|  | ITPA |  |
|  | SLC4A11 |  |
|  | C20orf194 |  |
|  | ATRN |  |
|  | GFRA4 |  |
|  | ADAM33 |  |
|  | SIGLEC1 |  |
|  | HSPA12B |  |
|  | C20orf27 |  |
|  | CENPB |  |
|  | CDC25B |  |
|  | AP5S1 |  |
|  | MAVS |  |
|  | PANK2 |  |
|  | RNF24 |  |
|  | SMOX |  |
|  | PRNP |  |
|  | PRND |  |
|  | RASSF2 |  |
|  | SLC23A2 |  |
|  | PCNA |  |
|  | CDS2 |  |
|  | PROKR2 |  |
|  | GPCPD1 |  |
|  | C20orf196 |  |
|  | CHGB |  |
|  | CRLS1 |  |
|  | LRRN4 |  |
|  | FERMT1 |  |
|  | BMP2 |  |
|  | TMX4 |  |
|  | PLCB1 |  |
|  | PLCB4 |  |
|  | LAMP5 |  |
|  | PAK5 |  |
|  | ANKEF1 |  |
|  | MKKS |  |
|  | SLX4IP |  |
|  | BTBD3 |  |
|  | TASP1 |  |
|  | ESF1 |  |
|  | SEL1L2 |  |
|  | MACROD2 |  |
|  | KIF16B |  |
|  | PCSK2 |  |

|  | BFSP1 |  |
| --- | --- | --- |
|  | DSTN |  |
|  | SNX5 |  |
|  | MGME1 |  |
|  | OVOL2 |  |
|  | KAT14 |  |
|  | ZNF133 |  |
|  | DZANK1 |  |
|  | POLR3F |  |
|  | RBBP9 |  |
|  | SEC23B |  |
|  | DTD1 |  |
|  | SLC24A3 |  |
|  | RIN2 |  |
|  | NAA20 |  |
|  | CFAP61 |  |
|  | INSM1 |  |
|  | RALGAPA2 |  |
|  | KIZ |  |
|  | XRN2 |  |
|  | NKX2-2 |  |
|  | PAX1 |  |
|  | FOXA2 |  |
|  | THBD |  |
|  | CD93 |  |
|  | NXT1 |  |
|  | GZF1 |  |
|  | CST8 |  |
|  | CST9 |  |
|  | CST3 |  |
|  | CST4 |  |
|  | CST7 |  |
|  | ACSS1 |  |
|  | VSX1 |  |
|  | ENTPD6 |  |
|  | PYGB |  |
|  | ABHD12 |  |
|  | GINS1 |  |
|  | NINL |  |
|  | NANP |  |
|  | ZNF337 |  |
|  | DEFB119 |  |
|  | HM13 |  |
|  | ID1 |  |
|  | COX4I2 |  |
|  | BCL2L1 |  |
|  | TPX2 |  |
|  | MYLK2 |  |
|  | FOXS1 |  |
|  | DUSP15 |  |
|  | TTLL9 |  |
|  | XKR7 |  |
|  | HCK |  |

|  | TM9SF4 |  |
| --- | --- | --- |
|  | PLAGL2 |  |
|  | KIF3B |  |
|  | ASXL1 |  |
|  | NOL4L |  |
|  | C20orf203 |  |
|  | COMMD7 |  |
|  | DNMT3B |  |
|  | MAPRE1 |  |
|  | EFCAB8 |  |
|  | SUN5 |  |
|  | BPIFB2 |  |
|  | BPIFB3 |  |
|  | BPIFB4 |  |
|  | BPIFA3 |  |
|  | BPIFA1 |  |
|  | BPIFB1 |  |
|  | CDK5RAP1 |  |
|  | SNTA1 |  |
|  | CBFA2T2 |  |
|  | NECAB3 |  |
|  | C20orf144 |  |
|  | E2F1 |  |
|  | PXMP4 |  |
|  | ZNF341 |  |
|  | CHMP4B |  |
|  | RALY |  |
|  | EIF2S2 |  |
|  | ASIP |  |
|  | ITCH |  |
|  | DYNLRB1 |  |
|  | MAP1LC3A |  |
|  | PIGU |  |
|  | TP53INP2 |  |
|  | NCOA6 |  |
|  | ACSS2 |  |
|  | TRPC3 |  |
|  | KIAA1109 |  |
|  | ADAD1 |  |
|  | BBS12 |  |
|  | FGF2 |  |
|  | NUDT6 |  |
|  | SPATA5 |  |
|  | SPRY1 |  |
|  | ANKRD50 |  |
|  | FAT4 |  |
|  | INTU |  |
|  | PLK4 |  |
|  | MFSD8 |  |
|  | ABHD18 |  |
|  | LARP1B |  |
|  | PGRMC2 |  |
|  | JADE1 |  |

|  | SCLT1 |  |
| --- | --- | --- |
|  | C4orf33 |  |
|  | PABPC4L |  |
|  | PCDH18 |  |
|  | SLC7A11 |  |
|  | ELF2 |  |
|  | NDUFC1 |  |
|  | NAA15 |  |
|  | RAB33B |  |
|  | SETD7 |  |
|  | MGST2 |  |
|  | MAML3 |  |
|  | SCOC |  |
|  | MGAT4D |  |
|  | TBC1D9 |  |
|  | RNF150 |  |
|  | ZNF330 |  |
|  | INPP4B |  |
|  | USP38 |  |
|  | GAB1 |  |
|  | SMARCA5 |  |
|  | FREM3 |  |
|  | GYPE |  |
|  | GYPA |  |
|  | HHIP |  |
|  | ANAPC10 |  |
|  | ABCE1 |  |
|  | OTUD4 |  |
|  | SMAD1 |  |
|  | MMAA |  |
|  | C4orf51 |  |
|  | ZNF827 |  |
|  | SLC10A7 |  |
|  | EDNRA |  |
|  | TMEM184C |  |
|  | PRMT9 |  |
|  | ARHGAP10 |  |
|  | NR3C2 |  |
|  | DCLK2 |  |
|  | LRBA |  |
|  | MAB21L2 |  |
|  | SH3D19 |  |
|  | PRSS48 |  |
|  | FAM160A1 |  |
|  | GATB |  |
|  | FBXW7 |  |
|  | TMEM154 |  |
|  | TIGD4 |  |
|  | ARFIP1 |  |
|  | FHDC1 |  |
|  | TRIM2 |  |
|  | MND1 |  |
|  | TMEM131L |  |

|  | TLR2 |  |
| --- | --- | --- |
|  | SFRP2 |  |
|  | DCHS2 |  |
|  | PLRG1 |  |
|  | FGB |  |
|  | FGA |  |
|  | FGG |  |
|  | LRAT |  |
|  | RBM46 |  |
|  | NPY2R |  |
|  | MAP9 |  |
|  | GUCY1A1 |  |
|  | GUCY1B1 |  |
|  | TDO2 |  |
|  | CTSO |  |
|  | GLRB |  |
|  | GRIA2 |  |
|  | FAM198B |  |
|  | RXFP1 |  |
|  | C4orf46 |  |
|  | PPID |  |
|  | FNIP2 |  |
|  | C4orf45 |  |
|  | RAPGEF2 |  |
|  | FSTL5 |  |
|  | NAF1 |  |
|  | NPY5R |  |
|  | SMIM31 |  |
|  | APELA |  |
|  | TRIM61 |  |
|  | TMEM192 |  |
|  | MSMO1 |  |
|  | TLL1 |  |
|  | SPOCK3 |  |
|  | ANXA10 |  |
|  | DDX60 |  |
|  | DDX60L |  |
|  | PALLD |  |
|  | SH3RF1 |  |
|  | CLCN3 |  |
|  | HPF1 |  |
|  | AADAT |  |
|  | GALNTL6 |  |
|  | GALNT7 |  |
|  | SCRG1 |  |
|  | FBXO8 |  |
|  | CEP44 |  |
|  | HPGD |  |
|  | GLRA3 |  |
|  | GPM6A |  |
|  | WDR17 |  |
|  | ASB5 |  |
|  | SPCS3 |  |

|  | TENM3 |  |
| --- | --- | --- |
|  | WWC2 |  |
|  | CLDN22 |  |
|  | CLDN24 |  |
|  | PYGM |  |
|  | SF1 |  |
|  | MAP4K2 |  |
|  | MEN1 |  |
|  | CDC42BPG |  |
|  | EHD1 |  |
|  | ATG2A |  |
|  | PPP2R5B |  |
|  | MAJIN |  |
|  | BATF2 |  |
|  | ARL2 |  |
|  | SAC3D1 |  |
|  | NAALADL1 |  |
|  | CDCA5 |  |
|  | ZFPL1 |  |
|  | VPS51 |  |
|  | TM7SF2 |  |
|  | FAU |  |
|  | SYVN1 |  |
|  | SPDYC |  |
|  | CAPN1 |  |
|  | POLA2 |  |
|  | DPF2 |  |
|  | TIGD3 |  |
|  | SLC25A45 |  |
|  | FRMD8 |  |
|  | SCYL1 |  |
|  | LTBP3 |  |
|  | SSSCA1 |  |
|  | FAM89B |  |
|  | EHBP1L1 |  |
|  | KCNK7 |  |
|  | PCNX3 |  |
|  | SIPA1 |  |
|  | RELA |  |
|  | KAT5 |  |
|  | RNASEH2C |  |
|  | AP5B1 |  |
|  | OVOL1 |  |
|  | CFL1 |  |
|  | MUS81 |  |
|  | EFEMP2 |  |
|  | CTSW |  |
|  | FIBP |  |
|  | CCDC85B |  |
|  | FOSL1 |  |
|  | C11orf68 |  |
|  | TSGA10IP |  |
|  | EIF1AD |  |

|  | BANF1 |  |
| --- | --- | --- |
|  | CST6 |  |
|  | CATSPER1 |  |
|  | GAL3ST3 |  |
|  | SF3B2 |  |
|  | PACS1 |  |
|  | KLC2 |  |
|  | RAB1B |  |
|  | CNIH2 |  |
|  | YIF1A |  |
|  | TMEM151A |  |
|  | CD248 |  |
|  | RIN1 |  |
|  | BRMS1 |  |
|  | SLC29A2 |  |
|  | NPAS4 |  |
|  | MRPL11 |  |
|  | PELI3 |  |
|  | DPP3 |  |
|  | BBS1 |  |
|  | ZDHHC24 |  |
|  | ACTN3 |  |
|  | CTSF |  |
|  | RBM14-RBM4 |  |
|  | RBM14 |  |
|  | RBM4 |  |
|  | RBM4B |  |
|  | SPTBN2 |  |
|  | C11orf80 |  |
|  | RCE1 |  |
|  | PC |  |
|  | LRFN4 |  |
|  | C11orf86 |  |
|  | SYT12 |  |
|  | RHOD |  |
|  | KDM2A |  |
|  | GRK2 |  |
|  | ANKRD13D |  |
|  | SSH3 |  |
|  | POLD4 |  |
|  | CLCF1 |  |
|  | RAD9A |  |
|  | CARNS1 |  |
|  | RPS6KB2 |  |
|  | PTPRCAP |  |
|  | CORO1B |  |
|  | GPR152 |  |
|  | CABP4 |  |
|  | TMEM134 |  |
|  | CDK2AP2 |  |
|  | CABP2 |  |
|  | NDUFV1 |  |
|  | NUDT8 |  |

|  | TBX10 |  |
| --- | --- | --- |
|  | ACY3 |  |
|  | CENPBD1 |  |
|  | DBNDD1 |  |
|  | LLGL2 |  |
|  | MYO15B |  |
|  | RECQL5 |  |
|  | SMIM5 |  |
|  | SAP30BP |  |
|  | ITGB4 |  |
|  | GALK1 |  |
|  | UNC13D |  |
|  | WBP2 |  |
|  | TRIM65 |  |
|  | MRPL38 |  |
|  | FBF1 |  |
|  | ACOX1 |  |
|  | TEN1 |  |
|  | EVPL |  |
|  | SRP68 |  |
|  | ZACN |  |
|  | EXOC7 |  |
|  | FOXJ1 |  |
|  | RNF157 |  |
|  | PRPSAP1 |  |
|  | SPHK1 |  |
|  | UBE2O |  |
|  | AANAT |  |
|  | RHBDF2 |  |
|  | CYGB |  |
|  | PRCD |  |
|  | ST6GALNAC2 |  |
|  | ST6GALNAC1 |  |
|  | MXRA7 |  |
|  | JMJD6 |  |
|  | METTL23 |  |
|  | MFSD11 |  |
|  | MGAT5B |  |
|  | SEC14L1 |  |
|  | 9-Sep |  |
|  | TNRC6C |  |
|  | TMC6 |  |
|  | TMC8 |  |
|  | C17orf99 |  |
|  | SYNGR2 |  |
|  | TK1 |  |
|  | AFMID |  |
|  | BIRC5 |  |
|  | TMEM235 |  |
|  | SOCS3 |  |
|  | PGS1 |  |
|  | DNAH17 |  |
|  | CYTH1 |  |

|  | USP36 |  |
| --- | --- | --- |
|  | TIMP2 |  |
|  | CEP295NL |  |
|  | LGALS3BP |  |
|  | CANT1 |  |
|  | C1QTNF1 |  |
|  | ENGASE |  |
|  | RBFOX3 |  |
|  | ENPP7 |  |
|  | CBX2 |  |
|  | CBX8 |  |
|  | TBC1D16 |  |
|  | CCDC40 |  |
|  | GAA |  |
|  | EIF4A3 |  |
|  | CARD14 |  |
|  | SGSH |  |
|  | SLC26A11 |  |
|  | RNF213 |  |
|  | ENDOV |  |
|  | NPTX1 |  |
|  | RPTOR |  |
|  | CHMP6 |  |
|  | BAIAP2 |  |
|  | AATK |  |
|  | PVALEF |  |
|  | CEP131 |  |
|  | TEPSIN |  |
|  | SLC38A10 |  |
|  | BAHCC1 |  |
|  | ACTG1 |  |
|  | FAAP100 |  |
|  | NPLOC4 |  |
|  | TSPAN10 |  |
|  | PDE6G |  |
|  | OXLD1 |  |
|  | ARL16 |  |
|  | MRPL12 |  |
|  | SLC25A10 |  |
|  | MCRIP1 |  |
|  | PPP1R27 |  |
|  | P4HB |  |
|  | ARHGDIA |  |
|  | ANAPC11 |  |
|  | NPB |  |
|  | SIRT7 |  |
|  | MAFG |  |
|  | PYCR1 |  |
|  | NOTUM |  |
|  | ASPSCR1 |  |
|  | CENPX |  |
|  | LRRC45 |  |
|  | RAC3 |  |

|  | DCXR |  |
| --- | --- | --- |
|  | RFNG |  |
|  | GPS1 |  |
|  | FASN |  |
|  | CCDC57 |  |
|  | SLC16A3 |  |
|  | CD7 |  |
|  | SECTM1 |  |
|  | TEX19 |  |
|  | OGFOD3 |  |
|  | HEXDC |  |
|  | C17orf62 |  |
|  | NARF |  |
|  | FOXK2 |  |
|  | FN3KRP |  |
|  | FN3K |  |
|  | TBCD |  |
|  | ZNF750 |  |
|  | B3GNTL1 |  |
|  | METRNL |  |
|  | TUBB8P12 |  |
|  | USP14 |  |
|  | COLEC12 |  |
|  | CLUL1 |  |
|  | TYMS |  |
|  | ENOSF1 |  |
|  | YES1 |  |
|  | ADCYAP1 |  |
|  | SMCHD1 |  |
|  | EMILIN2 |  |
|  | LPIN2 |  |
|  | MYOM1 |  |
|  | MYL12A |  |
|  | MYL12B |  |
|  | TGIF1 |  |
|  | DLGAP1 |  |
|  | AKAIN1 |  |
|  | ZBTB14 |  |
|  | EPB41L3 |  |
|  | TMEM200C |  |
|  | L3MBTL4 |  |
|  | ARHGAP28 |  |
|  | LAMA1 |  |
|  | LRRC30 |  |
|  | PTPRM |  |
|  | MTCL1 |  |
|  | ANKRD12 |  |
|  | RALBP1 |  |
|  | PPP4R1 |  |
|  | RAB31 |  |
|  | TXNDC2 |  |
|  | VAPA |  |
|  | NAPG |  |

|  | PIEZO2 |  |
| --- | --- | --- |
|  | EPM2A |  |
|  | SHPRH |  |
|  | GRM1 |  |
|  | ADGB |  |
|  | STXBP5 |  |
|  | SASH1 |  |
|  | UST |  |
|  | TAB2 |  |
|  | ZC3H12D |  |
|  | PPIL4 |  |
|  | GINM1 |  |
|  | KATNA1 |  |
|  | LATS1 |  |
|  | NUP43 |  |
|  | LRP11 |  |
|  | RAET1E |  |
|  | RAET1G |  |
|  | ULBP1 |  |
|  | ULBP3 |  |
|  | PPP1R14C |  |
|  | IYD |  |
|  | PLEKHG1 |  |
|  | MTHFD1L |  |
|  | AKAP12 |  |
|  | ZBTB2 |  |
|  | RMND1 |  |
|  | ARMT1 |  |
|  | ESR1 |  |
|  | SYNE1 |  |
|  | MYCT1 |  |
|  | VIP |  |
|  | FBXO5 |  |
|  | MTRF1L |  |
|  | RGS17 |  |
|  | OPRM1 |  |
|  | IPCEF1 |  |
|  | SCAF8 |  |
|  | TIAM2 |  |
|  | TFB1M |  |
|  | CLDN20 |  |
|  | ARID1B |  |
|  | TMEM242 |  |
|  | ZDHHC14 |  |
|  | SNX9 |  |
|  | SYNJ2 |  |
|  | SERAC1 |  |
|  | GTF2H5 |  |
|  | TULP4 |  |
|  | TMEM181 |  |
|  | DYNLT1 |  |
|  | SYTL3 |  |
|  | EZR |  |

|  | RSPH3 |  |
| --- | --- | --- |
|  | TAGAP |  |
|  | FNDC1 |  |
|  | SOD2 |  |
|  | WTAP |  |
|  | TCP1 |  |
|  | PNLDC1 |  |
|  | MAS1 |  |
|  | SLC22A1 |  |
|  | LPA |  |
|  | PLG |  |
|  | MAP3K4 |  |
|  | AGPAT4 |  |
|  | PRKN |  |
|  | PACRG |  |
|  | QKI |  |
|  | C6orf118 |  |
|  | PDE10A |  |
|  | TBXT |  |
|  | RPS6KA2 |  |
|  | RNASET2 |  |
|  | FGFR1OP |  |
|  | GPR31 |  |
|  | UNC93A |  |
|  | TTLL2 |  |
|  | AFDN |  |
|  | KIF25 |  |
|  | FRMD1 |  |
|  | DACT2 |  |
|  | SMOC2 |  |
|  | THBS2 |  |
|  | WDR27 |  |
|  | DLL1 |  |
|  | FAM120B |  |
|  | TBP |  |
|  | PDGFA |  |
|  | PRKAR1B |  |
|  | DNAAF5 |  |
|  | SUN1 |  |
|  | ADAP1 |  |
|  | COX19 |  |
|  | CYP2W1 |  |
|  | GPER1 |  |
|  | MICALL2 |  |
|  | INTS1 |  |
|  | MAFK |  |
|  | TMEM184A |  |
|  | PSMG3 |  |
|  | ELFN1 |  |
|  | MAD1L1 |  |
|  | MRM2 |  |
|  | NUDT1 |  |
|  | EIF3B |  |

|  | CHST12 |  |
| --- | --- | --- |
|  | BRAT1 |  |
|  | IQCE |  |
|  | TTYH3 |  |
|  | AMZ1 |  |
|  | GNA12 |  |
|  | CARD11 |  |
|  | SDK1 |  |
|  | AP5Z1 |  |
|  | RADIL |  |
|  | MMD2 |  |
|  | RBAK-RBAKDN |  |
|  | RBAK |  |
|  | WIPI2 |  |
|  | SLC29A4 |  |
|  | TNRC18 |  |
|  | FBXL18 |  |
|  | ACTB |  |
|  | FSCN1 |  |
|  | RNF216 |  |
|  | CCZ1 |  |
|  | RSPH10B |  |
|  | PMS2 |  |
|  | AIMP2 |  |
|  | EIF2AK1 |  |
|  | ANKRD61 |  |
|  | CYTH3 |  |
|  | FAM220A |  |
|  | RAC1 |  |
|  | DAGLB |  |
|  | KDELR2 |  |
|  | GRID2IP |  |
|  | C7orf26 |  |
|  | ZNF853 |  |
|  | ZNF316 |  |
|  | RSPH10B2 |  |
|  | CCZ1B |  |
|  | COL28A1 |  |
|  | RPA3 |  |
|  | UMAD1 |  |
|  | GLCCI1 |  |
|  | ICA1 |  |
|  | NDUFA4 |  |
|  | PHF14 |  |
|  | THSD7A |  |
|  | TMEM106B |  |
|  | VWDE |  |
|  | SCIN |  |
|  | ETV1 |  |
|  | FANCD2OS |  |
|  | BRK1 |  |
|  | VHL |  |
|  | IRAK2 |  |

|  | TATDN2 |  |
| --- | --- | --- |
|  | ATP2B2 |  |
|  | SLC6A11 |  |
|  | SLC6A1 |  |
|  | HRH1 |  |
|  | VGLL4 |  |
|  | TAMM41 |  |
|  | TIMP4 |  |
|  | PPARG |  |
|  | TSEN2 |  |
|  | MKRN2OS |  |
|  | MKRN2 |  |
|  | RAF1 |  |
|  | TMEM40 |  |
|  | CAND2 |  |
|  | RPL32 |  |
|  | IQSEC1 |  |
|  | HDAC11 |  |
|  | FBLN2 |  |
|  | WNT7A |  |
|  | CHCHD4 |  |
|  | TMEM43 |  |
|  | LSM3 |  |
|  | SLC6A6 |  |
|  | GRIP2 |  |
|  | CCDC174 |  |
|  | C3orf20 |  |
|  | FGD5 |  |
|  | NR2C2 |  |
|  | RBSN |  |
|  | CAPN7 |  |
|  | SH3BP5 |  |
|  | METTL6 |  |
|  | EAF1 |  |
|  | COLQ |  |
|  | HACL1 |  |
|  | BTD |  |
|  | ANKRD28 |  |
|  | GALNT15 |  |
|  | DPH3 |  |
|  | OXNAD1 |  |
|  | RFTN1 |  |
|  | DAZL |  |
|  | TBC1D5 |  |
|  | KCNH8 |  |
|  | EFHB |  |
|  | RAB5A |  |
|  | SGO1 |  |
|  | ZNF385D |  |
|  | UBE2E2 |  |
|  | UBE2E1 |  |
|  | NKIRAS1 |  |
|  | RPL15 |  |

|  | NR1D2 |  |
| --- | --- | --- |
|  | THRB |  |
|  | RARB |  |
|  | TOP2B |  |
|  | NGLY1 |  |
|  | LRRC3B |  |
|  | NEK10 |  |
|  | SLC4A7 |  |
|  | EOMES |  |
|  | CMC1 |  |
|  | AZI2 |  |
|  | ZCWPW2 |  |
|  | RBMS3 |  |
|  | TGFBR2 |  |
|  | STT3B |  |
|  | OSBPL10 |  |
|  | ZNF860 |  |
|  | CMTM7 |  |
|  | DYNC1LI1 |  |
|  | CNOT10 |  |
|  | TRIM71 |  |
|  | CCR4 |  |
|  | GLB1 |  |
|  | CRTAP |  |
|  | SUSD5 |  |
|  | FBXL2 |  |
|  | UBP1 |  |
|  | CLASP2 |  |
|  | PDCD6IP |  |
|  | ARPP21 |  |
|  | STAC |  |
|  | DCLK3 |  |
|  | TRANK1 |  |
|  | EPM2AIP1 |  |
|  | MLH1 |  |
|  | LRRFIP2 |  |
|  | GOLGA4 |  |
|  | ITGA9 |  |
|  | CTDSPL |  |
|  | VILL |  |
|  | PLCD1 |  |
|  | DLEC1 |  |
|  | MYD88 |  |
|  | OXSR1 |  |
|  | SLC22A13 |  |
|  | SLC22A14 |  |
|  | XYLB |  |
|  | ACVR2B |  |
|  | EXOG |  |
|  | SCN5A |  |
|  | SCN10A |  |
|  | SCN11A |  |
|  | WDR48 |  |

|  | GORASP1 |  |
| --- | --- | --- |
|  | TTC21A |  |
|  | CSRNP1 |  |
|  | XIRP1 |  |
|  | CX3CR1 |  |
|  | CCR8 |  |
|  | SLC25A38 |  |
|  | MOBP |  |
|  | MYRIP |  |
|  | EIF1B |  |
|  | ENTPD3 |  |
|  | RPL14 |  |
|  | ZNF619 |  |
|  | ZNF620 |  |
|  | ZNF621 |  |
|  | BUB1B |  |
|  | BUB1B-PAK6 |  |
|  | PAK6 |  |
|  | ANKRD63 |  |
|  | PLCB2 |  |
|  | CCDC9B |  |
|  | DISP2 |  |
|  | KNSTRN |  |
|  | IVD |  |
|  | BAHD1 |  |
|  | CHST14 |  |
|  | CCDC32 |  |
|  | RPUSD2 |  |
|  | KNL1 |  |
|  | RAD51 |  |
|  | RMDN3 |  |
|  | GCHFR |  |
|  | C15orf62 |  |
|  | ZFYVE19 |  |
|  | PPP1R14D |  |
|  | SPINT1 |  |
|  | RHOV |  |
|  | VPS18 |  |
|  | DLL4 |  |
|  | CHAC1 |  |
|  | INO80 |  |
|  | EXD1 |  |
|  | CHP1 |  |
|  | OIP5 |  |
|  | NUSAP1 |  |
|  | NDUFAF1 |  |
|  | RTF1 |  |
|  | ITPKA |  |
|  | LTK |  |
|  | RPAP1 |  |
|  | TYRO3 |  |
|  | MGA |  |
|  | MAPKBP1 |  |

|  | JMJD7-PLA2G4B |  |
| --- | --- | --- |
|  | JMJD7 |  |
|  | PLA2G4B |  |
|  | SPTBN5 |  |
|  | PLA2G4E |  |
|  | PLA2G4D |  |
|  | PLA2G4F |  |
|  | VPS39 |  |
|  | TMEM87A |  |
|  | GANC |  |
|  | CAPN3 |  |
|  | ZNF106 |  |
|  | SNAP23 |  |
|  | LRRC57 |  |
|  | HAUS2 |  |
|  | STARD9 |  |
|  | UBR1 |  |
|  | TMEM62 |  |
|  | CCNDBP1 |  |
|  | EPB42 |  |
|  | TGM5 |  |
|  | TGM7 |  |
|  | LCMT2 |  |
|  | ADAL |  |
|  | ZSCAN29 |  |
|  | TP53BP1 |  |
|  | MAP1A |  |
|  | PPIP5K1 |  |
|  | CKMT1B |  |
|  | STRC |  |
|  | CATSPER2 |  |
|  | CKMT1A |  |
|  | PDIA3 |  |
|  | ELL3 |  |
|  | SERF2 |  |
|  | SERINC4 |  |
|  | HYPK |  |
|  | WDR76 |  |
|  | FRMD5 |  |
|  | CASC4 |  |
|  | CTDSPL2 |  |
|  | EIF3J |  |
|  | SPG11 |  |
|  | PATL2 |  |
|  | B2M |  |
|  | TRIM69 |  |
|  | TERB2 |  |
|  | SORD |  |
|  | DUOX2 |  |
|  | DUOXA2 |  |
|  | DUOXA1 |  |
|  | DUOX1 |  |
|  | SHF |  |

|  | SLC28A2 |  |
| --- | --- | --- |
|  | SPATA5L1 |  |
|  | C15orf48 |  |
|  | SLC30A4 |  |
|  | BLOC1S6 |  |
|  | SQOR |  |
|  | SEMA6D |  |
|  | SLC24A5 |  |
|  | MYEF2 |  |
|  | CTXN2 |  |
|  | SLC12A1 |  |
|  | DUT |  |
|  | FBN1 |  |
|  | CEP152 |  |
|  | SHC4 |  |
|  | EID1 |  |
|  | SECISBP2L |  |
|  | GALK2 |  |
|  | FAM227B |  |
|  | FGF7 |  |
|  | DTWD1 |  |
|  | ATP8B4 |  |
|  | SLC27A2 |  |
|  | HDC |  |
|  | USP50 |  |
|  | TRPM7 |  |
|  | AP4E1 |  |
|  | CYP19A1 |  |
|  | SCG3 |  |
|  | TMOD2 |  |
|  | TMOD3 |  |
|  | GNB5 |  |
|  | MYO5C |  |
|  | MYO5A |  |
|  | ARPP19 |  |
|  | FAM214A |  |
|  | ONECUT1 |  |
|  | WDR72 |  |
|  | UNC13C |  |
|  | RAB27A |  |
|  | PIGBOS1 |  |
|  | DNAAF4 |  |
|  | PYGO1 |  |
|  | PRTG |  |
|  | NEDD4 |  |
|  | RFX7 |  |
|  | ZNF280D |  |
|  | TCF12 |  |
|  | CGNL1 |  |
|  | GCOM1 |  |
|  | MYZAP |  |
|  | POLR2M |  |
|  | ALDH1A2 |  |

|  | AQP9 |  |
| --- | --- | --- |
|  | LIPC |  |
|  | MINDY2 |  |
|  | SLTM |  |
|  | RNF111 |  |
|  | CCNB2 |  |
|  | MYO1E |  |
|  | FAM81A |  |
|  | GCNT3 |  |
|  | BNIP2 |  |
|  | FOXB1 |  |
|  | ANXA2 |  |
|  | ICE2 |  |
|  | RORA |  |
|  | VPS13C |  |
|  | C2CD4A |  |
|  | TLN2 |  |
|  | TPM1 |  |
|  | RPS27L |  |
|  | RAB8B |  |
|  | APH1B |  |
|  | CA12 |  |
|  | USP3 |  |
|  | FBXL22 |  |
|  | HERC1 |  |
|  | DAPK2 |  |
|  | BBX |  |
|  | CD47 |  |
|  | MYH15 |  |
|  | CIP2A |  |
|  | DZIP3 |  |
|  | RETNLB |  |
|  | TRAT1 |  |
|  | MORC1 |  |
|  | C3orf85 |  |
|  | DPPA2 |  |
|  | DPPA4 |  |
|  | NECTIN3 |  |
|  | CD96 |  |
|  | ZBED2 |  |
|  | PLCXD2 |  |
|  | PHLDB2 |  |
|  | ABHD10 |  |
|  | TAGLN3 |  |
|  | TMPRSS7 |  |
|  | C3orf52 |  |
|  | GCSAM |  |
|  | CD200 |  |
|  | BTLA |  |
|  | SLC35A5 |  |
|  | CCDC80 |  |
|  | CD200R1L |  |
|  | CD200R1 |  |

|  | GTPBP8 |  |
| --- | --- | --- |
|  | NEPRO |  |
|  | BOC |  |
|  | CFAP44 |  |
|  | SPICE1 |  |
|  | SIDT1 |  |
|  | USF3 |  |
|  | NAA50 |  |
|  | ATP6V1A |  |
|  | GRAMD1C |  |
|  | ZDHHC23 |  |
|  | CCDC191 |  |
|  | QTRT2 |  |
|  | DRD3 |  |
|  | ZNF80 |  |
|  | TIGIT |  |
|  | ZBTB20 |  |
|  | GAP43 |  |
|  | LSAMP |  |
|  | IGSF11 |  |
|  | C3orf30 |  |
|  | UPK1B |  |
|  | ARHGAP31 |  |
|  | TMEM39A |  |
|  | TIMMDC1 |  |
|  | CD80 |  |
|  | ADPRH |  |
|  | PLA1A |  |
|  | POPDC2 |  |
|  | COX17 |  |
|  | MAATS1 |  |
|  | NR1I2 |  |
|  | GSK3B |  |
|  | GPR156 |  |
|  | LRRC58 |  |
|  | FSTL1 |  |
|  | NDUFB4 |  |
|  | HGD |  |
|  | RABL3 |  |
|  | GTF2E1 |  |
|  | STXBP5L |  |
|  | POLQ |  |
|  | ARGFX |  |
|  | FBXO40 |  |
|  | HCLS1 |  |
|  | GOLGB1 |  |
|  | EAF2 |  |
|  | SLC15A2 |  |
|  | ILDR1 |  |
|  | CD86 |  |
|  | CASR |  |
|  | CSTA |  |
|  | CCDC58 |  |

|  | WDR5B |  |
| --- | --- | --- |
|  | KPNA1 |  |
|  | PARP9 |  |
|  | DTX3L |  |
|  | PARP15 |  |
|  | PARP14 |  |
|  | HSPBAP1 |  |
|  | SEMA5B |  |
|  | PDIA5 |  |
|  | SEC22A |  |
|  | ADCY5 |  |
|  | HACD2 |  |
|  | MYLK |  |
|  | CCDC14 |  |
|  | ROPN1 |  |
|  | KALRN |  |
|  | UMPS |  |
|  | MUC13 |  |
|  | HEG1 |  |
|  | SLC12A8 |  |
|  | ZNF148 |  |
|  | OSBPL11 |  |
|  | ALG1L |  |
|  | SLC41A3 |  |
|  | ALDH1L1 |  |
|  | KLF15 |  |
|  | CFAP100 |  |
|  | ZXDC |  |
|  | UROC1 |  |
|  | CHST13 |  |
|  | C3orf22 |  |
|  | TXNRD3 |  |
|  | CHCHD6 |  |
|  | PLXNA1 |  |
|  | TPRA1 |  |
|  | MCM2 |  |
|  | PODXL2 |  |
|  | ABTB1 |  |
|  | MGLL |  |
|  | KBTBD12 |  |
|  | SEC61A1 |  |
|  | RUVBL1 |  |
|  | EEFSEC |  |
|  | DNAJB8 |  |
|  | GATA2 |  |
|  | RAB7A |  |
|  | ACAD9 |  |
|  | KIAA1257 |  |
|  | EFCC1 |  |
|  | GP9 |  |
|  | ISY1-RAB43 |  |
|  | RAB43 |  |
|  | ISY1 |  |

|  | CNBP |  |
| --- | --- | --- |
|  | COPG1 |  |
|  | HMCES |  |
|  | EFCAB12 |  |
|  | MBD4 |  |
|  | IFT122 |  |
|  | RHO |  |
|  | PLXND1 |  |
|  | TMCC1 |  |
|  | TRH |  |
|  | ALG1L2 |  |
|  | COL6A5 |  |
|  | COL6A6 |  |
|  | PIK3R4 |  |
|  | ATP2C1 |  |
|  | ASTE1 |  |
|  | NEK11 |  |
|  | NUDT16 |  |
|  | MRPL3 |  |
|  | CPNE4 |  |
|  | ACPP |  |
|  | DNAJC13 |  |
|  | ACAD11 |  |
|  | ACKR4 |  |
|  | UBA5 |  |
|  | NPHP3 |  |
|  | TMEM108 |  |
|  | SHANK2 |  |
|  | DHCR7 |  |
|  | NADSYN1 |  |
|  | KRTAP5-8 |  |
|  | KRTAP5-9 |  |
|  | KRTAP5-10 |  |
|  | KRTAP5-11 |  |
|  | FAM86C1 |  |
|  | ZNF705E |  |
|  | RNF121 |  |
|  | IL18BP |  |
|  | NUMA1 |  |
|  | LRTOMT |  |
|  | LAMTOR1 |  |
|  | ANAPC15 |  |
|  | FOLR3 |  |
|  | FOLR1 |  |
|  | FOLR2 |  |
|  | INPPL1 |  |
|  | PHOX2A |  |
|  | CLPB |  |
|  | NLRC4 |  |
|  | BIRC6 |  |
|  | TTC27 |  |
|  | LTBP1 |  |
|  | RASGRP3 |  |

|  | FAM98A |  |
| --- | --- | --- |
|  | CRIM1 |  |
|  | FEZ2 |  |
|  | VIT |  |
|  | STRN |  |
|  | HEATR5B |  |
|  | GPATCH11 |  |
|  | EIF2AK2 |  |
|  | CEBPZOS |  |
|  | CEBPZ |  |
|  | PRKD3 |  |
|  | QPCT |  |
|  | CDC42EP3 |  |
|  | RMDN2 |  |
|  | CYP1B1 |  |
|  | ATL2 |  |
|  | HNRNPLL |  |
|  | GALM |  |
|  | SRSF7 |  |
|  | GEMIN6 |  |
|  | DHX57 |  |
|  | ARHGEF33 |  |
|  | SOS1 |  |
|  | CDKL4 |  |
|  | TMEM178A |  |
|  | THUMPD2 |  |
|  | SLC8A1 |  |
|  | PKDCC |  |
|  | EML4 |  |
|  | COX7A2L |  |
|  | MTA3 |  |
|  | HAAO |  |
|  | THADA |  |
|  | PLEKHH2 |  |
|  | C1GALT1C1L |  |
|  | ABCG5 |  |
|  | ABCG8 |  |
|  | LRPPRC |  |
|  | PPM1B |  |
|  | SLC3A1 |  |
|  | PREPL |  |
|  | CAMKMT |  |
|  | SIX3 |  |
|  | SIX2 |  |
|  | PRKCE |  |
|  | EPAS1 |  |
|  | RHOQ |  |
|  | PIGF |  |
|  | CRIPT |  |
|  | SOCS5 |  |
|  | MCFD2 |  |
|  | TTC7A |  |
|  | STPG4 |  |

|  | EPCAM |  |
| --- | --- | --- |
|  | MSH2 |  |
|  | FBXO11 |  |
|  | FOXN2 |  |
|  | PPP1R21 |  |
|  | STON1-GTF2A1L |  |
|  | STON1 |  |
|  | LHCGR |  |
|  | NRXN1 |  |
|  | ASB3 |  |
|  | CHAC2 |  |
|  | GPR75 |  |
|  | PSME4 |  |
|  | ACYP2 |  |
|  | TSPYL6 |  |
|  | SPTBN1 |  |
|  | RTN4 |  |
|  | CLHC1 |  |
|  | MTIF2 |  |
|  | CCDC88A |  |
|  | CFAP36 |  |
|  | PPP4R3B |  |
|  | PNPT1 |  |
|  | EFEMP1 |  |
|  | CCDC85A |  |
|  | FANCL |  |
|  | BCL11A |  |
|  | PAPOLG |  |
|  | REL |  |
|  | PUS10 |  |
|  | PEX13 |  |
|  | KIAA1841 |  |
|  | USP34 |  |
|  | XPO1 |  |
|  | FAM161A |  |
|  | CCT4 |  |
|  | COMMD1 |  |
|  | B3GNT2 |  |
|  | TMEM17 |  |
|  | EHBP1 |  |
|  | OTX1 |  |
|  | WDPCP |  |
|  | MDH1 |  |
|  | VPS54 |  |
|  | PELI1 |  |
|  | LGALSL |  |
|  | SERTAD2 |  |
|  | SLC1A4 |  |
|  | CEP68 |  |
|  | RAB1A |  |
|  | SPRED2 |  |
|  | MEIS1 |  |
|  | WDR92 |  |

|  | PNO1 |  |
| --- | --- | --- |
|  | PPP3R1 |  |
|  | CNRIP1 |  |
|  | PLEK |  |
|  | FBXO48 |  |
|  | PROKR1 |  |
|  | ARHGAP25 |  |
|  | BMP10 |  |
|  | ANTXR1 |  |
|  | GFPT1 |  |
|  | AAK1 |  |
|  | ANXA4 |  |
|  | GMCL1 |  |
|  | SNRNP27 |  |
|  | MXD1 |  |
|  | ASPRV1 |  |
|  | PCBP1 |  |
|  | C2orf42 |  |
|  | TIA1 |  |
|  | PCYOX1 |  |
|  | SNRPG |  |
|  | FAM136A |  |
|  | TGFA |  |
|  | ADD2 |  |
|  | CLEC4F |  |
|  | CD207 |  |
|  | VAX2 |  |
|  | ATP6V1B1 |  |
|  | ANKRD53 |  |
|  | TEX261 |  |
|  | NAGK |  |
|  | MPHOSPH10 |  |
|  | PAIP2B |  |
|  | DYSF |  |
|  | CYP26B1 |  |
|  | EXOC6B |  |
|  | POPDC3 |  |
|  | PREP |  |
|  | PRDM1 |  |
|  | ATG5 |  |
|  | CRYBG1 |  |
|  | RTN4IP1 |  |
|  | QRSL1 |  |
|  | CD24 |  |
|  | C6orf203 |  |
|  | BEND3 |  |
|  | PDSS2 |  |
|  | SCML4 |  |
|  | OSTM1 |  |
|  | NR2E1 |  |
|  | AFG1L |  |
|  | FOXO3 |  |
|  | ARMC2 |  |

|  | SESN1 |  |
| --- | --- | --- |
|  | CEP57L1 |  |
|  | CD164 |  |
|  | PPIL6 |  |
|  | MICAL1 |  |
|  | ZBTB24 |  |
|  | AK9 |  |
|  | FIG4 |  |
|  | GPR6 |  |
|  | WASF1 |  |
|  | CDC40 |  |
|  | METTL24 |  |
|  | SLC22A16 |  |
|  | CDK19 |  |
|  | RPF2 |  |
|  | SLC16A10 |  |
|  | MFSD4B |  |
|  | REV3L |  |
|  | TRAF3IP2 |  |
|  | FYN |  |
|  | WISP3 |  |
|  | FAM229B |  |
|  | LAMA4 |  |
|  | RFPL4B |  |
|  | HDAC2 |  |
|  | HS3ST5 |  |
|  | NT5DC1 |  |
|  | COL10A1 |  |
|  | TSPYL1 |  |
|  | DSE |  |
|  | TRAPPC3L |  |
|  | CALHM5 |  |
|  | CALHM4 |  |
|  | RWDD1 |  |
|  | ZUFSP |  |
|  | FAM162B |  |
|  | GPRC6A |  |
|  | VGLL2 |  |
|  | ROS1 |  |
|  | DCBLD1 |  |
|  | GOPC |  |
|  | NUS1 |  |
|  | SLC35F1 |  |
|  | CEP85L |  |
|  | MCM9 |  |
|  | FAM184A |  |
|  | MAN1A1 |  |
|  | TBC1D32 |  |
|  | HSF2 |  |
|  | FABP7 |  |
|  | SMPDL3A |  |
|  | CLVS2 |  |
|  | TRDN |  |

|  | RNF217 |  |
| --- | --- | --- |
|  | TPD52L1 |  |
|  | HEY2 |  |
|  | NCOA7 |  |
|  | HINT3 |  |
|  | CENPW |  |
|  | RSPO3 |  |
|  | SOGA3 |  |
|  | THEMIS |  |
|  | PTPRK |  |
|  | LAMA2 |  |
|  | L3MBTL3 |  |
|  | SAMD3 |  |
|  | TMEM200A |  |
|  | SMLR1 |  |
|  | EPB41L2 |  |
|  | AKAP7 |  |
|  | MED23 |  |
|  | ENPP3 |  |
|  | OR2A4 |  |
|  | CTAGE9 |  |
|  | ENPP1 |  |
|  | CTGF |  |
|  | MOXD1 |  |
|  | STX7 |  |
|  | TAAR9 |  |
|  | TAAR2 |  |
|  | TAAR1 |  |
|  | VNN1 |  |
|  | SLC18B1 |  |
|  | EYA4 |  |
|  | TCF21 |  |
|  | SLC2A12 |  |
|  | SGK1 |  |
|  | HBS1L |  |
|  | MYB |  |
|  | AHI1 |  |
|  | PDE7B |  |
|  | MTFR2 |  |
|  | BCLAF1 |  |
|  | MAP7 |  |
|  | MAP3K5 |  |
|  | SLC35D3 |  |
|  | IL22RA2 |  |
|  | IFNGR1 |  |
|  | OLIG3 |  |
|  | TNFAIP3 |  |
|  | PERP |  |
|  | ARFGEF3 |  |
|  | PBOV1 |  |
|  | HEBP2 |  |
|  | NHSL1 |  |
|  | CCDC28A |  |

|  | ECT2L |  |
| --- | --- | --- |
|  | REPS1 |  |
|  | HECA |  |
|  | CITED2 |  |
|  | VTA1 |  |
|  | ADGRG6 |  |
|  | HIVEP2 |  |
|  | AIG1 |  |
|  | ADAT2 |  |
|  | PEX3 |  |
|  | PHACTR2 |  |
|  | ZC2HC1B |  |
|  | PLAGL1 |  |
|  | SF3B5 |  |
|  | UTRN |  |
|  | SLC35G4 |  |
|  | GNAL |  |
|  | MPPE1 |  |
|  | IMPA2 |  |
|  | ANKRD62 |  |
|  | TUBB6 |  |
|  | AFG3L2 |  |
|  | PRELID3A |  |
|  | SPIRE1 |  |
|  | CEP76 |  |
|  | PSMG2 |  |
|  | PTPN2 |  |
|  | CEP192 |  |
|  | LDLRAD4 |  |
|  | FAM210A |  |
|  | RNMT |  |
|  | MC5R |  |
|  | MC2R |  |
|  | ZNF519 |  |
|  | POTEC |  |
|  | ROCK1 |  |
|  | GREB1L |  |
|  | ESCO1 |  |
|  | ABHD3 |  |
|  | GATA6 |  |
|  | CTAGE1 |  |
|  | CABLES1 |  |
|  | TMEM241 |  |
|  | RIOK3 |  |
|  | RMC1 |  |
|  | LAMA3 |  |
|  | TTC39C |  |
|  | CABYR |  |
|  | OSBPL1A |  |
|  | HRH4 |  |
|  | ZNF521 |  |
|  | SS18 |  |
|  | TAF4B |  |

|  | KCTD1 |  |
| --- | --- | --- |
|  | AQP4 |  |
|  | CHST9 |  |
|  | CDH2 |  |
|  | DSC3 |  |
|  | DSC2 |  |
|  | DSC1 |  |
|  | DSG1 |  |
|  | DSG4 |  |
|  | DSG3 |  |
|  | DSG2 |  |
|  | B4GALT6 |  |
|  | TRAPPC8 |  |
|  | RNF125 |  |
|  | RNF138 |  |
|  | GAREM1 |  |
|  | KLHL14 |  |
|  | CCDC178 |  |
|  | ASXL3 |  |
|  | NOL4 |  |
|  | DTNA |  |
|  | MAPRE2 |  |
|  | ZNF24 |  |
|  | ZNF396 |  |
|  | INO80C |  |
|  | C18orf21 |  |
|  | RPRD1A |  |
|  | SLC39A6 |  |
|  | ELP2 |  |
|  | MOCOS |  |
|  | FHOD3 |  |
|  | TPGS2 |  |
|  | KIAA1328 |  |
|  | CELF4 |  |
|  | PIK3C3 |  |
|  | RIT2 |  |
|  | SETBP1 |  |
|  | SLC14A2 |  |
|  | SLC14A1 |  |
|  | SIGLEC15 |  |
|  | EPG5 |  |
|  | PSTPIP2 |  |
|  | ATP5F1A |  |
|  | C18orf25 |  |
|  | RNF165 |  |
|  | LOXHD1 |  |
|  | ST8SIA5 |  |
|  | PIAS2 |  |
|  | KATNAL2 |  |
|  | ELOA3D |  |
|  | ELOA3B |  |
|  | ELOA3 |  |
|  | ELOA2 |  |

|  | IER3IP1 |  |
| --- | --- | --- |
|  | SKOR2 |  |
|  | SMAD2 |  |
|  | ZBTB7C |  |
|  | CTIF |  |
|  | SMAD7 |  |
|  | DYM |  |
|  | C18orf32 |  |
|  | LIPG |  |
|  | MYO5B |  |
|  | CFAP53 |  |
|  | MBD1 |  |
|  | CXXC1 |  |
|  | SKA1 |  |
|  | MAPK4 |  |
|  | MRO |  |
|  | ME2 |  |
|  | SMAD4 |  |
|  | DCC |  |
|  | MBD2 |  |
|  | POLI |  |
|  | STARD6 |  |
|  | C18orf54 |  |
|  | DYNAP |  |
|  | RAB27B |  |
|  | CCDC68 |  |
|  | TCF4 |  |
|  | TXNL1 |  |
|  | WDR7 |  |
|  | BOD1L2 |  |
|  | ONECUT2 |  |
|  | FECH |  |
|  | ATP8B1 |  |
|  | NEDD4L |  |
|  | ALPK2 |  |
|  | MALT1 |  |
|  | ZNF532 |  |
|  | RAX |  |
|  | CPLX4 |  |
|  | LMAN1 |  |
|  | CCBE1 |  |
|  | CDH20 |  |
|  | RNF152 |  |
|  | PIGN |  |
|  | KIAA1468 |  |
|  | TNFRSF11A |  |
|  | ZCCHC2 |  |
|  | PHLPP1 |  |
|  | BCL2 |  |
|  | KDSR |  |
|  | VPS4B |  |
|  | SERPINB5 |  |
|  | SERPINB12 |  |

|  | SERPINB13 |  |
| --- | --- | --- |
|  | SERPINB2 |  |
|  | SERPINB10 |  |
|  | SERPINB8 |  |
|  | CDH7 |  |
|  | IQCF2 |  |
|  | IQCF1 |  |
|  | RRP9 |  |
|  | PARP3 |  |
|  | PCBP4 |  |
|  | ABHD14B |  |
|  | ABHD14A-ACY1 |  |
|  | ABHD14A |  |
|  | ACY1 |  |
|  | DUSP7 |  |
|  | POC1A |  |
|  | ALAS1 |  |
|  | TLR9 |  |
|  | TWF2 |  |
|  | PPM1M |  |
|  | WDR82 |  |
|  | GLYCTK |  |
|  | DNAH1 |  |
|  | BAP1 |  |
|  | PHF7 |  |
|  | SEMA3G |  |
|  | TNNC1 |  |
|  | NISCH |  |
|  | STAB1 |  |
|  | NT5DC2 |  |
|  | PBRM1 |  |
|  | GNL3 |  |
|  | GLT8D1 |  |
|  | NEK4 |  |
|  | ITIH1 |  |
|  | ITIH3 |  |
|  | TMEM110-MUSTN1 |  |
|  | MUSTN1 |  |
|  | STIMATE |  |
|  | SFMBT1 |  |
|  | RFT1 |  |
|  | PRKCD |  |
|  | TKT |  |
|  | DCP1A |  |
|  | CACNA1D |  |
|  | IL17RB |  |
|  | ACTR8 |  |
|  | CACNA2D3 |  |
|  | LRTM1 |  |
|  | WNT5A |  |
|  | ERC2 |  |
|  | CCDC66 |  |
|  | FAM208A |  |

|  | ARHGEF3 |  |
| --- | --- | --- |
|  | SPATA12 |  |
|  | IL17RD |  |
|  | HESX1 |  |
|  | APPL1 |  |
|  | ASB14 |  |
|  | DNAH12 |  |
|  | PDE12 |  |
|  | DENND6A |  |
|  | SLMAP |  |
|  | FLNB |  |
|  | DNASE1L3 |  |
|  | ABHD6 |  |
|  | RPP14 |  |
|  | HTD2 |  |
|  | PXK |  |
|  | PDHB |  |
|  | KCTD6 |  |
|  | ACOX2 |  |
|  | FAM107A |  |
|  | C3orf67 |  |
|  | FHIT |  |
|  | PTPRG |  |
|  | FEZF2 |  |
|  | CADPS |  |
|  | SYNPR |  |
|  | ATXN7 |  |
|  | PRICKLE2 |  |
|  | ADAMTS9 |  |
|  | MAGI1 |  |
|  | SLC25A26 |  |
|  | LRIG1 |  |
|  | KBTBD8 |  |
|  | SUCLG2 |  |
|  | FAM19A1 |  |
|  | EOGT |  |
|  | TMF1 |  |
|  | ARL6IP5 |  |
|  | LMOD3 |  |
|  | FRMD4B |  |
|  | MITF |  |
|  | FOXP1 |  |
|  | PROK2 |  |
|  | RYBP |  |
|  | SHQ1 |  |
|  | PPP4R2 |  |
|  | EBLN2 |  |
|  | PDZRN3 |  |
|  | CNTN3 |  |
|  | FRG2C |  |
|  | ZNF717 |  |
|  | ROBO2 |  |
|  | ROBO1 |  |

|  | CADM2 |  |
| --- | --- | --- |
|  | VGLL3 |  |
|  | CHMP2B |  |
|  | POU1F1 |  |
|  | HTR1F |  |
|  | CGGBP1 |  |
|  | ZNF654 |  |
|  | EPHA3 |  |
|  | PROS1 |  |
|  | ARL13B |  |
|  | DHFR2 |  |
|  | NSUN3 |  |
|  | EPHA6 |  |
|  | ARL6 |  |
|  | CRYBG3 |  |
|  | RIOX2 |  |
|  | GABRR3 |  |
|  | OR5H14 |  |
|  | OR5H15 |  |
|  | OR5H6 |  |
|  | OR5K2 |  |
|  | CLDND1 |  |
|  | GPR15 |  |
|  | ST3GAL6 |  |
|  | DCBLD2 |  |
|  | FILIP1L |  |
|  | NIT2 |  |
|  | TOMM70 |  |
|  | LNP1 |  |
|  | TMEM45A |  |
|  | TFG |  |
|  | ABI3BP |  |
|  | IMPG2 |  |
|  | SENP7 |  |
|  | PCNP |  |
|  | NXPE3 |  |
|  | NFKBIZ |  |
|  | ALCAM |  |
|  | CBLB |  |
|  | SIGLEC14 |  |
|  | SPACA6 |  |
|  | FPR1 |  |
|  | FPR2 |  |
|  | FPR3 |  |
|  | ZNF577 |  |
|  | ZNF649 |  |
|  | ZNF613 |  |
|  | ZNF615 |  |
|  | ZNF432 |  |
|  | ZNF841 |  |
|  | ZNF616 |  |
|  | ZNF836 |  |
|  | PPP2R1A |  |

|  | ZNF766 |  |
| --- | --- | --- |
|  | ZNF610 |  |
|  | ZNF528 |  |
|  | ZNF534 |  |
|  | ZNF578 |  |
|  | ZNF808 |  |
|  | ZNF701 |  |
|  | ZNF83 |  |
|  | ZNF600 |  |
|  | ZNF28 |  |
|  | ZNF468 |  |
|  | ZNF320 |  |
|  | ZNF888 |  |
|  | ZNF816-ZNF321P |  |
|  | ERVV-1 |  |
|  | ERVV-2 |  |
|  | ZNF160 |  |
|  | ZNF415 |  |
|  | ZNF347 |  |
|  | ZNF665 |  |
|  | VN1R4 |  |
|  | ZNF845 |  |
|  | ZNF525 |  |
|  | ZNF765 |  |
|  | ZNF761 |  |
|  | ZNF813 |  |
|  | ZNF331 |  |
|  | DPRX |  |
|  | NLRP12 |  |
|  | MYADM |  |
|  | PRKCG |  |
|  | CACNG7 |  |
|  | CACNG8 |  |
|  | CACNG6 |  |
|  | VSTM1 |  |
|  | OSCAR |  |
|  | NDUFA3 |  |
|  | TFPT |  |
|  | PRPF31 |  |
|  | CNOT3 |  |
|  | LENG1 |  |
|  | TMC4 |  |
|  | MBOAT7 |  |
|  | TSEN34 |  |
|  | RPS9 |  |
|  | LILRB3 |  |
|  | LILRA6 |  |
|  | LILRB5 |  |
|  | LILRB2 |  |
|  | LILRA5 |  |
|  | LILRA4 |  |
|  | LAIR1 |  |
|  | TTYH1 |  |

|  | LENG8 |  |
| --- | --- | --- |
|  | LENG9 |  |
|  | CDC42EP5 |  |
|  | LILRA2 |  |
|  | LILRA1 |  |
|  | LILRB1 |  |
|  | LILRB4 |  |
|  | KIR3DL3 |  |
|  | KIR2DL3 |  |
|  | KIR2DL1 |  |
|  | KIR2DL4 |  |
|  | KIR3DL1 |  |
|  | KIR2DS4 |  |
|  | KIR3DL2 |  |
|  | FCAR |  |
|  | NCR1 |  |
|  | NLRP7 |  |
|  | NLRP2 |  |
|  | GP6 |  |
|  | RDH13 |  |
|  | EPS8L1 |  |
|  | PPP1R12C |  |
|  | TNNT1 |  |
|  | TNNI3 |  |
|  | DNAAF3 |  |
|  | SYT5 |  |
|  | PTPRH |  |
|  | TMEM86B |  |
|  | HSPBP1 |  |
|  | BRSK1 |  |
|  | TMEM150B |  |
|  | KMT5C |  |
|  | COX6B2 |  |
|  | IL11 |  |
|  | TMEM190 |  |
|  | TMEM238 |  |
|  | RPL28 |  |
|  | UBE2S |  |
|  | SHISA7 |  |
|  | ISOC2 |  |
|  | ZNF628 |  |
|  | NAT14 |  |
|  | SSC5D |  |
|  | ZNF579 |  |
|  | ZNF524 |  |
|  | ZNF865 |  |
|  | ZNF784 |  |
|  | ZNF580 |  |
|  | CCDC106 |  |
|  | U2AF2 |  |
|  | EPN1 |  |
|  | NLRP9 |  |
|  | RFPL4A |  |

|  | RFPL4AL1 |  |
| --- | --- | --- |
|  | NLRP11 |  |
|  | NLRP4 |  |
|  | NLRP13 |  |
|  | NLRP8 |  |
|  | NLRP5 |  |
|  | ZNF787 |  |
|  | ZNF444 |  |
|  | GALP |  |
|  | ZSCAN5B |  |
|  | ZSCAN5C |  |
|  | ZSCAN5A |  |
|  | EDDM13 |  |
|  | ZNF583 |  |
|  | ZNF667 |  |
|  | ZNF471 |  |
|  | ZNF470 |  |
|  | ZNF71 |  |
|  | ZNF835 |  |
|  | ZIM2 |  |
|  | PEG3 |  |
|  | USP29 |  |
|  | ZIM3 |  |
|  | DUXA |  |
|  | ZNF264 |  |
|  | AURKC |  |
|  | ZNF805 |  |
|  | ZNF543 |  |
|  | ZNF304 |  |
|  | ZNF547 |  |
|  | ZNF548 |  |
|  | ZNF17 |  |
|  | ZNF749 |  |
|  | VN1R1 |  |
|  | ZNF772 |  |
|  | ZNF419 |  |
|  | ZNF549 |  |
|  | ZNF550 |  |
|  | ZNF416 |  |
|  | ZIK1 |  |
|  | ZNF530 |  |
|  | ZNF134 |  |
|  | ZNF211 |  |
|  | ZSCAN4 |  |
|  | ZNF551 |  |
|  | ZNF154 |  |
|  | ZNF776 |  |
|  | ZNF586 |  |
|  | ZNF552 |  |
|  | ZNF587B |  |
|  | ZNF587 |  |
|  | ZNF814 |  |
|  | ZNF417 |  |

|  | ZNF418 |  |
| --- | --- | --- |
|  | ZNF256 |  |
|  | C19orf18 |  |
|  | ZNF606 |  |
|  | ZSCAN1 |  |
|  | ZNF135 |  |
|  | ZSCAN18 |  |
|  | ZNF329 |  |
|  | ZNF274 |  |
|  | ZNF544 |  |
|  | ZNF8 |  |
|  | ZSCAN22 |  |
|  | A1BG |  |
|  | ZNF497 |  |
|  | ZNF837 |  |
|  | RPS5 |  |
|  | RNF225 |  |
|  | ZNF584 |  |
|  | ZNF132 |  |
|  | ZNF324B |  |
|  | ZNF324 |  |
|  | ZNF446 |  |
|  | SLC27A5 |  |
|  | ZBTB45 |  |
|  | TRIM28 |  |
|  | UBE2M |  |
|  | MZF1 |  |
|  | DEFB126 |  |
|  | DEFB127 |  |
|  | ST6GALNAC4 |  |
|  | PIP5KL1 |  |
|  | FAM102A |  |
|  | NAIF1 |  |
|  | SLC25A25 |  |
|  | PTGES2 |  |
|  | LCN2 |  |
|  | C9orf16 |  |
|  | CIZ1 |  |
|  | DNM1 |  |
|  | GOLGA2 |  |
|  | SWI5 |  |
|  | TRUB2 |  |
|  | COQ4 |  |
|  | SLC27A4 |  |
|  | URM1 |  |
|  | CERCAM |  |
|  | ODF2 |  |
|  | GLE1 |  |
|  | WDR34 |  |
|  | SET |  |
|  | PKN3 |  |
|  | ZDHHC12 |  |
|  | ZER1 |  |

|  | TBC1D13 |  |
| --- | --- | --- |
|  | ENDOG |  |
|  | SPOUT1 |  |
|  | KYAT1 |  |
|  | PHYHD1 |  |
|  | NUP188 |  |
|  | SH3GLB2 |  |
|  | MIGA2 |  |
|  | DOLPP1 |  |
|  | CRAT |  |
|  | PTPA |  |
|  | ASB6 |  |
|  | PRRX2 |  |
|  | PTGES |  |
|  | TOR1B |  |
|  | TOR1A |  |
|  | C9orf78 |  |
|  | USP20 |  |
|  | FNBP1 |  |
|  | GPR107 |  |
|  | NCS1 |  |
|  | ASS1 |  |
|  | FUBP3 |  |
|  | EXOSC2 |  |
|  | ABL1 |  |
|  | QRFP |  |
|  | FIBCD1 |  |
|  | LAMC3 |  |
|  | AIF1L |  |
|  | NUP214 |  |
|  | FAM78A |  |
|  | PLPP7 |  |
|  | PRRC2B |  |
|  | UCK1 |  |
|  | RAPGEF1 |  |
|  | NTNG2 |  |
|  | SETX |  |
|  | TTF1 |  |
|  | CFAP77 |  |
|  | BARHL1 |  |
|  | DDX31 |  |
|  | GTF3C4 |  |
|  | AK8 |  |
|  | SPACA9 |  |
|  | TSC1 |  |
|  | GFI1B |  |
|  | GTF3C5 |  |
|  | CEL |  |
|  | RALGDS |  |
|  | GBGT1 |  |
|  | OBP2B |  |
|  | ABO |  |
|  | MED22 |  |

|  | SURF2 |  |
| --- | --- | --- |
|  | SURF4 |  |
|  | STKLD1 |  |
|  | REXO4 |  |
|  | ADAMTS13 |  |
|  | CACFD1 |  |
|  | SLC2A6 |  |
|  | MYMK |  |
|  | ADAMTSL2 |  |
|  | FAM163B |  |
|  | SARDH |  |
|  | VAV2 |  |
|  | BRD3OS |  |
|  | BRD3 |  |
|  | RXRA |  |
|  | COL5A1 |  |
|  | FCN1 |  |
|  | OLFM1 |  |
|  | PPP1R26 |  |
|  | MRPS2 |  |
|  | OBP2A |  |
|  | PAEP |  |
|  | GLT6D1 |  |
|  | SOHLH1 |  |
|  | KCNT1 |  |
|  | CAMSAP1 |  |
|  | NACC2 |  |
|  | TMEM250 |  |
|  | LHX3 |  |
|  | QSOX2 |  |
|  | CCDC187 |  |
|  | GPSM1 |  |
|  | CARD9 |  |
|  | SNAPC4 |  |
|  | SDCCAG3 |  |
|  | INPP5E |  |
|  | SEC16A |  |
|  | NOTCH1 |  |
|  | EGFL7 |  |
|  | FAM69B |  |
|  | LCN10 |  |
|  | LCN6 |  |
|  | LCN8 |  |
|  | TMEM141 |  |
|  | AJM1 |  |
|  | PHPT1 |  |
|  | MAMDC4 |  |
|  | EDF1 |  |
|  | TRAF2 |  |
|  | C8G |  |
|  | LCN12 |  |
|  | PTGDS |  |
|  | LCNL1 |  |

|  | CLIC3 |  |
| --- | --- | --- |
|  | ABCA2 |  |
|  | FUT7 |  |
|  | NPDC1 |  |
|  | ENTPD2 |  |
|  | UAP1L1 |  |
|  | MAN1B1 |  |
|  | GRIN1 |  |
|  | LRRC26 |  |
|  | SSNA1 |  |
|  | NDOR1 |  |
|  | RNF208 |  |
|  | CYSRT1 |  |
|  | RNF224 |  |
|  | SLC34A3 |  |
|  | TUBB4B |  |
|  | STPG3 |  |
|  | NELFB |  |
|  | EXD3 |  |
|  | NOXA1 |  |
|  | ENTPD8 |  |
|  | NSMF |  |
|  | PNPLA7 |  |
|  | DPH7 |  |
|  | ZMYND19 |  |
|  | ARRDC1 |  |
|  | SOWAHC |  |
|  | RGPD5 |  |
|  | LIMS3 |  |
|  | MALL |  |
|  | NPHP1 |  |
|  | SMIM37 |  |
|  | LIMS4 |  |
|  | BUB1 |  |
|  | ACOXL |  |
|  | BCL2L11 |  |
|  | ANAPC1 |  |
|  | MERTK |  |
|  | TMEM87B |  |
|  | FBLN7 |  |
|  | ZC3H6 |  |
|  | RGPD8 |  |
|  | TTL |  |
|  | POLR1B |  |
|  | CHCHD5 |  |
|  | SLC20A1 |  |
|  | NT5DC4 |  |
|  | CKAP2L |  |
|  | IL1A |  |
|  | IL37 |  |
|  | IL36A |  |
|  | IL36RN |  |
|  | IL1F10 |  |

|  | IL1RN |  |
| --- | --- | --- |
|  | PSD4 |  |
|  | PAX8 |  |
|  | FOXD4L1 |  |
|  | RABL2A |  |
|  | SLC35F5 |  |
|  | ACTR3 |  |
|  | DPP10 |  |
|  | DDX18 |  |
|  | CCDC93 |  |
|  | MARCO |  |
|  | STEAP3 |  |
|  | C2orf76 |  |
|  | DBI |  |
|  | TMEM37 |  |
|  | SCTR |  |
|  | CFAP221 |  |
|  | TMEM177 |  |
|  | PTPN4 |  |
|  | EPB41L5 |  |
|  | TMEM185B |  |
|  | RALB |  |
|  | INHBB |  |
|  | TFCP2L1 |  |
|  | CLASP1 |  |
|  | TSN |  |
|  | CNTNAP5 |  |
|  | GYPC |  |
|  | TEX51 |  |
|  | BIN1 |  |
|  | CYP27C1 |  |
|  | ERCC3 |  |
|  | MAP3K2 |  |
|  | PROC |  |
|  | IWS1 |  |
|  | MYO7B |  |
|  | LIMS2 |  |
|  | GPR17 |  |
|  | SFT2D3 |  |
|  | WDR33 |  |
|  | POLR2D |  |
|  | AMMECR1L |  |
|  | SAP130 |  |
|  | UGGT1 |  |
|  | HS6ST1 |  |
|  | RAB6C |  |
|  | CCDC74B |  |
|  | SMPD4 |  |
|  | MZT2B |  |
|  | TUBA3E |  |
|  | CCDC115 |  |
|  | IMP4 |  |
|  | PTPN18 |  |

|  | POTEI |  |
| --- | --- | --- |
|  | CFC1B |  |
|  | POTEJ |  |
|  | GPR148 |  |
|  | AMER3 |  |
|  | ARHGEF4 |  |
|  | FAM168B |  |
|  | PLEKHB2 |  |
|  | RAB6D |  |
|  | TUBA3D |  |
|  | CCDC74A |  |
|  | GPR39 |  |
|  | LYPD1 |  |
|  | NCKAP5 |  |
|  | MGAT5 |  |
|  | CCNT2 |  |
|  | MAP3K19 |  |
|  | RAB3GAP1 |  |
|  | ZRANB3 |  |
|  | R3HDM1 |  |
|  | UBXN4 |  |
|  | LCT |  |
|  | MCM6 |  |
|  | DARS |  |
|  | CXCR4 |  |
|  | THSD7B |  |
|  | HNMT |  |
|  | NXPH2 |  |
|  | KYNU |  |
|  | ARHGAP15 |  |
|  | GTDC1 |  |
|  | ZEB2 |  |
|  | ACVR2A |  |
|  | ORC4 |  |
|  | MBD5 |  |
|  | LYPD6B |  |
|  | LYPD6 |  |
|  | RND3 |  |
|  | RBM43 |  |
|  | RIF1 |  |
|  | NEB |  |
|  | CACNB4 |  |
|  | STAM2 |  |
|  | FMNL2 |  |
|  | PRPF40A |  |
|  | RPRM |  |
|  | GALNT13 |  |
|  | KCNJ3 |  |
|  | GPD2 |  |
|  | GALNT5 |  |
|  | ERMN |  |
|  | CYTIP |  |
|  | ACVR1C |  |

|  | ACVR1 |  |
| --- | --- | --- |
|  | UPP2 |  |
|  | PKP4 |  |
|  | DAPL1 |  |
|  | TANC1 |  |
|  | 7-Mar |  |
|  | LY75-CD302 |  |
|  | CD302 |  |
|  | LY75 |  |
|  | PLA2R1 |  |
|  | ITGB6 |  |
|  | RBMS1 |  |
|  | LY6G5C |  |
|  | ABHD16A |  |
|  | LY6G6F |  |
|  | LY6G6F-LY6G6D |  |
|  | LY6G6C |  |
|  | MPIG6B |  |
|  | DDAH2 |  |
|  | CLIC1 |  |
|  | MSH5 |  |
|  | VWA7 |  |
|  | VARS |  |
|  | HSPA1L |  |
|  | HSPA1A |  |
|  | HSPA1B |  |
|  | SLC44A4 |  |
|  | EHMT2 |  |
|  | C2 |  |
|  | CFB |  |
|  | NELFE |  |
|  | SKIV2L |  |
|  | DXO |  |
|  | STK19 |  |
|  | C4A |  |
|  | C4B |  |
|  | CYP21A2 |  |
|  | ATF6B |  |
|  | FKBPL |  |
|  | PRRT1 |  |
|  | PPT2 |  |
|  | RNF5 |  |
|  | PBX2 |  |
|  | GPSM3 |  |
|  | NOTCH4 |  |
|  | C6orf10 |  |
|  | BTNL2 |  |
|  | HLA-DRA |  |
|  | HLA-DRB5 |  |
|  | HLA-DRB1 |  |
|  | HLA-DQA1 |  |
|  | HLA-DQB1 |  |
|  | HLA-DQB2 |  |

|  | HLA-DOB |  |
| --- | --- | --- |
|  | TAP2 |  |
|  | PSMB8 |  |
|  | TAP1 |  |
|  | HLA-DMB |  |
|  | HLA-DMA |  |
|  | BRD2 |  |
|  | HLA-DOA |  |
|  | HLA-DPA1 |  |
|  | HLA-DPB1 |  |
|  | COL11A2 |  |
|  | RXRB |  |
|  | SLC39A7 |  |
|  | HSD17B8 |  |
|  | RING1 |  |
|  | VPS52 |  |
|  | RPS18 |  |
|  | B3GALT4 |  |
|  | WDR46 |  |
|  | PFDN6 |  |
|  | RGL2 |  |
|  | TAPBP |  |
|  | ZBTB22 |  |
|  | DAXX |  |
|  | PHF1 |  |
|  | CUTA |  |
|  | SYNGAP1 |  |
|  | ZBTB9 |  |
|  | BAK1 |  |
|  | ITPR3 |  |
|  | UQCC2 |  |
|  | IP6K3 |  |
|  | LEMD2 |  |
|  | MLN |  |
|  | GRM4 |  |
|  | HMGA1 |  |
|  | SMIM29 |  |
|  | NUDT3 |  |
|  | PACSIN1 |  |
|  | SPDEF |  |
|  | C6orf106 |  |
|  | SNRPC |  |
|  | UHRF1BP1 |  |
|  | TAF11 |  |
|  | ANKS1A |  |
|  | TCP11 |  |
|  | SCUBE3 |  |
|  | ZNF76 |  |
|  | DEF6 |  |
|  | PPARD |  |
|  | FANCE |  |
|  | TEAD3 |  |
|  | TULP1 |  |

|  | FKBP5 |  |
| --- | --- | --- |
|  | ARMC12 |  |
|  | CLPSL1 |  |
|  | CLPS |  |
|  | LHFPL5 |  |
|  | MAPK14 |  |
|  | MAPK13 |  |
|  | BRPF3 |  |
|  | PNPLA1 |  |
|  | C6orf222 |  |
|  | ETV7 |  |
|  | KCTD20 |  |
|  | STK38 |  |
|  | CDKN1A |  |
|  | RAB44 |  |
|  | CPNE5 |  |
|  | PPIL1 |  |
|  | C6orf89 |  |
|  | PI16 |  |
|  | MTCH1 |  |
|  | FGD2 |  |
|  | PIM1 |  |
|  | TMEM217 |  |
|  | TBC1D22B |  |
|  | RNF8 |  |
|  | CMTR1 |  |
|  | CCDC167 |  |
|  | MDGA1 |  |
|  | ZFAND3 |  |
|  | BTBD9 |  |
|  | GLO1 |  |
|  | DNAH8 |  |
|  | GLP1R |  |
|  | SAYSD1 |  |
|  | KCNK5 |  |
|  | KCNK17 |  |
|  | KCNK16 |  |
|  | KIF6 |  |
|  | DAAM2 |  |
|  | MOCS1 |  |
|  | LRFN2 |  |
|  | TSPO2 |  |
|  | NFYA |  |
|  | TREML1 |  |
|  | TREM2 |  |
|  | TREML2 |  |
|  | TREML4 |  |
|  | TREM1 |  |
|  | NCR2 |  |
|  | FOXP4 |  |
|  | MDFI |  |
|  | TFEB |  |
|  | PGC |  |

|  | FRS3 |  |
| --- | --- | --- |
|  | PRICKLE4 |  |
|  | USP49 |  |
|  | MED20 |  |
|  | BYSL |  |
|  | CCND3 |  |
|  | TAF8 |  |
|  | C6orf132 |  |
|  | GUCA1A |  |
|  | GUCA1B |  |
|  | MRPS10 |  |
|  | TRERF1 |  |
|  | UBR2 |  |
|  | PRPH2 |  |
|  | BICRAL |  |
|  | RPL7L1 |  |
|  | CNPY3 |  |
|  | PEX6 |  |
|  | PPP2R5D |  |
|  | MEA1 |  |
|  | KLHDC3 |  |
|  | CUL7 |  |
|  | MRPL2 |  |
|  | KLC4 |  |
|  | PTK7 |  |
|  | CUL9 |  |
|  | DNPH1 |  |
|  | MYH7B |  |
|  | TRPC4AP |  |
|  | EDEM2 |  |
|  | PROCR |  |
|  | MMP24 |  |
|  | MMP24OS |  |
|  | EIF6 |  |
|  | GDF5 |  |
|  | CEP250 |  |
|  | C20orf173 |  |
|  | ERGIC3 |  |
|  | SPAG4 |  |
|  | CPNE1 |  |
|  | RBM12 |  |
|  | NFS1 |  |
|  | PHF20 |  |
|  | SCAND1 |  |
|  | CNBD2 |  |
|  | EPB41L1 |  |
|  | AAR2 |  |
|  | DLGAP4 |  |
|  | MYL9 |  |
|  | TGIF2 |  |
|  | TGIF2-C20orf24 |  |
|  | C20orf24 |  |
|  | SLA2 |  |

|  | NDRG3 |  |
| --- | --- | --- |
|  | DSN1 |  |
|  | SOGA1 |  |
|  | TLDC2 |  |
|  | SAMHD1 |  |
|  | RBL1 |  |
|  | MROH8 |  |
|  | GHRH |  |
|  | MANBAL |  |
|  | SRC |  |
|  | BLCAP |  |
|  | NNAT |  |
|  | VSTM2L |  |
|  | TTI1 |  |
|  | RPRD1B |  |
|  | TGM2 |  |
|  | KIAA1755 |  |
|  | BPI |  |
|  | LBP |  |
|  | RALGAPB |  |
|  | ADIG |  |
|  | ARHGAP40 |  |
|  | SLC32A1 |  |
|  | ACTR5 |  |
|  | PPP1R16B |  |
|  | FAM83D |  |
|  | DHX35 |  |
|  | MAFB |  |
|  | TOP1 |  |
|  | PLCG1 |  |
|  | ZHX3 |  |
|  | LPIN3 |  |
|  | CHD6 |  |
|  | PTPRT |  |
|  | L3MBTL1 |  |
|  | SGK2 |  |
|  | IFT52 |  |
|  | MYBL2 |  |
|  | GTSF1L |  |
|  | TOX2 |  |
|  | JPH2 |  |
|  | OSER1 |  |
|  | GDAP1L1 |  |
|  | FITM2 |  |
|  | HNF4A |  |
|  | TTPAL |  |
|  | SERINC3 |  |
|  | PKIG |  |
|  | ADA |  |
|  | WISP2 |  |
|  | KCNK15 |  |
|  | RIMS4 |  |
|  | YWHAB |  |

|  | TOMM34 |  |
| --- | --- | --- |
|  | STK4 |  |
|  | KCNS1 |  |
|  | WFDC5 |  |
|  | WFDC12 |  |
|  | SEMG1 |  |
|  | SEMG2 |  |
|  | MATN4 |  |
|  | RBPJL |  |
|  | SYS1 |  |
|  | DBNDD2 |  |
|  | PIGT |  |
|  | WFDC6 |  |
|  | WFDC8 |  |
|  | WFDC9 |  |
|  | WFDC3 |  |
|  | DNTTIP1 |  |
|  | UBE2C |  |
|  | SNX21 |  |
|  | ACOT8 |  |
|  | ZSWIM1 |  |
|  | SPATA25 |  |
|  | NEURL2 |  |
|  | CTSA |  |
|  | PLTP |  |
|  | PCIF1 |  |
|  | ZNF335 |  |
|  | MMP9 |  |
|  | SLC12A5 |  |
|  | NCOA5 |  |
|  | CDH22 |  |
|  | SLC35C2 |  |
|  | ELMO2 |  |
|  | ZNF334 |  |
|  | OCSTAMP |  |
|  | SLC13A3 |  |
|  | TP53RK |  |
|  | SLC2A10 |  |
|  | EYA2 |  |
|  | ZMYND8 |  |
|  | NCOA3 |  |
|  | SULF2 |  |
|  | PREX1 |  |
|  | ARFGEF2 |  |
|  | CSE1L |  |
|  | STAU1 |  |
|  | ZNFX1 |  |
|  | KCNB1 |  |
|  | PTGIS |  |
|  | B4GALT5 |  |
|  | SLC9A8 |  |
|  | SPATA2 |  |
|  | RNF114 |  |

|  | SNAI1 |  |
| --- | --- | --- |
|  | TMEM189-UBE2V1 |  |
|  | UBE2V1 |  |
|  | TMEM189 |  |
|  | PTPN1 |  |
|  | RIPOR3 |  |
|  | PARD6B |  |
|  | BCAS4 |  |
|  | ADNP |  |
|  | DPM1 |  |
|  | MOCS3 |  |
|  | NFATC2 |  |
|  | ATP9A |  |
|  | SALL4 |  |
|  | ZFP64 |  |
|  | TSHZ2 |  |
|  | ZNF217 |  |
|  | BCAS1 |  |
|  | CYP24A1 |  |
|  | DOK5 |  |
|  | FAM210B |  |
|  | AURKA |  |
|  | CSTF1 |  |
|  | CASS4 |  |
|  | FAM209A |  |
|  | FAM209B |  |
|  | TFAP2C |  |
|  | BMP7 |  |
|  | SPO11 |  |
|  | RAE1 |  |
|  | MTRNR2L3 |  |
|  | RBM38 |  |
|  | CTCFL |  |
|  | PCK1 |  |
|  | ZBP1 |  |
|  | PMEPA1 |  |
|  | ANKRD60 |  |
|  | RAB22A |  |
|  | VAPB |  |
|  | APCDD1L |  |
|  | STX16 |  |
|  | NPEPL1 |  |
|  | DLG5 |  |
|  | RPS24 |  |
|  | ZMIZ1 |  |
|  | PPIF |  |
|  | ZCCHC24 |  |
|  | EIF5AL1 |  |
|  | SFTPA1 |  |
|  | NUTM2B |  |
|  | NUTM2E |  |
|  | TMEM254 |  |
|  | ANXA11 |  |

|  | DYDC1 |  |
| --- | --- | --- |
|  | DYDC2 |  |
|  | FAM213A |  |
|  | TSPAN14 |  |
|  | SH2D4B |  |
|  | NRG3 |  |
|  | C10orf99 |  |
|  | CDHR1 |  |
|  | LRIT2 |  |
|  | LRIT1 |  |
|  | RGR |  |
|  | CCSER2 |  |
|  | GRID1 |  |
|  | WAPL |  |
|  | OPN4 |  |
|  | LDB3 |  |
|  | BMPR1A |  |
|  | MMRN2 |  |
|  | SNCG |  |
|  | ADIRF |  |
|  | FAM35A |  |
|  | NUTM2A |  |
|  | NUTM2D |  |
|  | MINPP1 |  |
|  | PAPSS2 |  |
|  | ATAD1 |  |
|  | KLLN |  |
|  | PTEN |  |
|  | RNLS |  |
|  | LIPJ |  |
|  | LIPF |  |
|  | LIPK |  |
|  | ANKRD22 |  |
|  | STAMBPL1 |  |
|  | ACTA2 |  |
|  | FAS |  |
|  | LIPA |  |
|  | IFIT3 |  |
|  | IFIT1B |  |
|  | IFIT1 |  |
|  | IFIT5 |  |
|  | SLC16A12 |  |
|  | PANK1 |  |
|  | HTR7 |  |
|  | RPP30 |  |
|  | ANKRD1 |  |
|  | PCGF5 |  |
|  | HECTD2 |  |
|  | PPP1R3C |  |
|  | TNKS2 |  |
|  | FGFBP3 |  |
|  | CPEB3 |  |
|  | 5-Mar |  |

|  | IDE |  |
| --- | --- | --- |
|  | KIF11 |  |
|  | HHEX |  |
|  | EXOC6 |  |
|  | CYP26C1 |  |
|  | CYP26A1 |  |
|  | MYOF |  |
|  | FFAR4 |  |
|  | RBP4 |  |
|  | PDE6C |  |
|  | FRA10AC1 |  |
|  | LGI1 |  |
|  | PLCE1 |  |
|  | NOC3L |  |
|  | CYP2C18 |  |
|  | CYP2C19 |  |
|  | CYP2C9 |  |
|  | CYP2C8 |  |
|  | ACSM6 |  |
|  | PDLIM1 |  |
|  | SORBS1 |  |
|  | ALDH18A1 |  |
|  | TCTN3 |  |
|  | ENTPD1 |  |
|  | CC2D2B |  |
|  | CCNJ |  |
|  | ZNF518A |  |
|  | BLNK |  |
|  | DNTT |  |
|  | OPALIN |  |
|  | TLL2 |  |
|  | TM9SF3 |  |
|  | PIK3AP1 |  |
|  | LCOR |  |
|  | SLIT1 |  |
|  | ARHGAP19 |  |
|  | FRAT1 |  |
|  | FRAT2 |  |
|  | RRP12 |  |
|  | EXOSC1 |  |
|  | ZDHHC16 |  |
|  | MMS19 |  |
|  | UBTD1 |  |
|  | ANKRD2 |  |
|  | HOGA1 |  |
|  | MORN4 |  |
|  | PI4K2A |  |
|  | AVPI1 |  |
|  | MARVELD1 |  |
|  | ZFYVE27 |  |
|  | SFRP5 |  |
|  | GOLGA7B |  |
|  | CRTAC1 |  |

|  | R3HCC1L |  |
| --- | --- | --- |
|  | PYROXD2 |  |
|  | HPS1 |  |
|  | HPSE2 |  |
|  | CNNM1 |  |
|  | SLC25A28 |  |
|  | ENTPD7 |  |
|  | COX15 |  |
|  | ABCC2 |  |
|  | DNMBP |  |
|  | CPN1 |  |
|  | ERLIN1 |  |
|  | CHUK |  |
|  | CWF19L1 |  |
|  | BLOC1S2 |  |
|  | PKD2L1 |  |
|  | SCD |  |
|  | WNT8B |  |
|  | SEC31B |  |
|  | HIF1AN |  |
|  | PAX2 |  |
|  | SLF2 |  |
|  | SEMA4G |  |
|  | MRPL43 |  |
|  | TWNK |  |
|  | LZTS2 |  |
|  | PDZD7 |  |
|  | SFXN3 |  |
|  | KAZALD1 |  |
|  | TLX1 |  |
|  | LBX1 |  |
|  | BTRC |  |
|  | POLL |  |
|  | DPCD |  |
|  | FBXW4 |  |
|  | FGF8 |  |
|  | NPM3 |  |
|  | MGEA5 |  |
|  | KCNIP2 |  |
|  | ARMH3 |  |
|  | LDB1 |  |
|  | PPRC1 |  |
|  | TYW5 |  |
|  | MAIP1 |  |
|  | SPATS2L |  |
|  | KCTD18 |  |
|  | AOX1 |  |
|  | BZW1 |  |
|  | OR4C46 |  |
|  | TRIM48 |  |
|  | OR4A16 |  |
|  | OR4A15 |  |
|  | OR5D13 |  |

|  | OR5L1 |  |
| --- | --- | --- |
|  | OR5D18 |  |
|  | OR5L2 |  |
|  | OR5D16 |  |
|  | TRIM51 |  |
|  | OR5W2 |  |
|  | OR10AG1 |  |
|  | OR5AS1 |  |
|  | OR8I2 |  |
|  | OR8H3 |  |
|  | OR8J3 |  |
|  | OR8H1 |  |
|  | OR8U1 |  |
|  | OR5R1 |  |
|  | OR5M11 |  |
|  | OR9G1 |  |
|  | LRRC55 |  |
|  | APLNR |  |
|  | TNKS1BP1 |  |
|  | SSRP1 |  |
|  | P2RX3 |  |
|  | PRG2 |  |
|  | SLC43A1 |  |
|  | UBE2L6 |  |
|  | SERPING1 |  |
|  | YPEL4 |  |
|  | CLP1 |  |
|  | ZDHHC5 |  |
|  | TMX2 |  |
|  | SELENOH |  |
|  | BTBD18 |  |
|  | CTNND1 |  |
|  | OR9Q1 |  |
|  | OR6Q1 |  |
|  | OR9I1 |  |
|  | OR9Q2 |  |
|  | OR10Q1 |  |
|  | OR5B17 |  |
|  | OR5B3 |  |
|  | OR5B2 |  |
|  | OR5B12 |  |
|  | OR5B21 |  |
|  | LPXN |  |
|  | ZFP91 |  |
|  | GLYAT |  |
|  | GLYATL2 |  |
|  | GLYATL1 |  |
|  | GLYATL1B |  |
|  | FAM111A |  |
|  | DTX4 |  |
|  | OR5AN1 |  |
|  | OR5A1 |  |
|  | OR4D10 |  |

|  | OR4D11 |  |
| --- | --- | --- |
|  | OSBP |  |
|  | PATL1 |  |
|  | OR10V1 |  |
|  | STX3 |  |
|  | GIF |  |
|  | TCN1 |  |
|  | MS4A3 |  |
|  | MS4A2 |  |
|  | MS4A6A |  |
|  | MS4A4A |  |
|  | MS4A7 |  |
|  | MS4A14 |  |
|  | MS4A1 |  |
|  | MS4A18 |  |
|  | MS4A15 |  |
|  | MS4A10 |  |
|  | CCDC86 |  |
|  | PTGDR2 |  |
|  | ZP1 |  |
|  | PRPF19 |  |
|  | TMEM109 |  |
|  | SLC15A3 |  |
|  | CD6 |  |
|  | VPS37C |  |
|  | PGA3 |  |
|  | PGA4 |  |
|  | PGA5 |  |
|  | VWCE |  |
|  | DDB1 |  |
|  | TKFC |  |
|  | CYB561A3 |  |
|  | TMEM138 |  |
|  | CPSF7 |  |
|  | SDHAF2 |  |
|  | PPP1R32 |  |
|  | LRRC10B |  |
|  | SYT7 |  |
|  | DAGLA |  |
|  | MYRF |  |
|  | FADS1 |  |
|  | FADS2 |  |
|  | FADS3 |  |
|  | RAB3IL1 |  |
|  | BEST1 |  |
|  | FTH1 |  |
|  | INCENP |  |
|  | ASRGL1 |  |
|  | AHNAK |  |
|  | EEF1G |  |
|  | TUT1 |  |
|  | MTA2 |  |
|  | EML3 |  |

|  | ROM1 |  |
| --- | --- | --- |
|  | B3GAT3 |  |
|  | GANAB |  |
|  | INTS5 |  |
|  | LBHD1 |  |
|  | C11orf98 |  |
|  | CSKMT |  |
|  | UQCC3 |  |
|  | UBXN1 |  |
|  | BSCL2 |  |
|  | GNG3 |  |
|  | HNRNPUL2 |  |
|  | TTC9C |  |
|  | POLR2G |  |
|  | TAF6L |  |
|  | TMEM179B |  |
|  | TMEM223 |  |
|  | NXF1 |  |
|  | STX5 |  |
|  | WDR74 |  |
|  | SLC3A2 |  |
|  | CHRM1 |  |
|  | SLC22A6 |  |
|  | SLC22A8 |  |
|  | SLC22A24 |  |
|  | SLC22A25 |  |
|  | SLC22A10 |  |
|  | SLC22A9 |  |
|  | HRASLS5 |  |
|  | LGALS12 |  |
|  | RARRES3 |  |
|  | PLA2G16 |  |
|  | ATL3 |  |
|  | RTN3 |  |
|  | C11orf95 |  |
|  | SPINDOC |  |
|  | MARK2 |  |
|  | RCOR2 |  |
|  | NAA40 |  |
|  | OTUB1 |  |
|  | FLRT1 |  |
|  | FERMT3 |  |
|  | NUDT22 |  |
|  | DNAJC4 |  |
|  | VEGFB |  |
|  | PPP1R14B |  |
|  | PLCB3 |  |
|  | BAD |  |
|  | GPR137 |  |
|  | KCNK4 |  |
|  | CATSPERZ |  |
|  | ESRRA |  |
|  | TRMT112 |  |

|  | PRDX5 |  |
| --- | --- | --- |
|  | CCDC88B |  |
|  | SLC22A11 |  |
|  | SLC22A12 |  |
|  | NRXN2 |  |
|  | RASGRP2 |  |
|  | SKIL |  |
|  | CLDN11 |  |
|  | SLC7A14 |  |
|  | RPL22L1 |  |
|  | EIF5A2 |  |
|  | TNIK |  |
|  | PLD1 |  |
|  | FNDC3B |  |
|  | GHSR |  |
|  | TNFSF10 |  |
|  | NCEH1 |  |
|  | ECT2 |  |
|  | SPATA16 |  |
|  | NLGN1 |  |
|  | NAALADL2 |  |
|  | TBL1XR1 |  |
|  | KCNMB2 |  |
|  | ZMAT3 |  |
|  | PIK3CA |  |
|  | KCNMB3 |  |
|  | ZNF639 |  |
|  | MFN1 |  |
|  | GNB4 |  |
|  | ACTL6A |  |
|  | MRPL47 |  |
|  | USP13 |  |
|  | PEX5L |  |
|  | TTC14 |  |
|  | CCDC39 |  |
|  | FXR1 |  |
|  | DNAJC19 |  |
|  | SOX2 |  |
|  | ATP11B |  |
|  | DCUN1D1 |  |
|  | MCCC1 |  |
|  | LAMP3 |  |
|  | MCF2L2 |  |
|  | B3GNT5 |  |
|  | KLHL6 |  |
|  | KLHL24 |  |
|  | YEATS2 |  |
|  | PARL |  |
|  | ABCC5 |  |
|  | HTR3D |  |
|  | HTR3C |  |
|  | HTR3E |  |
|  | EIF2B5 |  |

|  | DVL3 |  |
| --- | --- | --- |
|  | AP2M1 |  |
|  | ABCF3 |  |
|  | VWA5B2 |  |
|  | ALG3 |  |
|  | EEF1AKMT4-ECE2 |  |
|  | ECE2 |  |
|  | EIF4G1 |  |
|  | FAM131A |  |
|  | CLCN2 |  |
|  | POLR2H |  |
|  | THPO |  |
|  | CHRD |  |
|  | VPS8 |  |
|  | C3orf70 |  |
|  | EHHADH |  |
|  | MAP3K13 |  |
|  | TMEM41A |  |
|  | LIPH |  |
|  | SENP2 |  |
|  | IGF2BP2 |  |
|  | TRA2B |  |
|  | ETV5 |  |
|  | DGKG |  |
|  | TBCCD1 |  |
|  | DNAJB11 |  |
|  | AHSG |  |
|  | HRG |  |
|  | KNG1 |  |
|  | RFC4 |  |
|  | ADIPOQ |  |
|  | ST6GAL1 |  |
|  | RPL39L |  |
|  | RTP1 |  |
|  | MASP1 |  |
|  | SST |  |
|  | RTP2 |  |
|  | BCL6 |  |
|  | LPP |  |
|  | TPRG1 |  |
|  | TP63 |  |
|  | P3H2 |  |
|  | CLDN1 |  |
|  | CLDN16 |  |
|  | TMEM207 |  |
|  | IL1RAP |  |
|  | GMNC |  |
|  | UTS2B |  |
|  | CCDC50 |  |
|  | FGF12 |  |
|  | HRASLS |  |
|  | ATP13A5 |  |
|  | ATP13A4 |  |

|  | OPA1 |  |
| --- | --- | --- |
|  | CPN2 |  |
|  | LRRC15 |  |
|  | GP5 |  |
|  | TMEM44 |  |
|  | LSG1 |  |
|  | FAM43A |  |
|  | XXYLT1 |  |
|  | ACAP2 |  |
|  | PPP1R2 |  |
|  | APOD |  |
|  | MUC20 |  |
|  | MUC4 |  |
|  | TNK2 |  |
|  | TFRC |  |
|  | ZDHHC19 |  |
|  | SLC51A |  |
|  | UBXN7 |  |
|  | RNF168 |  |
|  | WDR53 |  |
|  | FBXO45 |  |
|  | NRROS |  |
|  | CEP19 |  |
|  | PIGX |  |
|  | PAK2 |  |
|  | SENP5 |  |
|  | NCBP2 |  |
|  | PIGZ |  |
|  | MELTF |  |
|  | DLG1 |  |
|  | BDH1 |  |
|  | RUBCN |  |
|  | LRCH3 |  |
|  | IQCG |  |
|  | LMLN |  |
|  | ZNF595 |  |
|  | ZNF718 |  |
|  | ZNF732 |  |
|  | ZNF141 |  |
|  | PIGG |  |
|  | PDE6B |  |
|  | ATP5ME |  |
|  | MYL5 |  |
